# From shops to bins: a case study of consumer attitudes and behaviours towards plastics in a UK coastal city

**DOI:** 10.1007/s11625-022-01261-5

**Published:** 2023-01-30

**Authors:** Stephanie Lucy Northen, Laura Karoliina Nieminen, Serena Cunsolo, Steven Kator Iorfa, Keiron Philip Roberts, Stephen Fletcher

**Affiliations:** 1 Global Plastics Policy Centre, University of Portsmouth, Portsmouth, UK; 2 School of the Environment, Geography and Geosciences, University of Portsmouth, Portsmouth, UK; 3 School of Civil Engineering and Surveying, University of Portsmouth, Portsmouth, UK; 4 Portsmouth Business School, University of Portsmouth, Portsmouth, UK

**Keywords:** Single-use plastics, Recycling rate, Household waste generation, Sustainable consumption, Circularity, Consumer perceptions

## Abstract

Understanding the use behaviours of plastic items within households is important to enable informed policy development, particularly with the emerging and developing global plastic treaty. A survey of 400 permanent residents in Portsmouth aimed to identify the general trends in single-use plastic product (SUPP) use and disposal, and their personal motivations and barriers to reducing and recycling plastic. This included identifying common influencers of attitudes such as environmental values, situational characteristics, psychological factors and the individual demographic characteristics of residents. Key factors in consumer behaviour were found to be product availability, affordability and convenience. Often, less conveniently recycled plastics more frequently end up in landfill such as films, shopping bags and personal care items. The age of respondents was found to be the most significantly associated demographic with SUPP consumption, reuse and recycling behaviours. Other demographic variables such as a resident’s location within the city, income and vehicle ownership were potential drivers influencing individual attitudes and their incentives towards reducing and recycling their plastic waste. The findings from this study brought to light the importance of effective local plastic governance. This study also identified consumer perceptions and behaviours that could contribute to future holistic plastic policy recommendations.

**Supplementary Information:**

The online version contains supplementary material available at 10.1007/s11625-022-01261-5.

## Introduction

### Background

Plastics are ubiquitous with approximately 4.9 million metric tonnes of plastic produced for the United Kingdom (UK) market annually (Tiseo 2021). The volume of mismanaged plastic waste is increasing, with implications for human health and nature (Jambeck et al. 2015; Mason et al. 2018; Welden 2020). Since plastic manufacturing began in the 1950s, it has been estimated that approximately 4.9 billion tonnes (60%) of all the plastic produced has ended up as pollution. This waste is accumulating in landfills and the environment (as pollution), and only 10% of all plastic waste generated has been recycled (Geyer 2020; Geyer et al. 2017). In this paper, we briefly discuss single-use plastic product (SUPP) consumption and waste generation in the UK and consider some of the environmental impacts that plastic pollution and waste has on the environment. Here, we present a survey case study we conducted in Portsmouth and our findings regarding the trends in SUPP consumption, demographic influences, and plastic avoidance behaviours (PABs) and waste disposal attitudes (WDAs) of Portsmouth residents. Finally, we discuss the implications of our findings for future research and policy priorities to reduce the consumption of SUPPs.

Some of the drivers of plastic consumption and utilisation have been identified as environmental values, situational characteristics and psychological factors that can be used to predict purchase behaviour and waste management intentions (Barr 2007). The disposability of plastics and its average residence time within households is often dictated by availability, affordability and convenience (O’Brien and Thondhlana 2019). In 2017, 2.4 million tonnes of plastic were sold in the UK, 1.3 million of which was used for plastic packaging alone, which is often a highly discarded and single-use material in retail and hospitality (Burgess et al. 2021; WRAP 2018). The most littered items in the UK are bottles, bags and single-use food wrappers (Ocean Conservancy and International Coastal Cleanup 2017). The UK government in 2018 set out a plan to ban the sale of certain SUPPs such as plastic straws, drink stirrers, cups and plastic stemmed cotton buds, which entered into force in April 2020 (DEFRA 2019). The UK, along with other nations, believed that restricting the sale of some of the most frequently littered SUPPs would lead to a gradual phase out of all unnecessary SUPPs (DEFRA 2018). However, since the introduction of a plastic bag charge in 2015, the UK has seen little progress in effectively phasing out other SUPPs in legislation (WRAP 2020b).

Studies of SUPP reuse tend to focus mainly on plastic bags (Van Rensburg et al. 2020; Liu et al. 2021). Before a five pence plastic bag charge in the UK, 55% of consumers used plastic bags from supermarkets, which fell to 22% within 6 months of the introduction of the charge (Adeyanju et al. 2021; Thomas et al. 2019). The COVID-19 pandemic subsequently increased the purchase, disposal and litter of plastic packaged items and personal protective equipment (Roberts et al. 2022; Khan et al. 2020; Kitz et al. 2022; Vanapalli et al. 2021; Sharma et al. 2020; Silva et al. 2021; WRAP 2020a).

It is estimated that within the UK, 1.53 million tonnes of plastic waste was produced in 2016 from all sectors, with household waste contributing 8% of this figure (Smith 2022). The latest plastic waste arisings data for the UK show an increase of 24% between 2010 and 2016 (Smith 2022). If this rate of plastic waste increase continues, the UK is expected to produce 6.3 million tonnes of plastic waste per year by 2030. Plastic packaging will make up approximately two-thirds of the waste (Smith 2022). The UK exports approximately 40% of its plastic waste to Turkey and Southeast Asian countries annually for disposal or to be recycled (Tiseo 2021; Zhao et al. 2021), yet it is unclear if these countries have sufficient waste management capacity to deal with this additional waste (Lebreton and Andrady 2019). Up to 12.7 million tonnes of mismanaged plastic waste enters the oceans annually according to estimates from 2010, with 19–23 million tonnes predicted to enter aquatic ecosystems annually, equivalent to 11% of the global plastic waste produced (Bergmann et al. 2022; Borrelle et al. 2020; Jambeck et al. 2015; The Pew Charitable Trusts and SYSTEMIQ 2020). Plastic waste enters aquatic ecosystems through a number of different pathways including sewage effluents, surface runoff and groundwater flow. This eventually gets carried into rivers and oceans when unmanaged, particularly during storms or extreme weather conditions in coastal areas. This is a threat to marine life in various ways, ranging from entanglement in plastic items to plastic ingestion (Welden 2020). Moreover, plastic manufacturing is an emission-intensive process which exacerbates climate change impacts in the oceans (Center for International Environmental Law 2019; Shen et al. 2020).

As the awareness of the impacts of plastic waste and pollution on the environment and public health grows, the urgency of switching to more sustainable alternative materials and holistic interventions in plastic governance globally is clear. The recent United Nations Environment Assembly (UNEA-5.2) draft resolution, *End plastic pollution: Towards an international legally binding instrument* (UNEP 2022a, b; IUCN 2022), commits to develop a global plastics treaty by 2024 to substantively transform plastic economies and consumer behaviour towards a more sustainable and circular plastics economy.

A circular economy approach is often offered as a solution to reducing the impacts of plastics. The principles of a circular economy focus on the importance of cutting plastic production through reducing, reusing and refusing practices (Crippa et al. 2019). Although the global effort to participate in recycling practices is still supported for having some sustainable potential in contributing to the transition towards a circular economy and closing the loop on plastic pollution, there is still debate around the limitations of recycling in the current literature (Geyer et al. 2016). Recycled and recyclable products are more sustainable than producing and using virgin or unrecyclable plastic. However, recycling only reduces waste generation if it reduces primary material production; otherwise, the end pathway of the plastic is only delayed. Effective and well-managed recycling practices are not universal and adaptable to all nations’ different capacities across countries and are therefore not always a long-term viable solution. Recycling plastic indefinitely is not always recommended due to other environmental impacts involved in the process and that many types of plastic lose structural integrity and their potential to be reused after multiple recycling processes (Geyer et al. 2016; Bucknall 2020). Recyclability has become a convenient label on products by producers and retailers as a marketing tool; however, the reality of the complexity of processing different plastic materials in different areas of the UK alone is not as simple, and this often becomes misleading or ‘greenwashed’ messaging. This study will examine if any of these discussed considerations on plastic reuse and recycling perceptions and practices are similar amongst the findings in Portsmouth’s survey respondents.

### Case study: Portsmouth, UK

**Fig. 1:**
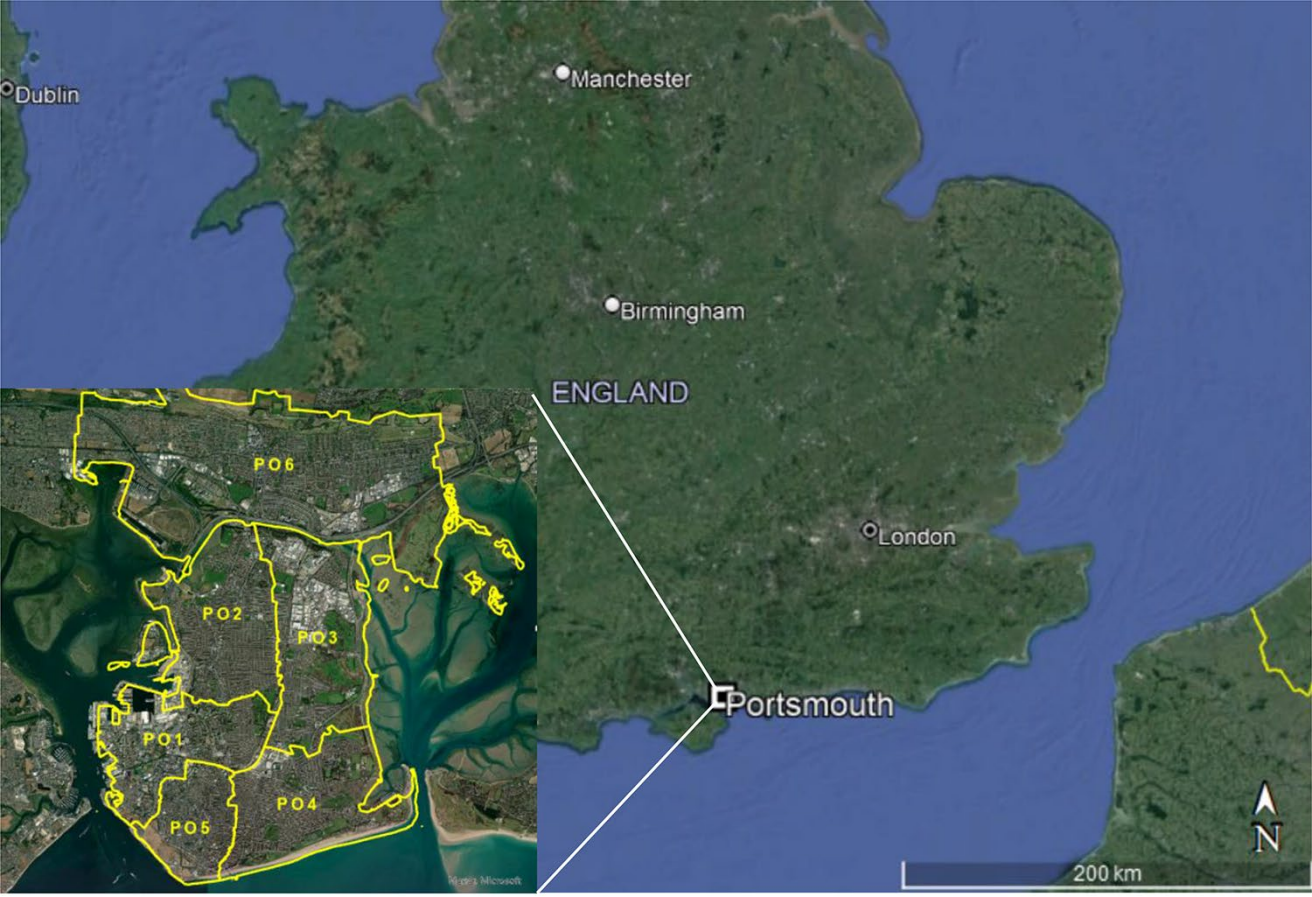
Map of the UK case study city (Portsmouth) showing the location of survey area postcodes (PO1–PO6)

Portsmouth is a densely populated coastal city on the South Coast of England, UK with 5315 people per sq. km in 2020 (Office for National Statistics 2021). This makes Portsmouth the second most densely populated city in the UK after London (5727 people per sq. km in 2020; Office for National Statistics 2021). Portsmouth is the UK’s only island city (Fig. 1). The immediate proximity of the city to the ocean carries multiple pollution risks including the erosion of historic landfill sites and beach litter. Due to its geographic limitations as an island and population density, Portsmouth has a limited recycling kerbside collection system. Currently, only paper, card, plastic bottles, metal cans, tins and aerosols are recycled (Portsmouth City Council 2022). The current recycling rate for Portsmouth is 24.7%, one of the lowest in the UK and considerably lower than the national average of 46.2% (Letsrecycle 2021; DEFRA 2021). Most other UK councils on average collect 54% of ‘rigid’ plastics (i.e. drinks and detergent bottles) and 4% of plastic consumer films (i.e. bags, sachets and wraps) (Burgess et al. 2021). However, Portsmouth has one of the lowest landfill rates in the UK, with only 4.2% of total collected waste going to landfill, with the majority of waste incinerated (Portsmouth City Council 2022).

England’s Waste Strategy previously set a target to reuse, recycle and compost 45% of household waste by 2015 and 50% by 2020 (DEFRA 2018; Timlett and Williams 2009). Portsmouth was not amongst the 22% of UK councils that met this target (Letsrecycle 2021). The total amount of plastic sent to a Materials’ Recovery Facility (MRF) in the southeast of England was greater (approximately 14,000 t) than any other area in the UK (< 8000 t) in 2012–2014 (Hahladakis et al. 2018). Approximately 50,000 t of all waste from Portsmouth and the surrounding towns is sorted for recycling at the MRF per year and 200,000 t is incinerated at the Energy Recovery Facility (Callingham 2020). However, there are no data on the plastic content of those wastes. A waste composition analysis for the Portsmouth City Council found that, in 2018, approximately 14% of waste from recycling bins was plastic, of which nearly 4% were “non-target plastics” such as plastic tubs, pots and trays that are not included in the current recycling collection scheme (Integra 2019).

As both a coastal and the second most densely populated city in the UK, Portsmouth can provide critical insights into the role of plastics in everyday life, including the challenges of managing plastic waste in a densely packed urban setting. Previous research on consumer attitudes, behaviours and flows of plastic through systems in cities similar to Portsmouth formed the basis for our research questions (RQ) (Barr 2007; Van Rensburg et al. 2020; Walker et al. 2021; Varkey et al. 2021). Our research provides an opportunity to explore this topic to better understand how households in Portsmouth operate in their plastic-saturated daily lives. We aimed to determine trends in household plastic purchase, reuse and disposal habits and approaches in Portsmouth. We identified the demographic factors that influence plastic-related consumer behaviour and perceptions and behaviours around plastics in general. Finally, we determined key PABs and WDAs of Portsmouth residents relevant to local businesses, waste management and recycling services. We set our RQ as follows:

RQ1: *What are the trends in SUPP flow through Portsmouth households?*

RQ2: *Which demographic factors influence SUPP trends and consumer attitudes and behaviours?*

RQ3: *What are the dominant PABs and WDAs in Portsmouth?*

## Materials and methods

### Survey method and questionnaire

We collected survey data on household attitudes and behaviours towards plastic items from 400 Portsmouth residents using a questionnaire between June and July 2021. Red Brick Media Company Ltd (a commercial survey company) sourced the respondents from general public research panels filtered by Portsmouth postcodes. Survey participants were incentivised using a points-based reward system. The pool of 1796 Portsmouth respondents was either qualified or disqualified based on survey completion and their responses checked for validity and quality. This way, the survey company was able to disqualify incomplete survey responses, respondents that “flat-lined” through the survey to get points, and responses with contradicting answers. The final sample (*n*) of 400 qualified and fully completed survey responses were weighted against the most recent 2020 census data based on age and gender distributions in the population in Portsmouth. Based on a power analysis, to have a confidence interval (CI) of 95% with a ± 5% margin of error for the results, minimum sample size was calculated at 317 respondents for a population of 1796.

**Fig. 2:**
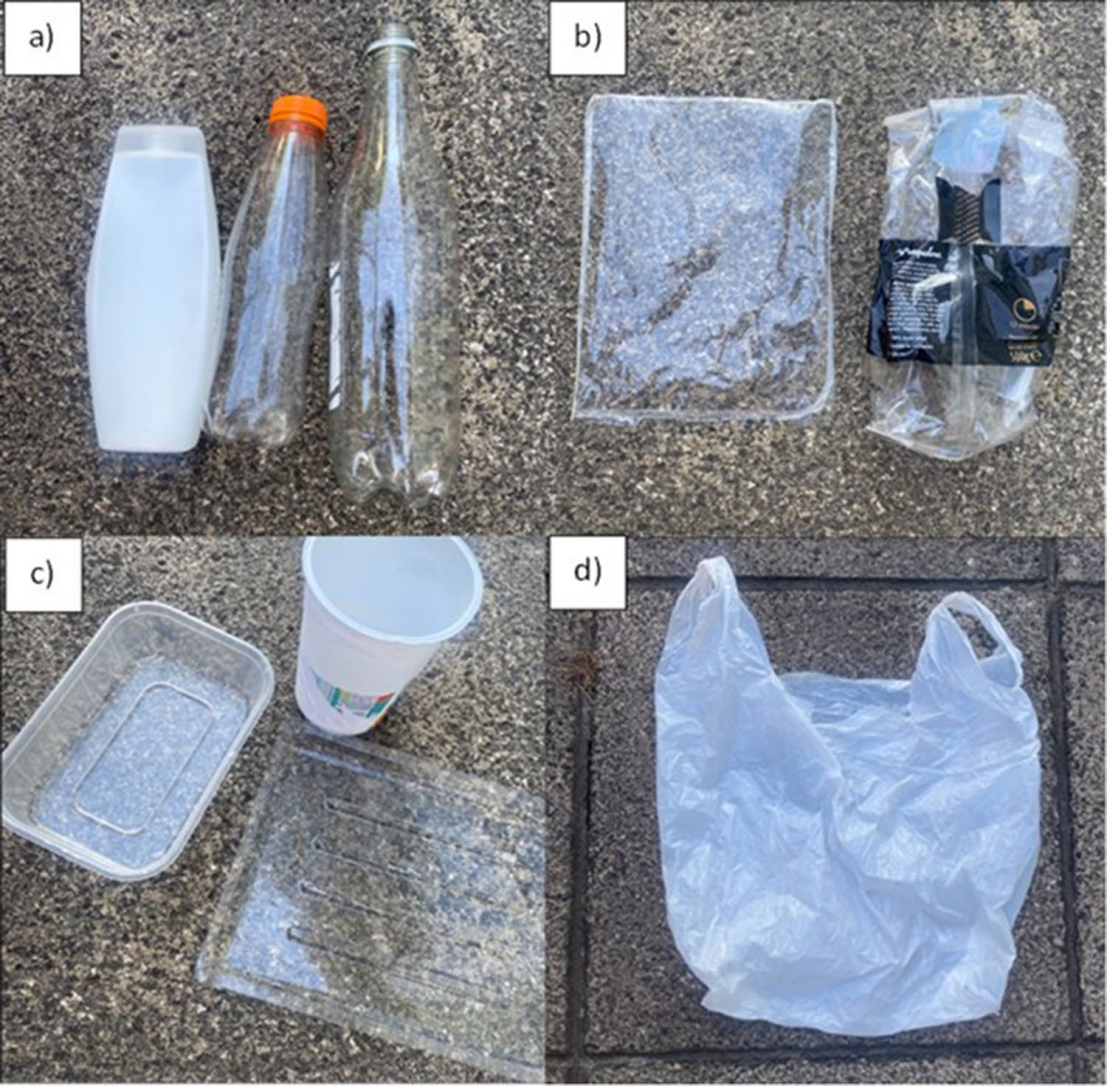
Image examples of the different types of single-use plastics included in this survey: **a** plastic bottles, **b** plastic films, **c** plastic tubs, **d** plastic bags. See Table 1 for further classifications. Source: Authors' conception

In addition to social demographics (‘age’, ‘gender’ and ‘postcode’), economic demographics (‘income’, ‘education’, ‘living situation’ and ‘vehicle ownership’) were mapped in the survey (see the key for factor levels in SI2). The five subscales of the questionnaire were SUPP consumption (purchase hereafter), usage (reuse hereafter), disposal and plastic-related attitudes, perceptions, and behaviours (see SI1) in relation to four types of SUPPs (bags, bottles, films and tubs) (Table 1; Fig. 2). We mapped SUPP purchase and reuse based on a weekly average per household and how they disposed of their SUPPs once they become waste. Regarding attitudes and perceptions, we asked respondents about their knowledge on climate action, marine litter, and the impact of an individual’s actions. In addition, we asked respondents a selection of Portsmouth-specific questions relating to local matters such as littering and awareness of local zero-waste shops (see the full list of questions in the SI1).

**Table 1:** Definitions for the SUPP items used in the survey questions (see SI1)

SUPP item	Includes
Plastic bottles	Bottles, e.g. for beverages, personal hygiene and cleaning products
Plastic film	Food wrapping and other thin packaging, e.g. salad, frozen foods, multi-pack tins
Plastic tubs	Tubs and pots, e.g. yogurt pots, fruit punnets, meat and fish packed in plastic trays
Plastic bags	Single-use shopping bags

### Data handling and analysis

The majority of the data collected were Likert-type, i.e. statements or questions with neither polar opposites nor neutral middles in the answer scale (see SI1). Due to the exploratory nature of our study (to explore which demographics and socio-economic variables have the widest impact on consumer behaviour towards SUPPs), we used simple Pearson’s Chi-squared tests for independence to identify impactful demographics in relation to SUPP purchase, reuse, and disposal. The postcodes included in the analysis were specific to Portsmouth, UK. Where the cell size of the sample was too small for Pearson’s Chi-squared tests for independence, we performed Monte Carlo simulations of the *p* values, i.e. Pearson’s Chi-squared coefficients (see R code in SI2). For any test with Monte Carlo simulation, the degrees of freedom (*df*) are not reported by convention. All statistical analyses were performed in R (R Core Team 2022) and with the CI of 95%. For the R packages used, please see Supplementary Information (SI2). As the main foci in our analysis were the attitudes and behaviours regarding the purchase, reuse, and disposal of four types of SUPPs, we used descriptive statistics to present the population of respondents (Table 2), the purchase and reuse phases of the SUPP household flow and the SUPP-related waste disposal behaviours (WDBs), the PABs and the WDAs of the respondents.

**Table 2:** Percentage of the proportion of respondents across the key demographic and socio-economic groups

Demographic subject	Group	% of respondents	Socio-economic factors	Group	% of respondents
Age (years)	30 and under	22	Income (£GBP)	< 25,000	38
	31–50	42		25,000–49,999	33
	51 and over	36		50,000 +	22
Gender	Male	48.5	Vehicle ownership	None	30
	Female	50.7		1 +	70
	Other	< 1			
Postcode	PO1	17	Education	None	0
	PO2	17		Primary	< 1
	PO3	13		Secondary	25
	PO4	21		High school/college	32
	PO5	18		University/higher education	33
	PO6	16		Postgraduate	9

## Results

### Sample demographics

All of the respondents were permanent residents of Portsmouth, and more than half (57%) have lived in Portsmouth for more than 20 years. Most respondents were either 31–50 years old (42%) or 51 years old and over (36%). The numbers of females, males and people representing other genders were 203, 194 and 3, respectively. The education level of most respondents was secondary school or above (74%). The majority owned their current residence (56%). The average household income was less than GBP 25,000 (38%) followed by GBP 25,000–49,999 (33%). The majority (70%) of Portsmouth households own one or more vehicles (i.e. motorbike, car or van) (Table 2).

### SUPP trends in Portsmouth households

**Fig. 3:**
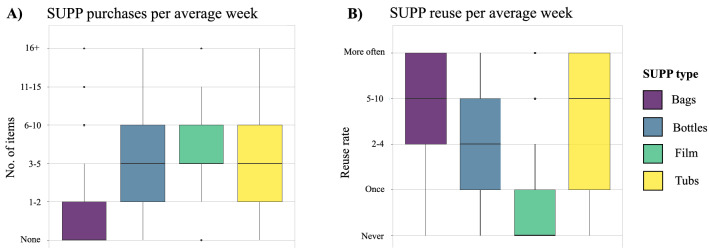
Consumer behaviour and SUPPs. **A** SUPP purchasing behaviour per average week in Portsmouth households, **B** SUPP reuse behaviour in Portsmouth households

The median weekly purchase rates for the four SUPP types were ‘none’ (bags) and 3–5 (bottles, films, and tubs; Table 3; Fig. 3A). The majority (61%) of respondents did not purchase any plastic bags in an average week, 89% in total bought 0–5 bags, and only 5% bought either between 6 and 10 or more than 11 bags per week. 61% of respondents bought between 0 and 5 plastic bottles in an average week, 18% bought 6–10 and 8% bought more than 11 bottles. Plastic tubs were often bought in quantities of 0–5 per week (66%), 21% purchasing 6–10 and 10% more than 11 tubs. Respondents bought plastic films more frequently at a rate of 0–5 per week (54%), or over 6 times per week (42%). The median weekly reuse rate for the measured SUPPs were 5–10 times (bags), 2–4 times (bottles), ‘never’ (films) and 5–10 times (tubs; Table 3; Fig. 3B). Respondents often used SUPPs more than 5 times before disposing of them, with most respondents reusing bags more frequently (58%), followed by tubs (48%), bottles (33%) and then rarely films (7%). 60% of respondents never reused film SUPPs.

**Table 3:** The percentage of respondents that consume and reuse different amounts of the SUPP items surveyed

SUPP item	Number of items bought	% of respondents	Number of reuses	% of respondents
Plastic bottles	0	4	Never	19
	1–2	34	Once	17
	3–5	33	2–4 times	31
	6–10	18	5–10 times	14
	11 +	8	More often	19
Plastic films	0	3	Never	60
	1–2	21	Once	20
	3–5	30	2–4 times	13
	6–10	28	5–10 times	3
	11 +	14	More often	4
Plastic tubs	0	3	Never	17
	1–2	28	Once	11
	3–5	35	2–4 times	24
	6–10	21	5–10 times	13
	11 +	10	More often	35
Plastic bags	0	61	Never	8
	1–2	18	Once	11
	3–5	10	2–4 times	23
	6–10	5	5–10 times	18
	11 +	5	More often	40

Any unaccounted-for respondents from the sample total responded to these questions with the response “don’t know”

**Fig. 4:**
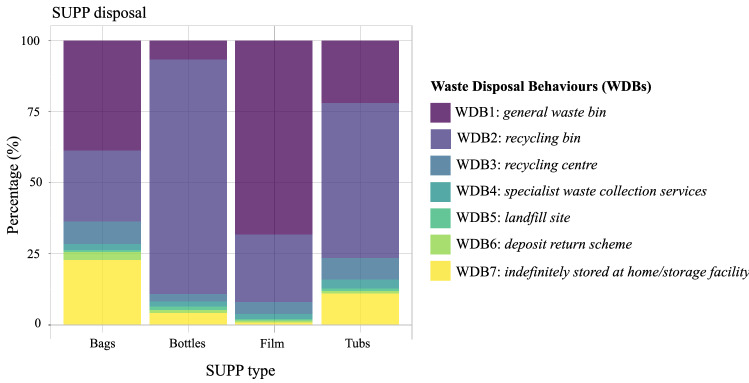
Waste disposal behaviours (WDBs) of households regarding SUPPs

The most common methods of plastic disposal were in a general waste bin (WDB1), a recycling bin (WDB2) and indefinite storing (WDB7; Fig. 4). The SUPP items with the highest household recycling rates (including domestic or public recycling bins (WDB2) and recycling centres (WDB3) were bottles (85%) and tubs (61%). The least recycled SUPPs were bags and films with household recycling rates of 30% and 27%, respectively. Figure 4 also shows the practice of “wish-cycling” (Somerville 2017), whereby non-recyclable SUPPs are put in recycling bins by consumers. The use of specialist waste collection services, landfills sites or deposit return schemes was not common and, combined they accounted for approximately only 3–5% per each SUPP type (Fig. 4). Although some personal care items such as makeup containers, disposable razors, cotton buds and toothpicks can be considered as SUPP items due to their low likelihood of reuse, we did not analyse them in the same detail. However, we found that most personal care items were disposed of permanently rather than recycled, with 76% of the personal care items ending up in landfills.

### Socio-economic factors and demographics of significance

#### SUPP flow through Portsmouth households

We found that the most significant demographics with the widest impact on plastic usage were age and gender, with each impacting 10 and 6 aspects of SUPP flow through Portsmouth households, respectively. Age significantly impacted the purchase rate of bottles, films, and bags (*p* < 0.05, Table 4). Age also significantly impacted the reuse of tubs, films, and bags, as well as the disposal of all four types of SUPPs (*p* < 0.05, Table 4). For example, 85% of respondents aged 51 years and above purchased zero plastic bags in an average week, compared to 39% of the youngest age group (≤ 30 years). The impact of gender was significant (*p* < 0.05, Table 4) on the purchase of bottles, the reuse of tubs and the disposal of all four types of SUPPs.

**Table 4:** Comparison of the different demographic factors amongst the respondents to find if these show any significant relationships (**bold** = *p* value < 0.05, *⍺* = 0.05) between the consumer behaviour questions concerning ‘How many of these SUPPs do you purchase, reuse, and dispose of per week?’ using a Pearson’s Chi-squared test for independence

SUPP consumer behaviour	SUPP type	Demographics (*p* values)		
		Age	Gender	Postcode
Purchase	Bottles	**0.0040**	**0.0200**	**0.0045**
	Tubs	0.2209	0.0780	0.2729
	Films	**0.0260**	0.1419	**0.0395**
	Bags	**< 0.001**	0.0900	0.1974
Reuse	Bottles	0.3916	0.2839	0.6453
	Tubs	**0.0392**	**0.0340**	0.2803
	Films	**< 0.001**	0.1139	0.2944
	Bags	**0.0411**	0.1954	0.0625
Disposal	Bottles	**0.0280**	**< 0.001**	0.7456
	Tubs	**0.0080**	**0.0410**	0.4328
	Films	**0.0105**	**0.0480**	0.7376
	Bags	**0.0190**	**0.0255**	0.**0085**

See full results in SI3 and SI4, and Survey Questions in SI1

Education, living situation and postcode impacted 3 aspects of SUPP consumer behaviours. Education significantly impacted the reuse of films and the disposal of bottles and films (*p* < 0.05, Table 5). Living situation significantly impacted the purchase of films and bags and the reuse of bags (*p* < 0.05, Table 5). The impact of postcode was significant (*p* < 0.05, Table 4) on the purchase of bottles and films and the disposal of bags. Both income and vehicle ownership impacted two aspects of SUPP consumer behaviour. The impact of income was significant (*p* < 0.05, Table 5) on the purchase of bottles and the disposal of films, and vehicle ownership significantly impacted the purchase and disposal of bottles. None of the tested demographics or socio-economic factors had a significant impact on the consumer behaviour around purchase of plastic tubs or on the reuse of plastic bottles (see SI3 and SI4 for full results).

**Table 5:** Comparison of the different socio-economic variables amongst the respondents to find if these show any significant relationships (**bold** = *p*-value < 0.05, *⍺* = 0.05) between the consumer behaviour questions concerning ‘How many of these SUPP products do you purchase, reuse, and dispose of per week?’ using a Pearson’s Chi-squared test for independence

SUPP consumer behaviour	SUPP type	Socio-economic factors (*p* values)			
		Education	Income	Living situation	Vehicle ownership
Purchase	Bottles	0.6787	**0.0470**	0.2249	**0.0195**
	Tubs	0.4633	0.1989	0.0950	0.5882
	Films	0.9170	0.5072	0.0090	0.2304
	Bags	0.7341	0.1599	**0.0215**	0.4343
Reuse	Bottles	0.3673	0.2649	0.3268	0.5123
	Tubs	0.1994	0.2954	0.5542	0.3713
	Films	**0.0100**	0.1394	0.1064	0.9545
	Bags	0.9675	0.4808	**0.0060**	0.8321
Disposal	Bottles	**0.0270**	0.1199	0.1839	**0.0125**
	Tubs	0.3623	0.4373	0.1509	0.1684
	Films	**0.0415**	**0.0480**	0.6002	0.7571
	Bags	0.2309	0.3293	0.1209	0.1304

See full results in SI3 and SI4, and Survey Questions in SI1

#### Consumer perceptions

With regard to consumer perceptions of plastic, we found age to have the widest impact with significant differences (*p* < 0.05, Table 6) in responses of three out of five of the perception-related test statements, namely ‘*awareness of zero-waste shops in Portsmouth*’, ‘*concerned that plastic waste ends up in the ocean*’ and ‘*main consideration when buying products or items*’. Respondents aged 31–50 years were more regular at shopping in Portsmouth zero-waste shops than their counterparts, while the oldest age group (> 50 years) reported being less aware and less willing to shop in Portsmouth zero-waste shops. Without accounting for age, the majority of respondents were not aware of any zero-waste shops in Portsmouth (*n* = 175, 44%) but would like to use them. Whereas 22% (*n* = 88) said they were aware of zero-waste shops but have never visited one, and 17% (*n* = 69) of the respondents said they were not aware and are not likely to use them. Occasional and regular customers of Portsmouth zero-waste shops comprised 8% (*n* = 30) and 4% (*n* = 14) of all respondents, respectively.

**Table 6:** The results from the Pearson’s Chi-squared tests on the demographic factors of the respondents and the following barriers to reducing their purchase and waste of plastic items

	Demographics (*p* values)		
	Age	Gender	Postcode
*Consumer perceptions*			
Awareness of zero-waste shops in Portsmouth (C60)	**< 0.001**	**0.0325**	**0.0035**
Belief in the power of individual actions (A30)	0.6372	0.1994	0.1764
Concerned that plastic waste ends up in the ocean (W40)	**0.0014**	0.1139	0.9535
Littering is a serious issue in Portsmouth (W50)	0.2394	0.2469	0.2704
Main consideration when buying plastic products or items (C30)	**0.0014**	0.5482	0.6497
*Consumer behaviour*			
Attitude towards plastic (C40)	0.6261	0.3808	0.6122
Attitude towards waste disposal (W30)	**0.005**	0.5912	0.5342
Barriers to recycling plastic (W80)	**< 0.001**	0.3453	0.7646
Barriers to reducing plastic (C180)	**< 0.001**	0.3263	0.4018
Choosing non-plastic over plastic (C50)	0.5421	0.2294	0.1944

Significant results (*p* value < 0.05) shown in bold. See the survey questions the statements refer to in SI1, Sections 2 and 4; Plastics Consumption and Waste Disposal, Questions C60, A30, W40, W50, C30, C40, W30, W80, C180, and C50

Younger respondents (≤ 30 years) were more concerned about plastic waste entering the ocean than their older counterparts (> 50 years). Overall, the concerns around how often plastic waste ends up in the ocean were distributed along the scale of always (5%, *n* = 19), most of the time (26%, *n* = 104), sometimes (50%, *n* = 200), rarely (15%, *n* = 60) and never (4%, *n* = 17). Regarding the main considerations when buying plastic products or items, price was more important as a purchasing consideration to respondents aged ≤ 30 years and 31–50 years, quality was a more predominant consideration to respondents aged > 50 years. Other significant differences within the main purchasing considerations by order of most likely age groups were sustainability (31–50 years) and ethics (≤ 30 years). Overall, value for money was the most important consideration (30%, *n* = 119), followed by price (24%, *n* = 97) and quality (22%, *n* = 22).

#### Consumer behaviours

Age was the demographic with the greatest impact on consumer behaviour with significant differences between different age groups (30 years and under, 31–50, and 51 and over) (*p* < 0.05, Table 6) in three out of five of the behaviour-related test statements, namely ‘*attitudes towards waste disposal*’, ‘*barriers to recycling plastic*’ and ‘*barriers to reducing plastic*’. Attitudes towards waste disposal consisted of a scale from 1 (“*I don’t really think about what happens to my waste once it is out of my hands*”) to 7 (“*I am very concerned about where my waste ends up and what impact it has on the environment*”). Older respondents (> 50 years) were more concerned about where their waste ends up compared to the other age groups. Overall, the majority of respondents (29%, *n* = 114) were extremely concerned about the final destination of their waste.

Regarding the barriers to recycling plastic, respondents aged ≤ 30 years found unclear information, forgetting to recycle and disagreements within households as their main barriers. Respondents aged 31–50 found the lack of both local plastic recycling facilities and local support as their main barriers. The oldest age group (> 50 years) stated limited council collection as their main barrier as well as stating that they already recycle everything they can. Overall, collection practices (29%, *n* = 115) and unclear recycling information (16%, *n* = 62) were the key barriers to recycling more plastic waste amongst respondents.

The main barrier to reducing plastic consumption for the youngest age group (≤ 30 years) was limited functionality of plastic alternatives, while a few (*n* = 6) respondents in the same age group stated that reducing plastics is not important. The higher price of plastic alternatives was the main barrier for reducing plastic for respondents aged 31–50. While stating limited availability of plastic alternatives as their barrier, the oldest age group (> 50 years) felt that there were no barriers for them to reduce plastic consumption compared to the other age groups. In general, limited availability of alternatives to SUPPs was the main barrier identified (23%, *n* = 90), followed by their preferred products not being available plastic-free and alternatives are too expensive (21%, *n* = 85 for each). Another wide impact demographic we found was vehicle ownership with significant differences in two out of five test statements (*p* < 0.05, Table 7), namely ‘barriers to recycling plastic’ and ‘barriers to reducing plastic’. Households with no vehicles experienced difficulty in transporting their non-collected recyclables as their key barrier to recycling more of their plastic waste. Households with one or more vehicles did not have distinctive key barriers to recycling their plastic waste more.

**Table 7:** The results from the Pearson’s Chi-squared tests on the socio-economic variables of the respondents and the following barriers to reducing their purchase and waste of plastic items

	Socio-economic variables (*p* values)			
	Education	Income	Living situation	Vehicle ownership
*Consumer perceptions*				
Awareness of zero-waste shops in Portsmouth (C60)	**0.0300**	**0.0085**	0.4043	0.5347
Belief in the power of individual actions (A30)	0.5307	0.3373	0.5167	0.5072
Concerned that plastic waste ends up in the ocean (W40)	0.7526	0.6192	**0.0445**	0.6923
Littering is a serious issue in Portsmouth (W50)	0.6437	0.0500	0.1009	0.0560
Main consideration when buying plastic products or items (C30)	0.1124	0.1389	0.3653	0.2719
*Consumer behaviour*				
Attitude towards plastic (C40)	0.4368	0.1224	0.2029	0.3387
Attitude towards waste disposal (W30)	0.1394	0.0890	0.1059	0.8484
Barriers to recycling plastic (W80)	0.3403	**0.0255**	0.5692	**0.0270**
Barriers to reducing plastic (C180)	0.1999	0.2009	0.1049	**0.0065**
Choosing non-plastic over plastic (C50)	0.1979	0.2259	0.3808	0.1154

Significant results (*p *value < 0.05) shown in bold. See the survey questions the statements refer to in SI1, Sections 2 and 4; Plastics Consumption and Waste Disposal, Questions C60, A30, W40, W50, C30, C40, W30, W80, C180, and C50

#### PABs and WDAs

**Fig. 5:**
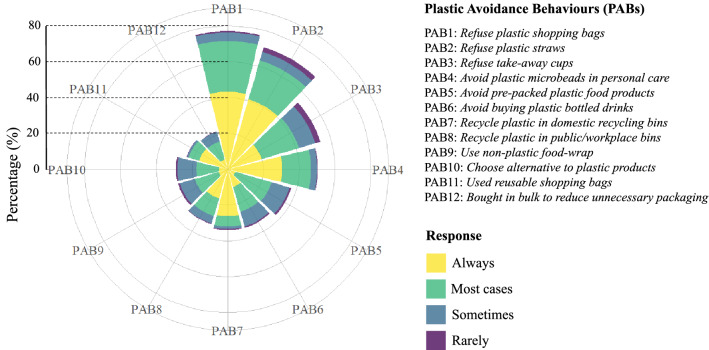
A rose chart showing the respondent’s PABs and how often they were expressed by the respondents (count data) based on a series of multiple-choice questions

One in four survey respondents will go out of their way to avoid SUPPs in everyday purchases, whereas the majority (54%) will only avoid SUPPs if an alternative option is readily available. Respondents (93%) most frequently avoided plastic shopping bags (PAB1; Fig. 5). Eighteen percent of respondents expressed PAB2 (refusing plastic straws) as one of their avoidance behaviours, with 57% of them saying they ‘always’ refuse plastic straws (Fig. 5). Some respondents reported buying in bulk to reduce excess plastic packaging (5%) and to use reusable shopping bags less frequently (6%). Only 8% of respondents stated that they use their domestic recycling bins. Again, only 8% reported using public/workplace recycling bins to dispose of their recyclable plastic waste (Fig. 5). The other frequently selected PABs were refusing take-away cups (14%) and avoiding personal care items containing plastic microbeads (13%; Fig. 5).

**Fig. 6:**
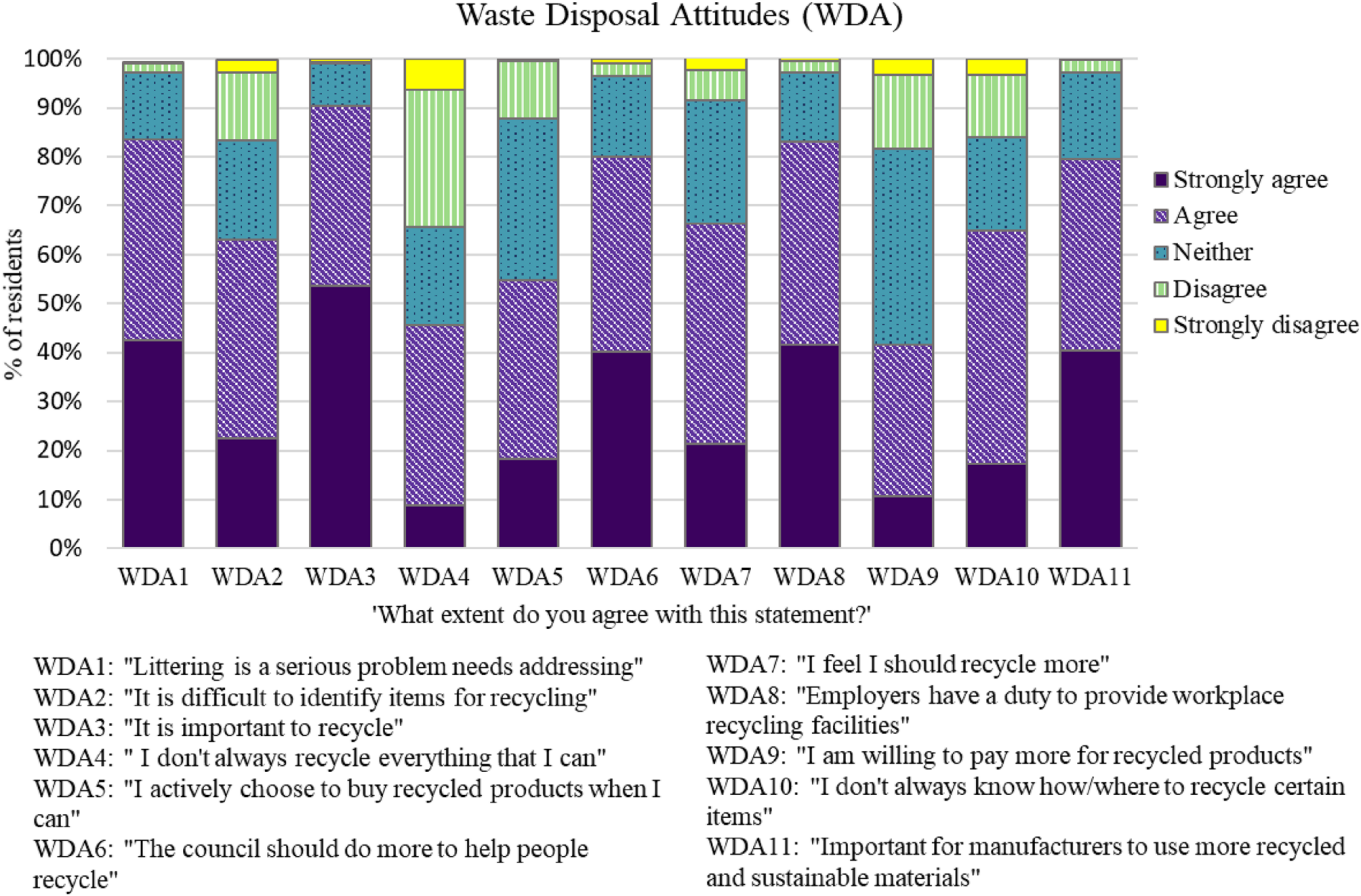
The percentage (%) of the respondents as to what extent they agree or disagree with the WDAs statements listed

We found that 65% of respondents often do not know how or where to recycle plastic items (Fig. 6). While most respondents agree (90%) that it is important to recycle (WDA3) and that littering is a serious problem that needs addressing (83.5%) (WDA1), many admit that they should do more (WDA7) to recycle (65%) (Fig. 6). Respondents agree that employers have a duty to provide recycling facilities in their workplace (83%) and that it is important to them that manufacturers use more recycled and sustainable materials in products (79.5%). The barriers identified by respondents to reducing their plastic intake were the price of alternative products (20%), difficulties in availability of alternatives (23%) and that their preferred products are not plastic-free (22%). The three major barriers to recycling highlighted by respondents were due to the council not collecting all items (35%), difficulties in knowing what and how to recycle (17%) and the belief that there are not enough local recycling facilities in Portsmouth (10%).

Portsmouth survey respondents then identified potential incentives that might encourage them to recycle more in the future. The most dominant incentive identified was for the council to collect more types of plastic for recycling (59%). Where SUPP is easily identified as recyclable and respondents know what can and cannot be recycled (43%), then the vast majority indicated that they would make the choice to recycle them (79%). The other key incentives that were identified by respondents were having the availability of recyclable products (33%) and local recycling centre information (31%). Expansion of recycling collection facilities in the city (30%), shopping centres (28%), places of work (17%) and local events (20%) would enable improvements in recycling behaviour. Currently, ≤ 3% of respondents use cash back or deposit schemes for recycling all the SUPP types in Portsmouth, but 40% said that they would be encouraged to use these if available locally.

## Discussion

### SUPP purchase and reuse in Portsmouth households

Plastic bags were rarely purchased by Portsmouth respondents, while the median rates for purchasing products in plastic bottles, packed in plastic film or plastic tubs were alike (RQ1; Fig. 3A). A study in South Africa found that the purchase rate of SUPP bags amongst the majority of Durban beachgoers was < 5 per week (48%; Van Rensburg et al. 2020), which is significantly higher than Portsmouth respondents. Varkey et al. (2021) found that a minority (3%) of their respondents in the coastal city of Halifax, Canada, used SUPPs once a month or less. However, it was not clear whether the “use of SUPPs” metric in their survey distinguished between the purchase and the reuse rates of SUPPs. Most Portsmouth respondents (approximately 80%) used SUPPs on a daily to weekly basis. Reuse rates amongst Portsmouth respondents were the highest for bags and tubs (RQ1; Fig. 3B). In Hanoi, Vietnam, approximately 69% of plastic shopping bags had a “high rate of temporary reuse” and they were most often reused as bin liners (Liu et al. 2021).

In Durban, a majority of their respondents reused all of their SUPP bags (42%) or reused some of them and threw the rest away (27%; Van Rensburg et al. 2020). The weekly reuse rate per bag was not part of their survey. Film packaging was rarely reused by Portsmouth respondents, which may be due to the flimsy nature of plastic film packaging and due to the limited ways in which it can be reused in its current form. Portsmouth respondents are already reusing plastic products that were intended for single use such as bottles, tubs and bags. Ertz et al. (2017) reiterated the importance of providing more plastic reuse than single-use options and making reusable containers more attractive than the perceived convenience of the SUPPs. Overall, SUPP trend studies are limited and highly variable with no uniform metrics. Quantification of SUPP purchase and reuse rates separately, as used by this study, is not widespread in consumer behaviour studies. Both could be used to determine potential reuse and refill applications, while also measuring the change in SUPP consumption. An enhancement to future data collection would be to include greater exploration of why people responded with certain attitudes and behaviours towards plastics.

### SUPP disposal in Portsmouth households

SUPP waste was mainly disposed of in general waste or recycling bins, or kept indefinitely for either reuse or storage purposes (RQ1; Fig. 4). Plastic film is not accepted for recycling in Portsmouth, leading to a high rate of disposal in general waste bins. This could be an important focus for investing in alternative innovations or implementing new regulations on film SUPPs, not only in Portsmouth but on a larger scale. This also applies to personal care items: disposable razors, toothbrushes, and cotton buds, for example, are un-reusable, unrecyclable, cannot be rehomed and are hazardous to the environment. Currently, 5% of the top ten most commonly found litter in the UK are personal care items such as cotton buds and sanitary products (Earthwatch Institute 2020). We found that, while not always considered as SUPPs, a vast majority of the personal care items in Portsmouth households ended up in landfill. Personal care items are frequently purchased and replaced and tend to accumulate in households over time. Alternatives to SUPP packaging and plastic-based personal care items provide opportunities for changing consumer behaviour. Clearly labelled items, recycling rules and easily accessible recycling points are essential to engage desired consumer action.

The most recycled SUPP waste were bottles and tubs (including domestic bins, public bins and recycling centres). The confusion caused by lack of clear product labelling and contradictory recycling advice on products and recycling policies are likely to be major factors in recycling compliance and are further reflected in variable consumer behaviour (Rhein and Schmid 2020). Consequently, recycling rates and standards can be difficult to maintain if communication about product labels and local recycling advice are conflicting, especially in the case of cross-contamination in recycling streams. In 2021, 647,000 t of recycling collected in England was rejected, due to contamination or “wish-cycling”, i.e. placing non-recyclable or not collected plastic items in the recycling bin (Callingham 2020; Somerville 2017; Valanidas 2018). We also observed “wish-cycling” amongst Portsmouth respondents, showing uncertainty whether those items are collected and processed by local waste management. This risks cross-contamination during the recycling process and its outputs. Low recycling rates amongst respondents and in Portsmouth overall may be due to the limited recycling collection in Portsmouth and contradictory messaging. Some respondents may feel discouraged to recycle due to the debate about the sustainability of these practices, as recycling is not a sole solution to the plastic problem, and can often complicate, delay or lead to further environmental health problems (Geyer et al. 2016).

A study from Burgess et al. (2021) proposed the systems-wide vision of ‘One bin to rule them all’ in the UK for the optimum recycling of household plastic items with an all-encompassing framework. This involves starting the stream of easily recyclable plastic materials into the system, removing complex mixed materials, and promoting the reuse of polymers and inclusive chemical and mechanical disposal pathways. If this out-engineering of complexity was implemented, it could be a step towards a circular plastic economy, while conserving resources through the reduction and eventual elimination of plastic leakage into the environment. However, ‘One bin to rule them all’ (Burgess et al. 2021) would require an internationally consistent approach, and might still encourage plastic production by promoting convenient recyclability for consumers as the solution, compared to a circular approach. The sorting and processing capacity for mixed waste is problematic in areas such as Portsmouth with limited recycling infrastructure. The information on which plastics are recyclable locally and how to recycle them needs to be clear and uniformly coherent. This should be facilitated through appropriate legislation and policy changes and collaboration with waste management service providers.

### Socio-economic factors and demographics of significance

Previous research has suggested that younger people are more socially, environmentally, and culturally conscious and more readily accept innovative ideas for sustainability (Deliana and Rum 2019; Hume 2010). Unexpectedly, our findings did not support this as older respondents overall showed more effort towards reducing and reusing their SUPPs (RQ2). Age of Portsmouth respondents significantly influenced 10 out of 12 SUPP purchasing and reuse behaviours (RQ2; Table 4) as well as on six out of 10 of the consumer perceptions and behaviours (Table 6). The oldest age group (> 50 years) purchased fewer SUPPs on average and reused bags more often than the two younger age groups, whereas younger respondents reused bottles and tubs more frequently (Table 4).

Younger generations often have higher levels of environmental awareness, exhibit ‘green behaviours’ and are more active than older generations on environmental issues (Deliana and Rum 2019). These green behaviours in consumers have been defined as being more adaptive to environmentally friendly or sustainable product choices. For Portsmouth respondents, this was often the opposite, with a significantly higher proportion of the older age group saying they have made an active effort to reduce their plastic consumption than younger age groups. The older age group was also most likely to consider sustainability and ease of recycling in their product choices, while the younger generation often expressed that “it is too much hassle to recycle”. Another study on plastic packaging found that 39% of younger generations shop in zero-waste stores very often and only 6% do not plan to shop in a zero-waste initiative, compared to over half of the older generation who have never visited a zero-waste store and 29% do not plan to (Holotová et al. 2020). Currently, Portsmouth only has three shops with varying degrees of zero-waste business models, which was reflected in the: (1) low awareness and use of zero-waste shops amongst all respondents and (2) low use of non-plastic food wraps (PAB9, Fig. 5). Growing reuse behaviour and zero-waste culture needs the support of a policy framework that backs the reduction of plastic consumption and more sustainable product design to achieve a more circular economy (Steinhorst and Beyerl 2021). Measuring existing consumption and use behaviours will be necessary to inform the development of effective plastic policies, especially in light of the recent UNEA-5.2 resolution (UNEP 2022b).

Age was followed by gender in significance, with an impact on six of the SUPP flow aspects (RQ2; Table 4). However, gender only impacted respondents' perceptions on their awareness of zero-waste stores (Table 6). Other UK studies have found gender to be the only significant contributor to avoidance of plastic bags and disposable coffee cups (Borg et al. 2020). In addition, we identified five less significant demographics and socio-economic factors: education, living situation, postcode, income and vehicle ownership, each impacting four different SUPP flow aspects, consumer perceptions and behaviours altogether. No significance was found between the education levels of respondents and their plastic product choices and sustainable attitudes and behaviours. However, a recent Dutch study found that, in addition to age, purchase decisions of consumers depended on their sustainable behaviour, knowledge of the circular economy and their perception of the usefulness of plastic (Núñez-Cacho et al. 2020). This was not the case in Portsmouth. Moreover, the choices of individuals have been found to be affected by personal knowledge and community behaviour (i.e. actions that society takes at different levels such as government policies and changes in the business models) (Cavaliere et al. 2020). Núñez-Cacho et al. (2020) found consumers with greater awareness of the impact of plastic express more concern and more effort in avoiding SUPPs. Higher education levels have also been predicted to correspond with higher levels of environmental awareness and ‘green behaviour’ (Deliana and Rum 2019; Zsóka et al. 2013).

### Barriers to sustainable consumer behaviour

Transport and location accessibility were major barriers to the sustainable purchasing and recycling habits of Portsmouth respondents. Portsmouth is a low-emission transport zone and has heavily restricted parking to reduce vehicle use in the city. These restrictions could result in fewer residents owning vehicles than other city locations (ONS 2012), but, currently, the majority (70%) of Portsmouth respondents own vehicles. If a respondent lives within PO4–PO5 postcodes, they have greater ease of accessibility to the three zero-waste stores in Portsmouth. However, these aforementioned postcodes would require transport to recycling centres or council locations from which they are then sent to landfill sites, the MRF or an Energy Recovery Facility located further outside of the city centre. Larger stores with integrated recycling facilities or collection points for drink cartons, plastic bags, film, glass bottles, and tubs are sparsely located around the city. Although postcodes were not found to have a significant impact on recycling, owning one or more vehicles may remove many of the accessibility and transportation barriers. In turn, those barriers still exist for households without a vehicle and may prevent them from recycling more of their plastic waste. These factors might influence the attitudes and behaviours of individuals, particularly those experiencing the inconvenience or frustration with the capacity and accessibility of nearest recycling facilities.

The barriers we identified to reduce plastic consumption showed that respondents felt that they are not completely responsible for their purchase behaviours. The respondents expressed that they would like to see more environmentally friendly decisions from designers, manufacturers, and retailers, which would facilitate the reduction of plastic consumption by consumers. As plastic remains as the most prevalent packaging and product material, it is difficult for consumers to avoid it. Consumers might also be reluctant to take the sole responsibility for reducing plastic consumption as convenience is often governed by the prices and availability of sustainable alternatives. A study on the attitudes and behaviours of businesses towards plastic consumption identified cost as the biggest challenge for 86% of businesses to reduce plastics and use sustainable alternatives (Varkey et al. 2021). Moreover, Carrete et al. (2012) identified three main themes causing uncertainty in consumers for adopting green behaviours: (1) consumer confusion, (2) trust and credibility, and (3) compatibility with individuals’ values. Future surveys should include these themes for more comprehensive insights.

### PABs and WDAs

We found four key PABs expressed by Portsmouth respondents (RQ3): the refusal of plastic shopping bags, refusal of plastic straws, refusal of plastic take-away cups and avoidance of personal care products containing plastic microbeads (Fig. 5). Unexpectedly, recycling their plastic waste at home or in public bins was only expressed by approximately 8% of the respondents in this section of the survey. Based on our study, we could classify behaviours as low-effort or high-effort behaviours. In low-effort behaviours, decisions are easily and quickly made during shopping or dining that do not require deep reflection beforehand (Jacobsen et al. 2022), including our four key PABs. These decisions are premade by the businesses through their provision (or lack) of alternatives. Safety concerns such as preventing COVID-19 spread can also result in preferring single-use plastics to reuse or non-plastic options even after reduced risk of transmission (Winton et al. 2022). Conversely, the high-effort behaviours need the backing of external infrastructure and policies (e.g. recycling), require space (i.e. buying in bulk) or can be more expensive and inaccessible to some (i.e. choosing plastic alternatives or zero-waste approaches) (Löhr et al. 2017; Sandhu et al. 2021; Tadesse et al. 2008). Our findings suggest that, when avoiding plastic, consumers are more likely to exhibit low-effort behaviours than high-effort behaviours. This may be due to busy lifestyles, inaccessible infrastructure for zero-waste shopping and recycling, or not being able to prioritise SUPP avoidance for socio-economic reasons (e.g. income, number of dependents in a household). This is a key aspect that could be investigated further in future research.

In regard to WDAs, in general, Portsmouth respondents showed willingness to recycle as much as they can and possessed a strong sense of responsibility around recycling (RQ3; Fig. 6). They also admitted to being confused about recycling advice and concerned about the state of littering in Portsmouth. Overall, respondents agreed with most of the WDA statements, such as the importance of recycling, littering being a serious problem and that they should do more to recycle, which suggests that they have a strong perception of responsible disposal behaviours (Fig. 6). A UK case study conducted in Exeter examined similar WDBs and attitudes finding ~ 68% of respondents buy products with as little packaging as possible (Barr 2007) compared to 5% of Portsmouth respondents who said they avoid pre-packed plastic food products. Approximately 56% of Exeter respondents said that they use their own bag and avoid buying shopping bags, compared to 19% of Portsmouth respondents that refuse plastic shopping bags. Recycling habits also varied significantly between the two cities with 70% of Exeter respondents recycling plastic bottles and only 8% of Portsmouth residents recycling their plastic waste in domestic, public and workplace recycling bins. Another coastal city study found beach goers with a higher environmental awareness had a more negative perception towards SUPPs and a stronger willingness to reduce their plastic consumption (Van Rensburg et al. 2020). These individuals also showed greater support for initiatives to combat plastic waste such as container deposit systems and plastic bag bans. Pay-as-you-throw schemes could be another option to encourage consumers to reduce and sort their plastic waste.

Awareness is an important part of SUPP consumer behaviour. Both retailers and customers are often aware of environmental issues associated with plastic, while a lack of awareness can take away momentum from behaviour change. 75% of Portsmouth residents were unaware of the local climate action group, 62% were unaware of the main zero-waste store and 21% did not believe that any of their plastic waste ends up in the ocean. If the consumption of SUPPs is to be reduced, raising awareness of the impacts of plastic should be a part of future shifts towards circularity, especially in densely populated coastal cities. The road should be paved by national and local governments to facilitate the transition to a circular economy amongst consumers and businesses. However, consumers still appreciate the purpose and convenience of plastic products and continue to routinely use them as they feel that there is a lack of feasible alternatives on offer from the producers and retailers to make these changes (Heidbreder et al. 2019).

### Study limitations and future research recommendations

Establishing each flow of different plastic items through a household accurately from purchase to disposal at home is difficult. In hindsight, the questionnaire was not structured in a way that would have allowed for extensive statistical analysis or modelling the flow of SUPP items through households. To enable this in future studies, a coherent survey structure with similar questions for purchase, reuse and disposal is recommended. Questions about consumers' awareness of important concepts such as circular economy would be useful to infer their influence on consumer behaviour as other studies have shown (Núñez-Cacho et al. 2020; Cavaliere et al. 2020). Specificity was another issue. Questions should be clear and specific about the plastic focus. Switching the focus from SUPPs to fast-moving consumer goods to incorporate other commonly used plastics such as personal care items and delivery packaging could also add value to future research. Another possible avenue is to group all SUPP items together to analyse consumer behaviour towards SUPPs in a more general but comprehensive way. Other unpreferable end-of-life destinations for plastic waste could be introduced for any unaccounted-for plastic in the flow such as irresponsible littering or dumping to monitor plastic pollution rates and to pinpoint the extent of plastic waste mismanagement. This study was conducted during the COVID-19 pandemic; therefore, purchase rates of SUPPs may have been higher than usual. However, there are no peer-reviewed studies or official data available on SUPP purchases in Portsmouth from before the pandemic. Therefore, it was not possible to compare Portsmouth-specific trends in a pandemic-context.

## Conclusions

The findings from this study have enabled an enhanced understanding of SUPP purchase, use and disposal trends in Portsmouth. The results demonstrate the value in researching which actions generate the most beneficial behaviour change amongst consumers. However, we recognise that consumers are not the target stakeholder carrying the responsibility for systemic change. While this study was purely exploratory in nature and provides an example of how the role of SUPPs in households could be researched, it has become clear that the way consumer behaviour around plastics has been studied to date is in need of increased standardisation through:

(i) uniform measurement units for plastic items to enable realistic comparisons,
(ii) robust but comprehensive questionnaires for analysis and modelling purposes,
(iii) survey databases from research across the globe to model and track how plastic products flow through households, which would also act as a valuable resource for the research community.

These standardisations could significantly improve the mapping of both behaviour and policy change options. While also informing governments and other stakeholders whether or not their current products, practices and policies are, in fact, viable or in need of redesign or amendments. As plastics are largely universal both in the sense of utilisation and pollution, the solutions to mitigate against the negative impacts of plastics must also be widely applied.

## Supplementary Information

Below is the link to the electronic supplementary material.

Supplementary file1 (PDF 369 KB)

Supplementary file2 (PDF 563 KB)

## Data Availability

Data from this study is available on request from the corresponding author.

## Abbreviations

MRF: Materials’ recovery facility
PAB: Plastic avoidance behaviour
SUPP: Single-use plastic product
WDA: Waste disposal attitude
WDB: Waste disposal behaviour
