# Supplementary material for: From shops to bins: a case study of consumer attitudes and behaviours towards plastics in a UK coastal city
Source: Sustain Sci. 2023 Jan 30;18(3):1379–95. doi: 10.1007/s11625-022-01261-5 (PMC9884600; doi:10.1007/s11625-022-01261-5)
Supplement: Supplementary file 1 — Supplementary file1 (PDF 369 KB) [file 11625_2022_1261_MOESM1_ESM.pdf]

University of Portsmouth – Revolution Plastics

Question Set
September 2019

Red Brick Research
1 Mount Ephraim Road
Royal Tunbridge Wells
KENT
TN1 1ET

INTRODUCTION

Welcome!

Thank you for taking the time to complete this survey on behalf of your local University, the University of Portsmouth.

The University is committed to playing a positive role in the local community, and on the national and international stage. This survey is designed to explore how Portsmouth residents feel about national and international issues, and conversations that are taking place around the world.

The survey should take around 10-15 minutes to complete. Your responses are anonymous, and there are no right or wrong answers; please do answer openly and honestly.

Please click through to begin the survey.

This survey is being conducted by Red Brick Research on behalf of the University of Portsmouth to help them understand how the local community feel about local and global issues. Your responses to the survey will be processed securely and confidentially by us in accordance with the General Data Protection Regulation, Data Protection Act and the Market Research Society Code of Conduct at all times. Any personal data you provide us will be destroyed as soon as possible (usually after any incentives have been administered) and will never be held for more than 3 months without explicit permission from you. By voluntarily participating in this survey you consent to any information you submit being used for analysis and research purposes only. We will never mislead you about the intended use of your personal data and will never pass them onto a third party. During this survey, we may ask for sensitive data in the form of your gender. You are entitled to withdraw from (end) the survey at any time and request that any or all of your personal data be destroyed. You are entitled to request the right to access your data at any time after submitting your responses. To comply with financial regulations, we may retain basic information about your identity if we award a prize or make an incentive payment to you. This payment record cannot be linked back to your survey responses. We use cookies and other similar technologies sparingly to reduce fraudulent and mischievous survey entries, and to provide an enhanced user experience.

SECTION: DEMOGRAPHICS

ASK ALL: SINGLE SELECT

DD00. Are you currently living in the City of Portsmouth?

Yes	**1**	
No	**2**	**[Screen out]**

ASK ALL: SINGLE SELECT

DD01. How long have you lived in Portsmouth?

Less than a year	**1**	
1-5 years	**2**	
6-10 years	**3**	
10-20 years	**4**	
More than 20 years	**5**	
I am living in Portsmouth temporarily (e.g. as a student)	**98**	**[Screen out]**

ASK ALL: DROP-DOWN LIST

DD02. In which part of Portsmouth do you currently live?

Please select your district postcode from the drop-down list below.

Dropdown list of postcodes PO1 – PO41

ASK ALL: SINGLE SELECT

DD03. Which of the following best describes your living situation?

I live alone	**1**
I live with my parents / family	**2**
I live in a house- / flat-share	**3**
I live with my partner / children	**4**
Other (please specify)	**99**

ASK IF DD03 ≠ 1 (I LIVE ALONE) – FREE TEXT

DD04. Including yourself, how many adults / children live in your household?

Please type in below.

Adults (18+)	[FREE TEXT]
Children	[FREE TEXT]

ASK ALL: SINGLE SELECT

DD05. What type of property do you live in?

Flat / Apartment	**1**
Detached House	**2**
Semi-Detached House	**3**
Terraced House	**4**
End-of-Terrace House	**5**
Cottage	**6**
Bungalow	**7**
Other (please specify)	**99**

ASK ALL: SINGLE SELECT

DD06. What is the size your residence?

Studio	**1**
1 bedroom	**2**
2 bedrooms	**3**

3 bedrooms	**4**
4 bedrooms	**5**
5 or more bedrooms	**6**

ASK ALL: SINGLE SELECT

DD07. How many bathrooms does your residence have?

1 bathroom	**1**
2 bathrooms	**2**
3 bathrooms	**3**
4 bathrooms	**4**
5 or more bathrooms	**5**

ASK ALL: SINGLE SELECT

DD08. Is your current residence rented or owned?

Owned	**1**
Rented	**2**
Other (please specify)	**98**
I don't know	**99**

ASK ALL: MULTI SELECT

DD09. How would you describe your involvement when it comes to your household's purchasing decisions?

I am solely or jointly responsible for **all** the purchasing decisions in my household	**1**
I make **most of** the purchasing decisions in my household	**2**
I make **some of** the purchasing decisions in my household	**3**
I make **none of** the purchasing decisions in my household	**4**

SECTION: PLASTICS CONSUMPTION

To begin with, we'd like to learn a little about your regular purchasing habits.

ASK ALL: MATRIX [RANDOMISE ROWS]

C10. In an average week, approximately how many of each of the following items do you / your household purchase?

Plastic bottles (e.g. drinks, shampoo, cleaning products, etc.)	**1**
Items wrapped in plastic film (e.g. crisps, salad, frozen foods, multi-pack tins / cans etc.)	**2**
Plastic tubs (e.g. yoghurt pots, punnets of fruit / veg, meat / fish packed in plastic containers etc.)	**3**
Plastic shopping bags (single-use)	**4**

None	**1**
1 – 2	**2**
3 – 5	**3**
6 – 10	**4**

11 – 15	**5**
16+	**6**
I don't know	**99**

ASK ALL – MULTI-SELECT [RANDOMISE]

C20. What are the most important considerations for you in your everyday supermarket purchases?

Please tick all that apply.

Price	**1**
Convenience	**2**
Deals / Discounts	**3**
Brand	**4**
Quality	**5**
Sustainability	**6**
Ethics	**7**
Use-by-dates / Longevity	**8**
Value for money	**9**
Ease of recycling packaging	**10**
Other (please specify)	**99**

ASK ALL – SINGLE-SELECT

C30. And which of these is your most important consideration?

[INSERT OPTIONS CHOSEN ABOVE]	**1**

ASK ALL – SINGLE-SELECT

C40. Which of the following best describes your attitude towards single-use plastics in your everyday supermarket purchases?

I will go out of my way to avoid single-use plastics in my everyday purchases	**1**
If the option is readily available, I will choose to avoid single-use plastics	**2**
I will avoid single-use plastics only where there is no cost to me in doing so	**3**
Avoiding single-use plastics is not a priority for me	**4**

ASK ALL – SINGLE-SELECT

C50. Food is often packaged in plastic to prolong its shelf life. Imagine you have the option to buy a product packaged in plastic with a longer shelf life, or the same product with non-plastic packaging and a shorter shelf life.

How often would you choose to buy the product with non-plastic packaging and a shorter shelf life?

Always	1
As often as I can	2
If there's another reason to choose it (e.g. it is the cheapest / on offer / my preferred brand)	3
Rarely / never	4

ASK ALL: SINGLE-SELECT

C60. Are you aware of Portsmouth's first plastic-free supermarket 'The Package Free Larder' in Southsea?

Yes, I shop there regularly	**1**
Yes, I shop there occasionally	**2**
Yes, I've visited at least once	**3**
Yes, but I've never visited	**4**
No, but I'd be keen to shop there in the future	**5**
No, and I'm not likely to shop there in the future	**6**

ASK ALL – MULTI-SELECT [RANDOMISE]

C70. Which, if any, of the following have you done in the past?

Please select all that apply.

Refuse plastic shopping bags	1
Refuse plastic straws	2
Refuse take-away cups (e.g. by taking your own or drinking in the café)	3
Avoid plastic microbeads in personal care products	4
Avoid food products pre-packed in plastic (e.g. fruit / veg)	5
Avoid buying bottled drinks (e.g. by using a reusable bottle)	6
Recycle plastic in domestic recycling bins (at home)	7
Recycle plastic in public / workplace recycling bins	8
Use non-plastic food-wrap	9
Choose alternative versions of products otherwise packed in plastic (e.g. solid shampoo, toothpaste tablets, deodorant bars etc.)	10
Used reusable shopping bags (e.g. fabric bags, bags for life etc.)	11
Bought in bulk to reduce unnecessary plastic packaging	12
None of the above	99

ASK IF C70 ≠ 99 (NONE OF THE ABOVE) – MATRIX

C80. How often do you do each of these things?

[Insert answers selected at C70]	1

Always	1
In most cases	2
Sometimes	3
Rarely	4

ASK ALL – SINGLE-SELECT

C80. How often do you buy new clothes for yourself or members of your household?

Every week	1
Most weeks	2
Most months	3
A few times a year	4
Once a year	5

Less often	6

ASK ALL – MULTI-SELECT [RANDOMISE]

C90. Which, if any, of the following retailers have you bought clothes from in the past?

Please select all that apply.

Primark	1
ASOS	2
Supermarket Fashion labels	3
Boden	4
Nomads	5
Bibico	6
Debenhams / Next	7
M&S	8
Rapanui	9
Patagonia	10
None of the above	99

ASK ALL – MULTI-SELECT

C100. Which retailers do you buy clothes from most regularly?

[Insert retailers selected above]	**1**
Other (please specify)	**2**

ASK ALL – MULTI-SELECT [RANDOMISE]

C110. What are the most important considerations for you in your clothing purchases?

Please tick all that apply.

Price	**1**
Convenience	**2**
Deals / Discounts	**3**
Brand	**4**
Quality	**5**
Sustainability	**6**
Ethics	**7**
Longevity	**8**
Style	**9**
Value for money	**10**
Fabric composition	**11**
Other (please specify)	**99**

ASK ALL – SINGLE-SELECT

C120. And which of these is your most important consideration?

[INSERT OPTIONS CHOSEN ABOVE]	**1**

Moving on, we'd like to find out more about the flow of different types of products through households in Portsmouth. This will help us to build up a picture of how we all use and store plastics across the city. If you can give us your best approximations across the following few questions, we'd really appreciate it – don't worry about being too precise!

ASK ALL: MATRIX [RANDOMISE]

C130. Approximately how many of the following items (containing or made entirely from plastic) are you / your household currently in possession of?

**Plastic toys**	**2**
**CDs / DVDs / Records / Tapes**	**3**
**Kitchen utensils** (e.g., pots, pans, bowls, ladles, spatulas etc.)	**4**
**Gardening items** (e.g., pots, tools, etc.)	**5**
**Personal care items** (e.g., toothbrushes, hairbrushes, razors, etc.)	**6**
**Reusable plastic containers** (e.g., water canisters, lunchboxes, fridge storage, etc.)	**7**

None	**1**
1 – 5 items	**2**
6 – 10 items	**3**
11 – 25 items	**4**
26 – 50 items	**5**
51 – 100 items	**6**
101 – 200 items	**7**
201 – 500 items	**8**
501+ items	**9**
I don’t know	**99**

ASK ALL: MATRIX [RANDOMISE]

C140. Approximately how many of the following items are you / your household currently in possession of?

**Motor Vehicles** (e.g., motorbike, car, van, etc.)	**1**
**Bicycles & Scooters**	**2**
**Small Appliances** (e.g. iron, blender, toaster, kettle, microwave etc.)	**3**
**Large Appliances** (e.g. fridge, freezer, oven, washing machine, tumble dryer etc.)	**4**
**Computing equipment** (e.g. computers, laptops, monitors, tablets and printers etc.)	**5**
**TVs & Audio systems**	**6**
**Mobile Phones**	**7**
**Gym Equipment** (e.g. large items such as treadmills, stationary bikes etc.)	**8**

None	**1**
1	**2**
2 – 3	**3**
4 - 5	**4**
6 - 10	**5**
11 - 15	**6**
16+	**7**
I don’t know	**99**

ASK ALL: MATRIX [RANDOMISE]

C150. Approximately how many of the following items of furniture are you / your household currently in possession of?

**Beds**	**1**
**Sofas**	**2**
**Armchairs**	**3**
**Plastic tables / desks**	**4**
**Plastic chairs**	**5**
**Plastic wardrobes / chests of drawers**	**6**
**Plastic storage / shelving units**	**7**

None	**1**
1	**2**
2 – 3	**3**
4 - 5	**4**
6 - 10	**5**
11 - 15	**6**
16+	**7**
I don't know	**99**

ASK ALL: MATRIX [RANDOMISE ROWS]

C160. To what extent, if at all, are you likely to consider the following when purchasing items for long-term use such as vehicles, appliances and furniture?

Ease of accessing replacement parts in the future	**1**
Use of sustainable materials in the product	**2**
Supplier's overall reputation for sustainable and ethical practices	**3**
Ease of recycling the product when I want to replace / dispose of it	**4**

Primary consideration	**1**
Important secondary factor	**2**
Potential differentiating factor	**3**
Does not factor in my decisions	**4**

ASK ALL – MULTI-SELECT [RANDOMISE]

C170. What, if anything, puts you off or prevents you from reducing your plastic use?

Please select all that apply.

It is difficult to find alternatives to single-use plastic products / materials	1
Alternative products / materials are too expensive	2
The products I want are not available without single-use plastics	3
Alternative products / materials do not work as well	4
I often forget to carry reusable products when out and about	5
I do not think it is important to reduce my plastic use	6
Other (please specify)	98
None of the above	99

ASK IF C170 ≠ 99 (NONE OF THE ABOVE) – SINGLE-SELECT

C180. Which of these is the biggest barrier to reducing your plastic use?

[Insert answers selected at P50a]	1

SECTION: PLASTICS USAGE

ASK ALL: MATRIX [RANDOMISE ROWS]

U10. How often, if at all, do you / your household typically re-use the following single-use plastics before disposing of them?

Plastic bottles	**1**
Plastic film	**2**
Plastic tubs	**3**
Plastic shopping bags (single-use)	**4**

Never re-use	**1**
Once	**2**
2 – 4 times	**3**
5 – 10 times	**4**
More often	**5**

ASK ALL: MATRIX [RANDOMISE ROWS]

U20. For approximately how long do you tend to keep each of the following types of items before disposing of / replacing them?

**Clothes**	**1**
**Toys**	**2**
**CDs / DVDs / Records / Tapes**	**3**
**Kitchen utensils** (e.g., pots, pans, bowls, ladles, spatulas, cutlery, etc.)	**4**
**Gardening items** (e.g., pots, tools, etc.)	**5**
**Personal care items** (e.g., toothbrushes, hairbrushes, razors, etc.)	**6**
**Reusable containers** (e.g., water canisters, lunchboxes, fridge storage, etc.)	**7**
**Motor Vehicles** (e.g., motorbike, car, van, etc.)	**8**
**Bicycles & Scooters**	**9**
**Furniture** (e.g., beds, sofas, armchairs, tables, desks, wardrobes, drawers, shelving, etc.)	**10**
**Small Appliances** (e.g. iron, blender, toaster, kettle, microwave etc.)	**11**
**Large Appliances** (e.g. fridge, freezer, oven, washing machine, tumble dryer etc.)	**12**
**Computing equipment** (e.g. computers, laptops, monitors, tablets and printers etc.)	**13**
**TVs & Audio systems**	**14**
**Mobile Phones**	**15**
**Gym Equipment** (e.g. large items such as treadmills, stationary bikes etc.)	**16**

Forever / indefinitely	**1**
11+ years	**2**
5 – 10 years	**3**
2 – 4 years	**4**
1 year	**5**
A few months	**6**
Less time	**7**
I don't know	**99**

ASK ALL – MULTI-SELECT [RANDOMISE]

U30. Which, if any, of the following have you done in the past?
Please select all that apply.

Repair your own clothes or shoes	**1**
Pay someone else to repair your clothes or shoes	**2**
Repurposed fabric from old clothes (e.g. to make new clothes / other items)	**3**
Bought replacement parts rather than replacing an item entirely	**4**
'Upcycled' old / second-hand furniture rather than buying something new	**5**
Thrown away / replaced a broken or malfunctioning item without attempting to get it repaired	**6**
None of the above	**99**

ASK IF U30 ≠ 99 (NONE OF THE ABOVE) – MATRIX
U40. How often do you do each of these things?

[Insert answers selected at U30]	**1**

Always	**1**
In most cases	**2**
Sometimes	**3**
Rarely	**4**

SECTION: WASTE DISPOSAL

ASK ALL: SINGLE SELECT DROPDOWNS [RANDOMISE]
W10. How do you / your household typically dispose of the following single-use plastics?

Plastic bottles	**1**
Plastic film	**2**
Plastic tubs	**3**
Plastic shopping bags (single-use)	**4**
Plastic delivery packaging (e.g. from online shopping orders)	**5**

General waste bin	**1**
Recycling bin	**2**
Take it to a recycling centre	**3**
Specialist waste collection services	**4**
Take it to a landfill site	**5**
Use a deposit return scheme	**6**
Store indefinitely in the home / storage facility	**7**
Other (please specify)	**98**
I don't know	**99**

ASK ALL: SINGLE SELECT DROPDOWNS [RANDOMISE] – [INCLUDE ALL ITEMS RESPONDENT HAS AT LEAST ONE OF IN PREVIOUS SECTION]
W20. How do you / your household typically dispose of the following items?

**Clothes**	**1**

**Toys**	**2**
**CDs / DVDs / Records / Tapes**	**3**
**Kitchen utensils** (e.g., pots, pans, bowls, ladles, spatulas, cutlery, etc.)	**4**
**Gardening items** (e.g., pots, tools, etc.)	**5**
**Personal care items** (e.g., toothbrushes, hairbrushes, razors, etc.)	**6**
**Reusable plastic containers** (e.g., water canisters, lunchboxes, fridge storage, etc.)	**7**
**Motor Vehicles** (e.g., motorbike, car, van, etc.)	**1**
**Bicycles & Scooters**	**2**
**Furniture** (e.g., beds, sofas, armchairs, tables, desks, wardrobes, drawers, shelving, etc.)	**3**
**Small Appliances** (e.g. iron, blender, toaster, kettle, microwave etc.)	**4**
**Large Appliances** (e.g. fridge, freezer, oven, washing machine, tumble dryer etc.)	**5**
**Computing equipment** (e.g. computers, laptops, monitors, tablets and printers etc.)	**6**
**TVs & Audio systems**	**7**
**Mobile Phones**	**8**
**Gym Equipment** (e.g. large items such as treadmills, stationary bikes etc.)	**9**

Put in general waste bins	**1**
Put in recycling bins	**2**
Take to a recycling centre	**3**
Make use of specialist waste collection services	**4**
Take to a landfill site	**5**
Use a deposit return scheme	**6**
Take to a scrap yard	**7**
Give to charity	**8**
Sell	**9**
Give to someone I know	**10**
Store indefinitely in the home / storage facility	**11**
Other (please specify)	**98**
I don't know	**99**

ASK ALL: SINGLE SELECT

W30. Where do you sit on the scale below in terms of your attitude to waste disposal?

1 - I don't really think about what happens to my waste once it is out of my hands	**1**
2	**2**
3	**3**
4	**4**
5	**5**
6	**6**
7- I am very concerned about where my waste ends up and what impact it has on the environment	**7**

ASK ALL: SINGLE SELECT

W40. How often do you believe the plastic you throw away ends up in the sea?

Always	**1**
Most of the time	**2**
Sometimes	**3**

Rarely	**4**
Never	**5**

ASK ALL: SINGLE SELECT

W50. To what extent do you agree or disagree with the following statement? "Littering is a serious problem that needs addressing in Portsmouth."

Strongly agree	**1**
Agree	**2**
Neither agree nor disagree	**3**
Disagree	**4**
Strongly disagree	**5**

ASK ALL – MATRIX [RANDOMISE]

W60. To what extent do you agree or disagree with the following statements about recycling?

It is difficult to identify which items I can / can't recycle	1
I believe it is important to recycle	2
I don't always recycle everything that I can	3
I actively choose to buy recycled products where I can	4
The council should do more to help people recycle in my area	5
I feel I should recycle more	6
Employers have a duty to provide recycling facilities in the workplace	7
I am willing to pay more for items made from recycled products	8
I don't always know how / where to recycle certain items	9
It is important to me that manufacturers use more recycled and sustainable materials	10

Strongly agree	1
Agree	2
Neither agree nor disagree	3
Disagree	4
Strongly disagree	5

ASK ALL – MULTI-SELECT [RANDOMISE]

W70. What, if anything, puts you off or prevents you from recycling more of your waste?
Please select all that apply.

It's too much hassle to recycle	1
The council doesn't collect all the things that could be recycled	2
There are not enough recycling facilities available to me locally	3
It's too difficult for me to transport my recycling to local facilities	4
Local establishments do not do enough to support recycling outside of the home	5
It is difficult to know what items can / can't be recycled	6
Other members of my household refuse to recycle	7
I often forget to recycle	8
I believe a lot of my 'recycling' ends up in landfill	9

I do not think recycling is important	10
Other (please specify)	98
Nothing - I already recycle everything that I can	99

ASK IF W70 ≠ 99 (NONE OF THE ABOVE) – MULTI-SELECT

W80. Which of these is the biggest barrier to recycling more of your waste?

[Insert answers selected at R50a]	1

ASK ALL – MULTI-SEECT - RANDOMISE

W90. What, if anything, might encourage you to recycle more in the future?
Please select all that apply.

Better availability of recyclable products	1
If my council collected more types of recycling	2
Improvements to local recycling facilities	3
More information about what I can / can't recycle	4
More information about the recycling facilities available in my local area	5
More incentives to recycle, e.g. cash-back schemes	6
If I could see that it made a real difference	7
Improvements to recycling facilities at my place of work	8
Improvements to recycling facilities at local events	9
Improvements to recycling facilities in local shopping areas	10
Other (please specify)	98
Realistically, I can't see myself recycling more in the future	99

SECTION: ACTION

ASK ALL – SINGLE-SELECT

A10. Were you aware that Portsmouth has a climate action group?

Yes	1
No	2

ASK ALL – SINGLE-SELECT

A20. Are you personally involved in any groups or organised efforts to address climate change and sustainable living?

Yes	**1**
No	**2**

ASK ALL – SINGLE-SELECT

A30. Do you believe the actions of individuals such as yourself can reduce the effects of climate change / help to protect the planet?

Yes, definitely	1
Yes, probably	2

No, probably not	3
No, definitely not	4
I don't know	99

ASK ALL – MULTI-SELECT [RANDOMISE]

A40. Thinking about the future, which of the following statements are true for you?
Please select all that apply.

I would like to do more to tackle issues around climate change	1
I would like to do more to tackle issues around sustainable living	
I support the University of Portsmouth in doing more to tackle issues around climate change and sustainable living	2
I would like to learn more about sustainability and climate action	3
I support the ambition of Portsmouth becoming a sustainable city	4
I would like Portsmouth to be known internationally as being a sustainable city	5
I would be proud if my city led the way in sustainability and climate action	6
I have made an effort to reduce my plastic consumption in the last few years	7
None of the above	99

SECTION: DEMOGRAPHICS

Thank you for taking the time to complete our survey. The last few questions are about you and will help us to better understand your responses.

ASK ALL – SINGLE SELECT

DD10. How would you describe yourself?

Male	**1**
Female	**2**
In another way	**3**

ASK ALL – SINGLE SELECT

DD20. What is your age?

Under 21	**1**
21 - 25	**2**
26 - 30	**3**
31 - 40	**4**
41- 50	**5**
51 - 60	**6**
61 - 70	**7**
71 - 80	**8**
81+	**9**

ASK ALL – SINGLE SELECT

DD30. What is your annual household income (before tax)?

Less than £12,000	**1**

£12,000 - £14,999	**2**
£15,000 - £19,999	**3**
£20,000 - £24,999	**4**
£25,000 - £34,999	**5**
£35,000 - £49,999	**6**
£50,000 - £74,999	**7**
£75,000 - £99,999	**8**
£100,000 - £149,000	**9**
£150,000 or more	**10**
Prefer not to say	**99**

ASK ALL – SINGLE SELECT

DD40. What is the highest degree or level of school you have completed?

Please tick one answer only.

None completed	**1**
Primary School	**2**
Secondary School	**3**
High School / Tertiary / Tech. College	**4**
University / Higher Education	**5**
Postgraduate Education	**6**

ASK ALL – SINGLE SELECT

DD50. Which of the following best describes your current employment status?

Working full time in paid employment	**1**
Working part time in paid employment	**2**
Self-employed (either part time or full time)	**3**
Working in a voluntary capacity (unpaid)	**4**
Student	**5**
Not currently working – looking for work	**6**
Not currently working – not looking for work	**7**
Retired	**8**
Homemaker	**9**
Other (Please specify)	**99**

ASK ALL – SINGLE SELECT

DD60. Choose one option that best describes your ethnic group or background:

**White**	
English / Welsh / Scottish / Northern Irish / British	**1**
Irish	**2**
Gypsy or Irish Traveler	**3**
Any other White background	**4**
**Mixed/ Multiple ethnic groups**	
White and Black Caribbean	**5**
White and Black African	**6**
White and Asian	**7**

Any other Mixed / Multiple ethnic background	**8**
**Asian / Asian British**	
Indian	**9**
Pakistani	**10**
Bangladeshi	**11**
Chinese	**12**
Any other Asian background	**13**
**Black / African / Caribbean / Black British**	
African	**14**
Caribbean	**15**
Any other Black / African / Caribbean background	**16**
**Other ethnic group**	
Arab	**17**
Any other ethnic group	**18**
Prefer not to say	**97**

ASK ALL – MULTI SELECT

DD70. Do you have any dependents? (Dependents may include, for example, children or elderly parents who are dependent on your income or care).

None	**1**
Child(ren)	**2**
Spouse / partner	**3**
Parent(s)	**4**
Other	**98**
Prefer not to say	**97**

ASK IF D60 = 2 (CHILDREN) – MULTI SELECT

DD80. How old are your children?

Please select all that apply.

0-3	**1**
4-10	**2**
11-17	**3**
18+	**4**
Prefer not to say	**99**

ASK ALL – MULTI SELECT

DD90. Do you have any grandchildren?

Yes	**1**
No	**2**
Prefer not to say	**3**
