# Supplementary material for: From shops to bins: a case study of consumer attitudes and behaviours towards plastics in a UK coastal city
Source: Sustain Sci. 2023 Jan 30;18(3):1379–95. doi: 10.1007/s11625-022-01261-5 (PMC9884600; doi:10.1007/s11625-022-01261-5)
Supplement: Supplementary file 2 — Supplementary file2 (PDF 563 KB) [file 11625_2022_1261_MOESM2_ESM.pdf]

SI2.

PIP - Code and Results

Author: (*redacted)*

19/04/2022

Contents

Used R packages **2**

Data summary **3**

Variable labeling 5

Label check 6

Statistical analysis **8**

Normality and chosen tests 8

Crosstab compilations **8**

'Age' factor: 8

'Education' factor: 15

'Gender' factor: 24

'Income' factor: 31

'Vehicle ownership' factor: 44

'Living situation' factor: 51

'Postcode' factor: 62

Pearson's Chi-squared tests for independence **71**

Age 71

Education 76

Gender 76

Income 76

Vehicle ownership 77

Living situation 82

Postcode 82

Pearson's Chi-square coefficients, i.e. Pearson's chi-square tests with Monte Carlo **84**
Age . . . 84
Education . . . 102
Gender . . . 131
Income . . . 153
Vehicle ownership . . . 193
Living situation . . . 210
Postcode . . . 243

Used R packages

```
library("rmarkdown")
library("knitr")
library("tinytex")
library("tidyverse")
library("broom")
library("fs")
library("usethis")
library("readxl")
library("car")
library("dunn.test")
library("lattice")
library("dplyr")
library("MASS")
```

Data summary

```
index1 <- 1:ncol(data)
data[ , index1] <- lapply(data[ , index1], as.factor)
str(data)
```

```
## tibble [400 x 30] (S3: tbl_df/tbl/data.frame)
##  $ Response ID          : Factor w/ 400 levels "65","66","79",..: 1 2 3 4 5 6 7 8 9 10 ...
##  $ gender               : Factor w/ 3 levels "1","2","3": 2 1 2 1 1 1 2 1 1 2 ...
##  $ age                  : Factor w/ 3 levels "1","2","3": 3 3 3 3 3 2 2 3 3 2 ...
##  $ living_situation     : Factor w/ 5 levels "1","2","3","4",..: 1 4 4 4 1 1 4 4 1 4 ...
##  $ annual_income        : Factor w/ 11 levels "1","2","3","4",..: 1 6 5 6 11 2 3 11 11 5 ...
##  $ education_level      : Factor w/ 5 levels "2","3","4","5",..: 3 4 3 3 2 3 4 5 4 4 ...
##  $ vehicle_ownership    : Factor w/ 2 levels "1","2": 2 2 2 2 1 1 1 2 2 2 ...
##  $ postcode             : Factor w/ 6 levels "1","2","3","4",..: 2 2 1 6 1 1 1 4 6 4 ...
##  $ bottles_avgweek      : Factor w/ 7 levels "1","2","3","4",..: 3 2 2 3 2 2 5 4 3 7 ...
##  $ film_avgweek         : Factor w/ 7 levels "1","2","3","4",..: 2 2 4 3 4 3 6 7 3 7 ...
##  $ tubs_avgweek         : Factor w/ 7 levels "1","2","3","4",..: 1 6 4 3 3 2 6 4 2 7 ...
##  $ bags_avgweek         : Factor w/ 7 levels "1","2","3","4",..: 1 1 1 1 1 1 1 1 1 2 ...
##  $ bottles_reuses       : Factor w/ 5 levels "1","2","3","4",..: 1 3 4 1 2 5 5 1 3 1 ...
##  $ film_reuses          : Factor w/ 5 levels "1","2","3","4",..: 1 1 1 3 1 3 1 1 1 1 ...
##  $ tubs_reuses          : Factor w/ 5 levels "1","2","3","4",..: 5 3 3 2 5 5 5 5 1 1 ...
##  $ bags_reuses          : Factor w/ 5 levels "1","2","3","4",..: 5 4 4 3 1 4 5 5 4 1 ...
##  $ bottles_disposal     : Factor w/ 9 levels "1","2","3","4",..: 2 2 2 2 2 2 2 2 2 1 ...
##  $ film_disposal        : Factor w/ 9 levels "1","2","3","4",..: 9 1 1 2 1 2 1 1 1 1 ...
##  $ tubs_disposal        : Factor w/ 9 levels "1","2","3","4",..: 7 2 1 2 2 2 2 2 1 1 ...
##  $ bags_disposal        : Factor w/ 9 levels "1","2","3","4",..: 7 1 1 2 2 2 3 7 1 1 ...
##  $ attitude_disposal    : Factor w/ 7 levels "1","2","3","4",..: 4 7 5 3 4 4 5 5 5 4 ...
##  $ plasticwaste_ocean   : Factor w/ 5 levels "1","2","3","4",..: 3 5 3 3 3 4 4 4 4 5 ...
##  $ littering_portsmouth : Factor w/ 5 levels "1","2","3","4",..: 2 1 2 2 2 3 2 3 2 2 ...
##  $ barrier_recyling     : Factor w/ 12 levels "1","2","3","4",..: 3 7 12 2 1 4 1 6 2 4 ...
##  $ barrier_reducing     : Factor w/ 8 levels "1","2","3","4",..: 3 2 8 1 1 4 4 8 4 8 ...
##  $ main_con_plastic     : Factor w/ 11 levels "1","2","3","4",..: 3 1 1 1 1 1 2 2 3 3 ...
##  $ attitude_plastic     : Factor w/ 4 levels "1","2","3","4": 3 1 2 2 3 2 3 2 3 3 ...
##  $ choice_plastic       : Factor w/ 4 levels "1","2","3","4": 3 2 2 3 3 3 3 2 4 2 ...
##  $ individual_actions   : Factor w/ 5 levels "1","2","3","4",..: 3 1 2 1 3 3 2 2 2 5 ...
##  $ awareness_zerowaste  : Factor w/ 6 levels "1","2","3","4",..: 5 5 6 5 4 6 4 5 6 5 ...
```

```r
summary(data)
```

```
##   Response ID  gender   age     living_situation annual_income education_level
##  65     :  1   1:158   1:103   1: 92            6      :76    2:  2
##  66     :  1   2:239   2:176   2: 51            5      :52    3: 97
##  79     :  1   3:  3   3:121   3: 40            4      :46    4:130
##  80     :  1                   4:213            3      :45    5:135
##  81     :  1                   5:  4            7      :45    6: 36
##  83     :  1                                    1      :42
##  (Other):394                                    (Other):94
##  vehicle_ownership postcode bottles_avgweek film_avgweek tubs_avgweek
##  1:118             1:69     1: 12           1: 12        1: 12
##  2:282             2:66     2:137           2: 84        2:108
##                    3:49     3:134           3:122        3:143
##                    4:82     4: 74           4:113        4: 85
##                    5:72     5: 19           5: 39        5: 29
##                    6:62     6: 14           6: 18        6: 13
##                             7: 10           7: 12        7: 10
##  bags_avgweek bottles_reuses film_reuses tubs_reuses bags_reuses
##  1:232        1: 71          1:236       1: 61       1: 31
##  2: 77        2: 66          2: 83       2: 41       2: 44
##  3: 43        3:129          3: 54       3: 97       3: 93
##  4: 19        4: 57          4: 12       4: 58       4: 70
##  5: 14        5: 77          5: 15       5:143       5:162
##  6:  8
##  7:  7
##  bottles_disposal film_disposal tubs_disposal bags_disposal attitude_disposal
##  2      :328      1      :265   2      :211   1      :143   1: 27
##  1      : 27      2      : 92   1      : 86   2      : 92   2: 20
##  7      : 16      3      : 17   7      : 42   7      : 83   3: 41
##  3      : 10      4      :  6   3      : 29   3      : 29   4: 73
##  4      :  7      8      :  6   4      : 12   8      : 23   5:114
##  5      :  5      9      :  6   8      :  7   6      : 11   6: 56
##  (Other):  7      (Other):  8   (Other): 13   (Other): 19   7: 69
##  plasticwaste_ocean littering_portsmouth   barrier_recyling barrier_reducing
##  1: 19              1:173                  1      :115      1      :90
##  2:104              2:162                  2      : 62      2      :85
##  3:200              3: 53                  12     : 57      3      :85
##  4: 60              4:  8                  4      : 35      5      :40
##  5: 17              5:  4                  6      : 33      8      :40
##                                            3      : 31      4      :39
##                                            (Other): 67      (Other):21
##  main_con_plastic attitude_plastic choice_plastic individual_actions
##  1      :119      1:102            1: 46          1:142
##  2      : 97      2:218            2:208          2:191
##  3      : 88      3: 58            3:116          3: 43
##  4      : 26      4: 22            4: 30          4: 11
##  7      : 22                                      5: 13
##  5      : 14
##  (Other): 34
##  awareness_zerowaste
##  1: 14
##  2: 30
```

```
##   3: 24
##   4: 88
##   5:175
##   6: 69
##
```

Variable labeling

```
data$gender <- recode_factor(data$gender,
 "1" = "Male",
 "2" = "Female",
 "3" = "Other")
data$age <- recode_factor(data$age, "1" = "30 and under",
 "2" = "31-50",
 "3" = "50+")
data$living_situation <- recode_factor(data$living_situation,
 "1" = "I live alone",
 "2" = "I live with my parents/family",
 "3" = "I live in a house-/ flat-share",
 "4" = "I live with my partner/children",
 "5" = "Other")
data$annual_income <- recode_factor(data$annual_income,
 "1" = "Less than £12,000",
 "2" = "£12,000-14,999",
 "3" = "£15,000-19,999",
 "4" = "£20,000-24,999",
 "5" = "£25,000-34,999",
 "6" = "£35,000-49,999",
 "7" = "£50,000-74,999",
 "8" = "£100,000-149,000",
 "9" = "£15,000-19,999",
 "10" = "£150,000 or more",
 "11" = "Prefer not to say")
data$education_level <- recode_factor(data$education_level,
 "1" = "None",
 "2" = "Primary",
 "3" = "Secondary",
 "4" = "High school",
 "5" = "Undergraduate",
 "6" = "Postgraduate")
data$vehicle_ownership <- recode_factor(data$vehicle_ownership,
 "1" = "No vehicle",
 "2" = "1 or more vehicles")
data$postcode <- recode_factor(data$postcode,
"1" = "PO1",
"2" = "PO2",
"3" = "PO3",
"4" = "PO4",
"5" = "PO5",
"6" = "PO6")
```

Label check

See the recoded factor levels below.

```r
summary(data)
```

```
##   Response ID     gender              age     
##  65     :  1   Male  :158   30 and under:103  
##  66     :  1   Female:239   31-50       :176  
##  79     :  1   Other :  3   50+         :121  
##  80     :  1                                  
##  81     :  1                                  
##  83     :  1                                  
##  (Other):394                                  
##                          living_situation           annual_income
##  I live alone                    : 92     £35,000-49,999   :76   
##  I live with my parents/family   : 51     £15,000-19,999   :63   
##  I live in a house-/ flat-share  : 40     £25,000-34,999   :52   
##  I live with my partner/children:213     £20,000-24,999   :46   
##  Other                           :  4     £50,000-74,999   :45   
##                                           Less than £12,000:42   
##                                           (Other)          :76   
##       education_level          vehicle_ownership postcode     bottles_avgweek
##  Primary      :  2    No vehicle        :118     PO1:69   None        : 12  
##  Secondary    : 97    1 or more vehicles:282     PO2:66   1-2         :137  
##  High school  :130                               PO3:49   3-5         :134  
##  Undergraduate:135                               PO4:82   6-10        : 74  
##  Postgraduate : 36                               PO5:72   11-15       : 19  
##                                                  PO6:62   16+         : 14  
##                                                           I don't know: 10  
##        film_avgweek       tubs_avgweek       bags_avgweek    bottles_reuses
##  None        : 12   None        : 12   None        :232   Never     : 71  
##  1-2         : 84   1-2         :108   1-2         : 77   Once      : 66  
##  3-5         :122   3-5         :143   3-5         : 43   2-4 times :129  
##  6-10        :113   6-10        : 85   6-10        : 19   5-10 times: 57  
##  11-15       : 39   11-15       : 29   11-15       : 14   More often: 77  
##  16+         : 18   16+         : 13   16+         :  8                   
##  I don't know: 12   I don't know: 10   I don't know:  7                   
##      film_reuses      tubs_reuses      bags_reuses 
##  Never     :236   Never     : 61   Never     : 31  
##  Once      : 83   Once      : 41   Once      : 44  
##  2-4 times : 54   2-4 times : 97   2-4 times : 93  
##  5-10 times: 12   5-10 times: 58   5-10 times: 70  
##  More often: 15   More often:143   More often:162  
##                                                    
##                                                    
##                        bottles_disposal                      film_disposal
##  Recycling bin                :328     General waste bin           :265  
##  General waste bin            : 27     Recycling bin               : 92  
##  Indefinite storage           : 16     Recycling centre            : 17  
##  Recycling centre             : 10     Specialist waste collection:  6  
##  Specialist waste collection:  7     Other                       :  6  
##  Landfill                     :  5     I don't know                :  6  
##  (Other)                      :  7     (Other)                     :  8  
```

```
##                     tubs_disposal              bags_disposal
##  Recycling bin             :211   General waste bin    :143
##  General waste bin         : 86   Recycling bin        : 92
##  Indefinite storage        : 42   Indefinite storage   : 83
##  Recycling centre          : 29   Recycling centre     : 29
##  Specialist waste collection: 12   Other                : 23
##  Other                     :  7   Deposit return scheme: 11
##  (Other)                   : 13   (Other)              : 19
##         attitude_disposal       plasticwaste_ocean littering_portsmouth
##  1 (Not concerned): 27     Always          : 19      1:173
##  2               : 20     Most of the time:104      2:162
##  3               : 41     Sometimes       :200      3: 53
##  4               : 73     Rarely          : 60      4:  8
##  5               :114     Never           : 17      5:  4
##  6               : 56
##  7 (Very concerned: 69
##                 barrier_recyling                 barrier_reducing
##  Council collection      :115     Limited alternatives    :90
##  Unclear information     : 62     No SUP-free alternatives:85
##  Already doing everything: 57     Alternatives expensive  :85
##  No local facilities     : 35     Forgetting reusables    :40
##  Ends up in landfills    : 33     No barriers             :40
##  Difficult transport     : 31     Limited functioning     :39
##  (Other)                 : 67     (Other)                 :21
##                    main_con_plastic
##  Value for money           :119
##  Price                     : 97
##  Quality                   : 88
##  Deals/discounts           : 26
##  Ease of recycling packaging: 22
##  Use-by-dates/longevity    : 14
##  (Other)                   : 34
##                                   attitude_plastic
##  Will go out of their way to avoid         :102
##  If option is readily available, will avoid:218
##  Will avoid only without extra costs       : 58
##  Not a priority                            : 22
##
##
##
##                 choice_plastic          individual_actions awareness_zerowaste
##  Always                 : 46    Yes, definitely   :142      1: 14
##  As often as they can   :208    Yes, probably     :191      2: 30
##  If cheaper or preferred:116    No, probably not  : 43      3: 24
##  Rarely or never        : 30    No, definitely not: 11      4: 88
##                                 I don't know      : 13      5:175
##                                                             6: 69
##
##                      littering                              zerowaste
##  Strongly agree            :173   Yes, shops regularly           : 14
##  Agree                     :162   Yes, shops occasionally        : 30
##  Neither agree nor disagree: 53   Yes, visited at least once     : 24
##  Disagree                  :  8   Yes, never visited             : 88
##  Strongly disagree         :  4   No, but would like to shop there:175
```

```
##                                    No, not likely to shop there    : 69
##
```

Statistical analysis

Normality and chosen tests

The data is categorical-ordinal. Categorical data is not normally distributed and, thus, non-parametric tests were chosen.

Pearson's Chi-squared tests for independence were chosen. They were first run as normal without simulations. Monte Carlo simulation was then used if the cell size was too small.

Crosstab compilations

'Age' factor:

```
age.choice <- table(data$age, data$choice_plastic)
age.barrier <- table(data$age, data$barrier_recyling)
age.barrier2 <- table(data$age, data$barrier_reducing)
age.mainconsid <- table(data$age, data$main_con_plastic)
age.litter <- table(data$age, data$littering)
age.attitude <- table(data$age, data$attitude_disposal)
age.ocean <- table(data$age, data$plasticwaste_ocean)
age.actions <- table(data$age, data$individual_actions)
age.attplast <- table(data$age, data$attitude_plastic)
age.zerow <- table(data$age, data$zerowaste)

## SUPs
age.bottpurchase <- table(data$age, data$bottles_avgweek)
age.bottreuse <- table(data$age, data$bottles_reuses)
age.bottdisp <- table(data$age, data$bottles_disposal)
age.tubpurchase <- table(data$age, data$tubs_avgweek)
age.tubreuse <- table(data$age, data$tubs_reuses)
age.tubdisp <- table(data$age, data$tubs_disposal)
age.filmpur <- table(data$age, data$film_avgweek)
age.filmreuse <- table(data$age, data$film_reuses)
age.filmdisp <- table(data$age, data$film_disposal)
age.bagpurchase <-table(data$age, data$bags_avgweek)
age.bagreuse <- table(data$age, data$bags_reuses)
age.bagdisp <- table(data$age, data$bags_disposal)
```

```
addmargins(age.choice)
```

```
##
##                 Always As often as they can If cheaper or preferred
##   30 and under     13                    47                      36
##   31-50            23                    94                      47
##   50+              10                    67                      33
```

```
##   Sum              46                   208                        116
##
##                 Rarely or never Sum
##   30 and under                7 103
##   31-50                      12 176
##   50+                        11 121
##   Sum                        30 400
```

```r
addmargins(age.barrier)
```

```
##
##                 Council collection Unclear information Difficult transport
##   30 and under                  16                  27                   7
##   31-50                         53                  17                  14
##   50+                           46                  18                  10
##   Sum                          115                  62                  31
##
##                 No local facilities No support Ends up in landfills Forgetting
##   30 and under                    7          4                    9         13
##   31-50                          22         12                   18          9
##   50+                             6          2                    6          3
##   Sum                            35         18                   33         25
##
##                 Recycling a hassle Household disagrees Recycling not important
##   30 and under                   4                   3                       1
##   31-50                          4                   2                       3
##   50+                            2                   0                       1
##   Sum                           10                   5                       5
##
##                 Other Already doing everything Sum
##   30 and under      1                       11 103
##   31-50             2                       20 176
##   50+               1                       26 121
##   Sum               4                       57 400
```

```r
addmargins(age.barrier2)
```

```
##
##                 Limited alternatives No SUP-free alternatives
##   30 and under                    18                       24
##   31-50                           35                       31
##   50+                             37                       30
##   Sum                             90                       85
##
##                 Alternatives expensive Limited functioning Forgetting reusables
##   30 and under                      22                  18                    9
##   31-50                             51                  13                   23
##   50+                               12                   8                    8
##   Sum                               85                  39                   40
##
##                 Reducing not important Other No barriers Sum
##   30 and under                       6     1           5 103
##   31-50                              5     2          16 176
```

```
##   50+                                2      5           19  121
##   Sum                               13      8           40  400
```

```r
addmargins(age.mainconsid)
```

```
##
##                  Value for money Price Quality Deals/discounts
##   30 and under                32    32      14               6
##   31-50                       46    51      37              15
##   50+                         41    14      37               5
##   Sum                        119    97      88              26
##
##                  Use-by-dates/longevity Convenience Ease of recycling packaging
##   30 and under                        4           4                           4
##   31-50                               4           1                          10
##   50+                                 6           5                           8
##   Sum                                14          10                          22
##
##                  Sustainability Brand Ethics Other Sum
##   30 and under                3     1      3     0 103
##   31-50                       9     3      0     0 176
##   50+                         1     0      2     2 121
##   Sum                        13     4      5     2 400
```

```r
addmargins(age.litter)
```

```
##
##                  Strongly agree Agree Neither agree nor disagree Disagree
##   30 and under               45    40                         14        2
##   31-50                      66    83                         21        5
##   50+                        62    39                         18        1
##   Sum                       173   162                         53        8
##
##                  Strongly disagree Sum
##   30 and under                   2 103
##   31-50                          1 176
##   50+                            1 121
##   Sum                            4 400
```

```r
addmargins(age.attitude)
```

```
##
##                  1 (Not concerned)   2   3   4   5   6 7 (Very concerned Sum
##   30 and under                  13   7  15  21  28  11                 8 103
##   31-50                          7   9  19  36  51  25                29 176
##   50+                            7   4   7  16  35  20                32 121
##   Sum                           27  20  41  73 114  56                69 400
```

```r
addmargins(age.ocean)
```

```
##
```

```
##                 Always Most of the time Sometimes Rarely Never Sum
##   30 and under      9               38        48      6     2 103
##   31-50             7               40        91     33     5 176
##   50+               3               26        61     21    10 121
##   Sum              19              104       200     60    17 400
```

```r
addmargins(age.actions)
```

```
##
##                 Yes, definitely Yes, probably No, probably not
##   30 and under               33            52                9
##   31-50                      67            84               19
##   50+                        42            55               15
##   Sum                       142           191               43
##
##                 No, definitely not I don't know Sum
##   30 and under                   5            4 103
##   31-50                          2            4 176
##   50+                            4            5 121
##   Sum                           11           13 400
```

```r
addmargins(age.attplast)
```

```
##
##                 Will go out of their way to avoid
##   30 and under                                 26
##   31-50                                        47
##   50+                                          29
##   Sum                                         102
##
##                 If option is readily available, will avoid
##   30 and under                                          58
##   31-50                                                 99
##   50+                                                   61
##   Sum                                                  218
##
##                 Will avoid only without extra costs Not a priority Sum
##   30 and under                                   15              4 103
##   31-50                                          22              8 176
##   50+                                            21             10 121
##   Sum                                            58             22 400
```

```r
addmargins(age.zerow)
```

```
##
##                 Yes, shops regularly Yes, shops occasionally
##   30 and under                     4                       7
##   31-50                           10                      20
##   50+                              0                       3
##   Sum                             14                      30
##
##                 Yes, visited at least once Yes, never visited
```

```
##   30 and under                          4                  29
##   31-50                                16                  36
##   50+                                   4                  23
##   Sum                                  24                  88
##
##                No, but would like to shop there No, not likely to shop there
##   30 and under                               51                            8
##   31-50                                      70                           24
##   50+                                        54                           37
##   Sum                                       175                           69
##
##                Sum
##   30 and under 103
##   31-50        176
##   50+          121
##   Sum          400
```

```
## SUPs
addmargins(age.bottpurchase)
```

```
##
##                None 1-2 3-5 6-10 11-15 16+ I don't know Sum
##   30 and under    2  31  35   20     5   6            4 103
##   31-50           3  48  66   38     8   8            5 176
##   50+             7  58  33   16     6   0            1 121
##   Sum            12 137 134   74    19  14           10 400
```

```
addmargins(age.bottreuse)
```

```
##
##                Never Once 2-4 times 5-10 times More often Sum
##   30 and under    11   16        39         16         21 103
##   31-50           33   33        56         21         33 176
##   50+             27   17        34         20         23 121
##   Sum             71   66       129         57         77 400
```

```
addmargins(age.bottdisp)
```

```
##
##                General waste bin Recycling bin Recycling centre
##   30 and under                 8            78                6
##   31-50                       14           144                2
##   50+                          5           106                2
##   Sum                         27           328               10
##
##                Specialist waste collection Landfill Deposit return scheme
##   30 and under                           1        1                     1
##   31-50                                  5        4                     3
##   50+                                    1        0                     0
##   Sum                                    7        5                     4
##
##                Indefinite storage Other I don't know Sum
```

```
##    30 and under                   7     0             1 103
##    31-50                          4     0             0 176
##    50+                            5     2             0 121
##    Sum                           16     2             1 400
```

```r
addmargins(age.tubpurchase)
```

```
##
##                  None 1-2 3-5 6-10 11-15 16+ I don't know Sum
##    30 and under     4  24  39   20     9   5            2 103
##    31-50            4  38  64   45    13   6            6 176
##    50+              4  46  40   20     7   2            2 121
##    Sum             12 108 143   85    29  13           10 400
```

```r
addmargins(age.tubreuse)
```

```
##
##                  Never Once 2-4 times 5-10 times More often Sum
##    30 and under     11    9        20         18         45 103
##    31-50            24   20        50         30         52 176
##    50+              26   12        27         10         46 121
##    Sum              61   41        97         58        143 400
```

```r
addmargins(age.tubdisp)
```

```
##
##                  General waste bin Recycling bin Recycling centre
##    30 and under                 17            46               10
##    31-50                        41           101                9
##    50+                          28            64               10
##    Sum                          86           211               29
##
##                  Specialist waste collection Landfill Deposit return scheme
##    30 and under                            8        1                     1
##    31-50                                   4        2                     3
##    50+                                     0        0                     0
##    Sum                                    12        3                     4
##
##                  Indefinite storage Other I don't know Sum
##    30 and under                  15     1            4 103
##    31-50                         12     3            1 176
##    50+                           15     3            1 121
##    Sum                           42     7            6 400
```

```r
addmargins(age.filmpur)
```

```
##
##                  None 1-2 3-5 6-10 11-15 16+ I don't know Sum
##    30 and under     2  19  29   30    13   7            3 103
##    31-50            3  27  55   57    18   9            7 176
##    50+              7  38  38   26     8   2            2 121
##    Sum             12  84 122  113    39  18           12 400
```

```r
addmargins(age.filmreuse)
```

```
##
##                Never Once 2-4 times 5-10 times More often Sum
##   30 and under    51   21        21          6          4 103
##   31-50           95   40        28          4          9 176
##   50+             90   22         5          2          2 121
##   Sum            236   83        54         12         15 400
```

```r
addmargins(age.filmdisp)
```

```
##
##                General waste bin Recycling bin Recycling centre
##   30 and under                60            28                8
##   31-50                      107            46                9
##   50+                         98            18                0
##   Sum                        265            92               17
##
##                Specialist waste collection Landfill Deposit return scheme
##   30 and under                           2        1                     0
##   31-50                                  4        2                     2
##   50+                                    0        0                     0
##   Sum                                    6        3                     2
##
##                Indefinite storage Other I don't know Sum
##   30 and under                  1     1            2 103
##   31-50                         2     3            1 176
##   50+                           0     2            3 121
##   Sum                           3     6            6 400
```

```r
addmargins(age.bagpurchase)
```

```
##
##                None 1-2 3-5 6-10 11-15 16+ I don't know Sum
##   30 and under   41  26  21    7     4   1            3 103
##   31-50          88  36  21   12    10   7            2 176
##   50+           103  15   1    0     0   0            2 121
##   Sum           232  77  43   19    14   8            7 400
```

```r
addmargins(age.bagreuse)
```

```
##
##                Never Once 2-4 times 5-10 times More often Sum
##   30 and under     5    9        28         16         45 103
##   31-50           12   28        43         32         61 176
##   50+             14    7        22         22         56 121
##   Sum             31   44        93         70        162 400
```

```r
addmargins(age.bagdisp)
```

```
##
##                 General waste bin Recycling bin Recycling centre
##   30 and under                 37            21               11
##   31-50                        61            49               12
##   50+                          45            22                6
##   Sum                         143            92               29
##
##                 Specialist waste collection Landfill Deposit return scheme
##   30 and under                           3        0                     2
##   31-50                                  5        2                     8
##   50+                                    0        0                     1
##   Sum                                    8        2                    11
##
##                 Indefinite storage Other I don't know Sum
##   30 and under                 23     2            4 103
##   31-50                        26    10            3 176
##   50+                          34    11            2 121
##   Sum                          83    23            9 400
```

'Education' factor:

```
edu.choice <- table(data$education_level, data$choice_plastic)
edu.barrier <- table(data$education_level, data$barrier_recyling)
edu.barrier2 <- table(data$education_level, data$barrier_reducing)
edu.mainconsid <- table(data$education_level, data$main_con_plastic)
edu.litter <- table(data$education_level, data$littering)
edu.attitude <- table(data$education_level, data$attitude_disposal)
edu.ocean <- table(data$education_level, data$plasticwaste_ocean)
edu.actions <- table(data$education_level, data$individual_actions)
edu.attplast <- table(data$education_level, data$attitude_plastic)
edu.zerow <- table(data$education_level, data$zerowaste)



# SUPs
edu.bottpurchase <- table(data$education_level, data$bottles_avgweek)
edu.bottreuse <- table(data$education_level, data$bottles_reuses)
edu.bottdisp <- table(data$education_level, data$bottles_disposal)
edu.tubspurchase <- table(data$education_level, data$tubs_avgweek)
edu.tubsreuse <- table(data$education_level, data$tubs_reuses)
edu.tubdisp <- table(data$education_level, data$tubs_disposal)
edu.filmpurchase <- table(data$education_level, data$film_avgweek)
edu.filmreuse <- table(data$education_level, data$film_reuses)
edu.filmdisp <- table(data$education_level, data$film_disposal)
edu.bagpurchase <- table(data$education_level, data$bags_avgweek)
edu.bagreuse <- table(data$education_level, data$bags_reuses)
edu.bagdisp <- table(data$education_level, data$bags_disposal)
```

```
addmargins(edu.choice)
```

```
##
```

```
##                 Always As often as they can If cheaper or preferred
##   Primary            1                    1                        0
##   Secondary          7                   50                       31
##   High school        9                   67                       43
##   Undergraduate     23                   71                       32
##   Postgraduate       6                   19                       10
##   Sum               46                  208                      116
##
##                 Rarely or never Sum
##   Primary                     0   2
##   Secondary                   9  97
##   High school                11 130
##   Undergraduate               9 135
##   Postgraduate                1  36
##   Sum                        30 400
```

```
addmargins(edu.barrier)
```

```
##
##                 Council collection Unclear information Difficult transport
##   Primary                        1                   0                   0
##   Secondary                     22                  20                   7
##   High school                   37                  23                   8
##   Undergraduate                 44                  14                  14
##   Postgraduate                  11                   5                   2
##   Sum                          115                  62                  31
##
##                 No local facilities No support Ends up in landfills Forgetting
##   Primary                         1          0                    0          0
##   Secondary                       9          1                    8          5
##   High school                     8          5                    9          8
##   Undergraduate                  15          8                   13          8
##   Postgraduate                    2          4                    3          4
##   Sum                            35         18                   33         25
##
##                 Recycling a hassle Household disagrees Recycling not important
##   Primary                        0                   0                       0
##   Secondary                      3                   0                       1
##   High school                    3                   2                       1
##   Undergraduate                  3                   2                       1
##   Postgraduate                   1                   1                       2
##   Sum                           10                   5                       5
##
##                 Other Already doing everything Sum
##   Primary           0                        0   2
##   Secondary         1                       20  97
##   High school       3                       23 130
##   Undergraduate     0                       13 135
##   Postgraduate      0                        1  36
##   Sum               4                       57 400
```

```
addmargins(edu.barrier2)
```

```
##
##                  Limited alternatives No SUP-free alternatives
##   Primary                           2                        0
##   Secondary                        22                       13
##   High school                      35                       29
##   Undergraduate                    23                       31
##   Postgraduate                      8                       12
##   Sum                              90                       85
##
##                  Alternatives expensive Limited functioning Forgetting reusables
##   Primary                             0                   0                    0
##   Secondary                          22                   7                   10
##   High school                        23                  12                   16
##   Undergraduate                      34                  16                   13
##   Postgraduate                        6                   4                    1
##   Sum                                85                  39                   40
##
##                  Reducing not important Other No barriers Sum
##   Primary                             0     0           0   2
##   Secondary                           5     3          15  97
##   High school                         2     1          12 130
##   Undergraduate                       3     4          11 135
##   Postgraduate                        3     0           2  36
##   Sum                                13     8          40 400
```

```r
addmargins(edu.mainconsid)
```

```
##
##                  Value for money Price Quality Deals/discounts
##   Primary                      0     0       0               0
##   Secondary                   27    27      18               7
##   High school                 40    29      30               7
##   Undergraduate               41    34      28              10
##   Postgraduate                11     7      12               2
##   Sum                        119    97      88              26
##
##                  Use-by-dates/longevity Convenience Ease of recycling packaging
##   Primary                             1           0                           0
##   Secondary                           2           3                           6
##   High school                         7           3                          10
##   Undergraduate                       3           4                           5
##   Postgraduate                        1           0                           1
##   Sum                                14          10                          22
##
##                  Sustainability Brand Ethics Other Sum
##   Primary                     1     0      0     0   2
##   Secondary                   2     2      1     2  97
##   High school                 1     1      2     0 130
##   Undergraduate               8     1      1     0 135
##   Postgraduate                1     0      1     0  36
##   Sum                        13     4      5     2 400
```

```r
addmargins(edu.litter)
```

```
##
##                  Strongly agree Agree Neither agree nor disagree Disagree
##   Primary                     0     2                          0        0
##   Secondary                  42    37                         17        1
##   High school                57    54                         16        2
##   Undergraduate              56    55                         16        5
##   Postgraduate               18    14                          4        0
##   Sum                       173   162                         53        8
##
##                  Strongly disagree Sum
##   Primary                        0   2
##   Secondary                      0  97
##   High school                    1 130
##   Undergraduate                  3 135
##   Postgraduate                   0  36
##   Sum                            4 400
```

```r
addmargins(edu.attitude)
```

```
##
##                  1 (Not concerned)   2   3   4   5   6 7 (Very concerned Sum
##   Primary                        0   0   1   0   0   1                 0   2
##   Secondary                      9   8   7  15  25  12                21  97
##   High school                    7   1  19  28  40  16                19 130
##   Undergraduate                  8  10  14  25  36  18                24 135
##   Postgraduate                   3   1   0   5  13   9                 5  36
##   Sum                           27  20  41  73 114  56                69 400
```

```r
addmargins(edu.ocean)
```

```
##
##                  Always Most of the time Sometimes Rarely Never Sum
##   Primary             0                1         1      0     0   2
##   Secondary           5               20        50     16     6  97
##   High school         8               31        72     17     2 130
##   Undergraduate       5               41        62     21     6 135
##   Postgraduate        1               11        15      6     3  36
##   Sum                19              104       200     60    17 400
```

```r
addmargins(edu.actions)
```

```
##
##                  Yes, definitely Yes, probably No, probably not
##   Primary                      2             0                0
##   Secondary                   30            46               15
##   High school                 45            62               15
##   Undergraduate               55            62               11
##   Postgraduate                10            21                2
##   Sum                        142           191               43
```

```
##
##                  No, definitely not I don't know Sum
##   Primary                         0            0   2
##   Secondary                       2            4  97
##   High school                     5            3 130
##   Undergraduate                   2            5 135
##   Postgraduate                    2            1  36
##   Sum                            11           13 400
```

```r
addmargins(edu.attplast)
```

```
##
##                  Will go out of their way to avoid
##   Primary                                        0
##   Secondary                                     17
##   High school                                   35
##   Undergraduate                                 36
##   Postgraduate                                  14
##   Sum                                          102
##
##                  If option is readily available, will avoid
##   Primary                                                 2
##   Secondary                                              54
##   High school                                            71
##   Undergraduate                                          76
##   Postgraduate                                           15
##   Sum                                                   218
##
##                  Will avoid only without extra costs Not a priority Sum
##   Primary                                          0              0   2
##   Secondary                                       19              7  97
##   High school                                     16              8 130
##   Undergraduate                                   17              6 135
##   Postgraduate                                     6              1  36
##   Sum                                             58             22 400
```

```r
addmargins(edu.zerow)
```

```
##
##                  Yes, shops regularly Yes, shops occasionally
##   Primary                           1                       0
##   Secondary                         3                       4
##   High school                       3                       7
##   Undergraduate                     4                      14
##   Postgraduate                      3                       5
##   Sum                              14                      30
##
##                  Yes, visited at least once Yes, never visited
##   Primary                                 0                  0
##   Secondary                               3                 17
##   High school                             8                 29
##   Undergraduate                           9                 38
##   Postgraduate                            4                  4
```

```
##    Sum                                 24                 88
##
##                 No, but would like to shop there No, not likely to shop there
##    Primary                                    1                            0
##    Secondary                                 48                           22
##    High school                               60                           23
##    Undergraduate                             51                           19
##    Postgraduate                              15                            5
##    Sum                                      175                           69
##
##                 Sum
##    Primary         2
##    Secondary      97
##    High school   130
##    Undergraduate 135
##    Postgraduate   36
##    Sum           400
```

```
## SUPs
addmargins(edu.bottpurchase)
```

```
##
##                 None 1-2 3-5 6-10 11-15 16+ I don't know Sum
##    Primary          0   0   1    1     0   0            0   2
##    Secondary        2  36  29   21     6   1            2  97
##    High school      5  44  44   22     6   3            6 130
##    Undergraduate    3  46  49   23     6   6            2 135
##    Postgraduate     2  11  11    7     1   4            0  36
##    Sum             12 137 134   74    19  14           10 400
```

```
addmargins(edu.bottreuse)
```

```
##
##                 Never Once 2-4 times 5-10 times More often Sum
##    Primary           1    0         1          0          0   2
##    Secondary        23   18        23         20         13  97
##    High school      23   22        39         18         28 130
##    Undergraduate    19   21        53         15         27 135
##    Postgraduate      5    5        13          4          9  36
##    Sum              71   66       129         57         77 400
```

```
addmargins(edu.bottdisp)
```

```
##
##                 General waste bin Recycling bin Recycling centre
##    Primary                      0             1                0
##    Secondary                    7            86                1
##    High school                  8           105                3
##    Undergraduate                9           108                5
##    Postgraduate                 3            28                1
##    Sum                         27           328               10
##
```

```
##                  Specialist waste collection Landfill Deposit return scheme
##   Primary                                  0        0                     1
##   Secondary                                1        1                     0
##   High school                              2        0                     2
##   Undergraduate                            2        4                     1
##   Postgraduate                             2        0                     0
##   Sum                                      7        5                     4
##
##                  Indefinite storage Other I don't know Sum
##   Primary                         0     0            0   2
##   Secondary                       0     0            1  97
##   High school                     9     1            0 130
##   Undergraduate                   5     1            0 135
##   Postgraduate                    2     0            0  36
##   Sum                            16     2            1 400
```

```
addmargins(edu.tubspurchase)
```

```
##
##                  None 1-2 3-5 6-10 11-15 16+ I don't know Sum
##   Primary           0   0   2    0     0   0            0   2
##   Secondary         5  28  41   12     8   1            2  97
##   High school       4  35  43   32    10   2            4 130
##   Undergraduate     3  38  43   30     9   8            4 135
##   Postgraduate      0   7  14   11     2   2            0  36
##   Sum              12 108 143   85    29  13           10 400
```

```
addmargins(edu.tubsreuse)
```

```
##
##                  Never Once 2-4 times 5-10 times More often Sum
##   Primary            0    0         1          0          1   2
##   Secondary         18   14        24         14         27  97
##   High school       12   13        32         17         56 130
##   Undergraduate     28    9        33         18         47 135
##   Postgraduate       3    5         7          9         12  36
##   Sum               61   41        97         58        143 400
```

```
addmargins(edu.tubdisp)
```

```
##
##                  General waste bin Recycling bin Recycling centre
##   Primary                        0             2                0
##   Secondary                     24            55                3
##   High school                   22            68                9
##   Undergraduate                 33            68               12
##   Postgraduate                   7            18                5
##   Sum                           86           211               29
##
##                  Specialist waste collection Landfill Deposit return scheme
##   Primary                                  0        0                     0
##   Secondary                                3        1                     0
```

```
##   High school                          5        0                     2
##   Undergraduate                        3        1                     2
##   Postgraduate                         1        1                     0
##   Sum                                 12        3                     4
##
##                  Indefinite storage Other I don't know Sum
##   Primary                         0     0            0   2
##   Secondary                       9     2            0  97
##   High school                    19     0            5 130
##   Undergraduate                  10     5            1 135
##   Postgraduate                    4     0            0  36
##   Sum                            42     7            6 400
```

```
addmargins(edu.filmpurchase)
```

```
##
##                  None 1-2 3-5 6-10 11-15 16+ I don't know Sum
##   Primary           0   1   0    1     0   0            0   2
##   Secondary         3  20  29   31    10   2            2  97
##   High school       5  29  36   38    13   5            4 130
##   Undergraduate     4  29  41   37    11   8            5 135
##   Postgraduate      0   5  16    6     5   3            1  36
##   Sum              12  84 122  113    39  18           12 400
```

```
addmargins(edu.filmreuse)
```

```
##
##                  Never Once 2-4 times 5-10 times More often Sum
##   Primary            0    0         1          0          1   2
##   Secondary         62   20         8          6          1  97
##   High school       84   27        13          2          4 130
##   Undergraduate     70   30        22          4          9 135
##   Postgraduate      20    6        10          0          0  36
##   Sum              236   83        54         12         15 400
```

```
addmargins(edu.filmdisp)
```

```
##
##                  General waste bin Recycling bin Recycling centre
##   Primary                        0             1                0
##   Secondary                     71            18                4
##   High school                   89            34                1
##   Undergraduate                 85            28               11
##   Postgraduate                  20            11                1
##   Sum                          265            92               17
##
##                  Specialist waste collection Landfill Deposit return scheme
##   Primary                                  0        0                     0
##   Secondary                                0        0                     0
##   High school                              2        0                     1
##   Undergraduate                            3        1                     1
##   Postgraduate                             1        2                     0
```

```
##   Sum                                    6        3                     2
##
##                 Indefinite storage Other I don't know Sum
##   Primary                        0     1            0   2
##   Secondary                      0     1            3  97
##   High school                    1     0            2 130
##   Undergraduate                  2     3            1 135
##   Postgraduate                   0     1            0  36
##   Sum                            3     6            6 400
```

```
addmargins(edu.bagpurchase)
```

```
##
##                 None 1-2 3-5 6-10 11-15 16+ I don't know Sum
##   Primary          1   0   1    0     0   0            0   2
##   Secondary       53  18  15    3     5   1            2  97
##   High school     78  22  15    6     4   2            3 130
##   Undergraduate   79  32   7    7     4   4            2 135
##   Postgraduate    21   5   5    3     1   1            0  36
##   Sum            232  77  43   19    14   8            7 400
```

```
addmargins(edu.bagreuse)
```

```
##
##                 Never Once 2-4 times 5-10 times More often Sum
##   Primary           0    0         1          0          1   2
##   Secondary        11   12        24         18         32  97
##   High school      10   13        29         21         57 130
##   Undergraduate     8   16        31         26         54 135
##   Postgraduate      2    3         8          5         18  36
##   Sum              31   44        93         70        162 400
```

```
addmargins(edu.bagdisp)
```

```
##
##                 General waste bin Recycling bin Recycling centre
##   Primary                       1             1                0
##   Secondary                    43            21                5
##   High school                  48            31                6
##   Undergraduate                37            33               14
##   Postgraduate                 14             6                4
##   Sum                         143            92               29
##
##                 Specialist waste collection Landfill Deposit return scheme
##   Primary                                 0        0                     0
##   Secondary                               2        0                     1
##   High school                             3        0                     3
##   Undergraduate                           3        1                     6
##   Postgraduate                            0        1                     1
##   Sum                                     8        2                    11
##
##                 Indefinite storage Other I don't know Sum
```

```
##    Primary                          0     0           0   2
##    Secondary                       21     1           3  97
##    High school                     31     4           4 130
##    Undergraduate                   23    16           2 135
##    Postgraduate                     8     2           0  36
##    Sum                             83    23           9 400
```

'Gender' factor:

```
gen.choice <- table(data$gender, data$choice_plastic)
gen.barrier <- table(data$gender, data$barrier_recyling)
gen.barrier2 <- table(data$gender, data$barrier_reducing)
gen.mainconsid <- table(data$gender, data$main_con_plastic)
gen.litter <- table(data$gender, data$littering)
gen.attitude <- table(data$gender, data$attitude_disposal)
gen.ocean <- table(data$gender, data$plasticwaste_ocean)
gen.actions <- table(data$gender, data$individual_actions)
gen.attplast <- table(data$gender, data$attitude_plastic)
gen.zerow <- table(data$gender, data$zerowaste)

## SUPs
gen.bottpurchase <- table(data$gender, data$bottles_avgweek)
gen.bottreuse <- table(data$gender, data$bottles_reuses)
gen.bottdisp <- table(data$gender, data$bottles_disposal)
gen.tubspurchase <- table(data$gender, data$tubs_avgweek)
gen.tubreuse <- table(data$gender, data$tubs_reuses)
gen.tubdisp <- table(data$gender, data$tubs_disposal)
gen.filmpurchase <- table(data$gender, data$film_avgweek)
gen.filmreuse <- table(data$gender, data$film_reuses)
gen.filmdisp <- table(data$gender, data$film_disposal)
gen.bagpurchase <- table(data$gender, data$bags_avgweek)
gen.bagreuse <- table(data$gender, data$bags_reuses)
gen.bagdisp <- table(data$gender, data$bags_disposal)
```

```
addmargins(gen.choice)
```

```
##
##           Always As often as they can If cheaper or preferred Rarely or never
##    Male       21                   75                      46              16
##    Female     25                  132                      69              13
##    Other       0                    1                       1               1
##    Sum        46                  208                     116              30
##
##           Sum
##    Male   158
##    Female 239
##    Other    3
##    Sum    400
```

```r
addmargins(gen.barrier)
```

```
##
##         Council collection Unclear information Difficult transport
##   Male                  47                  18                  10
##   Female                68                  44                  21
##   Other                  0                   0                   0
##   Sum                  115                  62                  31
##
##         No local facilities No support Ends up in landfills Forgetting
##   Male                   10         10                   14         15
##   Female                 25          8                   18          9
##   Other                   0          0                    1          1
##   Sum                    35         18                   33         25
##
##         Recycling a hassle Household disagrees Recycling not important Other
##   Male                   5                   2                       2     1
##   Female                 5                   3                       3     3
##   Other                  0                   0                       0     0
##   Sum                   10                   5                       5     4
##
##         Already doing everything Sum
##   Male                        24 158
##   Female                      32 239
##   Other                        1   3
##   Sum                         57 400
```

```r
addmargins(gen.barrier2)
```

```
##
##         Limited alternatives No SUP-free alternatives Alternatives expensive
##   Male                    39                       40                     27
##   Female                  50                       45                     57
##   Other                    1                        0                      1
##   Sum                     90                       85                     85
##
##         Limited functioning Forgetting reusables Reducing not important Other
##   Male                   18                    8                      5     4
##   Female                 21                   32                      8     4
##   Other                   0                    0                      0     0
##   Sum                    39                   40                     13     8
##
##         No barriers Sum
##   Male           17 158
##   Female         22 239
##   Other           1   3
##   Sum            40 400
```

```r
addmargins(gen.mainconsid)
```

```
##
```

```
##          Value for money Price Quality Deals/discounts Use-by-dates/longevity
##   Male                47    33      43               7                      3
##   Female              70    63      45              19                     11
##   Other                2     1       0               0                      0
##   Sum                119    97      88              26                     14
##
##          Convenience Ease of recycling packaging Sustainability Brand Ethics
##   Male             4                          11              5     2      1
##   Female           6                          11              8     2      4
##   Other            0                           0              0     0      0
##   Sum             10                          22             13     4      5
##
##          Other Sum
##   Male       2 158
##   Female     0 239
##   Other      0   3
##   Sum        2 400
```

```
addmargins(gen.litter)
```

```
##
##          Strongly agree Agree Neither agree nor disagree Disagree
##   Male               57    74                         23        2
##   Female            114    88                         29        6
##   Other               2     0                          1        0
##   Sum               173   162                         53        8
##
##          Strongly disagree Sum
##   Male                   2 158
##   Female                 2 239
##   Other                  0   3
##   Sum                    4 400
```

```
addmargins(gen.attitude)
```

```
##
##          1 (Not concerned)   2   3   4   5   6 7 (Very concerned Sum
##   Male                  11   9  15  23  43  26                31 158
##   Female                15  11  26  50  70  29                38 239
##   Other                  1   0   0   0   1   1                 0   3
##   Sum                   27  20  41  73 114  56                69 400
```

```
addmargins(gen.ocean)
```

```
##
##          Always Most of the time Sometimes Rarely Never Sum
##   Male        6               41        75     29     7 158
##   Female     13               61       125     31     9 239
##   Other       0                2         0      0     1   3
##   Sum        19              104       200     60    17 400
```

```r
addmargins(gen.actions)
```

```
##
##          Yes, definitely Yes, probably No, probably not No, definitely not
##   Male                58            71                19                  5
##   Female              83           119                24                  5
##   Other                1             1                 0                  1
##   Sum                142           191                43                 11
##
##          I don't know Sum
##   Male              5 158
##   Female            8 239
##   Other             0   3
##   Sum              13 400
```

```r
addmargins(gen.attplast)
```

```
##
##          Will go out of their way to avoid
##   Male                                  37
##   Female                                64
##   Other                                  1
##   Sum                                  102
##
##          If option is readily available, will avoid
##   Male                                           89
##   Female                                        128
##   Other                                           1
##   Sum                                           218
##
##          Will avoid only without extra costs Not a priority Sum
##   Male                                    22             10 158
##   Female                                  36             11 239
##   Other                                    0              1   3
##   Sum                                     58             22 400
```

```r
addmargins(gen.zerow)
```

```
##
##          Yes, shops regularly Yes, shops occasionally
##   Male                      7                      15
##   Female                    7                      14
##   Other                     0                       1
##   Sum                      14                      30
##
##          Yes, visited at least once Yes, never visited
##   Male                            13                 30
##   Female                          11                 57
##   Other                            0                  1
##   Sum                             24                 88
##
```

```
##          No, but would like to shop there No, not likely to shop there Sum
##   Male                                55                          38 158
##   Female                             119                          31 239
##   Other                                1                           0   3
##   Sum                                175                          69 400
```

```r
## SUPs
addmargins(gen.bottpurchase)
```

```
##
##          None 1-2 3-5 6-10 11-15 16+ I don't know Sum
##   Male      7  47  49   33    12   8            2 158
##   Female    5  90  85   39     7   6            7 239
##   Other     0   0   0    2     0   0            1   3
##   Sum      12 137 134   74    19  14           10 400
```

```r
addmargins(gen.bottreuse)
```

```
##
##          Never Once 2-4 times 5-10 times More often Sum
##   Male      34   31        48         22         23 158
##   Female    36   34        81         35         53 239
##   Other      1    1         0          0          1   3
##   Sum       71   66       129         57         77 400
```

```r
addmargins(gen.bottdisp)
```

```
##
##          General waste bin Recycling bin Recycling centre
##   Male                   8           134                5
##   Female                19           193                5
##   Other                  0             1                0
##   Sum                   27           328               10
##
##          Specialist waste collection Landfill Deposit return scheme
##   Male                             3        3                      2
##   Female                           4        1                      2
##   Other                            0        1                      0
##   Sum                              7        5                      4
##
##          Indefinite storage Other I don't know Sum
##   Male                    3     0            0 158
##   Female                 13     2            0 239
##   Other                   0     0            1   3
##   Sum                    16     2            1 400
```

```r
addmargins(gen.tubspurchase)
```

```
##
##          None 1-2 3-5 6-10 11-15 16+ I don't know Sum
##   Male      8  44  54   33     8   8            3 158
```

```
##   Female     4  63  88   52    21   5            6 239
##   Other      0   1   1    0     0   0            1   3
##   Sum       12 108 143   85    29  13           10 400
```

```r
addmargins(gen.tubreuse)
```

```
##
##          Never Once 2-4 times 5-10 times More often Sum
##   Male      32   21        38         17         50 158
##   Female    29   20        59         39         92 239
##   Other      0    0         0          2          1   3
##   Sum       61   41        97         58        143 400
```

```r
addmargins(gen.tubdisp)
```

```
##
##          General waste bin Recycling bin Recycling centre
##   Male                  32            94               14
##   Female                54           117               15
##   Other                  0             0                0
##   Sum                   86           211               29
##
##          Specialist waste collection Landfill Deposit return scheme
##   Male                             4        1                     2
##   Female                           7        2                     2
##   Other                            1        0                     0
##   Sum                             12        3                     4
##
##          Indefinite storage Other I don't know Sum
##   Male                    8     2            1 158
##   Female                 33     4            5 239
##   Other                   1     1            0   3
##   Sum                    42     7            6 400
```

```r
addmargins(gen.filmpurchase)
```

```
##
##          None 1-2 3-5 6-10 11-15 16+ I don't know Sum
##   Male      5  29  46   52    12   9            5 158
##   Female    7  55  76   60    26   9            6 239
##   Other     0   0   0    1     1   0            1   3
##   Sum      12  84 122  113    39  18           12 400
```

```r
addmargins(gen.filmreuse)
```

```
##
##          Never Once 2-4 times 5-10 times More often Sum
##   Male      92   28        26          4          8 158
##   Female   143   55        27          8          6 239
##   Other      1    0         1          0          1   3
##   Sum      236   83        54         12         15 400
```

```r
addmargins(gen.filmdisp)
```

```
##
##          General waste bin Recycling bin Recycling centre
##   Male                  97            44                7
##   Female               167            48               10
##   Other                  1             0                0
##   Sum                  265            92               17
##
##          Specialist waste collection Landfill Deposit return scheme
##   Male                             4        2                     1
##   Female                           2        1                     1
##   Other                            0        0                     0
##   Sum                              6        3                     2
##
##          Indefinite storage Other I don't know Sum
##   Male                    0     2            1 158
##   Female                  3     3            4 239
##   Other                   0     1            1   3
##   Sum                     3     6            6 400
```

```r
addmargins(gen.bagpurchase)
```

```
##
##          None 1-2 3-5 6-10 11-15 16+ I don't know Sum
##   Male     94  24  17   10     7   4            2 158
##   Female  137  52  26    9     7   4            4 239
##   Other     1   1   0    0     0   0            1   3
##   Sum     232  77  43   19    14   8            7 400
```

```r
addmargins(gen.bagreuse)
```

```
##
##          Never Once 2-4 times 5-10 times More often Sum
##   Male      14   24        36         32         52 158
##   Female    17   20        56         38        108 239
##   Other      0    0         1          0          2   3
##   Sum       31   44        93         70        162 400
```

```r
addmargins(gen.bagdisp)
```

```
##
##          General waste bin Recycling bin Recycling centre
##   Male                  63            45                9
##   Female                80            47               20
##   Other                  0             0                0
##   Sum                  143            92               29
##
##          Specialist waste collection Landfill Deposit return scheme
##   Male                             2        1                     4
##   Female                           5        1                     7
```

```
##   Other                          1        0                      0
##   Sum                            8        2                     11
##
##          Indefinite storage Other I don't know Sum
##   Male                     23     9            2 158
##   Female                   59    14            6 239
##   Other                     1     0            1   3
##   Sum                      83    23            9 400
```

'Income' factor:

```
inc.choice <- table(data$annual_income, data$choice_plastic)
inc.barrier <- table(data$annual_income, data$barrier_recyling)
inc.barrier2 <- table(data$annual_income, data$barrier_reducing)
inc.mainconsid <- table(data$annual_income, data$main_con_plastic)
inc.litter <- table(data$annual_income, data$littering)
inc.attitude <- table(data$annual_income, data$attitude_disposal)
inc.ocean <- table(data$annual_income, data$plasticwaste_ocean)
inc.actions <- table(data$annual_income, data$individual_actions)
inc.attplast <- table(data$annual_income, data$attitude_plastic)
inc.zerow <- table(data$annual_income, data$zerowaste)


## SUPs
inc.bottpurchase <- table(data$annual_income, data$bottles_avgweek)
inc.bottreuse <- table(data$annual_income, data$bottles_reuses)
inc.bottdisp <- table(data$annual_income, data$bottles_disposal)
inc.tubspurchase <- table(data$annual_income, data$tubs_avgweek)
inc.tubreuse <- table(data$annual_income, data$tubs_reuses)
inc.tubdisp <- table(data$annual_income, data$tubs_disposal)
inc.filmpurchase <- table(data$annual_income, data$film_avgweek)
inc.filmreuse <- table(data$annual_income, data$film_reuses)
inc.filmdisp <- table(data$annual_income, data$film_disposal)
inc.bagpurchase <- table(data$annual_income, data$bags_avgweek)
inc.bagreuse <- table(data$annual_income, data$bags_reuses)
inc.bagdisp <- table(data$annual_income, data$bags_disposal)
```

```
addmargins(inc.choice)
```

```
##
##                       Always As often as they can If cheaper or preferred
##   Less than £12,000      4                    17                      15
##   £12,000-14,999         1                    15                      10
##   £15,000-19,999        15                    28                      14
##   £20,000-24,999         4                    25                      15
##   £25,000-34,999         4                    31                      14
##   £35,000-49,999        12                    40                      19
##   £50,000-74,999         2                    27                      12
##   £100,000-149,000       2                    11                       3
##   £150,000 or more       1                     2                       1
##   Prefer not to say      1                    12                      13
```

```
##   Sum                   46                 208                      116
##
##                        Rarely or never Sum
##   Less than £12,000                  6  42
##   £12,000-14,999                     2  28
##   £15,000-19,999                     6  63
##   £20,000-24,999                     2  46
##   £25,000-34,999                     3  52
##   £35,000-49,999                     5  76
##   £50,000-74,999                     4  45
##   £100,000-149,000                   0  16
##   £150,000 or more                   0   4
##   Prefer not to say                  2  28
##   Sum                               30 400
```

```r
addmargins(inc.barrier)
```

```
##
##                        Council collection Unclear information Difficult transport
##   Less than £12,000                     9                   7                   6
##   £12,000-14,999                        5                  10                   1
##   £15,000-19,999                       17                   9                   6
##   £20,000-24,999                       13                   4                   3
##   £25,000-34,999                       12                   3                   4
##   £35,000-49,999                       28                  13                   7
##   £50,000-74,999                       15                   7                   3
##   £100,000-149,000                      6                   3                   0
##   £150,000 or more                      1                   0                   0
##   Prefer not to say                     9                   6                   1
##   Sum                                 115                  62                  31
##
##                        No local facilities No support Ends up in landfills
##   Less than £12,000                      3          0                    3
##   £12,000-14,999                         3          0                    2
##   £15,000-19,999                         4          5                    4
##   £20,000-24,999                         6          0                    2
##   £25,000-34,999                         8          5                    6
##   £35,000-49,999                         7          0                    6
##   £50,000-74,999                         1          3                    6
##   £100,000-149,000                       1          1                    3
##   £150,000 or more                       0          2                    0
##   Prefer not to say                      2          2                    1
##   Sum                                   35         18                   33
##
##                        Forgetting Recycling a hassle Household disagrees
##   Less than £12,000             2                  2                   1
##   £12,000-14,999                2                  1                   0
##   £15,000-19,999                3                  2                   2
##   £20,000-24,999                4                  1                   1
##   £25,000-34,999                2                  0                   0
##   £35,000-49,999                6                  2                   0
##   £50,000-74,999                5                  1                   1
##   £100,000-149,000              0                  0                   0
##   £150,000 or more              0                  0                   0
```

```
##   Prefer not to say          1                 1                   0
##   Sum                       25                10                   5
##
##                     Recycling not important Other Already doing everything Sum
##   Less than £12,000                       0     0                        9  42
##   £12,000-14,999                          0     2                        2  28
##   £15,000-19,999                          1     1                        9  63
##   £20,000-24,999                          2     1                        9  46
##   £25,000-34,999                          0     0                       12  52
##   £35,000-49,999                          0     0                        7  76
##   £50,000-74,999                          0     0                        3  45
##   £100,000-149,000                        0     0                        2  16
##   £150,000 or more                        1     0                        0   4
##   Prefer not to say                       1     0                        4  28
##   Sum                                     5     4                       57 400
```

```r
addmargins(inc.barrier2)
```

```
##
##                     Limited alternatives No SUP-free alternatives
##   Less than £12,000                    7                        8
##   £12,000-14,999                       6                        4
##   £15,000-19,999                      10                       16
##   £20,000-24,999                       8                        6
##   £25,000-34,999                      13                        8
##   £35,000-49,999                      21                       19
##   £50,000-74,999                      11                       11
##   £100,000-149,000                     2                        4
##   £150,000 or more                     0                        2
##   Prefer not to say                   12                        7
##   Sum                                 90                       85
##
##                     Alternatives expensive Limited functioning
##   Less than £12,000                      9                   4
##   £12,000-14,999                         9                   5
##   £15,000-19,999                        14                   6
##   £20,000-24,999                        11                   6
##   £25,000-34,999                         7                   3
##   £35,000-49,999                        17                   7
##   £50,000-74,999                         6                   5
##   £100,000-149,000                       6                   2
##   £150,000 or more                       1                   0
##   Prefer not to say                      5                   1
##   Sum                                   85                  39
##
##                     Forgetting reusables Reducing not important Other
##   Less than £12,000                    9                      0     0
##   £12,000-14,999                       2                      1     1
##   £15,000-19,999                       4                      3     3
##   £20,000-24,999                       4                      3     0
##   £25,000-34,999                       9                      1     1
##   £35,000-49,999                       6                      2     1
##   £50,000-74,999                       5                      2     1
##   £100,000-149,000                     1                      0     0
```

```
## £150,000 or more                   0                      1     0
## Prefer not to say                  0                      0     1
## Sum                               40                     13     8
##
##                     No barriers Sum
## Less than £12,000            5  42
## £12,000-14,999               0  28
## £15,000-19,999               7  63
## £20,000-24,999               8  46
## £25,000-34,999              10  52
## £35,000-49,999               3  76
## £50,000-74,999               4  45
## £100,000-149,000             1  16
## £150,000 or more             0   4
## Prefer not to say            2  28
## Sum                         40 400
```

```r
addmargins(inc.mainconsid)
```

```
##
##                     Value for money Price Quality Deals/discounts
## Less than £12,000                12    12      11               4
## £12,000-14,999                    9     6       3               2
## £15,000-19,999                   18    11      16               5
## £20,000-24,999                   10    17      10               3
## £25,000-34,999                   18    15       9               1
## £35,000-49,999                   23    19      18               2
## £50,000-74,999                   16     4      10               8
## £100,000-149,000                  5     3       7               0
## £150,000 or more                  0     1       1               1
## Prefer not to say                 8     9       3               0
## Sum                             119    97      88              26
##
##                     Use-by-dates/longevity Convenience
## Less than £12,000                        1           1
## £12,000-14,999                           1           2
## £15,000-19,999                           1           2
## £20,000-24,999                           2           0
## £25,000-34,999                           1           0
## £35,000-49,999                           2           2
## £50,000-74,999                           2           1
## £100,000-149,000                         1           0
## £150,000 or more                         0           0
## Prefer not to say                        3           2
## Sum                                     14          10
##
##                     Ease of recycling packaging Sustainability Brand Ethics
## Less than £12,000                             1              0     0      0
## £12,000-14,999                                2              1     1      1
## £15,000-19,999                                1              4     2      1
## £20,000-24,999                                2              2     0      0
## £25,000-34,999                                6              2     0      0
## £35,000-49,999                                6              2     1      1
## £50,000-74,999                                3              1     0      0
```

```
##   £100,000-149,000                          0              0     0      0
##   £150,000 or more                          0              0     0      1
##   Prefer not to say                         1              1     0      1
##   Sum                                      22             13     4      5
##
##                     Other Sum
##   Less than £12,000     0  42
##   £12,000-14,999        0  28
##   £15,000-19,999        2  63
##   £20,000-24,999        0  46
##   £25,000-34,999        0  52
##   £35,000-49,999        0  76
##   £50,000-74,999        0  45
##   £100,000-149,000      0  16
##   £150,000 or more      0   4
##   Prefer not to say     0  28
##   Sum                   2 400
```

```
addmargins(inc.litter)
```

```
##
##                     Strongly agree Agree Neither agree nor disagree Disagree
##   Less than £12,000             18    14                          4        2
##   £12,000-14,999                11    14                          2        1
##   £15,000-19,999                26    27                          8        2
##   £20,000-24,999                17    18                         11        0
##   £25,000-34,999                26    16                          9        1
##   £35,000-49,999                33    35                          7        1
##   £50,000-74,999                20    19                          5        1
##   £100,000-149,000               6     9                          1        0
##   £150,000 or more               3     1                          0        0
##   Prefer not to say             13     9                          6        0
##   Sum                          173   162                         53        8
##
##                     Strongly disagree Sum
##   Less than £12,000                 4  42
##   £12,000-14,999                    0  28
##   £15,000-19,999                    0  63
##   £20,000-24,999                    0  46
##   £25,000-34,999                    0  52
##   £35,000-49,999                    0  76
##   £50,000-74,999                    0  45
##   £100,000-149,000                  0  16
##   £150,000 or more                  0   4
##   Prefer not to say                 0  28
##   Sum                               4 400
```

```
addmargins(inc.attitude)
```

```
##
##                     1 (Not concerned)   2   3   4   5   6 7 (Very concerned Sum
##   Less than £12,000                 4   1   8   8   8   2               11  42
##   £12,000-14,999                    2   2   2   6   5   3                8  28
```

```
##   £15,000-19,999                    6   1   7  13  15   9                12  63
##   £20,000-24,999                    4   3   4   7  18   4                 6  46
##   £25,000-34,999                    2   2   8  10  10  11                 9  52
##   £35,000-49,999                    3   3   4  19  22  14                11  76
##   £50,000-74,999                    2   5   4   6  13  10                 5  45
##   £100,000-149,000                  1   0   0   1  11   0                 3  16
##   £150,000 or more                  0   0   0   0   2   2                 0   4
##   Prefer not to say                 3   3   4   3  10   1                 4  28
##   Sum                              27  20  41  73 114  56                69 400
```

```r
addmargins(inc.ocean)
```

```
##
##                      Always Most of the time Sometimes Rarely Never Sum
##   Less than £12,000      4               10        19      5     4  42
##   £12,000-14,999         3                4        13      5     3  28
##   £15,000-19,999         4               19        30      7     3  63
##   £20,000-24,999         2               10        28      6     0  46
##   £25,000-34,999         1               16        25      9     1  52
##   £35,000-49,999         3               24        35     12     2  76
##   £50,000-74,999         1               10        23      9     2  45
##   £100,000-149,000       0                5         9      1     1  16
##   £150,000 or more       1                0         3      0     0   4
##   Prefer not to say      0                6        15      6     1  28
##   Sum                   19              104       200     60    17 400
```

```r
addmargins(inc.actions)
```

```
##
##                      Yes, definitely Yes, probably No, probably not
##   Less than £12,000               17            16                6
##   £12,000-14,999                  10            13                5
##   £15,000-19,999                  19            37                5
##   £20,000-24,999                  17            19                8
##   £25,000-34,999                  16            27                2
##   £35,000-49,999                  34            35                4
##   £50,000-74,999                  16            18                8
##   £100,000-149,000                 3            11                1
##   £150,000 or more                 1             3                0
##   Prefer not to say                9            12                4
##   Sum                            142           191               43
##
##                      No, definitely not I don’t know Sum
##   Less than £12,000                   2            1  42
##   £12,000-14,999                      0            0  28
##   £15,000-19,999                      2            0  63
##   £20,000-24,999                      1            1  46
##   £25,000-34,999                      3            4  52
##   £35,000-49,999                      2            1  76
##   £50,000-74,999                      1            2  45
##   £100,000-149,000                    0            1  16
##   £150,000 or more                    0            0   4
##   Prefer not to say                   0            3  28
##   Sum                                11           13 400
```

```r
addmargins(inc.attplast)
```

```
##
##                       Will go out of their way to avoid
##   Less than £12,000                                  11
##   £12,000-14,999                                      9
##   £15,000-19,999                                     23
##   £20,000-24,999                                      8
##   £25,000-34,999                                     14
##   £35,000-49,999                                     21
##   £50,000-74,999                                      7
##   £100,000-149,000                                    5
##   £150,000 or more                                    1
##   Prefer not to say                                   3
##   Sum                                               102
##
##                       If option is readily available, will avoid
##   Less than £12,000                                           16
##   £12,000-14,999                                              15
##   £15,000-19,999                                              29
##   £20,000-24,999                                              26
##   £25,000-34,999                                              29
##   £35,000-49,999                                              46
##   £50,000-74,999                                              30
##   £100,000-149,000                                             9
##   £150,000 or more                                             3
##   Prefer not to say                                           15
##   Sum                                                        218
##
##                       Will avoid only without extra costs Not a priority Sum
##   Less than £12,000                                    10              5  42
##   £12,000-14,999                                        3              1  28
##   £15,000-19,999                                        7              4  63
##   £20,000-24,999                                        9              3  46
##   £25,000-34,999                                        5              4  52
##   £35,000-49,999                                        6              3  76
##   £50,000-74,999                                        8              0  45
##   £100,000-149,000                                      1              1  16
##   £150,000 or more                                      0              0   4
##   Prefer not to say                                     9              1  28
##   Sum                                                  58             22 400
```

```r
addmargins(inc.zerow)
```

```
##
##                       Yes, shops regularly Yes, shops occasionally
##   Less than £12,000                      2                       0
##   £12,000-14,999                         0                       2
##   £15,000-19,999                         2                      12
##   £20,000-24,999                         2                       3
##   £25,000-34,999                         0                       3
##   £35,000-49,999                         4                       4
```

```
##   £50,000-74,999                       1                        3
##   £100,000-149,000                     2                        3
##   £150,000 or more                     0                        0
##   Prefer not to say                    1                        0
##   Sum                                 14                       30
##
##                      Yes, visited at least once Yes, never visited
##   Less than £12,000                           4                  8
##   £12,000-14,999                              1                  6
##   £15,000-19,999                              4                 16
##   £20,000-24,999                              3                  6
##   £25,000-34,999                              2                  9
##   £35,000-49,999                              2                 23
##   £50,000-74,999                              5                  8
##   £100,000-149,000                            1                  3
##   £150,000 or more                            2                  2
##   Prefer not to say                           0                  7
##   Sum                                        24                 88
##
##                      No, but would like to shop there
##   Less than £12,000                                23
##   £12,000-14,999                                   13
##   £15,000-19,999                                   20
##   £20,000-24,999                                   22
##   £25,000-34,999                                   30
##   £35,000-49,999                                   33
##   £50,000-74,999                                   18
##   £100,000-149,000                                  5
##   £150,000 or more                                  0
##   Prefer not to say                                11
##   Sum                                             175
##
##                      No, not likely to shop there Sum
##   Less than £12,000                             5  42
##   £12,000-14,999                                6  28
##   £15,000-19,999                                9  63
##   £20,000-24,999                               10  46
##   £25,000-34,999                                8  52
##   £35,000-49,999                               10  76
##   £50,000-74,999                               10  45
##   £100,000-149,000                              2  16
##   £150,000 or more                              0   4
##   Prefer not to say                             9  28
##   Sum                                          69 400
```

```
## SUPs
addmargins(inc.bottpurchase)
```

```
##
##                      None 1-2 3-5 6-10 11-15 16+ I don't know Sum
##   Less than £12,000     2  15  13   10     1   0            1  42
##   £12,000-14,999        1  15   6    3     1   1            1  28
##   £15,000-19,999        1  27  15    9     4   5            2  63
##   £20,000-24,999        2  14  15   12     1   1            1  46
```

```
##   £25,000-34,999       1  13  21   12    3   0            2  52
##   £35,000-49,999       5  21  31   11    5   3            0  76
##   £50,000-74,999       0  13  14   13    3   1            1  45
##   £100,000-149,000     0   6   8    2    0   0            0  16
##   £150,000 or more     0   1   1    0    0   2            0   4
##   Prefer not to say    0  12  10    2    1   1            2  28
##   Sum                 12 137 134   74   19  14           10 400
```

```r
addmargins(inc.bottreuse)
```

```
##
##                     Never Once 2-4 times 5-10 times More often Sum
##   Less than £12,000     8    4        13          5         12  42
##   £12,000-14,999        4    6         8          4          6  28
##   £15,000-19,999        6   10        19          7         21  63
##   £20,000-24,999       11    4        11         10         10  46
##   £25,000-34,999       11    8        16         10          7  52
##   £35,000-49,999       14   13        27         12         10  76
##   £50,000-74,999        7   12        16          7          3  45
##   £100,000-149,000      2    2         7          1          4  16
##   £150,000 or more      2    0         2          0          0   4
##   Prefer not to say     6    7        10          1          4  28
##   Sum                  71   66       129         57         77 400
```

```r
addmargins(inc.bottdisp)
```

```
##
##                     General waste bin Recycling bin Recycling centre
##   Less than £12,000                 7            32                1
##   £12,000-14,999                    3            23                1
##   £15,000-19,999                    3            43                2
##   £20,000-24,999                    4            36                1
##   £25,000-34,999                    3            46                0
##   £35,000-49,999                    3            67                4
##   £50,000-74,999                    2            40                0
##   £100,000-149,000                  1            12                0
##   £150,000 or more                  0             3                1
##   Prefer not to say                 1            26                0
##   Sum                              27           328               10
##
##                     Specialist waste collection Landfill Deposit return scheme
##   Less than £12,000                           0        0                     0
##   £12,000-14,999                              0        0                     0
##   £15,000-19,999                              2        3                     2
##   £20,000-24,999                              2        0                     1
##   £25,000-34,999                              0        1                     0
##   £35,000-49,999                              1        0                     0
##   £50,000-74,999                              0        1                     1
##   £100,000-149,000                            2        0                     0
##   £150,000 or more                            0        0                     0
##   Prefer not to say                           0        0                     0
##   Sum                                         7        5                     4
##
```

```
##                       Indefinite storage Other I don't know Sum
##   Less than £12,000                    0     1            1  42
##   £12,000-14,999                       1     0            0  28
##   £15,000-19,999                       8     0            0  63
##   £20,000-24,999                       2     0            0  46
##   £25,000-34,999                       1     1            0  52
##   £35,000-49,999                       1     0            0  76
##   £50,000-74,999                       1     0            0  45
##   £100,000-149,000                     1     0            0  16
##   £150,000 or more                     0     0            0   4
##   Prefer not to say                    1     0            0  28
##   Sum                                 16     2            1 400
```

```r
addmargins(inc.tubspurchase)
```

```
##
##                       None 1-2 3-5 6-10 11-15 16+ I don't know Sum
##   Less than £12,000      3  11  18    7     2   0            1  42
##   £12,000-14,999         1  11  10    4     2   0            0  28
##   £15,000-19,999         3  17  16   17     2   6            2  63
##   £20,000-24,999         0  15  15    8     4   2            2  46
##   £25,000-34,999         0  16  18   11     5   0            2  52
##   £35,000-49,999         3  14  30   22     4   2            1  76
##   £50,000-74,999         2   9  13   11     8   1            1  45
##   £100,000-149,000       0   4   8    1     2   1            0  16
##   £150,000 or more       0   1   2    0     0   1            0   4
##   Prefer not to say      0  10  13    4     0   0            1  28
##   Sum                   12 108 143   85    29  13           10 400
```

```r
addmargins(inc.tubreuse)
```

```
##
##                       Never Once 2-4 times 5-10 times More often Sum
##   Less than £12,000       5    3        12          6         16  42
##   £12,000-14,999          3    4         7          3         11  28
##   £15,000-19,999         10    2        11         10         30  63
##   £20,000-24,999          7    6        11          5         17  46
##   £25,000-34,999         10    3         9         13         17  52
##   £35,000-49,999          8   12        23          5         28  76
##   £50,000-74,999         10    5        12          9          9  45
##   £100,000-149,000        1    2         6          3          4  16
##   £150,000 or more        2    1         0          1          0   4
##   Prefer not to say       5    3         6          3         11  28
##   Sum                    61   41        97         58        143 400
```

```r
addmargins(inc.tubdisp)
```

```
##
##                       General waste bin Recycling bin Recycling centre
##   Less than £12,000                   7            24                2
##   £12,000-14,999                      6            14                0
##   £15,000-19,999                      8            33                3
```

```
##   £20,000-24,999                    11           25                 3
##   £25,000-34,999                    10           28                 7
##   £35,000-49,999                    17           41                 4
##   £50,000-74,999                    16           22                 5
##   £100,000-149,000                   3            8                 3
##   £150,000 or more                   1            1                 0
##   Prefer not to say                  7           15                 2
##   Sum                               86          211                29
##
##                      Specialist waste collection Landfill Deposit return scheme
##   Less than £12,000                            2        0                      0
##   £12,000-14,999                               2        0                      0
##   £15,000-19,999                               3        1                      2
##   £20,000-24,999                               0        1                      0
##   £25,000-34,999                               0        0                      0
##   £35,000-49,999                               3        1                      2
##   £50,000-74,999                               0        0                      0
##   £100,000-149,000                             1        0                      0
##   £150,000 or more                             1        0                      0
##   Prefer not to say                            0        0                      0
##   Sum                                         12        3                      4
##
##                      Indefinite storage Other I don't know Sum
##   Less than £12,000                   4     3            0  42
##   £12,000-14,999                      5     0            1  28
##   £15,000-19,999                     12     1            0  63
##   £20,000-24,999                      5     0            1  46
##   £25,000-34,999                      5     0            2  52
##   £35,000-49,999                      6     1            1  76
##   £50,000-74,999                      1     1            0  45
##   £100,000-149,000                    1     0            0  16
##   £150,000 or more                    1     0            0   4
##   Prefer not to say                   2     1            1  28
##   Sum                                42     7            6 400
```

```
addmargins(inc.filmpurchase)
```

```
##
##                      None 1-2 3-5 6-10 11-15 16+ I don't know Sum
##   Less than £12,000     2  10  14    9     6   0            1  42
##   £12,000-14,999        3   7  10    5     3   0            0  28
##   £15,000-19,999        4  13  15   16     4   7            4  63
##   £20,000-24,999        0  10  14   13     5   3            1  46
##   £25,000-34,999        0  13  16   16     4   1            2  52
##   £35,000-49,999        1  12  30   24     5   3            1  76
##   £50,000-74,999        1   9  10   14     9   1            1  45
##   £100,000-149,000      0   3   4    7     1   1            0  16
##   £150,000 or more      0   1   1    1     0   1            0   4
##   Prefer not to say     1   6   8    8     2   1            2  28
##   Sum                  12  84 122  113    39  18           12 400
```

```
addmargins(inc.filmreuse)
```

```
##
##                       Never Once 2-4 times 5-10 times More often Sum
##   Less than £12,000      23    7         8          1          3  42
##   £12,000-14,999         17    3         5          2          1  28
##   £15,000-19,999         34   12        10          2          5  63
##   £20,000-24,999         27   12         3          2          2  46
##   £25,000-34,999         40    3         6          2          1  52
##   £35,000-49,999         41   25         9          1          0  76
##   £50,000-74,999         27   10         4          2          2  45
##   £100,000-149,000        7    2         6          0          1  16
##   £150,000 or more        2    1         1          0          0   4
##   Prefer not to say      18    8         2          0          0  28
##   Sum                   236   83        54         12         15 400
```

```
addmargins(inc.filmdisp)
```

```
##
##                       General waste bin Recycling bin Recycling centre
##   Less than £12,000                  25            10                2
##   £12,000-14,999                     17             9                1
##   £15,000-19,999                     41            12                3
##   £20,000-24,999                     30            12                2
##   £25,000-34,999                     34            14                2
##   £35,000-49,999                     51            17                3
##   £50,000-74,999                     31            13                1
##   £100,000-149,000                   12             1                2
##   £150,000 or more                    2             0                0
##   Prefer not to say                  22             4                1
##   Sum                               265            92               17
##
##                       Specialist waste collection Landfill Deposit return scheme
##   Less than £12,000                             0        0                     0
##   £12,000-14,999                                1        0                     0
##   £15,000-19,999                                3        1                     1
##   £20,000-24,999                                0        0                     0
##   £25,000-34,999                                0        0                     0
##   £35,000-49,999                                1        0                     1
##   £50,000-74,999                                0        0                     0
##   £100,000-149,000                              0        1                     0
##   £150,000 or more                              1        1                     0
##   Prefer not to say                             0        0                     0
##   Sum                                           6        3                     2
##
##                       Indefinite storage Other I don't know Sum
##   Less than £12,000                    1     1            3  42
##   £12,000-14,999                       0     0            0  28
##   £15,000-19,999                       0     2            0  63
##   £20,000-24,999                       1     1            0  46
##   £25,000-34,999                       0     1            1  52
##   £35,000-49,999                       1     0            2  76
##   £50,000-74,999                       0     0            0  45
##   £100,000-149,000                     0     0            0  16
##   £150,000 or more                     0     0            0   4
##   Prefer not to say                    0     1            0  28
```

```
##   Sum                                3     6             6  400
```

```r
addmargins(inc.bagpurchase)
```

```
##
##                        None 1-2 3-5 6-10 11-15 16+ I don't know Sum
##   Less than £12,000      24  12   3    0     2   0            1  42
##   £12,000-14,999         11   9   5    2     1   0            0  28
##   £15,000-19,999         33  11   2    6     5   4            2  63
##   £20,000-24,999         23  10   7    2     1   2            1  46
##   £25,000-34,999         33  13   5    1     0   0            0  52
##   £35,000-49,999         51   9   8    3     2   1            2  76
##   £50,000-74,999         23   9   7    3     2   1            0  45
##   £100,000-149,000       10   1   4    0     1   0            0  16
##   £150,000 or more        1   1   1    1     0   0            0   4
##   Prefer not to say      23   2   1    1     0   0            1  28
##   Sum                   232  77  43   19    14   8            7 400
```

```r
addmargins(inc.bagreuse)
```

```
##
##                        Never Once 2-4 times 5-10 times More often Sum
##   Less than £12,000        4    4         6          5         23  42
##   £12,000-14,999           1    3         7          6         11  28
##   £15,000-19,999           4    9        14         13         23  63
##   £20,000-24,999           3    2        13          7         21  46
##   £25,000-34,999           5    5        12          7         23  52
##   £35,000-49,999           6   11        17         14         28  76
##   £50,000-74,999           2    7         9         14         13  45
##   £100,000-149,000         1    2         6          1          6  16
##   £150,000 or more         1    0         3          0          0   4
##   Prefer not to say        4    1         6          3         14  28
##   Sum                     31   44        93         70        162 400
```

```r
addmargins(inc.bagdisp)
```

```
##
##                        General waste bin Recycling bin Recycling centre
##   Less than £12,000                   13            11                1
##   £12,000-14,999                      10             8                0
##   £15,000-19,999                      17            12                5
##   £20,000-24,999                      16            15                3
##   £25,000-34,999                      20             6                5
##   £35,000-49,999                      34            17                6
##   £50,000-74,999                      18            10                4
##   £100,000-149,000                     3             7                1
##   £150,000 or more                     1             0                2
##   Prefer not to say                   11             6                2
##   Sum                                143            92               29
##
##                        Specialist waste collection Landfill Deposit return scheme
##   Less than £12,000                              0        0                     2
```

```
##   £12,000-14,999                            1        0                      1
##   £15,000-19,999                            2        1                      5
##   £20,000-24,999                            0        0                      0
##   £25,000-34,999                            1        0                      1
##   £35,000-49,999                            2        0                      0
##   £50,000-74,999                            2        0                      2
##   £100,000-149,000                          0        1                      0
##   £150,000 or more                          0        0                      0
##   Prefer not to say                         0        0                      0
##   Sum                                       8        2                     11
##
##                       Indefinite storage Other I don't know Sum
##   Less than £12,000                    9     4            2  42
##   £12,000-14,999                       8     0            0  28
##   £15,000-19,999                      13     6            2  63
##   £20,000-24,999                      10     1            1  46
##   £25,000-34,999                      12     5            2  52
##   £35,000-49,999                      12     4            1  76
##   £50,000-74,999                       7     2            0  45
##   £100,000-149,000                     3     1            0  16
##   £150,000 or more                     1     0            0   4
##   Prefer not to say                    8     0            1  28
##   Sum                                 83    23            9 400
```

'Vehicle ownership' factor:

```
veh.choice <- table(data$vehicle_ownership, data$choice_plastic)
veh.barrier <- table(data$vehicle_ownership, data$barrier_recyling)
veh.barrier2 <- table(data$vehicle_ownership, data$barrier_reducing)
veh.mainconsid <- table(data$vehicle_ownership, data$main_con_plastic)
veh.litter <- table(data$vehicle_ownership, data$littering)
veh.attitude <- table(data$vehicle_ownership, data$attitude_disposal)
veh.ocean <- table(data$vehicle_ownership, data$plasticwaste_ocean)
veh.actions <- table(data$vehicle_ownership, data$individual_actions)
veh.attplast <- table(data$vehicle_ownership, data$attitude_plastic)
veh.zerow <- table(data$vehicle_ownership, data$zerowaste)


## SUPs
veh.bottpurchase <- table(data$vehicle_ownership, data$bottles_avgweek)
veh.bottreuse <- table(data$vehicle_ownership, data$bottles_reuses)
veh.bottdisp <- table(data$vehicle_ownership, data$bottles_disposal)
veh.tubspurchase <- table(data$vehicle_ownership, data$tubs_avgweek)
veh.tubreuse <- table(data$vehicle_ownership, data$tubs_reuses)
veh.tubdisp <- table(data$vehicle_ownership, data$tubs_disposal)
veh.filmpurchase <- table(data$vehicle_ownership, data$film_avgweek)
veh.filmreuse <- table(data$vehicle_ownership, data$film_reuses)
veh.filmdisp <- table(data$vehicle_ownership, data$film_disposal)
veh.bagpurchase <- table(data$vehicle_ownership, data$bags_avgweek)
veh.bagreuse <- table(data$vehicle_ownership, data$bags_reuses)
veh.bagdisp <- table(data$vehicle_ownership, data$bags_disposal)
```

```r
addmargins(veh.choice)
```

```
##
##                      Always As often as they can If cheaper or preferred
##   No vehicle             10                   56                      44
##   1 or more vehicles     36                  152                      72
##   Sum                    46                  208                     116
##
##                      Rarely or never Sum
##   No vehicle                       8 118
##   1 or more vehicles              22 282
##   Sum                             30 400
```

```r
addmargins(veh.barrier)
```

```
##
##                      Council collection Unclear information Difficult transport
##   No vehicle                         29                  18                  18
##   1 or more vehicles                 86                  44                  13
##   Sum                               115                  62                  31
##
##                      No local facilities No support Ends up in landfills
##   No vehicle                          10          3                    9
##   1 or more vehicles                  25         15                   24
##   Sum                                 35         18                   33
##
##                      Forgetting Recycling a hassle Household disagrees
##   No vehicle                  7                  3                   2
##   1 or more vehicles         18                  7                   3
##   Sum                        25                 10                   5
##
##                      Recycling not important Other Already doing everything Sum
##   No vehicle                               0     3                       16 118
##   1 or more vehicles                       5     1                       41 282
##   Sum                                      5     4                       57 400
```

```r
addmargins(veh.barrier2)
```

```
##
##                      Limited alternatives No SUP-free alternatives
##   No vehicle                           30                       26
##   1 or more vehicles                   60                       59
##   Sum                                  90                       85
##
##                      Alternatives expensive Limited functioning
##   No vehicle                             27                   9
##   1 or more vehicles                     58                  30
##   Sum                                    85                  39
##
##                      Forgetting reusables Reducing not important Other
##   No vehicle                           19                      1     2
```

```
##   1 or more vehicles                     21                    12     6
##   Sum                                    40                    13     8
##
##                       No barriers Sum
##   No vehicle                    4 118
##   1 or more vehicles           36 282
##   Sum                          40 400
```

```r
addmargins(veh.mainconsid)
```

```
##
##                       Value for money Price Quality Deals/discounts
##   No vehicle                       40    34      23               4
##   1 or more vehicles               79    63      65              22
##   Sum                             119    97      88              26
##
##                       Use-by-dates/longevity Convenience
##   No vehicle                               3           5
##   1 or more vehicles                      11           5
##   Sum                                     14          10
##
##                       Ease of recycling packaging Sustainability Brand Ethics
##   No vehicle                                    4              4     0      1
##   1 or more vehicles                           18              9     4      4
##   Sum                                          22             13     4      5
##
##                       Other Sum
##   No vehicle              0 118
##   1 or more vehicles      2 282
##   Sum                     2 400
```

```r
addmargins(veh.litter)
```

```
##
##                       Strongly agree Agree Neither agree nor disagree Disagree
##   No vehicle                      53    52                          9        1
##   1 or more vehicles             120   110                         44        7
##   Sum                            173   162                         53        8
##
##                       Strongly disagree Sum
##   No vehicle                          3 118
##   1 or more vehicles                  1 282
##   Sum                                 4 400
```

```r
addmargins(veh.attitude)
```

```
##
##                       1 (Not concerned)   2   3   4   5   6 7 (Very concerned
##   No vehicle                         11   6  10  22  31  16               22
##   1 or more vehicles                 16  14  31  51  83  40               47
##   Sum                                27  20  41  73 114  56               69
##
```

```
##                        Sum
##   No vehicle           118
##   1 or more vehicles   282
##   Sum                  400
```

```r
addmargins(veh.ocean)
```

```
##
##                        Always Most of the time Sometimes Rarely Never Sum
##   No vehicle                3               31        62     18     4 118
##   1 or more vehicles       16               73       138     42    13 282
##   Sum                      19              104       200     60    17 400
```

```r
addmargins(veh.actions)
```

```
##
##                        Yes, definitely Yes, probably No, probably not
##   No vehicle                        39            63               12
##   1 or more vehicles               103           128               31
##   Sum                              142           191               43
##
##                        No, definitely not I don’t know Sum
##   No vehicle                            2            2 118
##   1 or more vehicles                    9           11 282
##   Sum                                  11           13 400
```

```r
addmargins(veh.attplast)
```

```
##
##                        Will go out of their way to avoid
##   No vehicle                                          30
##   1 or more vehicles                                  72
##   Sum                                                102
##
##                        If option is readily available, will avoid
##   No vehicle                                                   65
##   1 or more vehicles                                          153
##   Sum                                                         218
##
##                        Will avoid only without extra costs Not a priority Sum
##   No vehicle                                            20              3 118
##   1 or more vehicles                                    38             19 282
##   Sum                                                   58             22 400
```

```r
addmargins(veh.zerow)
```

```
##
##                        Yes, shops regularly Yes, shops occasionally
##   No vehicle                              2                       8
##   1 or more vehicles                     12                      22
##   Sum                                    14                      30
```

```
##
##                         Yes, visited at least once Yes, never visited
##   No vehicle                                     4                 28
##   1 or more vehicles                            20                 60
##   Sum                                           24                 88
##
##                         No, but would like to shop there
##   No vehicle                                          54
##   1 or more vehicles                                 121
##   Sum                                                175
##
##                         No, not likely to shop there Sum
##   No vehicle                                        22 118
##   1 or more vehicles                                47 282
##   Sum                                               69 400
```

```r
## SUPs
addmargins(veh.bottpurchase)
```

```
##
##                         None 1-2 3-5 6-10 11-15 16+ I don't know Sum
##   No vehicle               6  45  40   16     9   0            2 118
##   1 or more vehicles       6  92  94   58    10  14            8 282
##   Sum                     12 137 134   74    19  14           10 400
```

```r
addmargins(veh.bottreuse)
```

```
##
##                         Never Once 2-4 times 5-10 times More often Sum
##   No vehicle               17   17        39         17         28 118
##   1 or more vehicles       54   49        90         40         49 282
##   Sum                      71   66       129         57         77 400
```

```r
addmargins(veh.bottdisp)
```

```
##
##                         General waste bin Recycling bin Recycling centre
##   No vehicle                           12           100                0
##   1 or more vehicles                   15           228               10
##   Sum                                  27           328               10
##
##                         Specialist waste collection Landfill Deposit return scheme
##   No vehicle                                      0        0                     1
##   1 or more vehicles                              7        5                     3
##   Sum                                             7        5                     4
##
##                         Indefinite storage Other I don't know Sum
##   No vehicle                             3     2            0 118
##   1 or more vehicles                    13     0            1 282
##   Sum                                   16     2            1 400
```

```r
addmargins(veh.tubspurchase)
```

```
##
##                       None 1-2 3-5 6-10 11-15 16+ I don't know Sum
##   No vehicle             6  35  42   22     8   2            3 118
##   1 or more vehicles     6  73 101   63    21  11            7 282
##   Sum                   12 108 143   85    29  13           10 400
```

```r
addmargins(veh.tubreuse)
```

```
##
##                       Never Once 2-4 times 5-10 times More often Sum
##   No vehicle             17    8        31         14         48 118
##   1 or more vehicles     44   33        66         44         95 282
##   Sum                    61   41        97         58        143 400
```

```r
addmargins(veh.tubdisp)
```

```
##
##                       General waste bin Recycling bin Recycling centre
##   No vehicle                         25            72                6
##   1 or more vehicles                 61           139               23
##   Sum                                86           211               29
##
##                       Specialist waste collection Landfill Deposit return scheme
##   No vehicle                                    1        0                     0
##   1 or more vehicles                           11        3                     4
##   Sum                                          12        3                     4
##
##                       Indefinite storage Other I don't know Sum
##   No vehicle                           9     2            3 118
##   1 or more vehicles                  33     5            3 282
##   Sum                                 42     7            6 400
```

```r
addmargins(veh.filmpurchase)
```

```
##
##                       None 1-2 3-5 6-10 11-15 16+ I don't know Sum
##   No vehicle             7  24  33   32    15   3            4 118
##   1 or more vehicles     5  60  89   81    24  15            8 282
##   Sum                   12  84 122  113    39  18           12 400
```

```r
addmargins(veh.filmreuse)
```

```
##
##                       Never Once 2-4 times 5-10 times More often Sum
##   No vehicle             70   26        14          3          5 118
##   1 or more vehicles    166   57        40          9         10 282
##   Sum                   236   83        54         12         15 400
```

```r
addmargins(veh.filmdisp)
```

```
##
##                         General waste bin Recycling bin Recycling centre
##    No vehicle                          74            32                5
##    1 or more vehicles                 191            60               12
##    Sum                                265            92               17
##
##                         Specialist waste collection Landfill Deposit return scheme
##    No vehicle                                     1        0                     0
##    1 or more vehicles                             5        3                     2
##    Sum                                            6        3                     2
##
##                         Indefinite storage Other I don't know Sum
##    No vehicle                            1     3            2 118
##    1 or more vehicles                    2     3            4 282
##    Sum                                   3     6            6 400
```

```r
addmargins(veh.bagpurchase)
```

```
##
##                         None 1-2 3-5 6-10 11-15 16+ I don't know Sum
##    No vehicle             64  25  15    6     6   0            2 118
##    1 or more vehicles    168  52  28   13     8   8            5 282
##    Sum                   232  77  43   19    14   8            7 400
```

```r
addmargins(veh.bagreuse)
```

```
##
##                         Never Once 2-4 times 5-10 times More often Sum
##    No vehicle               8   10        29         22         49 118
##    1 or more vehicles      23   34        64         48        113 282
##    Sum                     31   44        93         70        162 400
```

```r
addmargins(veh.bagdisp)
```

```
##
##                         General waste bin Recycling bin Recycling centre
##    No vehicle                          32            36                9
##    1 or more vehicles                 111            56               20
##    Sum                                143            92               29
##
##                         Specialist waste collection Landfill Deposit return scheme
##    No vehicle                                     3        0                     2
##    1 or more vehicles                             5        2                     9
##    Sum                                            8        2                    11
##
##                         Indefinite storage Other I don't know Sum
##    No vehicle                           25     6            5 118
##    1 or more vehicles                   58    17            4 282
##    Sum                                  83    23            9 400
```

'Living situation' factor:

```r
liv.choice <- table(data$living_situation, data$choice_plastic)
liv.barrier <- table(data$living_situation, data$barrier_recyling)
liv.barrier2 <- table(data$living_situation, data$barrier_reducing)
liv.mainconsid <- table(data$living_situation, data$main_con_plastic)
liv.litter <- table(data$living_situation, data$littering)
liv.attitude <- table(data$living_situation, data$attitude_disposal)
liv.ocean <- table(data$living_situation, data$plasticwaste_ocean)
liv.actions <- table(data$living_situation, data$individual_actions)
liv.attplast <- table(data$living_situation, data$attitude_plastic)
liv.zerow <- table(data$living_situation, data$zerowaste)


## SUPs
liv.bottpurchase <- table(data$living_situation, data$bottles_avgweek)
liv.bottreuse <- table(data$living_situation, data$bottles_reuses)
liv.bottdisp <- table(data$living_situation, data$bottles_disposal)
liv.tubspurchase <- table(data$living_situation, data$tubs_avgweek)
liv.tubreuse <- table(data$living_situation, data$tubs_reuses)
liv.tubdisp <- table(data$living_situation, data$tubs_disposal)
liv.filmpurchase <- table(data$living_situation, data$film_avgweek)
liv.filmreuse <- table(data$living_situation, data$film_reuses)
liv.filmdisp <- table(data$living_situation, data$film_disposal)
liv.bagpurchase <- table(data$living_situation, data$bags_avgweek)
liv.bagreuse <- table(data$living_situation, data$bags_reuses)
liv.bagdisp <- table(data$living_situation, data$bags_disposal)
```

```r
addmargins(liv.choice)
```

```
##
##                                   Always As often as they can
##    I live alone                       10                   44
##    I live with my parents/family       7                   22
##    I live in a house-/ flat-share      7                   21
##    I live with my partner/children    22                  119
##    Other                               0                    2
##    Sum                                46                  208
##
##                                   If cheaper or preferred Rarely or never Sum
##    I live alone                                        28              10  92
##    I live with my parents/family                       21               1  51
##    I live in a house-/ flat-share                       9               3  40
##    I live with my partner/children                     57              15 213
##    Other                                                1               1   4
##    Sum                                                116              30 400
```

```r
addmargins(liv.barrier)
```

```
##
##                                   Council collection Unclear information
```

```
##   I live alone                                    26                10
##   I live with my parents/family                   10                12
##   I live in a house-/ flat-share                  11                 7
##   I live with my partner/children                 67                32
##   Other                                            1                 1
##   Sum                                            115                62
##
##                                   Difficult transport No local facilities
##   I live alone                                     10                   7
##   I live with my parents/family                     5                   4
##   I live in a house-/ flat-share                    2                   5
##   I live with my partner/children                  14                  18
##   Other                                             0                   1
##   Sum                                              31                  35
##
##                                   No support Ends up in landfills Forgetting
##   I live alone                             5                    2          7
##   I live with my parents/family            4                    3          5
##   I live in a house-/ flat-share           1                    5          2
##   I live with my partner/children          8                   23         10
##   Other                                    0                    0          1
##   Sum                                     18                   33         25
##
##                                   Recycling a hassle Household disagrees
##   I live alone                                     4                   1
##   I live with my parents/family                    3                   1
##   I live in a house-/ flat-share                   1                   1
##   I live with my partner/children                  2                   2
##   Other                                            0                   0
##   Sum                                             10                   5
##
##                                   Recycling not important Other
##   I live alone                                          2     0
##   I live with my parents/family                         1     0
##   I live in a house-/ flat-share                        0     1
##   I live with my partner/children                       2     3
##   Other                                                 0     0
##   Sum                                                   5     4
##
##                                   Already doing everything Sum
##   I live alone                                          18  92
##   I live with my parents/family                          3  51
##   I live in a house-/ flat-share                         4  40
##   I live with my partner/children                       32 213
##   Other                                                  0   4
##   Sum                                                   57 400
```

```
addmargins(liv.barrier2)
```

```
##
##                                   Limited alternatives No SUP-free alternatives
##   I live alone                                      28                       15
##   I live with my parents/family                      7                       11
##   I live in a house-/ flat-share                     6                       11
```

```
##   I live with my partner/children                      49                        47
##   Other                                                 0                         1
##   Sum                                                  90                        85
##
##                                    Alternatives expensive Limited functioning
##   I live alone                                         15                   9
##   I live with my parents/family                        17                   5
##   I live in a house-/ flat-share                        7                   5
##   I live with my partner/children                      45                  20
##   Other                                                 1                   0
##   Sum                                                  85                  39
##
##                                    Forgetting reusables Reducing not important
##   I live alone                                       10                      1
##   I live with my parents/family                       6                      4
##   I live in a house-/ flat-share                      5                      3
##   I live with my partner/children                    19                      4
##   Other                                               0                      1
##   Sum                                                40                     13
##
##                                    Other No barriers Sum
##   I live alone                         1          13  92
##   I live with my parents/family        0           1  51
##   I live in a house-/ flat-share       1           2  40
##   I live with my partner/children      6          23 213
##   Other                                0           1   4
##   Sum                                  8          40 400
```

```
addmargins(liv.mainconsid)
```

```
##
##                                    Value for money Price Quality Deals/discounts
##   I live alone                                  26    16      27               5
##   I live with my parents/family                 15    18       6               3
##   I live in a house-/ flat-share                17     9       4               4
##   I live with my partner/children               58    53      51              14
##   Other                                          3     1       0               0
##   Sum                                          119    97      88              26
##
##                                    Use-by-dates/longevity Convenience
##   I live alone                                         3           4
##   I live with my parents/family                        0           1
##   I live in a house-/ flat-share                       2           0
##   I live with my partner/children                      9           5
##   Other                                                0           0
##   Sum                                                 14          10
##
##                                    Ease of recycling packaging Sustainability
##   I live alone                                               5              2
##   I live with my parents/family                              2              3
##   I live in a house-/ flat-share                             3              1
##   I live with my partner/children                           12              7
##   Other                                                      0              0
##   Sum                                                       22             13
```

```
##
##                                    Brand Ethics Other Sum
##   I live alone                          0      2     2  92
##   I live with my parents/family         2      1     0  51
##   I live in a house-/ flat-share        0      0     0  40
##   I live with my partner/children       2      2     0 213
##   Other                                 0      0     0   4
##   Sum                                   4      5     2 400
```

```
addmargins(liv.litter)
```

```
##
##                                    Strongly agree Agree
##   I live alone                                 31    43
##   I live with my parents/family                23    18
##   I live in a house-/ flat-share               15    14
##   I live with my partner/children             104    85
##   Other                                         0     2
##   Sum                                         173   162
##
##                                    Neither agree nor disagree Disagree
##   I live alone                                             14        3
##   I live with my parents/family                             8        1
##   I live in a house-/ flat-share                            7        3
##   I live with my partner/children                          22        1
##   Other                                                     2        0
##   Sum                                                      53        8
##
##                                    Strongly disagree Sum
##   I live alone                                     1  92
##   I live with my parents/family                    1  51
##   I live in a house-/ flat-share                   1  40
##   I live with my partner/children                  1 213
##   Other                                            0   4
##   Sum                                              4 400
```

```
addmargins(liv.attitude)
```

```
##
##                                    1 (Not concerned)   2   3   4   5   6
##   I live alone                                     6   4   9  16  25  12
##   I live with my parents/family                    8   2   5  14  13   6
##   I live in a house-/ flat-share                   5   2   7   6   6   4
##   I live with my partner/children                  7  12  19  36  69  34
##   Other                                            1   0   1   1   1   0
##   Sum                                             27  20  41  73 114  56
##
##                                    7 (Very concerned Sum
##   I live alone                                    20  92
##   I live with my parents/family                    3  51
##   I live in a house-/ flat-share                  10  40
##   I live with my partner/children                 36 213
##   Other                                            0   4
##   Sum                                             69 400
```

```r
addmargins(liv.ocean)
```

```
##
##                                    Always Most of the time Sometimes Rarely
##    I live alone                          2               18        50     19
##    I live with my parents/family         5               15        26      5
##    I live in a house-/ flat-share        2               14        20      0
##    I live with my partner/children      10               56       103     35
##    Other                                 0                1         1      1
##    Sum                                  19              104       200     60
##
##                                    Never Sum
##    I live alone                        3  92
##    I live with my parents/family       0  51
##    I live in a house-/ flat-share      4  40
##    I live with my partner/children     9 213
##    Other                               1   4
##    Sum                                17 400
```

```r
addmargins(liv.actions)
```

```
##
##                                    Yes, definitely Yes, probably
##    I live alone                                 39            38
##    I live with my parents/family                13            27
##    I live in a house-/ flat-share               13            20
##    I live with my partner/children              77           102
##    Other                                         0             4
##    Sum                                         142           191
##
##                                    No, probably not No, definitely not
##    I live alone                                  12                  1
##    I live with my parents/family                  6                  1
##    I live in a house-/ flat-share                 5                  1
##    I live with my partner/children               20                  8
##    Other                                          0                  0
##    Sum                                           43                 11
##
##                                    I don't know Sum
##    I live alone                               2  92
##    I live with my parents/family              4  51
##    I live in a house-/ flat-share             1  40
##    I live with my partner/children            6 213
##    Other                                      0   4
##    Sum                                       13 400
```

```r
addmargins(liv.attplast)
```

```
##
##                                    Will go out of their way to avoid
##    I live alone                                                   31
```

```
##   I live with my parents/family                                  14
##   I live in a house-/ flat-share                                 12
##   I live with my partner/children                                45
##   Other                                                           0
##   Sum                                                           102
##
##                                     If option is readily available, will avoid
##   I live alone                                                                39
##   I live with my parents/family                                               29
##   I live in a house-/ flat-share                                              20
##   I live with my partner/children                                            127
##   Other                                                                        3
##   Sum                                                                        218
##
##                                     Will avoid only without extra costs
##   I live alone                                                      15
##   I live with my parents/family                                      5
##   I live in a house-/ flat-share                                     7
##   I live with my partner/children                                   31
##   Other                                                              0
##   Sum                                                               58
##
##                                     Not a priority Sum
##   I live alone                                   7  92
##   I live with my parents/family                  3  51
##   I live in a house-/ flat-share                 1  40
##   I live with my partner/children               10 213
##   Other                                          1   4
##   Sum                                           22 400
```

```
addmargins(liv.zerow)
```

```
##
##                                     Yes, shops regularly Yes, shops occasionally
##   I live alone                                         5                       7
##   I live with my parents/family                        2                       4
##   I live in a house-/ flat-share                       1                       2
##   I live with my partner/children                      6                      17
##   Other                                                0                       0
##   Sum                                                 14                      30
##
##                                     Yes, visited at least once Yes, never visited
##   I live alone                                               7                 15
##   I live with my parents/family                              2                 13
##   I live in a house-/ flat-share                             4                 16
##   I live with my partner/children                           11                 44
##   Other                                                      0                  0
##   Sum                                                       24                 88
##
##                                     No, but would like to shop there
##   I live alone                                                    39
##   I live with my parents/family                                   23
##   I live in a house-/ flat-share                                  15
##   I live with my partner/children                                 96
```

```
##   Other                                                  2
##   Sum                                                  175
##
##                                No, not likely to shop there Sum
##   I live alone                                           19  92
##   I live with my parents/family                           7  51
##   I live in a house-/ flat-share                          2  40
##   I live with my partner/children                        39 213
##   Other                                                   2   4
##   Sum                                                    69 400
```

```
## SUPs
addmargins(liv.bottpurchase)
```

```
##
##                                None 1-2 3-5 6-10 11-15 16+ I don't know Sum
##   I live alone                     5  43  27   11     1   3            2  92
##   I live with my parents/family    1  15  15    9     4   5            2  51
##   I live in a house-/ flat-share   0  14  16    6     2   1            1  40
##   I live with my partner/children  6  65  74   46    12   5            5 213
##   Other                            0   0   2    2     0   0            0   4
##   Sum                             12 137 134   74    19  14           10 400
```

```
addmargins(liv.bottreuse)
```

```
##
##                                Never Once 2-4 times 5-10 times More often
##   I live alone                     17   13        27         11         24
##   I live with my parents/family     8    9        19          4         11
##   I live in a house-/ flat-share    4    6        11         11          8
##   I live with my partner/children  40   38        71         30         34
##   Other                             2    0         1          1          0
##   Sum                              71   66       129         57         77
##
##                                Sum
##   I live alone                     92
##   I live with my parents/family    51
##   I live in a house-/ flat-share   40
##   I live with my partner/children 213
##   Other                             4
##   Sum                             400
```

```
addmargins(liv.bottdisp)
```

```
##
##                                General waste bin Recycling bin
##   I live alone                                  7            75
##   I live with my parents/family                 9            32
##   I live in a house-/ flat-share                3            32
##   I live with my partner/children               7           186
##   Other                                         1             3
##   Sum                                          27           328
```

```
##
##                                     Recycling centre Specialist waste collection
##   I live alone                                     3                           0
##   I live with my parents/family                    2                           2
##   I live in a house-/ flat-share                   1                           1
##   I live with my partner/children                  4                           4
##   Other                                            0                           0
##   Sum                                             10                           7
##
##                                     Landfill Deposit return scheme
##   I live alone                              1                      1
##   I live with my parents/family             1                      1
##   I live in a house-/ flat-share            0                      0
##   I live with my partner/children           3                      2
##   Other                                     0                      0
##   Sum                                       5                      4
##
##                                     Indefinite storage Other I don't know Sum
##   I live alone                                       4     1            0  92
##   I live with my parents/family                      4     0            0  51
##   I live in a house-/ flat-share                     1     1            1  40
##   I live with my partner/children                    7     0            0 213
##   Other                                              0     0            0   4
##   Sum                                               16     2            1 400
```

```r
addmargins(liv.tubspurchase)
```

```
##
##                                     None 1-2 3-5 6-10 11-15 16+ I don't know Sum
##   I live alone                         4  33  35   12     2   3            3  92
##   I live with my parents/family        1   8  22   11     5   2            2  51
##   I live in a house-/ flat-share       0  15   9    9     5   1            1  40
##   I live with my partner/children      7  51  77   52    15   7            4 213
##   Other                                0   1   0    1     2   0            0   4
##   Sum                                 12 108 143   85    29  13           10 400
```

```r
addmargins(liv.tubreuse)
```

```
##
##                                     Never Once 2-4 times 5-10 times More often
##   I live alone                         19    9        17         10         37
##   I live with my parents/family         6    5        10          8         22
##   I live in a house-/ flat-share        4    3        12          7         14
##   I live with my partner/children      30   24        57         32         70
##   Other                                 2    0         1          1          0
##   Sum                                  61   41        97         58        143
##
##                                     Sum
##   I live alone                       92
##   I live with my parents/family      51
##   I live in a house-/ flat-share     40
##   I live with my partner/children 213
##   Other                               4
##   Sum                               400
```

```r
addmargins(liv.tubdisp)
```

```
##
##                                    General waste bin Recycling bin
##   I live alone                                    19            46
##   I live with my parents/family                   10            21
##   I live in a house-/ flat-share                   8            21
##   I live with my partner/children                 47           121
##   Other                                            2             2
##   Sum                                             86           211
##
##                                    Recycling centre Specialist waste collection
##   I live alone                                    5                           3
##   I live with my parents/family                   2                           2
##   I live in a house-/ flat-share                  2                           1
##   I live with my partner/children                20                           6
##   Other                                           0                           0
##   Sum                                            29                          12
##
##                                    Landfill Deposit return scheme
##   I live alone                            0                      0
##   I live with my parents/family           2                      2
##   I live in a house-/ flat-share          1                      0
##   I live with my partner/children         0                      2
##   Other                                   0                      0
##   Sum                                     3                      4
##
##                                    Indefinite storage Other I don't know Sum
##   I live alone                                     15     2            2  92
##   I live with my parents/family                     8     1            3  51
##   I live in a house-/ flat-share                    5     1            1  40
##   I live with my partner/children                  14     3            0 213
##   Other                                             0     0            0   4
##   Sum                                              42     7            6 400
```

```r
addmargins(liv.filmpurchase)
```

```
##
##                                    None 1-2 3-5 6-10 11-15 16+ I don't know Sum
##   I live alone                        8  21  34   21     3   3            2  92
##   I live with my parents/family       2   8  13   17     2   5            4  51
##   I live in a house-/ flat-share      0   8   8   13     9   1            1  40
##   I live with my partner/children     2  47  67   60    24   8            5 213
##   Other                               0   0   0    2     1   1            0   4
##   Sum                                12  84 122  113    39  18           12 400
```

```r
addmargins(liv.filmreuse)
```

```
##
##                                    Never Once 2-4 times 5-10 times More often
##   I live alone                        57   16        13          2          4
```

```
##    I live with my parents/family      28    9         9           3           2
##    I live in a house-/ flat-share     15   11         6           4           4
##    I live with my partner/children   133   46        26           3           5
##    Other                               3    1         0           0           0
##    Sum                               236   83        54          12          15
##
##                                    Sum
##    I live alone                     92
##    I live with my parents/family    51
##    I live in a house-/ flat-share   40
##    I live with my partner/children 213
##    Other                             4
##    Sum                             400
```

```r
addmargins(liv.filmdisp)
```

```
##
##                                    General waste bin Recycling bin
##    I live alone                                   61            22
##    I live with my parents/family                  32            11
##    I live in a house-/ flat-share                 21            13
##    I live with my partner/children               148            45
##    Other                                           3             1
##    Sum                                           265            92
##
##                                    Recycling centre Specialist waste collection
##    I live alone                                   4                           1
##    I live with my parents/family                  3                           0
##    I live in a house-/ flat-share                 3                           0
##    I live with my partner/children                7                           5
##    Other                                          0                           0
##    Sum                                           17                           6
##
##                                    Landfill Deposit return scheme
##    I live alone                           0                     0
##    I live with my parents/family          1                     0
##    I live in a house-/ flat-share         0                     0
##    I live with my partner/children        2                     2
##    Other                                  0                     0
##    Sum                                    3                     2
##
##                                    Indefinite storage Other I don't know Sum
##    I live alone                                     1     2            1  92
##    I live with my parents/family                    2     1            1  51
##    I live in a house-/ flat-share                   0     1            2  40
##    I live with my partner/children                  0     2            2 213
##    Other                                            0     0            0   4
##    Sum                                              3     6            6 400
```

```r
addmargins(liv.bagpurchase)
```

```
##
##                                    None 1-2 3-5 6-10 11-15 16+ I don't know Sum
```

```
##   I live alone                      61  17   3    4     2   3             2  92
##   I live with my parents/family     22   8  11    3     4   2             1  51
##   I live in a house-/ flat-share    12  12  11    2     2   0             1  40
##   I live with my partner/children  135  38  18   10     6   3             3 213
##   Other                              2   2   0    0     0   0             0   4
##   Sum                              232  77  43   19    14   8             7 400
```

```
addmargins(liv.bagreuse)
```

```
##
##                                   Never Once 2-4 times 5-10 times More often
##   I live alone                        7    9        15         13         48
##   I live with my parents/family       2    7        17          4         21
##   I live in a house-/ flat-share      0    4        10         16         10
##   I live with my partner/children    22   24        51         36         80
##   Other                               0    0         0          1          3
##   Sum                                31   44        93         70        162
##
##                                   Sum
##   I live alone                     92
##   I live with my parents/family    51
##   I live in a house-/ flat-share   40
##   I live with my partner/children 213
##   Other                             4
##   Sum                             400
```

```
addmargins(liv.bagdisp)
```

```
##
##                                   General waste bin Recycling bin
##   I live alone                                   31            26
##   I live with my parents/family                  14             9
##   I live in a house-/ flat-share                 13             8
##   I live with my partner/children                84            48
##   Other                                           1             1
##   Sum                                           143            92
##
##                                   Recycling centre Specialist waste collection
##   I live alone                                   4                           0
##   I live with my parents/family                  6                           4
##   I live in a house-/ flat-share                 3                           0
##   I live with my partner/children               16                           4
##   Other                                          0                           0
##   Sum                                           29                           8
##
##                                   Landfill Deposit return scheme
##   I live alone                           0                     0
##   I live with my parents/family          0                     2
##   I live in a house-/ flat-share         0                     1
##   I live with my partner/children        2                     8
##   Other                                  0                     0
##   Sum                                    2                    11
##
```

```
##                                         Indefinite storage Other I don't know Sum
##   I live alone                                          23     6            2  92
##   I live with my parents/family                         13     1            2  51
##   I live in a house-/ flat-share                         6     7            2  40
##   I live with my partner/children                       39     9            3 213
##   Other                                                  2     0            0   4
##   Sum                                                   83    23            9 400
```

'Postcode' factor:

```
post.choice <- table(data$postcode, data$choice_plastic)
post.barrier <- table(data$postcode, data$barrier_recyling)
post.barrier2 <- table(data$postcode, data$barrier_reducing)
post.mainconsid <- table(data$postcode, data$main_con_plastic)
post.litter <- table(data$postcode, data$littering)
post.attitude <- table(data$postcode, data$attitude_disposal)
post.ocean <- table(data$postcode, data$plasticwaste_ocean)
post.actions <- table(data$postcode, data$individual_actions)
post.attplast <- table(data$postcode, data$attitude_plastic)
post.zerow <- table(data$postcode, data$zerowaste)


## SUPs
post.bottpurchase <- table(data$postcode, data$bottles_avgweek)
post.bottreuse <- table(data$postcode, data$bottles_reuses)
post.bottdisp <- table(data$postcode, data$bottles_disposal)
post.tubspurchase <- table(data$postcode, data$tubs_avgweek)
post.tubreuse <- table(data$postcode, data$tubs_reuses)
post.tubdisp <- table(data$postcode, data$tubs_disposal)
post.filmpurchase <- table(data$postcode, data$film_avgweek)
post.filmreuse <- table(data$postcode, data$film_reuses)
post.filmdisp <- table(data$postcode, data$film_disposal)
post.bagpurchase <- table(data$postcode, data$bags_avgweek)
post.bagreuse <- table(data$postcode, data$bags_reuses)
post.bagdisp <- table(data$postcode, data$bags_disposal)
```

```
addmargins(post.choice)
```

```
##
##       Always As often as they can If cheaper or preferred Rarely or never Sum
##   P01      8                   31                      23               7  69
##   P02      3                   42                      19               2  66
##   P03      4                   30                      14               1  49
##   P04     14                   41                      22               5  82
##   P05      9                   37                      20               6  72
##   P06      8                   27                      18               9  62
##   Sum     46                  208                     116              30 400
```

```
addmargins(post.barrier)
```

```
##
```

```
##       Council collection Unclear information Difficult transport
##   P01                 19                  11                   3
##   P02                 22                   8                   8
##   P03                 16                   6                   3
##   P04                 22                  19                   8
##   P05                 20                   7                   5
##   P06                 16                  11                   4
##   Sum                115                  62                  31
##
##       No local facilities No support Ends up in landfills Forgetting
##   P01                   7          3                    9          1
##   P02                   4          4                    1          5
##   P03                   4          2                    8          4
##   P04                   5          6                    4          7
##   P05                   9          2                    8          4
##   P06                   6          1                    3          4
##   Sum                  35         18                   33         25
##
##       Recycling a hassle Household disagrees Recycling not important Other
##   P01                  2                   0                       1     1
##   P02                  1                   1                       0     1
##   P03                  1                   0                       0     0
##   P04                  1                   0                       1     0
##   P05                  2                   2                       1     1
##   P06                  3                   2                       2     1
##   Sum                 10                   5                       5     4
##
##       Already doing everything Sum
##   P01                       12  69
##   P02                       11  66
##   P03                        5  49
##   P04                        9  82
##   P05                       11  72
##   P06                        9  62
##   Sum                       57 400
```

```
addmargins(post.barrier2)
```

```
##
##       Limited alternatives No SUP-free alternatives Alternatives expensive
##   P01                   20                       15                     11
##   P02                   13                       13                     16
##   P03                    8                       11                     14
##   P04                   18                       16                     14
##   P05                   13                       20                     15
##   P06                   18                       10                     15
##   Sum                   90                       85                     85
##
##       Limited functioning Forgetting reusables Reducing not important Other
##   P01                  10                    5                      2     0
##   P02                   5                   11                      3     2
##   P03                   4                    4                      0     1
##   P04                   7                    7                      4     1
##   P05                   6                    9                      4     2
```

```
##   P06                  7                    4                      0     2
##   Sum                 39                   40                     13     8
##
##       No barriers Sum
##   P01           6  69
##   P02           3  66
##   P03           7  49
##   P04          15  82
##   P05           3  72
##   P06           6  62
##   Sum          40 400
```

```r
addmargins(post.mainconsid)
```

```
##
##       Value for money Price Quality Deals/discounts Use-by-dates/longevity
##   P01              22    16      12               6                      5
##   P02              19    17       8               6                      3
##   P03              18     9      13               2                      1
##   P04              21    25      16               6                      0
##   P05              25    15      20               3                      2
##   P06              14    15      19               3                      3
##   Sum             119    97      88              26                     14
##
##       Convenience Ease of recycling packaging Sustainability Brand Ethics Other
##   P01           2                           1              3     1      1     0
##   P02           2                           7              2     1      1     0
##   P03           0                           4              0     1      1     0
##   P04           2                           4              3     1      2     2
##   P05           1                           3              3     0      0     0
##   P06           3                           3              2     0      0     0
##   Sum          10                          22             13     4      5     2
##
##       Sum
##   P01  69
##   P02  66
##   P03  49
##   P04  82
##   P05  72
##   P06  62
##   Sum 400
```

```r
addmargins(post.litter)
```

```
##
##       Strongly agree Agree Neither agree nor disagree Disagree
##   P01             26    31                          8        1
##   P02             37    20                          9        0
##   P03             19    22                          7        1
##   P04             37    31                         11        2
##   P05             25    36                          8        3
##   P06             29    22                         10        1
##   Sum            173   162                         53        8
```

```
##
##        Strongly disagree Sum
##    P01                 3  69
##    P02                 0  66
##    P03                 0  49
##    P04                 1  82
##    P05                 0  72
##    P06                 0  62
##    Sum                 4 400
```

```r
addmargins(post.attitude)
```

```
##
##        1 (Not concerned)   2   3   4   5   6 7 (Very concerned Sum
##    P01                 5   2   9   9  15  11                18  69
##    P02                 5   3   9  13  12  10                14  66
##    P03                 2   2   3  12  16   8                 6  49
##    P04                 4   5   5  20  25  10                13  82
##    P05                 4   6   7   8  26   9                12  72
##    P06                 7   2   8  11  20   8                 6  62
##    Sum                27  20  41  73 114  56                69 400
```

```r
addmargins(post.ocean)
```

```
##
##        Always Most of the time Sometimes Rarely Never Sum
##    P01      5               19        31     11     3  69
##    P02      3               18        34      9     2  66
##    P03      0               14        28      5     2  49
##    P04      6               22        38     11     5  82
##    P05      4               17        37     12     2  72
##    P06      1               14        32     12     3  62
##    Sum     19              104       200     60    17 400
```

```r
addmargins(post.actions)
```

```
##
##        Yes, definitely Yes, probably No, probably not No, definitely not
##    P01              21            31                12                  2
##    P02              30            22                10                  1
##    P03              19            25                 3                  1
##    P04              22            43                 9                  6
##    P05              27            38                 5                  0
##    P06              23            32                 4                  1
##    Sum             142           191                43                 11
##
##        I don't know Sum
##    P01            3  69
##    P02            3  66
##    P03            1  49
##    P04            2  82
##    P05            2  72
```

```
##   P06            2  62
##   Sum           13 400
```

```r
addmargins(post.attplast)
```

```
##
##       Will go out of their way to avoid
##   P01                                20
##   P02                                15
##   P03                                15
##   P04                                20
##   P05                                17
##   P06                                15
##   Sum                               102
##
##       If option is readily available, will avoid
##   P01                                         33
##   P02                                         37
##   P03                                         31
##   P04                                         43
##   P05                                         38
##   P06                                         36
##   Sum                                        218
##
##       Will avoid only without extra costs Not a priority Sum
##   P01                                  14              2  69
##   P02                                  10              4  66
##   P03                                   2              1  49
##   P04                                  13              6  82
##   P05                                  13              4  72
##   P06                                   6              5  62
##   Sum                                  58             22 400
```

```r
addmargins(post.zerow)
```

```
##
##       Yes, shops regularly Yes, shops occasionally Yes, visited at least once
##   P01                    3                       3                          5
##   P02                    0                       1                          3
##   P03                    1                       4                          1
##   P04                    2                       6                          6
##   P05                    6                       9                          6
##   P06                    2                       7                          3
##   Sum                   14                      30                         24
##
##       Yes, never visited No, but would like to shop there
##   P01                 15                               30
##   P02                 11                               33
##   P03                 12                               23
##   P04                 22                               40
##   P05                 22                               23
##   P06                  6                               26
##   Sum                 88                              175
```

```
##
##       No, not likely to shop there Sum
##   P01                           13  69
##   P02                           18  66
##   P03                            8  49
##   P04                            6  82
##   P05                            6  72
##   P06                           18  62
##   Sum                           69 400
```

```r
## SUPs
addmargins(post.bottpurchase)
```

```
##
##       None 1-2 3-5 6-10 11-15 16+ I don't know Sum
##   P01    1  24  21   15     6   2            0  69
##   P02    0  21  26   13     5   0            1  66
##   P03    7  18  13    7     2   1            1  49
##   P04    2  26  31   14     3   2            4  82
##   P05    1  29  24   13     2   3            0  72
##   P06    1  19  19   12     1   6            4  62
##   Sum   12 137 134   74    19  14           10 400
```

```r
addmargins(post.bottreuse)
```

```
##
##       Never Once 2-4 times 5-10 times More often Sum
##   P01    13   13        18         10         15  69
##   P02    11    7        24         12         12  66
##   P03     5   10        22          4          8  49
##   P04    20   10        26         11         15  82
##   P05    13   11        21         13         14  72
##   P06     9   15        18          7         13  62
##   Sum    71   66       129         57         77 400
```

```r
addmargins(post.bottdisp)
```

```
##
##       General waste bin Recycling bin Recycling centre
##   P01                 4            57                1
##   P02                 3            58                1
##   P03                 2            44                0
##   P04                 6            64                5
##   P05                 7            53                2
##   P06                 5            52                1
##   Sum                27           328               10
##
##       Specialist waste collection Landfill Deposit return scheme
##   P01                           1        1                     2
##   P02                           1        0                     1
##   P03                           1        0                     0
##   P04                           0        1                     0
```

```
##    P05                          3        2                      0
##    P06                          1        1                      1
##    Sum                          7        5                      4
##
##        Indefinite storage Other I don't know Sum
##    P01                  2     1            0  69
##    P02                  2     0            0  66
##    P03                  1     1            0  49
##    P04                  5     0            1  82
##    P05                  5     0            0  72
##    P06                  1     0            0  62
##    Sum                 16     2            1 400
```

```
addmargins(post.tubspurchase)
```

```
##
##        None 1-2 3-5 6-10 11-15 16+ I don't know Sum
##    P01     2  22  30    8     5   2            0  69
##    P02     1  14  28   13     7   2            1  66
##    P03     1  13  12   19     4   0            0  49
##    P04     3  24  26   17     7   2            3  82
##    P05     3  15  26   18     4   4            2  72
##    P06     2  20  21   10     2   3            4  62
##    Sum    12 108 143   85    29  13           10 400
```

```
addmargins(post.tubreuse)
```

```
##
##        Never Once 2-4 times 5-10 times More often Sum
##    P01     14   10        15          7         23  69
##    P02      9    5        19         13         20  66
##    P03      7    9         6          4         23  49
##    P04     14    6        19         17         26  82
##    P05      8    7        20         11         26  72
##    P06      9    4        18          6         25  62
##    Sum     61   41        97         58        143 400
```

```
addmargins(post.tubdisp)
```

```
##
##        General waste bin Recycling bin Recycling centre
##    P01                12            44                4
##    P02                21            33                3
##    P03                10            25                6
##    P04                15            40                5
##    P05                15            39                4
##    P06                13            30                7
##    Sum                86           211               29
##
##        Specialist waste collection Landfill Deposit return scheme
##    P01                           1        1                      1
##    P02                           0        1                      0
```

```
##    P03                          2        0                      0
##    P04                          6        0                      2
##    P05                          2        1                      1
##    P06                          1        0                      0
##    Sum                         12        3                      4
##
##        Indefinite storage Other I don't know Sum
##    P01                  6     0            0  69
##    P02                  6     1            1  66
##    P03                  4     0            2  49
##    P04                 11     3            0  82
##    P05                  8     0            2  72
##    P06                  7     3            1  62
##    Sum                 42     7            6 400
```

```r
addmargins(post.filmpurchase)
```

```
##
##        None 1-2 3-5 6-10 11-15 16+ I don't know Sum
##    P01    5  13  23   18     8   2            0  69
##    P02    1  13  21   19     8   2            2  66
##    P03    2   9  15   19     1   3            0  49
##    P04    3  26  26   18     4   1            4  82
##    P05    0  13  18   21    13   6            1  72
##    P06    1  10  19   18     5   4            5  62
##    Sum   12  84 122  113    39  18           12 400
```

```r
addmargins(post.filmreuse)
```

```
##
##        Never Once 2-4 times 5-10 times More often Sum
##    P01    37   15        11          3          3  69
##    P02    43   11         7          4          1  66
##    P03    30   15         2          2          0  49
##    P04    46   13        16          1          6  82
##    P05    44   13        10          2          3  72
##    P06    36   16         8          0          2  62
##    Sum   236   83        54         12         15 400
```

```r
addmargins(post.filmdisp)
```

```
##
##        General waste bin Recycling bin Recycling centre
##    P01                45            16                4
##    P02                43            19                0
##    P03                38            11                0
##    P04                45            22                6
##    P05                50            12                4
##    P06                44            12                3
##    Sum               265            92               17
##
##        Specialist waste collection Landfill Deposit return scheme
```

```
##   P01                          1        0                     0
##   P02                          1        0                     0
##   P03                          0        0                     0
##   P04                          2        1                     1
##   P05                          2        1                     1
##   P06                          0        1                     0
##   Sum                          6        3                     2
##
##       Indefinite storage Other I don't know Sum
##   P01                  0     2            1  69
##   P02                  0     2            1  66
##   P03                  0     0            0  49
##   P04                  2     1            2  82
##   P05                  0     0            2  72
##   P06                  1     1            0  62
##   Sum                  3     6            6 400
```

```
addmargins(post.bagpurchase)
```

```
##
##       None 1-2 3-5 6-10 11-15 16+ I don't know Sum
##   P01   37  14   7    5     5   1            0  69
##   P02   38  16   6    3     1   0            2  66
##   P03   30  12   4    2     0   1            0  49
##   P04   50  16   8    3     3   0            2  82
##   P05   36  13  13    4     4   2            0  72
##   P06   41   6   5    2     1   4            3  62
##   Sum  232  77  43   19    14   8            7 400
```

```
addmargins(post.bagreuse)
```

```
##
##       Never Once 2-4 times 5-10 times More often Sum
##   P01     7   10        22          8         22  69
##   P02     2    5        18         12         29  66
##   P03     4    8        10          6         21  49
##   P04     9    3        10         20         40  82
##   P05     3   12        15         15         27  72
##   P06     6    6        18          9         23  62
##   Sum    31   44        93         70        162 400
```

```
addmargins(post.bagdisp)
```

```
##
##       General waste bin Recycling bin Recycling centre
##   P01                22            15                9
##   P02                29            13                3
##   P03                24            14                2
##   P04                17            19                5
##   P05                27            13                5
##   P06                24            18                5
##   Sum               143            92               29
```

```
##
##       Specialist waste collection Landfill Deposit return scheme
##   P01                           3        0                     2
##   P02                           0        0                     3
##   P03                           0        0                     0
##   P04                           4        0                     1
##   P05                           0        2                     5
##   P06                           1        0                     0
##   Sum                           8        2                    11
##
##       Indefinite storage Other I don't know Sum
##   P01                 12     4            2  69
##   P02                 16     0            2  66
##   P03                  8     0            1  49
##   P04                 25     9            2  82
##   P05                 12     7            1  72
##   P06                 10     3            1  62
##   Sum                 83    23            9 400
```

Pearson's Chi-squared tests for independence

Tests without Monte Carlo simulated p-value:

Age

```
chi.age.choice <- chisq.test(age.choice)
chi.age.attitude <- chisq.test(age.attitude)
chi.age.bottreuse <- chisq.test(age.bottreuse)
chi.age.tubreuse <- chisq.test(age.tubreuse)
chi.age.bagreuse <- chisq.test(age.bagreuse)
chi.age.attplast <- chisq.test(age.attplast)
```

```
chi.age.choice
```

```
##
##  Pearson's Chi-squared test
##
## data:  age.choice
## X-squared = 5.0135, df = 6, p-value = 0.5421
```

```
chi.age.attitude
```

```
##
##  Pearson's Chi-squared test
##
## data:  age.attitude
## X-squared = 28.288, df = 12, p-value = 0.00502
```

```r
chi.age.bottreuse
```

```
##
##  Pearson's Chi-squared test
##
## data:  age.bottreuse
## X-squared = 8.4416, df = 8, p-value = 0.3916
```

```r
chi.age.tubreuse
```

```
##
##  Pearson's Chi-squared test
##
## data:  age.tubreuse
## X-squared = 16.234, df = 8, p-value = 0.03915
```

```r
chi.age.bagreuse
```

```
##
##  Pearson's Chi-squared test
##
## data:  age.bagreuse
## X-squared = 16.091, df = 8, p-value = 0.0411
```

```r
chi.age.attplast
```

```
##
##  Pearson's Chi-squared test
##
## data:  age.attplast
## X-squared = 4.3744, df = 6, p-value = 0.6261
```

```r
chi.age.choice$residuals
```

```
##
##                    Always As often as they can If cheaper or preferred
##   30 and under  0.3355942           -0.8963622               1.1216126
##   31-50         0.6134848            0.2592350              -0.5654914
##   50+          -1.0495187            0.5143584              -0.3528211
##
##                Rarely or never
##   30 and under      -0.2608488
##   31-50             -0.3302891
##   50+                0.6390097
```

```r
chi.age.attitude$residuals
```

```
##
##                1 (Not concerned)           2           3           4
```

```
##   30 and under        2.29353503  0.81520740  1.36724672  0.50800252
##   31-50              -1.41583160  0.06741999  0.22602317  0.68461113
##   50+                -0.40851905 -0.83344352 -1.53405227 -1.29436869
##
##                             5           6 7 (Very concerned
##   30 and under -0.25009111 -0.90062392       -2.31723595
##   31-50         0.11860432  0.07252407       -0.24682436
##   50+           0.08769849  0.74347218        2.43562469
```

```
chi.age.bottreuse$residuals
```

```
##
##                      Never        Once   2-4 times  5-10 times  More often
##   30 and under -1.70318845 -0.24135844  1.00330314  0.34519909  0.26331698
##   31-50         0.31488876  0.73484692 -0.10087706 -0.81469753 -0.15118579
##   50+           1.19163667 -0.66357504 -0.80401176  0.66407236 -0.06060631
```

```
chi.age.tubreuse$residuals
```

```
##
##                     Never       Once  2-4 times 5-10 times More often
##   30 and under -1.1877822 -0.4793442 -0.9959483  0.7930998  1.3476090
##   31-50        -0.5481849  0.4614640  1.1204665  0.8868245 -1.3766650
##   50+           1.7570152 -0.1142908 -0.4324452 -1.8012855  0.4169807
```

```
chi.age.bagreuse$residuals
```

```
##
##                     Never       Once  2-4 times 5-10 times More often
##   30 and under -1.0556282 -0.6922149  0.8281193 -0.4769660  0.5086151
##   31-50        -0.4440549  1.9636364  0.3251588  0.2162250 -1.2176130
##   50+           1.5095009 -1.7295787 -1.1562011  0.1792843  0.9992347
```

```
chi.age.attplast$residuals
```

```
##
##                Will go out of their way to avoid
##   30 and under                       -0.05170793
##   31-50                               0.31645316
##   50+                                -0.33394976
##
##                If option is readily available, will avoid
##   30 and under                                 0.24892125
##   31-50                                        0.31448225
##   50+                                         -0.60894120
##
##                Will avoid only without extra costs Not a priority
##   30 and under                          0.01681941    -0.69954278
##   31-50                                -0.69679067    -0.53997245
##   50+                                   0.82484314     1.29664799
```

```r
chi.age.choice$observed
```

```
##
##               Always As often as they can If cheaper or preferred
##   30 and under     13                   47                      36
##   31-50            23                   94                      47
##   50+              10                   67                      33
##
##               Rarely or never
##   30 and under               7
##   31-50                     12
##   50+                       11
```

```r
chi.age.choice$expected
```

```
##
##               Always As often as they can If cheaper or preferred
##   30 and under 11.845                53.56                   29.87
##   31-50        20.240                91.52                   51.04
##   50+          13.915                62.92                   35.09
##
##               Rarely or never
##   30 and under           7.725
##   31-50                 13.200
##   50+                    9.075
```

```r
chi.age.attitude$observed
```

```
##
##               1 (Not concerned)  2  3  4  5  6 7 (Very concerned
##   30 and under                13  7 15 21 28 11                8
##   31-50                        7  9 19 36 51 25               29
##   50+                          7  4  7 16 35 20               32
```

```r
chi.age.attitude$expected
```

```
##
##               1 (Not concerned)    2       3       4      5     6
##   30 and under            6.9525 5.15 10.5575 18.7975 29.355 14.42
##   31-50                  11.8800 8.80 18.0400 32.1200 50.160 24.64
##   50+                     8.1675 6.05 12.4025 22.0825 34.485 16.94
##
##               7 (Very concerned
##   30 and under           17.7675
##   31-50                  30.3600
##   50+                    20.8725
```

```r
chi.age.bottreuse$observed
```

```
##
##                Never Once 2-4 times 5-10 times More often
##   30 and under    11   16        39         16         21
##   31-50           33   33        56         21         33
##   50+             27   17        34         20         23
```

```r
chi.age.bottreuse$expected
```

```
##
##                  Never   Once 2-4 times 5-10 times More often
##   30 and under 18.2825 16.995   33.2175    14.6775    19.8275
##   31-50        31.2400 29.040   56.7600    25.0800    33.8800
##   50+          21.4775 19.965   39.0225    17.2425    23.2925
```

```r
chi.age.tubreuse$observed
```

```
##
##                Never Once 2-4 times 5-10 times More often
##   30 and under    11    9        20         18         45
##   31-50           24   20        50         30         52
##   50+             26   12        27         10         46
```

```r
chi.age.tubreuse$expected
```

```
##
##                  Never    Once 2-4 times 5-10 times More often
##   30 and under 15.7075 10.5575   24.9775     14.935    36.8225
##   31-50        26.8400 18.0400   42.6800     25.520    62.9200
##   50+          18.4525 12.4025   29.3425     17.545    43.2575
```

```r
chi.age.bagreuse$observed
```

```
##
##                Never Once 2-4 times 5-10 times More often
##   30 and under     5    9        28         16         45
##   31-50           12   28        43         32         61
##   50+             14    7        22         22         56
```

```r
chi.age.bagreuse$expected
```

```
##
##                  Never  Once 2-4 times 5-10 times More often
##   30 and under  7.9825 11.33   23.9475     18.025     41.715
##   31-50        13.6400 19.36   40.9200     30.800     71.280
##   50+           9.3775 13.31   28.1325     21.175     49.005
```

```r
chi.age.attplast$observed
```

```
##
##                Will go out of their way to avoid
##   30 and under                               26
##   31-50                                      47
##   50+                                        29
##
##                If option is readily available, will avoid
##   30 and under                                        58
##   31-50                                               99
##   50+                                                 61
##
##                Will avoid only without extra costs Not a priority
##   30 and under                                  15              4
##   31-50                                         22              8
##   50+                                           21             10
```

```
chi.age.attplast$expected
```

```
##
##                Will go out of their way to avoid
##   30 and under                           26.265
##   31-50                                  44.880
##   50+                                    30.855
##
##                If option is readily available, will avoid
##   30 and under                                    56.135
##   31-50                                           95.920
##   50+                                             65.945
##
##                Will avoid only without extra costs Not a priority
##   30 and under                              14.935          5.665
##   31-50                                     25.520          9.680
##   50+                                       17.545          6.655
```

Education

All tests for 'education' with Monte Carlo. See the results under *Pearson's Chi-square coefficients, i.e. Pearson's chi-square tests with Monte Carlo.*

Gender

All tests for 'gender' with Monte Carlo. See the results under *Pearson's Chi-square coefficients, i.e. Pearson's chi-square tests with Monte Carlo.*

Income

All tests for 'income' with Monte Carlo. See the results under *Pearson's Chi-square coefficients, i.e. Pearson's chi-square tests with Monte Carlo.*

Vehicle ownership

```
chi.veh.choice <- chisq.test(veh.choice)
chi.veh.attitude <- chisq.test(veh.attitude)
chi.veh.ocean <- chisq.test(veh.ocean)
chi.veh.attplast <- chisq.test(veh.attplast)
chi.veh.bottreuse <- chisq.test(veh.bottreuse)
chi.veh.tubreuse <- chisq.test(veh.tubreuse)
chi.veh.bagreuse <- chisq.test(veh.bagreuse)
```

```
chi.veh.choice
```

```
##
##  Pearson's Chi-squared test
##
## data:  veh.choice
## X-squared = 6.0768, df = 3, p-value = 0.1079
```

```
chi.veh.attitude
```

```
##
##  Pearson's Chi-squared test
##
## data:  veh.attitude
## X-squared = 2.6753, df = 6, p-value = 0.8484
```

```
chi.veh.ocean
```

```
##
##  Pearson's Chi-squared test
##
## data:  veh.ocean
## X-squared = 2.237, df = 4, p-value = 0.6923
```

```
chi.veh.attplast
```

```
##
##  Pearson's Chi-squared test
##
## data:  veh.attplast
## X-squared = 3.3653, df = 3, p-value = 0.3387
```

```
chi.veh.bottreuse
```

```
##
##  Pearson's Chi-squared test
##
## data:  veh.bottreuse
## X-squared = 3.2788, df = 4, p-value = 0.5123
```

```
chi.veh.tubreuse
```

```
##
##  Pearson's Chi-squared test
##
## data:  veh.tubreuse
## X-squared = 4.2654, df = 4, p-value = 0.3713
```

```
chi.veh.bagreuse
```

```
##
##  Pearson's Chi-squared test
##
## data:  veh.bagreuse
## X-squared = 1.4691, df = 4, p-value = 0.8321
```

```
chi.veh.choice$residuals
```

```
##
##                          Always As often as they can If cheaper or preferred
##   No vehicle         -0.9691216           -0.6842615               1.6718559
##   1 or more vehicles  0.6268949            0.4426277              -1.0814721
##
##                      Rarely or never
##   No vehicle              -0.2857244
##   1 or more vehicles       0.1848263
```

```
chi.veh.attitude$residuals
```

```
##
##                      1 (Not concerned)           2           3           4
##   No vehicle                1.07538953  0.04116935 -0.60239463  0.10020295
##   1 or more vehicles       -0.69563633 -0.02663118  0.38967051 -0.06481820
##
##                                5           6 7 (Very concerned
##   No vehicle         -0.45351569 -0.12793764        0.36461149
##   1 or more vehicles  0.29336531  0.08275891       -0.23585593
```

```
chi.veh.ocean$residuals
```

```
##
##                           Always Most of the time   Sometimes      Rarely
##   No vehicle         -1.10032231       0.05777265  0.39056673  0.07130740
##   1 or more vehicles  0.71176457      -0.03737135 -0.25264558 -0.04612656
##
##                            Never
##   No vehicle         -0.45324244
##   1 or more vehicles  0.29318856
```

```r
chi.veh.attplast$residuals
```

```
##
##                      Will go out of their way to avoid
##   No vehicle                                -0.01640708
##   1 or more vehicles                         0.01061324
##
##                      If option is readily available, will avoid
##   No vehicle                                          0.08604187
##   1 or more vehicles                                 -0.05565783
##
##                      Will avoid only without extra costs Not a priority
##   No vehicle                                  0.69867119    -1.36994483
##   1 or more vehicles                         -0.45194885     0.88617507
```

```r
chi.veh.bottreuse$residuals
```

```
##
##                            Never        Once   2-4 times  5-10 times
##   No vehicle         -0.86199913 -0.55977562  0.15318842  0.04511524
##   1 or more vehicles  0.55760065  0.36210159 -0.09909287 -0.02918366
##
##                       More often
##   No vehicle          1.10889039
##   1 or more vehicles -0.71730700
```

```r
chi.veh.tubreuse$residuals
```

```
##
##                           Never       Once  2-4 times 5-10 times More often
##   No vehicle         -0.2345563 -1.1774730  0.4458528 -0.7518572  0.8953043
##   1 or more vehicles  0.1517273  0.7616710 -0.2884084  0.4863533 -0.5791448
```

```r
chi.veh.bagreuse$residuals
```

```
##
##                           Never       Once  2-4 times 5-10 times More often
##   No vehicle         -0.3786288 -0.8271398  0.2987871  0.2970802  0.1750318
##   1 or more vehicles  0.2449233  0.5350512 -0.1932762 -0.1921720 -0.1132226
```

```r
chi.veh.choice$observed
```

```
##
##                      Always As often as they can If cheaper or preferred
##   No vehicle             10                   56                      44
##   1 or more vehicles     36                  152                      72
##
##                      Rarely or never
##   No vehicle                       8
##   1 or more vehicles              22
```

```r
chi.veh.choice$expected
```

```
##
##                      Always As often as they can If cheaper or preferred
##   No vehicle          13.57               61.36                   34.22
##   1 or more vehicles  32.43              146.64                   81.78
##
##                      Rarely or never
##   No vehicle                    8.85
##   1 or more vehicles           21.15
```

```r
chi.veh.attitude$observed
```

```
##
##                      1 (Not concerned)  2  3  4  5  6 7 (Very concerned
##   No vehicle                        11  6 10 22 31 16                22
##   1 or more vehicles                16 14 31 51 83 40                47
```

```r
chi.veh.attitude$expected
```

```
##
##                      1 (Not concerned)    2      3      4     5     6
##   No vehicle                     7.965  5.9 12.095 21.535 33.63 16.52
##   1 or more vehicles            19.035 14.1 28.905 51.465 80.37 39.48
##
##                      7 (Very concerned
##   No vehicle                    20.355
##   1 or more vehicles            48.645
```

```r
chi.veh.ocean$observed
```

```
##
##                      Always Most of the time Sometimes Rarely Never
##   No vehicle              3               31        62     18     4
##   1 or more vehicles     16               73       138     42    13
```

```r
chi.veh.ocean$expected
```

```
##
##                      Always Most of the time Sometimes Rarely  Never
##   No vehicle          5.605            30.68        59   17.7  5.015
##   1 or more vehicles 13.395            73.32       141   42.3 11.985
```

```r
chi.veh.attplast$observed
```

```
##
##                      Will go out of their way to avoid
##   No vehicle                                        30
##   1 or more vehicles                                72
```

```
##
##                        If option is readily available, will avoid
##   No vehicle                                                   65
##   1 or more vehicles                                          153
##
##                        Will avoid only without extra costs Not a priority
##   No vehicle                                            20              3
##   1 or more vehicles                                    38             19
```

```r
chi.veh.attplast$expected
```

```
##
##                        Will go out of their way to avoid
##   No vehicle                                      30.09
##   1 or more vehicles                              71.91
##
##                        If option is readily available, will avoid
##   No vehicle                                                64.31
##   1 or more vehicles                                       153.69
##
##                        Will avoid only without extra costs Not a priority
##   No vehicle                                         17.11           6.49
##   1 or more vehicles                                 40.89          15.51
```

```r
chi.veh.bottreuse$observed
```

```
##
##                        Never Once 2-4 times 5-10 times More often
##   No vehicle              17   17        39         17         28
##   1 or more vehicles      54   49        90         40         49
```

```r
chi.veh.bottreuse$expected
```

```
##
##                         Never  Once 2-4 times 5-10 times More often
##   No vehicle           20.945 19.47    38.055     16.815     22.715
##   1 or more vehicles   50.055 46.53    90.945     40.185     54.285
```

```r
chi.veh.tubreuse$observed
```

```
##
##                        Never Once 2-4 times 5-10 times More often
##   No vehicle              17    8        31         14         48
##   1 or more vehicles      44   33        66         44         95
```

```r
chi.veh.tubreuse$expected
```

```
##
##                         Never   Once 2-4 times 5-10 times More often
##   No vehicle           17.995 12.095    28.615      17.11     42.185
##   1 or more vehicles   43.005 28.905    68.385      40.89    100.815
```

```r
chi.veh.bagreuse$observed
```

```
##
##                        Never Once 2-4 times 5-10 times More often
##   No vehicle               8   10        29         22         49
##   1 or more vehicles      23   34        64         48        113
```

```r
chi.veh.bagreuse$expected
```

```
##
##                         Never  Once 2-4 times 5-10 times More often
##   No vehicle            9.145 12.98    27.435      20.65      47.79
##   1 or more vehicles 21.855 31.02    65.565      49.35     114.21
```

The remaining tests for 'vehicle ownership' with Monte Carlo. See the results under *Pearson's Chi-square coefficients, i.e. Pearson's chi-square tests with Monte Carlo.*

Living situation

All tests for 'living situation' with Monte Carlo. See the results under *Pearson's Chi-square coefficients, i.e. Pearson's chi-square tests with Monte Carlo.*

Postcode

```r
chi.post.bottreuse <- chisq.test(post.bottreuse)
chi.post.tubreuse <- chisq.test(post.tubreuse)
```

```r
chi.post.bottreuse
```

```
##
##  Pearson's Chi-squared test
##
## data:  post.bottreuse
## X-squared = 17.119, df = 20, p-value = 0.6453
```

```r
chi.post.tubreuse
```

```
##
##  Pearson's Chi-squared test
##
## data:  post.tubreuse
## X-squared = 23.175, df = 20, p-value = 0.2803
```

```r
chi.post.bottreuse$residuals
```

```
##
##             Never        Once   2-4 times  5-10 times  More often
##   P01  0.21502194  0.47863672 -0.90147755  0.05341741  0.47125594
##   P02 -0.20889829 -1.17878788  0.58848176  0.84617066 -0.19778884
##   P03 -1.25375005  0.67348630  1.55902700 -1.12869079 -0.46642436
##   P04  1.42722281 -0.95967859 -0.08653429 -0.20039008 -0.19758199
##   P05  0.06153997 -0.25531390 -0.46070388  0.85541504  0.03760507
##   P06 -0.60439290  1.49135338 -0.44615133 -0.61735190  0.30827506
```

```
chi.post.tubreuse$residuals
```

```
##
##             Never        Once   2-4 times  5-10 times  More often
##   P01  1.07203181  1.10080508 -0.42353843 -0.95002696 -0.33574015
##   P02 -0.33569333 -0.67859524  0.74863304  1.10876154 -0.74009744
##   P03 -0.17284979  1.77480325 -1.70650680 -1.16487564  1.30991338
##   P04  0.42276531 -0.82955698 -0.19846342  1.48193779 -0.61226380
##   P05 -0.89932174 -0.13987993  0.60787124  0.17331565  0.05124707
##   P06 -0.14797237 -0.93418553  0.76466812 -0.99722083  0.60217011
```

```
chi.post.bottreuse$observed
```

```
##
##       Never Once 2-4 times 5-10 times More often
##   P01    13   13        18         10         15
##   P02    11    7        24         12         12
##   P03     5   10        22          4          8
##   P04    20   10        26         11         15
##   P05    13   11        21         13         14
##   P06     9   15        18          7         13
```

```
chi.post.bottreuse$expected
```

```
##
##         Never   Once 2-4 times 5-10 times More often
##   P01 12.2475 11.385   22.2525     9.8325    13.2825
##   P02 11.7150 10.890   21.2850     9.4050    12.7050
##   P03  8.6975  8.085   15.8025     6.9825     9.4325
##   P04 14.5550 13.530   26.4450    11.6850    15.7850
##   P05 12.7800 11.880   23.2200    10.2600    13.8600
##   P06 11.0050 10.230   19.9950     8.8350    11.9350
```

```
chi.post.tubreuse$observed
```

```
##
##       Never Once 2-4 times 5-10 times More often
##   P01    14   10        15          7         23
##   P02     9    5        19         13         20
##   P03     7    9         6          4         23
##   P04    14    6        19         17         26
##   P05     8    7        20         11         26
##   P06     9    4        18          6         25
```

```r
chi.post.tubreuse$expected
```

```
##
##         Never   Once 2-4 times 5-10 times More often
##   P01 10.5225 7.0725   16.7325     10.005    24.6675
##   P02 10.0650 6.7650   16.0050      9.570    23.5950
##   P03  7.4725 5.0225   11.8825      7.105    17.5175
##   P04 12.5050 8.4050   19.8850     11.890    29.3150
##   P05 10.9800 7.3800   17.4600     10.440    25.7400
##   P06  9.4550 6.3550   15.0350      8.990    22.1650
```

The remaining tests for 'postcode' with Monte Carlo. See the results under *Pearson's Chi-square coefficients, i.e. Pearson's chi-square tests with Monte Carlo*.

Pearson's Chi-square coefficients, i.e. Pearson's chi-square tests with Monte Carlo

If the non-simulated test resulted in a warning, then Monte Carlo simulation was applied to the P-value.

Age

```r
chi.age.barrier <- chisq.test(age.barrier)
```

```
## Warning in chisq.test(age.barrier): Chi-squared approximation may be incorrect
```

```r
chi.age.barrier.sim <- chisq.test(age.barrier, simulate.p.value = TRUE)
chi.age.barrier.sim
```

```
##
##  Pearson's Chi-squared test with simulated p-value (based on 2000
##  replicates)
##
## data:  age.barrier
## X-squared = 55.559, df = NA, p-value = 0.0009995
```

```r
chi.age.barrier2 <- chisq.test(age.barrier2)
```

```
## Warning in chisq.test(age.barrier2): Chi-squared approximation may be incorrect
```

```r
chi.age.barrier2.sim <- chisq.test(age.barrier2, simulate.p.value = TRUE)
chi.age.barrier2.sim
```

```
##
##  Pearson's Chi-squared test with simulated p-value (based on 2000
##  replicates)
##
## data:  age.barrier2
## X-squared = 45.121, df = NA, p-value = 0.0004998
```

```r
chi.age.mainconsid <- chisq.test(age.mainconsid)
```

```
## Warning in chisq.test(age.mainconsid): Chi-squared approximation may be
## incorrect
```

```r
chi.age.mainconsid.sim <- chisq.test(age.mainconsid, simulate.p.value =
TRUE)
chi.age.mainconsid.sim
```

```
##
##  Pearson's Chi-squared test with simulated p-value (based on 2000
##  replicates)
##
## data:  age.mainconsid
## X-squared = 45.252, df = NA, p-value = 0.001499
```

```r
chi.age.litter.sim <- chisq.test(age.litter, simulate.p.value = TRUE)
chi.age.litter.sim
```

```
##
##  Pearson's Chi-squared test with simulated p-value (based on 2000
##  replicates)
##
## data:  age.litter
## X-squared = 10.393, df = NA, p-value = 0.2344
```

```r
chi.age.ocean.sim <- chisq.test(age.ocean, simulate.p.value = TRUE)
chi.age.ocean.sim
```

```
##
##  Pearson's Chi-squared test with simulated p-value (based on 2000
##  replicates)
##
## data:  age.ocean
## X-squared = 26.28, df = NA, p-value = 0.001499
```

```r
chi.age.actions.sim <- chisq.test(age.actions, simulate.p.value = TRUE)
chi.age.actions.sim
```

```
##
##  Pearson's Chi-squared test with simulated p-value (based on 2000
##  replicates)
##
## data:  age.actions
## X-squared = 6.0794, df = NA, p-value = 0.6352
```

```r
chi.age.zerow <- chisq.test(age.zerow)
```

```
## Warning in chisq.test(age.zerow): Chi-squared approximation may be incorrect
```

```r
chi.age.zerow.sim <- chisq.test(age.zerow, simulate.p.value = TRUE)
chi.age.zerow.sim
```

```
##
##  Pearson's Chi-squared test with simulated p-value (based on 2000
##  replicates)
##
## data:  age.zerow
## X-squared = 42.405, df = NA, p-value = 0.0004998
```

```r
chi.age.bottpurchase <- chisq.test(age.bottpurchase)
```

```
## Warning in chisq.test(age.bottpurchase): Chi-squared approximation may be
## incorrect
```

```r
chi.age.bottpurchase.sim <- chisq.test(age.bottpurchase, simulate.p.value =
TRUE)
chi.age.bottpurchase.sim
```

```
##
##  Pearson's Chi-squared test with simulated p-value (based on 2000
##  replicates)
##
## data:  age.bottpurchase
## X-squared = 27.789, df = NA, p-value = 0.005997
```

```r
chi.age.bottdisp <- chisq.test(age.bottdisp)
```

```
## Warning in chisq.test(age.bottdisp): Chi-squared approximation may be incorrect
```

```r
chi.age.bottdisp.sim <- chisq.test(age.bottdisp, simulate.p.value = TRUE)
chi.age.bottdisp.sim
```

```
##
##  Pearson's Chi-squared test with simulated p-value (based on 2000
##  replicates)
##
## data:  age.bottdisp
## X-squared = 27.039, df = NA, p-value = 0.02599
```

```r
chi.age.tubpurchase <- chisq.test(age.tubpurchase)
```

```
## Warning in chisq.test(age.tubpurchase): Chi-squared approximation may be
## incorrect
```

```r
chi.age.tubpurchase.sim <- chisq.test(age.tubpurchase, simulate.p.value =
TRUE)
chi.age.tubpurchase.sim
```

```
##
##  Pearson's Chi-squared test with simulated p-value (based on 2000
##  replicates)
##
## data:  age.tubpurchase
## X-squared = 15.367, df = NA, p-value = 0.2244
```

```
chi.age.tubdisp <- chisq.test(age.tubdisp)
```

```
## Warning in chisq.test(age.tubdisp): Chi-squared approximation may be incorrect
```

```
chi.age.tubdisp.sim <- chisq.test(age.tubdisp, simulate.p.value = TRUE)
chi.age.tubdisp.sim
```

```
##
##  Pearson's Chi-squared test with simulated p-value (based on 2000
##  replicates)
##
## data:  age.tubdisp
## X-squared = 31.216, df = NA, p-value = 0.007996
```

```
chi.age.filmpur <- chisq.test(age.filmpur)
```

```
## Warning in chisq.test(age.filmpur): Chi-squared approximation may be incorrect
```

```
chi.age.filmpur.sim <- chisq.test(age.filmpur, simulate.p.value = TRUE)
chi.age.filmpur.sim
```

```
##
##  Pearson's Chi-squared test with simulated p-value (based on 2000
##  replicates)
##
## data:  age.filmpur
## X-squared = 24.006, df = NA, p-value = 0.02099
```

```
chi.age.filmreuse <- chisq.test(age.filmreuse)
```

```
## Warning in chisq.test(age.filmreuse): Chi-squared approximation may be incorrect
```

```
chi.age.filmreuse.sim <- chisq.test(age.filmreuse, simulate.p.value = TRUE)
chi.age.filmreuse.sim
```

```
##
##  Pearson's Chi-squared test with simulated p-value (based on 2000
##  replicates)
##
## data:  age.filmreuse
## X-squared = 26.219, df = NA, p-value = 0.0004998
```

```r
chi.age.filmdisp <- chisq.test(age.filmdisp)
```

```
## Warning in chisq.test(age.filmdisp): Chi-squared approximation may be incorrect
```

```r
chi.age.filmdisp.sim <- chisq.test(age.filmdisp, simulate.p.value = TRUE)
chi.age.filmdisp.sim
```

```
##
##  Pearson's Chi-squared test with simulated p-value (based on 2000
##  replicates)
##
## data:  age.filmdisp
## X-squared = 29.243, df = NA, p-value = 0.01549
```

```r
chi.age.bagpurchase <- chisq.test(age.bagpurchase)
```

```
## Warning in chisq.test(age.bagpurchase): Chi-squared approximation may be
## incorrect
```

```r
chi.age.bagpurchase.sim <- chisq.test(age.bagpurchase, simulate.p.value =
TRUE)
chi.age.bagpurchase.sim
```

```
##
##  Pearson's Chi-squared test with simulated p-value (based on 2000
##  replicates)
##
## data:  age.bagpurchase
## X-squared = 70.888, df = NA, p-value = 0.0004998
```

```r
chi.age.bagdisp <- chisq.test(age.bagdisp)
```

```
## Warning in chisq.test(age.bagdisp): Chi-squared approximation may be incorrect
```

```r
chi.age.bagdisp.sim <- chisq.test(age.bagdisp, simulate.p.value = TRUE)
chi.age.bagdisp.sim
```

```
##
##  Pearson's Chi-squared test with simulated p-value (based on 2000
##  replicates)
##
## data:  age.bagdisp
## X-squared = 28.893, df = NA, p-value = 0.01749
```

Simulated residuals for 'age':

```r
chi.age.barrier.sim$residuals
```

```
##
##               Council collection Unclear information Difficult transport
##   30 and under        -2.50149913          2.76177234         -0.34774676
##   31-50                0.33739293         -1.96820772          0.09747546
##   50+                  1.90103821         -0.17433654          0.20328055
##
##               No local facilities  No support Ends up in landfills  Forgetting
##   30 and under         -0.67036796 -0.29495031           0.17238143  2.58648936
##   31-50                 1.68183573  1.44976487           0.91326315 -0.60302269
##   50+                  -1.40987096 -1.47635325          -1.26048048 -1.65909091
##
##               Recycling a hassle Household disagrees Recycling not important
##   30 and under         0.88802716          1.50923532             -0.25337527
##   31-50               -0.19069252         -0.13483997              0.53935989
##   50+                 -0.58933356         -1.22983739             -0.41672176
##
##                     Other Already doing everything
##   30 and under -0.02955988              -0.95990143
##   31-50         0.18090681              -1.01437829
##   50+          -0.19090909               2.10901675
```

```
chi.age.barrier2.sim$residuals
```

```
##
##               Limited alternatives No SUP-free alternatives
##   30 and under          -1.07498024               0.45154251
##   31-50                 -0.73098799              -1.04651190
##   50+                    1.87340995               0.84553576
##
##               Alternatives expensive Limited functioning Forgetting reusables
##   30 and under             0.02404664          2.51105212          -0.40506502
##   31-50                    2.22383780         -1.00423346           1.28717450
##   50+                     -2.70423537         -1.10561212          -1.17866713
##
##               Reducing not important       Other No barriers
##   30 and under             1.44975662 -0.73853700 -1.65141893
##   31-50                   -0.30104712 -0.81016272 -0.38138504
##   50+                     -0.97450739  1.65848681  1.98361053
```

```
chi.age.mainconsid.sim$residuals
```

```
##
##               Value for money       Price     Quality Deals/discounts
##   30 and under      0.24523234  1.40513245 -1.81923138     -0.26860217
##   31-50            -0.87893609  1.27353569 -0.27641447      1.05253606
##   50+               0.83377895 -2.83235428  2.01183949     -1.02158678
##
##               Use-by-dates/longevity Convenience Ease of recycling packaging
##   30 and under             0.20803886  0.88802716                 -0.69954278
##   31-50                   -0.87028880 -1.62088640                  0.10285190
##   50+                      0.85766562  1.13554516                  0.52137266
##
##               Sustainability       Brand      Ethics       Other
```

```
##   30 and under    -0.18993041 -0.02955988  1.50923532 -0.71763500
##   31-50            1.37143690  0.93468517 -1.48323970 -0.93808315
##   50+             -1.47878030 -1.10000000  0.39639387  1.79347993
```

```
chi.age.litter.sim$residuals
```

```
##
##                 Strongly agree       Agree Neither agree nor disagree
##   30 and under      0.06779644 -0.26553269                 0.09541850
##   31-50            -1.15992824  1.38817355                -0.48042291
##   50+               1.33637530 -1.42921280                 0.49137620
##
##                   Disagree Strongly disagree
##   30 and under -0.04180398        0.95576940
##   31-50         0.78884265       -0.57287155
##   50+          -0.91281057       -0.19090909
```

```
chi.age.ocean.sim$residuals
```

```
##
##                     Always Most of the time   Sometimes      Rarely       Never
##   30 and under  1.85700107       2.16814124 -0.48771311 -2.40418325 -1.13633779
##   31-50        -0.47036584      -0.85148985  0.31980107  1.28452326 -0.90677781
##   50+          -1.14603587      -0.97344918  0.06428243  0.66896985  2.14203054
```

```
chi.age.actions.sim$residuals
```

```
##
##                 Yes, definitely Yes, probably No, probably not
##   30 and under    -0.589558283   0.401752534     -0.622833114
##   31-50            0.571831301  -0.004363319      0.018392020
##   50+             -0.145712399  -0.365405176      0.552460730
##
##                 No, definitely not I don't know
##   30 and under        1.287876287  0.356631929
##   31-50              -1.290909091 -0.719168129
##   50+                 0.368666144  0.538311327
```

```
chi.age.zerow.sim$residuals
```

```
##
##                 Yes, shops regularly Yes, shops occasionally
##   30 and under            0.2080389              -0.2608488
##   31-50                   1.5471801               1.8716384
##   50+                    -2.0579116              -2.0166149
##
##                 Yes, visited at least once Yes, never visited
##   30 and under                  -0.8769246          1.3318622
##   31-50                          1.6740443         -0.4371206
##   50+                           -1.2098995         -0.7016242
##
```

```
##                No, but would like to shop there No, not likely to shop there
##   30 and under                        0.8844962                   -2.3172360
##   31-50                              -0.7977240                   -1.1542669
##   50+                                 0.1460317                    3.5300415
```

```
chi.age.bottpurchase.sim$residuals
```

```
##
##                       None        1-2         3-5        6-10       11-15
##   30 and under -0.62007934 -0.72018010  0.08426829  0.21648476  0.04860076
##   31-50        -0.99224264 -1.58165495  0.91684078  0.95335951 -0.12450860
##   50+           1.76879128  2.57200489 -1.18349974 -1.34952941  0.10532268
##
##                        16+ I don't know
##   30 and under  1.26140016   0.88802716
##   31-50         0.74135713   0.28603878
##   50+          -2.05791156  -1.16429314
```

```
chi.age.bottdisp.sim$residuals
```

```
##
##                General waste bin Recycling bin Recycling centre
##   30 and under        0.39726795   -0.70292175       2.13438106
##   31-50               0.61507438   -0.02663709      -1.14415511
##   50+                -1.10833756    0.68065977      -0.58933356
##
##                Specialist waste collection    Landfill Deposit return scheme
##   30 and under                 -0.59773324 -0.25337527           -0.02955988
##   31-50                         1.09402153  1.21355975            0.93468517
##   50+                          -0.76795509 -1.22983739           -1.10000000
##
##                Indefinite storage       Other I don't know
##   30 and under         1.41887416 -0.71763500   1.46321398
##   31-50               -1.14574311 -0.93808315  -0.66332496
##   50+                  0.07272727  1.79347993  -0.55000000
```

```
chi.age.tubpurchase.sim$residuals
```

```
##
##                      None        1-2        3-5       6-10      11-15
##   30 and under  0.5176809 -0.7224778  0.3588406 -0.4034492  0.5608063
##   31-50        -0.5570485 -1.3810161  0.1361537  1.2427329  0.0671871
##   50+           0.1941996  2.3321452 -0.4952833 -1.1265593 -0.5984454
##
##                       16+ I don't know
##   30 and under  0.9031943   -0.3583267
##   31-50         0.1170739    0.7627701
##   50+          -0.9745074   -0.5893336
```

```
chi.age.tubdisp.sim$residuals
```

```
##
##                 General waste bin Recycling bin Recycling centre
##   30 and under       -1.09332061   -1.13043469       0.92674839
##   31-50               0.51370231    0.84688140      -1.05259792
##   50+                 0.38917828    0.02159162       0.41443823
##
##                 Specialist waste collection    Landfill Deposit return scheme
##   30 and under                   2.79320141  0.25884046           -0.02955988
##   31-50                         -0.55704850  0.59186403            0.93468517
##   50+                           -1.90525589 -0.95262794           -1.10000000
##
##                 Indefinite storage       Other I don't know
##   30 and under          1.27257152 -0.59773324   1.97509166
##   31-50                -1.50738442 -0.04558423  -1.00935023
##   50+                   0.64386580  0.60646118  -0.60494974
```

```
chi.age.filmpur.sim$residuals
```

```
##
##                        None         1-2         3-5        6-10       11-15
##   30 and under -0.62007934 -0.56549333 -0.43087233  0.16730903  0.93326254
##   31-50        -0.99224264 -1.63829972  0.18016386  1.03244237  0.20277791
##   50+           1.76879128  2.49760294  0.18024844 -1.39953609 -1.10561212
##
##                         16+ I don't know
##   30 and under  1.09851572  -0.05119921
##   31-50         0.38376129   0.74853392
##   50+          -1.47635325  -0.85552813
```

```
chi.age.filmreuse.sim$residuals
```

```
##
##                       Never        Once   2-4 times  5-10 times  More often
##   30 and under -1.25328530 -0.08057468  1.90268504  1.65544116  0.06996300
##   31-50        -0.86750088  0.57585595  0.86984654 -0.55704850  0.93419873
##   50+           2.20255967 -0.62016807 -2.80454204 -0.85552813 -1.19123579
```

```
chi.age.filmdisp.sim$residuals
```

```
##
##                 General waste bin Recycling bin Recycling centre
##   30 and under        -0.9972037     0.8855126        1.7313916
##   31-50               -0.8890413     0.8675986        0.5557670
##   50+                  1.9922708    -1.8633606       -2.2677081
##
##                 Specialist waste collection   Landfill Deposit return scheme
##   30 and under                    0.3660557  0.2588405            -0.7176350
##   31-50                           0.8370221  0.5918640             1.1939240
##   50+                            -1.3472194 -0.9526279            -0.7778175
##
##                 Indefinite storage      Other I don't know
##   30 and under           0.2588405 -0.4384623    0.3660557
```

```
##   31-50                 0.5918640  0.2215647   -1.0093502
##   50+                  -0.9526279  0.1373199    0.8795895
```

```
chi.age.bagpurchase.sim$residuals
```

```
##
##                        None         1-2         3-5        6-10       11-15
##   30 and under -2.42458256  1.38620391  2.98343824  0.95280092  0.20803886
##   31-50        -1.39358135  0.36422031  0.47819251  1.25892032  1.54718009
##   50+           3.91770648 -1.71821469 -3.32932106 -2.39739442 -2.05791156
##
##                         16+ I don't know
##   30 and under -0.73853700   0.89194461
##   31-50         1.85484623  -0.61538711
##   50+          -1.55563492  -0.08074696
```

```
chi.age.bagdisp.sim$residuals
```

```
##
##                General waste bin Recycling bin Recycling centre
##   30 and under        0.02925107   -0.55267491       1.29269049
##   31-50              -0.24205099    1.33911953      -0.21275915
##   50+                 0.26493667   -1.10512639      -0.93607332
##
##                Specialist waste collection    Landfill Deposit return scheme
##   30 and under                  0.65492903 -0.71763500           -0.49465145
##   31-50                         0.78884265  1.19392401            1.43636364
##   50+                          -1.55563492 -0.77781746           -1.27594119
##
##                Indefinite storage       Other I don't know
##   30 and under         0.35204107 -1.61179728   1.10521101
##   31-50               -1.74080592 -0.03772168  -0.48241815
##   50+                  1.77468851  1.53258094  -0.43787879
```

Simulated observed and expected values for 'age':

```
chi.age.barrier.sim$observed
```

```
##
##                Council collection Unclear information Difficult transport
##   30 and under                 16                  27                   7
##   31-50                        53                  17                  14
##   50+                          46                  18                  10
##
##                No local facilities No support Ends up in landfills Forgetting
##   30 and under                   7          4                    9         13
##   31-50                         22         12                   18          9
##   50+                            6          2                    6          3
##
##                Recycling a hassle Household disagrees Recycling not important
##   30 and under                  4                   3                       1
##   31-50                         4                   2                       3
```

```
##   50+                          2                 0                       1
##
##                Other Already doing everything
##   30 and under     1                       11
##   31-50            2                       20
##   50+              1                       26
```

```
chi.age.barrier.sim$expected
```

```
##
##                Council collection Unclear information Difficult transport
##   30 and under            29.6125              15.965              7.9825
##   31-50                   50.6000              27.280             13.6400
##   50+                     34.7875              18.755              9.3775
##
##                No local facilities No support Ends up in landfills Forgetting
##   30 and under              9.0125      4.635               8.4975     6.4375
##   31-50                    15.4000      7.920              14.5200    11.0000
##   50+                      10.5875      5.445               9.9825     7.5625
##
##                Recycling a hassle Household disagrees Recycling not important
##   30 and under              2.575              1.2875                  1.2875
##   31-50                     4.400              2.2000                  2.2000
##   50+                       3.025              1.5125                  1.5125
##
##                Other Already doing everything
##   30 and under  1.03                  14.6775
##   31-50         1.76                  25.0800
##   50+           1.21                  17.2425
```

```
chi.age.barrier2.sim$observed
```

```
##
##                Limited alternatives No SUP-free alternatives
##   30 and under                   18                       24
##   31-50                          35                       31
##   50+                            37                       30
##
##                Alternatives expensive Limited functioning Forgetting reusables
##   30 and under                     22                  18                    9
##   31-50                            51                  13                   23
##   50+                              12                   8                    8
##
##                Reducing not important Other No barriers
##   30 and under                      6     1           5
##   31-50                             5     2          16
##   50+                               2     5          19
```

```
chi.age.barrier2.sim$expected
```

```
##
##                Limited alternatives No SUP-free alternatives
```

```
## 30 and under              23.175               21.8875
## 31-50                     39.600               37.4000
## 50+                       27.225               25.7125
##
##              Alternatives expensive Limited functioning Forgetting reusables
## 30 and under               21.8875             10.0425                 10.3
## 31-50                      37.4000             17.1600                 17.6
## 50+                        25.7125             11.7975                 12.1
##
##              Reducing not important Other No barriers
## 30 and under                3.3475  2.06        10.3
## 31-50                       5.7200  3.52        17.6
## 50+                         3.9325  2.42        12.1
```

```
chi.age.mainconsid.sim$observed
```

```
##
##              Value for money Price Quality Deals/discounts
## 30 and under              32    32      14               6
## 31-50                     46    51      37              15
## 50+                       41    14      37               5
##
##              Use-by-dates/longevity Convenience Ease of recycling packaging
## 30 and under                      4           4                           4
## 31-50                             4           1                          10
## 50+                               6           5                           8
##
##              Sustainability Brand Ethics Other
## 30 and under              3     1      3     0
## 31-50                     9     3      0     0
## 50+                       1     0      2     2
```

```
chi.age.mainconsid.sim$expected
```

```
##
##              Value for money   Price Quality Deals/discounts
## 30 and under         30.6425 24.9775   22.66           6.695
## 31-50                52.3600 42.6800   38.72          11.440
## 50+                  35.9975 29.3425   26.62           7.865
##
##              Use-by-dates/longevity Convenience Ease of recycling packaging
## 30 and under                  3.605       2.575                       5.665
## 31-50                         6.160       4.400                       9.680
## 50+                           4.235       3.025                       6.655
##
##              Sustainability Brand Ethics Other
## 30 and under         3.3475  1.03 1.2875 0.515
## 31-50                5.7200  1.76 2.2000 0.880
## 50+                  3.9325  1.21 1.5125 0.605
```

```
chi.age.litter.sim$observed
```

```
##
##                Strongly agree Agree Neither agree nor disagree Disagree
##   30 and under             45    40                         14        2
##   31-50                    66    83                         21        5
##   50+                      62    39                         18        1
##
##                Strongly disagree
##   30 and under                 2
##   31-50                        1
##   50+                          1
```

```
chi.age.litter.sim$expected
```

```
##
##                Strongly agree  Agree Neither agree nor disagree Disagree
##   30 and under        44.5475 41.715                    13.6475     2.06
##   31-50               76.1200 71.280                    23.3200     3.52
##   50+                 52.3325 49.005                    16.0325     2.42
##
##                Strongly disagree
##   30 and under              1.03
##   31-50                     1.76
##   50+                       1.21
```

```
chi.age.ocean.sim$observed
```

```
##
##                Always Most of the time Sometimes Rarely Never
##   30 and under      9               38        48      6     2
##   31-50             7               40        91     33     5
##   50+               3               26        61     21    10
```

```
chi.age.ocean.sim$expected
```

```
##
##                Always Most of the time Sometimes Rarely  Never
##   30 and under 4.8925            26.78      51.5  15.45 4.3775
##   31-50        8.3600            45.76      88.0  26.40 7.4800
##   50+          5.7475            31.46      60.5  18.15 5.1425
```

```
chi.age.actions.sim$observed
```

```
##
##                Yes, definitely Yes, probably No, probably not
##   30 and under              33            52                9
##   31-50                     67            84               19
##   50+                       42            55               15
##
##                No, definitely not I don't know
##   30 and under                  5            4
##   31-50                         2            4
##   50+                           4            5
```

```r
chi.age.actions.sim$expected
```

```
##
##                 Yes, definitely Yes, probably No, probably not
##   30 and under           36.565       49.1825          11.0725
##   31-50                  62.480       84.0400          18.9200
##   50+                    42.955       57.7775          13.0075
##
##                 No, definitely not I don't know
##   30 and under              2.8325       3.3475
##   31-50                     4.8400       5.7200
##   50+                       3.3275       3.9325
```

```r
chi.age.zerow.sim$observed
```

```
##
##                 Yes, shops regularly Yes, shops occasionally
##   30 and under                     4                       7
##   31-50                           10                      20
##   50+                              0                       3
##
##                 Yes, visited at least once Yes, never visited
##   30 and under                           4                 29
##   31-50                                 16                 36
##   50+                                    4                 23
##
##                 No, but would like to shop there No, not likely to shop there
##   30 and under                                51                            8
##   31-50                                         70                           24
##   50+                                           54                           37
```

```r
chi.age.zerow.sim$expected
```

```
##
##                 Yes, shops regularly Yes, shops occasionally
##   30 and under                 3.605                   7.725
##   31-50                        6.160                  13.200
##   50+                          4.235                   9.075
##
##                 Yes, visited at least once Yes, never visited
##   30 and under                        6.18              22.66
##   31-50                              10.56              38.72
##   50+                                 7.26              26.62
##
##                 No, but would like to shop there No, not likely to shop there
##   30 and under                           45.0625                      17.7675
##   31-50                                  77.0000                      30.3600
##   50+                                    52.9375                      20.8725
```

```r
chi.age.bottpurchase.sim$observed
```

```
##
##                None 1-2 3-5 6-10 11-15 16+ I don't know
##   30 and under    2  31  35   20     5   6            4
##   31-50           3  48  66   38     8   8            5
##   50+             7  58  33   16     6   0            1
```

```r
chi.age.bottpurchase.sim$expected
```

```
##
##                None     1-2    3-5   6-10  11-15   16+ I don't know
##   30 and under 3.09 35.2775 34.505 19.055 4.8925 3.605        2.575
##   31-50        5.28 60.2800 58.960 32.560 8.3600 6.160        4.400
##   50+          3.63 41.4425 40.535 22.385 5.7475 4.235        3.025
```

```r
chi.age.bottdisp.sim$observed
```

```
##
##                General waste bin Recycling bin Recycling centre
##   30 and under                 8            78                6
##   31-50                       14           144                2
##   50+                          5           106                2
##
##                Specialist waste collection Landfill Deposit return scheme
##   30 and under                           1        1                     1
##   31-50                                  5        4                     3
##   50+                                    1        0                     0
##
##                Indefinite storage Other I don't know
##   30 and under                  7     0            1
##   31-50                         4     0            0
##   50+                           5     2            0
```

```r
chi.age.bottdisp.sim$expected
```

```
##
##                General waste bin Recycling bin Recycling centre
##   30 and under            6.9525         84.46            2.575
##   31-50                  11.8800        144.32            4.400
##   50+                     8.1675         99.22            3.025
##
##                Specialist waste collection Landfill Deposit return scheme
##   30 and under                      1.8025   1.2875                  1.03
##   31-50                             3.0800   2.2000                  1.76
##   50+                               2.1175   1.5125                  1.21
##
##                Indefinite storage Other I don't know
##   30 and under               4.12 0.515       0.2575
##   31-50                      7.04 0.880       0.4400
##   50+                        4.84 0.605       0.3025
```

```r
chi.age.tubpurchase.sim$observed
```

```
##
##               None 1-2 3-5 6-10 11-15 16+ I don't know
##   30 and under    4  24  39   20     9   5            2
##   31-50           4  38  64   45    13   6            6
##   50+             4  46  40   20     7   2            2
```

```r
chi.age.tubpurchase.sim$expected
```

```
##
##               None   1-2     3-5    6-10   11-15    16+ I don't know
##   30 and under 3.09 27.81 36.8225 21.8875  7.4675 3.3475        2.575
##   31-50        5.28 47.52 62.9200 37.4000 12.7600 5.7200        4.400
##   50+          3.63 32.67 43.2575 25.7125  8.7725 3.9325        3.025
```

```r
chi.age.tubdisp.sim$observed
```

```
##
##               General waste bin Recycling bin Recycling centre
##   30 and under                17            46               10
##   31-50                       41           101                9
##   50+                         28            64               10
##
##               Specialist waste collection Landfill Deposit return scheme
##   30 and under                           8        1                      1
##   31-50                                  4        2                      3
##   50+                                    0        0                      0
##
##               Indefinite storage Other I don't know
##   30 and under                 15     1            4
##   31-50                        12     3            1
##   50+                          15     3            1
```

```r
chi.age.tubdisp.sim$expected
```

```
##
##               General waste bin Recycling bin Recycling centre
##   30 and under            22.145       54.3325           7.4675
##   31-50                   37.840       92.8400          12.7600
##   50+                     26.015       63.8275           8.7725
##
##               Specialist waste collection Landfill Deposit return scheme
##   30 and under                        3.09   0.7725                   1.03
##   31-50                               5.28   1.3200                   1.76
##   50+                                 3.63   0.9075                   1.21
##
##               Indefinite storage  Other I don't know
##   30 and under             10.815 1.8025        1.545
##   31-50                    18.480 3.0800        2.640
##   50+                      12.705 2.1175        1.815
```

```r
chi.age.filmpur.sim$observed
```

```
##
##                None 1-2 3-5 6-10 11-15 16+ I don't know
##   30 and under    2  19  29   30    13   7            3
##   31-50           3  27  55   57    18   9            7
##   50+             7  38  38   26     8   2            2
```

```r
chi.age.filmpur.sim$expected
```

```
##
##                None   1-2    3-5    6-10   11-15   16+ I don't know
##   30 and under 3.09 21.63 31.415 29.0975 10.0425 4.635         3.09
##   31-50        5.28 36.96 53.680 49.7200 17.1600 7.920         5.28
##   50+          3.63 25.41 36.905 34.1825 11.7975 5.445         3.63
```

```r
chi.age.filmreuse.sim$observed
```

```
##
##                Never Once 2-4 times 5-10 times More often
##   30 and under    51   21        21          6          4
##   31-50           95   40        28          4          9
##   50+             90   22         5          2          2
```

```r
chi.age.filmreuse.sim$expected
```

```
##
##                 Never    Once 2-4 times 5-10 times More often
##   30 and under  60.77 21.3725    13.905       3.09     3.8625
##   31-50        103.84 36.5200    23.760       5.28     6.6000
##   50+           71.39 25.1075    16.335       3.63     4.5375
```

```r
chi.age.filmdisp.sim$observed
```

```
##
##                General waste bin Recycling bin Recycling centre
##   30 and under                60            28                8
##   31-50                      107            46                9
##   50+                         98            18                0
##
##                Specialist waste collection Landfill Deposit return scheme
##   30 and under                           2        1                     0
##   31-50                                  4        2                     2
##   50+                                    0        0                     0
##
##                Indefinite storage Other I don't know
##   30 and under                  1     1            2
##   31-50                         2     3            1
##   50+                           0     2            3
```

```r
chi.age.filmdisp.sim$expected
```

```
##
##                General waste bin Recycling bin Recycling centre
##   30 and under           68.2375         23.69           4.3775
##   31-50                 116.6000         40.48           7.4800
##   50+                    80.1625         27.83           5.1425
##
##                Specialist waste collection Landfill Deposit return scheme
##   30 and under                       1.545   0.7725                 0.515
##   31-50                              2.640   1.3200                 0.880
##   50+                                1.815   0.9075                 0.605
##
##                Indefinite storage Other I don't know
##   30 and under             0.7725 1.545        1.545
##   31-50                    1.3200 2.640        2.640
##   50+                      0.9075 1.815        1.815
```

```r
chi.age.bagpurchase.sim$observed
```

```
##
##                None 1-2 3-5 6-10 11-15 16+ I don't know
##   30 and under   41  26  21    7     4   1            3
##   31-50          88  36  21   12    10   7            2
##   50+           103  15   1    0     0   0            2
```

```r
chi.age.bagpurchase.sim$expected
```

```
##
##                  None     1-2     3-5   6-10 11-15  16+ I don't know
##   30 and under  59.74 19.8275 11.0725 4.8925 3.605 2.06       1.8025
##   31-50        102.08 33.8800 18.9200 8.3600 6.160 3.52       3.0800
##   50+           70.18 23.2925 13.0075 5.7475 4.235 2.42       2.1175
```

```r
chi.age.bagdisp.sim$observed
```

```
##
##                General waste bin Recycling bin Recycling centre
##   30 and under                37            21               11
##   31-50                       61            49               12
##   50+                         45            22                6
##
##                Specialist waste collection Landfill Deposit return scheme
##   30 and under                           3        0                     2
##   31-50                                  5        2                     8
##   50+                                    0        0                     1
##
##                Indefinite storage Other I don't know
##   30 and under                 23     2            4
##   31-50                        26    10            3
##   50+                          34    11            2
```

```r
chi.age.bagdisp.sim$expected
```

```
##
##                  General waste bin Recycling bin Recycling centre
##   30 and under             36.8225         23.69           7.4675
##   31-50                    62.9200         40.48          12.7600
##   50+                      43.2575         27.83           8.7725
##
##                  Specialist waste collection Landfill Deposit return scheme
##   30 and under                          2.06    0.515                2.8325
##   31-50                                 3.52    0.880                4.8400
##   50+                                   2.42    0.605                3.3275
##
##                  Indefinite storage   Other I don't know
##   30 and under              21.3725  5.9225       2.3175
##   31-50                     36.5200 10.1200       3.9600
##   50+                       25.1075  6.9575       2.7225
```

Education

```r
chi.edu.choice <- chisq.test(edu.choice)
```

```
## Warning in chisq.test(edu.choice): Chi-squared approximation may be incorrect
```

```r
chi.edu.choice.sim <- chisq.test(edu.choice, simulate.p.value = TRUE)
chi.edu.choice.sim
```

```
##
##  Pearson's Chi-squared test with simulated p-value (based on 2000
##  replicates)
##
## data:  edu.choice
## X-squared = 15.809, df = NA, p-value = 0.1974
```

```r
chi.edu.barrier <- chisq.test(edu.barrier)
```

```
## Warning in chisq.test(edu.barrier): Chi-squared approximation may be incorrect
```

```r
chi.edu.barrier.sim <- chisq.test(edu.barrier, simulate.p.value = TRUE)
chi.edu.barrier.sim
```

```
##
##  Pearson's Chi-squared test with simulated p-value (based on 2000
##  replicates)
##
## data:  edu.barrier
## X-squared = 46.267, df = NA, p-value = 0.3098
```

```r
chi.edu.barrier2 <- chisq.test(edu.barrier2)
```

```
## Warning in chisq.test(edu.barrier2): Chi-squared approximation may be incorrect
```

```r
chi.edu.barrier2.sim <- chisq.test(edu.barrier2, simulate.p.value = TRUE)
chi.edu.barrier2.sim
```

```
##
##  Pearson's Chi-squared test with simulated p-value (based on 2000
##  replicates)
##
## data:  edu.barrier2
## X-squared = 34.124, df = NA, p-value = 0.1994
```

```r
chi.edu.mainconsid <- chisq.test(edu.mainconsid)
```

```
## Warning in chisq.test(edu.mainconsid): Chi-squared approximation may be
## incorrect
```

```r
chi.edu.mainconsid.sim <- chisq.test(edu.mainconsid, simulate.p.value =
TRUE)
chi.edu.mainconsid.sim
```

```
##
##  Pearson's Chi-squared test with simulated p-value (based on 2000
##  replicates)
##
## data:  edu.mainconsid
## X-squared = 53.357, df = NA, p-value = 0.1144
```

```r
chi.edu.litter <- chisq.test(edu.litter)
```

```
## Warning in chisq.test(edu.litter): Chi-squared approximation may be incorrect
```

```r
chi.edu.litter.sim <- chisq.test(edu.litter, simulate.p.value = TRUE)
chi.edu.litter.sim
```

```
##
##  Pearson's Chi-squared test with simulated p-value (based on 2000
##  replicates)
##
## data:  edu.litter
## X-squared = 12.056, df = NA, p-value = 0.6517
```

```r
chi.edu.attitude <- chisq.test(edu.attitude)
```

```
## Warning in chisq.test(edu.attitude): Chi-squared approximation may be incorrect
```

```r
chi.edu.attitude.sim <- chisq.test(edu.attitude, simulate.p.value = TRUE)
chi.edu.attitude.sim
```

```
##
##  Pearson's Chi-squared test with simulated p-value (based on 2000
##  replicates)
##
## data:  edu.attitude
## X-squared = 32.012, df = NA, p-value = 0.1254
```

```r
chi.edu.ocean <- chisq.test(edu.ocean)
```

```
## Warning in chisq.test(edu.ocean): Chi-squared approximation may be incorrect
```

```r
chi.edu.ocean.sim <- chisq.test(edu.ocean, simulate.p.value = TRUE)
chi.edu.ocean.sim
```

```
##
##  Pearson's Chi-squared test with simulated p-value (based on 2000
##  replicates)
##
## data:  edu.ocean
## X-squared = 11.53, df = NA, p-value = 0.7321
```

```r
chi.edu.actions <- chisq.test(edu.actions)
```

```
## Warning in chisq.test(edu.actions): Chi-squared approximation may be incorrect
```

```r
chi.edu.actions.sim <- chisq.test(edu.actions, simulate.p.value = TRUE)
chi.edu.actions.sim
```

```
##
##  Pearson's Chi-squared test with simulated p-value (based on 2000
##  replicates)
##
## data:  edu.actions
## X-squared = 13.906, df = NA, p-value = 0.5242
```

```r
chi.edu.attplast <- chisq.test(edu.attplast)
```

```
## Warning in chisq.test(edu.attplast): Chi-squared approximation may be incorrect
```

```r
chi.edu.attplast.sim <- chisq.test(edu.attplast, simulate.p.value = TRUE)
chi.edu.attplast.sim
```

```
##
##  Pearson's Chi-squared test with simulated p-value (based on 2000
##  replicates)
##
## data:  edu.attplast
## X-squared = 11.984, df = NA, p-value = 0.4368
```

```r
chi.edu.zerow <- chisq.test(edu.zerow)
```

```
## Warning in chisq.test(edu.zerow): Chi-squared approximation may be incorrect
```

```r
chi.edu.zerow.sim <- chisq.test(edu.zerow, simulate.p.value = TRUE)
chi.edu.zerow.sim
```

```
##
##  Pearson's Chi-squared test with simulated p-value (based on 2000
##  replicates)
##
## data:  edu.zerow
## X-squared = 35.131, df = NA, p-value = 0.02999
```

```r
chi.edu.bottpurchase <- chisq.test(edu.bottpurchase)
```

```
## Warning in chisq.test(edu.bottpurchase): Chi-squared approximation may be
## incorrect
```

```r
chi.edu.bottpurchase.sim <- chisq.test(edu.bottpurchase,simulate.p.value =
TRUE)
chi.edu.bottpurchase.sim
```

```
##
##  Pearson's Chi-squared test with simulated p-value (based on 2000
##  replicates)
##
## data:  edu.bottpurchase
## X-squared = 19.015, df = NA, p-value = 0.6862
```

```r
chi.edu.bottreuse <- chisq.test(edu.bottreuse)
```

```
## Warning in chisq.test(edu.bottreuse): Chi-squared approximation may be incorrect
```

```r
chi.edu.bottreuse.sim <- chisq.test(edu.bottreuse, simulate.p.value = TRUE)
chi.edu.bottreuse.sim
```

```
##
##  Pearson's Chi-squared test with simulated p-value (based on 2000
##  replicates)
##
## data:  edu.bottreuse
## X-squared = 17.437, df = NA, p-value = 0.3333
```

```r
chi.edu.bottdisp <- chisq.test(edu.bottdisp)
```

```
## Warning in chisq.test(edu.bottdisp): Chi-squared approximation may be incorrect
```

```r
chi.edu.bottdisp.sim <- chisq.test(edu.bottdisp, simulate.p.value = TRUE)
chi.edu.bottdisp.sim
```

```
##
##  Pearson's Chi-squared test with simulated p-value (based on 2000
##  replicates)
##
## data:  edu.bottdisp
## X-squared = 72.654, df = NA, p-value = 0.03298
```

```r
chi.edu.tubspurchase <- chisq.test(edu.tubspurchase)
```

```
## Warning in chisq.test(edu.tubspurchase): Chi-squared approximation may be
## incorrect
```

```r
chi.edu.tubspurchase.sim <- chisq.test(edu.tubspurchase, simulate.p.value =
TRUE)
chi.edu.tubspurchase.sim
```

```
##
##  Pearson's Chi-squared test with simulated p-value (based on 2000
##  replicates)
##
## data:  edu.tubspurchase
## X-squared = 23.149, df = NA, p-value = 0.4703
```

```r
chi.edu.tubsreuse <- chisq.test(edu.tubsreuse)
```

```
## Warning in chisq.test(edu.tubsreuse): Chi-squared approximation may be incorrect
```

```r
chi.edu.tubsreuse.sim <- chisq.test(edu.tubsreuse, simulate.p.value = TRUE)
chi.edu.tubsreuse.sim
```

```
##
##  Pearson's Chi-squared test with simulated p-value (based on 2000
##  replicates)
##
## data:  edu.tubsreuse
## X-squared = 20.014, df = NA, p-value = 0.1929
```

```r
chi.edu.tubdisp <- chisq.test(edu.tubdisp)
```

```
## Warning in chisq.test(edu.tubdisp): Chi-squared approximation may be incorrect
```

```r
chi.edu.tubdisp.sim <- chisq.test(edu.tubdisp, simulate.p.value = TRUE)
chi.edu.tubdisp.sim
```

```
##
##  Pearson's Chi-squared test with simulated p-value (based on 2000
##  replicates)
##
## data:  edu.tubdisp
## X-squared = 31.983, df = NA, p-value = 0.3538
```

```
chi.edu.filmpurchase <- chisq.test(edu.filmpurchase)
```

```
## Warning in chisq.test(edu.filmpurchase): Chi-squared approximation may be
## incorrect
```

```
chi.edu.filmpurchase.sim <- chisq.test(edu.filmpurchase, simulate.p.value =
TRUE)
chi.edu.filmpurchase.sim
```

```
##
##  Pearson's Chi-squared test with simulated p-value (based on 2000
##  replicates)
##
## data:  edu.filmpurchase
## X-squared = 14.176, df = NA, p-value = 0.913
```

```
chi.edu.filmreuse <- chisq.test(edu.filmreuse)
```

```
## Warning in chisq.test(edu.filmreuse): Chi-squared approximation may be incorrect
```

```
chi.edu.filmreuse.sim <- chisq.test(edu.filmreuse, simulate.p.value = TRUE)
chi.edu.filmreuse.sim
```

```
##
##  Pearson's Chi-squared test with simulated p-value (based on 2000
##  replicates)
##
## data:  edu.filmreuse
## X-squared = 38.951, df = NA, p-value = 0.007996
```

```
chi.edu.filmdisp <- chisq.test(edu.filmdisp)
```

```
## Warning in chisq.test(edu.filmdisp): Chi-squared approximation may be incorrect
```

```
chi.edu.filmdisp.sim <- chisq.test(edu.filmdisp, simulate.p.value = TRUE)
chi.edu.filmdisp.sim
```

```
##
##  Pearson's Chi-squared test with simulated p-value (based on 2000
##  replicates)
##
## data:  edu.filmdisp
## X-squared = 70.19, df = NA, p-value = 0.05047
```

```
chi.edu.bagpurchase <- chisq.test(edu.bagpurchase)
```

```
## Warning in chisq.test(edu.bagpurchase): Chi-squared approximation may be
## incorrect
```

```
chi.edu.bagpurchase.sim <- chisq.test(edu.bagpurchase, simulate.p.value =
TRUE)
chi.edu.bagpurchase.sim
```

```
##
##  Pearson's Chi-squared test with simulated p-value (based on 2000
##  replicates)
##
## data:  edu.bagpurchase
## X-squared = 17.299, df = NA, p-value = 0.7381
```

```
chi.edu.bagreuse <- chisq.test(edu.bagreuse)
```

```
## Warning in chisq.test(edu.bagreuse): Chi-squared approximation may be incorrect
```

```
chi.edu.bagreuse.sim <- chisq.test(edu.bagreuse, simulate.p.value = TRUE)
chi.edu.bagreuse.sim
```

```
##
##  Pearson's Chi-squared test with simulated p-value (based on 2000
##  replicates)
##
## data:  edu.bagreuse
## X-squared = 7.7961, df = NA, p-value = 0.965
```

```
chi.edu.bagdisp <- chisq.test(edu.bagdisp)
```

```
## Warning in chisq.test(edu.bagdisp): Chi-squared approximation may be incorrect
```

```
chi.edu.bagdisp.sim <- chisq.test(edu.bagdisp, simulate.p.value = TRUE)
chi.edu.bagdisp.sim
```

```
##
##  Pearson's Chi-squared test with simulated p-value (based on 2000
##  replicates)
##
## data:  edu.bagdisp
## X-squared = 37.543, df = NA, p-value = 0.2319
```

Simulated residuals for 'education'

```
chi.edu.choice.sim$residuals
```

```
##
##                    Always As often as they can If cheaper or preferred
##   Primary       1.60556099         -0.03922323            -0.76157731
##   Secondary    -1.24404543         -0.06195340             0.54112429
##   High school  -1.53885029         -0.07297564             0.86318760
##   Undergraduate 1.89712258          0.09548198            -1.14272087
##   Postgraduate  0.91414013          0.06471502            -0.13617658
##
##                Rarely or never
##   Primary          -0.38729833
##   Secondary         0.63954720
##   High school       0.40032038
##   Undergraduate    -0.35355339
##   Postgraduate     -1.03458705
```

```
chi.edu.barrier.sim$residuals
```

```
##
##                Council collection Unclear information Difficult transport
##   Primary            0.5604734024       -0.5567764363       -0.3937003937
##   Secondary         -1.1148748756        1.2804644828       -0.1887442091
##   High school       -0.0613395615        0.6349029351       -0.6537257224
##   Undergraduate      0.8326688172       -1.5138646376        1.0936508958
##   Postgraduate       0.2020427085       -0.2455335789       -0.4729606287
##
##                No local facilities    No support Ends up in landfills
##   Primary             1.9721272054 -0.3000000000        -0.4062019202
##   Secondary           0.1759154470 -1.6106194692        -0.0008837454
##   High school        -1.0006865775 -0.3514317480        -0.5267329043
##   Undergraduate       0.9274260335  0.7810117950         0.5580876639
##   Postgraduate       -0.6479515953  1.8699045991         0.0174077656
##
##                   Forgetting Recycling a hassle Household disagrees
##   Primary       -0.3535533906      -0.2236067977       -0.1581138830
##   Secondary     -0.4315221202       0.3692427470       -1.1011357773
##   High school   -0.0438529010      -0.1386750491        0.2941742027
##   Undergraduate -0.1506160190      -0.2041241452        0.2405626122
##   Postgraduate   1.1666666667       0.1054092553        0.8198915917
##
##                Recycling not important         Other Already doing everything
##   Primary                -0.1581138830 -0.1414213562            -0.5338539126
##   Secondary              -0.1929825589  0.0304603850             1.6615731084
##   High school            -0.4902903378  1.4909986328             1.0397140944
##   Undergraduate          -0.5292377468 -1.1618950039            -1.4221199936
##   Postgraduate            2.3106035767 -0.6000000000            -1.8234395449
```

```
chi.edu.barrier2.sim$residuals
```

```
##
##                Limited alternatives No SUP-free alternatives
##   Primary                2.31060358              -0.65192024
##   Secondary              0.03745941              -1.67672551
##   High school            1.06317538               0.26160832
```

```
##   Undergraduate          -1.33814717              0.43175303
##   Postgraduate           -0.03513642              1.57274582
##
##                  Alternatives expensive Limited functioning Forgetting reusables
##   Primary                   -0.65192024         -0.44158804          -0.44721360
##   Secondary                  0.30561007         -0.79910777           0.09632419
##   High school               -0.87995526         -0.18959627           0.83205029
##   Undergraduate              0.99186506          0.78210791          -0.13608276
##   Postgraduate              -0.59655876          0.26154265          -1.37032032
##
##                  Reducing not important       Other No barriers
##   Primary                   -0.25495098 -0.20000000 -0.44721360
##   Secondary                  1.04053549  0.76103565  1.70172744
##   High school               -1.08247197 -0.99227788 -0.27735010
##   Undergraduate             -0.66240626  0.79115481 -0.68041382
##   Postgraduate               1.69183560 -0.84852814 -0.84327404
```

```
chi.edu.mainconsid.sim$residuals
```

```
##
##                  Value for money       Price     Quality Deals/discounts
##   Primary            -0.77136243 -0.69641941 -0.66332496     -0.36055513
##   Secondary          -0.34577968  0.71701031 -0.72301825      0.27678481
##   High school         0.21305941 -0.44971134  0.26178516     -0.49881516
##   Undergraduate       0.13215221  0.22065239 -0.31193973      0.41353524
##   Postgraduate        0.08861419 -0.58551629  1.44976487     -0.22226495
##
##                  Use-by-dates/longevity Convenience Ease of recycling packaging
##   Primary                    3.51506960 -0.22360680                 -0.33166248
##   Secondary                 -0.75710187  0.36924275                  0.28790846
##   High school                1.14857772 -0.13867505                  1.06583957
##   Undergraduate             -0.79357539  0.34020691                 -0.88994571
##   Postgraduate              -0.23162641 -0.94868330                 -0.69645567
##
##                  Sustainability       Brand      Ethics       Other
##   Primary            3.66737173 -0.14142136 -0.15811388 -0.10000000
##   Secondary         -0.64910266  1.04580655 -0.19298256  2.17541322
##   High school       -1.56897622 -0.26311741  0.29417420 -0.80622577
##   Undergraduate      1.72464332 -0.30123204 -0.52923775 -0.82158384
##   Postgraduate      -0.15716506 -0.60000000  0.81989159 -0.42426407
```

```
chi.edu.litter.sim$residuals
```

```
##
##                  Strongly agree        Agree Neither agree nor disagree
##   Primary          -0.930053762  1.322222222               -0.514781507
##   Secondary         0.007333557 -0.364563198                1.156891389
##   High school       0.103356304  0.186052102               -0.295159301
##   Undergraduate    -0.312452094  0.043953045               -0.446284843
##   Postgraduate      0.615831596 -0.151897012               -0.352558781
##
##                      Disagree Strongly disagree
##   Primary        -0.200000000      -0.141421356
```

```
##   Secondary     -0.674880669      -0.984885780
##   High school   -0.372104204      -0.263117406
##   Undergraduate  1.399735425       1.420093894
##   Postgraduate  -0.848528137      -0.600000000
```

```
chi.edu.attitude.sim$residuals
```

```
##
##                 1 (Not concerned)           2           3           4
##   Primary             -0.36742346 -0.31622777  1.75586126 -0.60415230
##   Secondary            0.95845397  1.43034132 -0.93318698 -0.64231550
##   High school         -0.59920413 -2.15727749  1.55464866  0.87767355
##   Undergraduate       -0.36853713  1.25092558  0.04368422  0.07303141
##   Postgraduate         0.36565517 -0.59628479 -1.92093727 -0.61251534
##
##                           5           6 7 (Very concerned
##   Primary       -0.75498344  1.36067210       -0.58736701
##   Secondary     -0.50305721 -0.42875303        1.04326134
##   High school    0.48464966 -0.51568795       -0.72325984
##   Undergraduate -0.39901194 -0.20701967        0.14764659
##   Postgraduate   0.85541504  1.76392420       -0.48555627
```

```
chi.edu.ocean.sim$residuals
```

```
##
##                     Always Most of the time  Sometimes     Rarely      Never
##   Primary       -0.3082207        0.6656402  0.0000000 -0.5477226 -0.2915476
##   Secondary      0.1828551       -1.0394365  0.2153874  0.3801338  0.9246974
##   High school    0.7344198       -0.4816146  0.8682431 -0.5661385 -1.4996606
##   Undergraduate -0.5577952        0.9958604 -0.6694387  0.1666667  0.1095893
##   Postgraduate  -0.5429506        0.5360508 -0.7071068  0.2581989  1.1884246
```

```
chi.edu.actions.sim$residuals
```

```
##
##                 Yes, definitely Yes, probably No, probably not
##   Primary           1.530948339  -0.977241014     -0.463680925
##   Secondary        -0.755776842  -0.046652097      1.416001179
##   High school      -0.169282470  -0.009519254      0.274187693
##   Undergraduate     1.021987028  -0.306706279     -0.922030350
##   Postgraduate     -0.777641392   0.918939749     -0.950574422
##
##                 No, definitely not I don't know
##   Primary             -0.234520788 -0.254950976
##   Secondary           -0.408694747  0.477322778
##   High school          0.753662387 -0.595967713
##   Undergraduate       -0.888786295  0.292413574
##   Postgraduate         1.015088193 -0.157165056
```

```
chi.edu.attplast.sim$residuals
```

```
##
##                  Will go out of their way to avoid
##   Primary                              -0.71414284
##   Secondary                            -1.55526486
##   High school                           0.32131423
##   Undergraduate                         0.26843775
##   Postgraduate                          1.59083705
##
##                  If option is readily available, will avoid
##   Primary                                       0.87162192
##   Secondary                                     0.15610318
##   High school                                   0.01782056
##   Undergraduate                                 0.28271354
##   Postgraduate                                 -1.04301961
##
##                  Will avoid only without extra costs Not a priority
##   Primary                                -0.53851648    -0.33166248
##   Secondary                               1.31588304     0.72085352
##   High school                            -0.65643115     0.31788198
##   Undergraduate                          -0.58200451    -0.52295779
##   Postgraduate                            0.34139673    -0.69645567
```

```
chi.edu.zerow.sim$residuals
```

```
##
##                  Yes, shops regularly Yes, shops occasionally
##   Primary                  3.51506960             -0.38729833
##   Secondary               -0.21437651             -1.21421280
##   High school             -0.72665121             -0.88070485
##   Undergraduate           -0.33353169              1.21779501
##   Postgraduate             1.55011520              1.39973542
##
##                  Yes, visited at least once Yes, never visited
##   Primary                        -0.34641016        -0.66332496
##   Secondary                      -1.16892761        -0.93949078
##   High school                     0.07161149         0.07479576
##   Undergraduate                   0.31622777         1.52299988
##   Postgraduate                    1.25196142        -1.39291135
##
##                  No, but would like to shop there No, not likely to shop there
##   Primary                              0.13363062                 -0.58736701
##   Secondary                            0.85387663                  1.28772797
##   High school                          0.41437097                  0.12142318
##   Undergraduate                       -1.04909258                 -0.88846986
##   Postgraduate                        -0.18898224                 -0.48555627
```

```
chi.edu.bottpurchase.sim$residuals
```

```
##
##                         None         1-2         3-5        6-10       11-15
##   Primary        -0.24494897 -0.82764727  0.40315917  1.03571362 -0.30822070
##   Secondary      -0.53345145  0.48187890 -0.61311072  0.72117304  0.64872783
##   High school     0.55700665 -0.07867873  0.06818965 -0.41801929 -0.07042382
```

```
##   Undergraduate -0.52174919 -0.03492741  0.56134217 -0.39519765 -0.16289594
##   Postgraduate   0.88527041 -0.37876523 -0.30523351  0.13174732 -0.54295057
##
##                         16+ I don't know
##   Primary       -0.26457513  -0.22360680
##   Secondary     -1.29982722  -0.27291855
##   High school   -0.72665121   1.52542554
##   Undergraduate  0.58655573  -0.74845520
##   Postgraduate   2.44098601  -0.94868330
```

```
chi.edu.bottreuse.sim$residuals
```

```
##
##                       Never        Once   2-4 times  5-10 times  More often
##   Primary        1.08254395 -0.57445626  0.44202669 -0.53385391 -0.62048368
##   Secondary      1.39357580  0.49867209 -1.48084880  1.66157311 -1.31272353
##   High school   -0.01561315  0.11875422 -0.45174082 -0.12197763  0.59470272
##   Undergraduate -1.01375838 -0.27014773  1.43408254 -0.96612961  0.19861534
##   Postgraduate  -0.54987550 -0.38568667  0.40794225 -0.49890719  0.78632798
```

```
chi.edu.bottdisp.sim$residuals
```

```
##
##                 General waste bin Recycling bin Recycling centre
##   Primary             -0.36742346   -0.49975604      -0.22360680
##   Secondary            0.17684013    0.72433542      -0.91507985
##   High school         -0.26162434   -0.15496777      -0.13867505
##   Undergraduate       -0.03726780   -0.25661968       0.88453796
##   Postgraduate         0.36565517   -0.27975987       0.10540926
##
##                 Specialist waste collection    Landfill Deposit return scheme
##   Primary                       -0.18708287 -0.15811388            6.92964646
##   Secondary                     -0.53535187 -0.19298256           -0.98488578
##   High school                   -0.18232322 -1.27475488            0.61394061
##   Undergraduate                 -0.23584252  1.78016333           -0.30123204
##   Postgraduate                   1.72603776 -0.67082039           -0.60000000
##
##                 Indefinite storage       Other I don't know
##   Primary              -0.28284271 -0.10000000  -0.07071068
##   Secondary            -1.96977156 -0.69641941   1.53824944
##   High school           1.66641024  0.43412157  -0.57008771
##   Undergraduate        -0.17213259  0.39557740  -0.58094750
##   Postgraduate          0.46666667 -0.42426407  -0.30000000
```

```
chi.edu.tubspurchase.sim$residuals
```

```
##
##                        None         1-2         3-5        6-10       11-15
##   Primary       -0.24494897 -0.73484692  1.51967286 -0.65192024 -0.38078866
##   Secondary      1.22517970  0.35368026  1.07365549 -1.89698502  0.36483467
##   High school    0.05063697 -0.01687899 -0.50973577  0.83239011  0.18729542
##   Undergraduate -0.52174919  0.25673373 -0.75750796  0.24504901 -0.25171823
```

```
##   Postgraduate  -1.03923048 -0.87244041  0.31498449  1.21119506 -0.37758052
##
##                         16+ I don't know
##   Primary       -0.25495098  -0.22360680
##   Secondary     -1.21231537  -0.27291855
##   High school   -1.08247197   0.41602515
##   Undergraduate  1.72464332   0.34020691
##   Postgraduate   0.76733527  -0.94868330
```

```
chi.edu.tubsreuse.sim$residuals
```

```
##
##                       Never        Once   2-4 times  5-10 times  More often
##   Primary       -0.55226805 -0.45276926  0.73949690 -0.53851648  0.33704807
##   Secondary      0.83396126  1.28679904  0.09845361 -0.01733179 -1.30375484
##   High school   -1.75742884 -0.08903274  0.08459916 -0.42610443  1.39718941
##   Undergraduate  1.63366461 -1.30044551  0.04587822 -0.35598334 -0.18172994
##   Postgraduate  -1.06270610  0.68195876 -0.58551629  1.65446105 -0.24251018
```

```
chi.edu.tubdisp.sim$residuals
```

```
##
##                 General waste bin Recycling bin Recycling centre
##   Primary             -0.65574385    0.92003760      -0.38078866
##   Secondary            0.68867696    0.53577823      -1.52061582
##   High school         -1.12544962   -0.06943604      -0.13843575
##   Undergraduate        0.73782101   -0.38068435       0.70720837
##   Postgraduate        -0.26598751   -0.22718137       1.47937286
##
##                 Specialist waste collection    Landfill Deposit return scheme
##   Primary                       -0.24494897 -0.12247449           -0.14142136
##   Secondary                      0.05275893  0.31948466           -0.98488578
##   High school                    0.55700665 -0.98742088            0.61394061
##   Undergraduate                 -0.52174919 -0.01242260            0.55943093
##   Postgraduate                  -0.07698004  1.40488566           -0.60000000
##
##                 Indefinite storage       Other I don't know
##   Primary              -0.45825757 -0.18708287  -0.17320508
##   Secondary            -0.37131102  0.23217769  -1.20623381
##   High school           1.44806300 -1.50831031   2.18415037
##   Undergraduate        -1.10890654  1.71595763  -0.72029658
##   Postgraduate          0.11315579 -0.79372539  -0.73484692
```

```
chi.edu.filmpurchase.sim$residuals
```

```
##
##                        None         1-2         3-5        6-10       11-15
##   Primary       -0.24494897  0.89495943 -0.78102497  0.57871539 -0.44158804
##   Secondary      0.05275893 -0.08197968 -0.10755239  0.68723570  0.17640527
##   High school    0.55700665  0.32536258 -0.57965725  0.21039203  0.09128709
##   Undergraduate -0.02484520  0.12207784 -0.02727227 -0.18419392 -0.59605581
##   Postgraduate  -1.03923048 -0.93106300  1.51496482 -1.30760201  0.79530316
```

```
##
##                         16+ I don't know
##   Primary       -0.30000000  -0.24494897
##   Secondary     -1.13198070  -0.53345145
##   High school   -0.35143175   0.05063697
##   Undergraduate  0.78101179   0.47205880
##   Postgraduate   1.08423040  -0.07698004
```

```
chi.edu.filmreuse.sim$residuals
```

```
##
##                        Never         Once    2-4 times   5-10 times
##   Primary       -1.086278049 -0.644204936  1.404885655 -0.244948974
##   Secondary      0.630531091 -0.028419424 -1.407963632  1.811390080
##   High school    0.833537567  0.004813481 -1.086107559 -0.962102399
##   Undergraduate -1.081270671  0.375518383  0.884266531 -0.024845200
##   Postgraduate  -0.269057083 -0.537844874  2.331551348 -1.039230485
##
##                   More often
##   Primary        3.377622438
##   Secondary     -1.382900607
##   High school   -0.396296962
##   Undergraduate  1.750000000
##   Postgraduate  -1.161895004
```

```
chi.edu.filmdisp.sim$residuals
```

```
##
##                 General waste bin Recycling bin Recycling centre
##   Primary             -1.15108644    0.79618656      -0.29154759
##   Secondary            0.84046565   -0.91248868      -0.06033312
##   High school          0.30979416    0.74980488      -1.92509622
##   Undergraduate       -0.46922219   -0.54735493       2.19700355
##   Postgraduate        -0.78834540    0.94526534      -0.42847960
##
##                 Specialist waste collection    Landfill Deposit return scheme
##   Primary                       -0.17320508 -0.12247449           -0.10000000
##   Secondary                     -1.20623381 -0.85293611           -0.69641941
##   High school                    0.03580574 -0.98742088            0.43412157
##   Undergraduate                  0.68516016 -0.01242260            0.39557740
##   Postgraduate                   0.62598071  3.32938655           -0.42426407
##
##                 Indefinite storage       Other I don't know
##   Primary              -0.12247449  5.60029761  -0.17320508
##   Secondary            -0.85293611 -0.37720714   1.28084621
##   High school           0.02531848 -1.39642400   0.03580574
##   Undergraduate         0.98138539  0.68516016  -0.72029658
##   Postgraduate         -0.51961524  0.62598071  -0.73484692
```

```
chi.edu.bagpurchase.sim$residuals
```

```
##
```

```
##                        None         1-2         3-5        6-10       11-15
##   Primary       -0.14855627 -0.62048368  1.69297454 -0.30822070 -0.26457513
##   Secondary     -0.43462803 -0.15562919  1.41600118 -0.74889048  0.87107419
##   High school    0.29942474 -0.60469773  0.27418769 -0.07042382 -0.25784398
##   Undergraduate  0.07910741  1.17943181 -1.97202932  0.23200331 -0.33353169
##   Postgraduate   0.02626129 -0.73314638  0.57441128  0.98648766 -0.23162641
##
##                         16+ I don't know
##   Primary       -0.20000000  -0.18708287
##   Secondary     -0.67488067   0.23217769
##   High school   -0.37210420   0.48067032
##   Undergraduate  0.79115481  -0.23584252
##   Postgraduate   0.32998316  -0.79372539
```

```
chi.edu.bagreuse.sim$residuals
```

```
##
##                       Never        Once   2-4 times  5-10 times  More often
##   Primary       -0.39370039 -0.46904158  0.78456207 -0.59160798  0.21111111
##   Secondary      1.27014823  0.40716406  0.30480440  0.24878201 -1.16229449
##   High school   -0.02362864 -0.34377583 -0.22281937 -0.36689969  0.59950122
##   Undergraduate -0.76130469  0.29842460 -0.06916611  0.48862727 -0.09128709
##   Postgraduate  -0.47296063 -0.48241815 -0.12789071 -0.51793240  0.89566859
```

```
chi.edu.bagdisp.sim$residuals
```

```
##
##                 General waste bin Recycling bin Recycling centre
##   Primary              0.33704807    0.79618656      -0.38078866
##   Secondary            1.41328553   -0.27734575      -0.76643563
##   High school          0.22369699    0.20116716      -1.11562926
##   Undergraduate       -1.62117500    0.34994824       1.34649277
##   Postgraduate         0.31498449   -0.79235477       0.86038840
##
##                 Specialist waste collection    Landfill Deposit return scheme
##   Primary                       -0.20000000 -0.10000000           -0.23452079
##   Secondary                      0.04307749 -0.69641941           -1.02097152
##   High school                    0.24806947 -0.80622577           -0.30410938
##   Undergraduate                  0.18257419  0.39557740            1.18721089
##   Postgraduate                  -0.84852814  1.93275854            0.01005038
##
##                 Indefinite storage       Other I don't know
##   Primary              -0.64420494 -0.33911650  -0.21213203
##   Secondary             0.19447802 -1.93824451   0.55336366
##   High school           0.77497048 -1.27101071   0.62855825
##   Undergraduate        -0.94706209  2.95661395  -0.59529188
##   Postgraduate          0.19391686 -0.04865336  -0.90000000
```

Simulated observed and expected values for 'education'

```
chi.edu.choice.sim$observed
```

```
##
##                Always As often as they can If cheaper or preferred
##   Primary           1                    1                       0
##   Secondary         7                   50                      31
##   High school       9                   67                      43
##   Undergraduate    23                   71                      32
##   Postgraduate      6                   19                      10
##
##                Rarely or never
##   Primary                    0
##   Secondary                  9
##   High school               11
##   Undergraduate              9
##   Postgraduate               1
```

```
chi.edu.choice.sim$expected
```

```
##
##                Always As often as they can If cheaper or preferred
##   Primary        0.230                 1.04                    0.58
##   Secondary     11.155                50.44                   28.13
##   High school   14.950                67.60                   37.70
##   Undergraduate 15.525                70.20                   39.15
##   Postgraduate   4.140                18.72                   10.44
##
##                Rarely or never
##   Primary                0.150
##   Secondary              7.275
##   High school            9.750
##   Undergraduate         10.125
##   Postgraduate           2.700
```

```
chi.edu.barrier.sim$observed
```

```
##
##                Council collection Unclear information Difficult transport
##   Primary                       1                   0                   0
##   Secondary                    22                  20                   7
##   High school                  37                  23                   8
##   Undergraduate                44                  14                  14
##   Postgraduate                 11                   5                   2
##
##                No local facilities No support Ends up in landfills Forgetting
##   Primary                        1          0                    0          0
##   Secondary                      9          1                    8          5
##   High school                    8          5                    9          8
##   Undergraduate                 15          8                   13          8
##   Postgraduate                   2          4                    3          4
##
##                Recycling a hassle Household disagrees Recycling not important
##   Primary                       0                   0                       0
##   Secondary                     3                   0                       1
##   High school                   3                   2                       1
```

```
##   Undergraduate                  3                 2                       1
##   Postgraduate                   1                 1                       2
##
##                 Other Already doing everything
##   Primary           0                        0
##   Secondary         1                       20
##   High school       3                       23
##   Undergraduate     0                       13
##   Postgraduate      0                        1
```

```r
chi.edu.barrier.sim$expected
```

```
##
##                 Council collection Unclear information Difficult transport
##   Primary                   0.5750               0.310              0.1550
##   Secondary                27.8875              15.035              7.5175
##   High school              37.3750              20.150             10.0750
##   Undergraduate            38.8125              20.925             10.4625
##   Postgraduate             10.3500               5.580              2.7900
##
##                 No local facilities No support Ends up in landfills Forgetting
##   Primary                    0.1750      0.090               0.1650     0.1250
##   Secondary                  8.4875      4.365               8.0025     6.0625
##   High school               11.3750      5.850              10.7250     8.1250
##   Undergraduate             11.8125      6.075              11.1375     8.4375
##   Postgraduate               3.1500      1.620               2.9700     2.2500
##
##                 Recycling a hassle Household disagrees Recycling not important
##   Primary                    0.050              0.0250                  0.0250
##   Secondary                  2.425              1.2125                  1.2125
##   High school                3.250              1.6250                  1.6250
##   Undergraduate              3.375              1.6875                  1.6875
##   Postgraduate               0.900              0.4500                  0.4500
##
##                 Other Already doing everything
##   Primary        0.02                   0.2850
##   Secondary      0.97                  13.8225
##   High school    1.30                  18.5250
##   Undergraduate  1.35                  19.2375
##   Postgraduate   0.36                   5.1300
```

```r
chi.edu.barrier2.sim$observed
```

```
##
##                 Limited alternatives No SUP-free alternatives
##   Primary                          2                        0
##   Secondary                       22                       13
##   High school                     35                       29
##   Undergraduate                   23                       31
##   Postgraduate                     8                       12
##
##                 Alternatives expensive Limited functioning Forgetting reusables
##   Primary                            0                   0                    0
```

```
##   Secondary                         22                   7                    10
##   High school                       23                  12                    16
##   Undergraduate                     34                  16                    13
##   Postgraduate                       6                   4                     1
##
##                 Reducing not important Other No barriers
##   Primary                            0     0           0
##   Secondary                          5     3          15
##   High school                        2     1          12
##   Undergraduate                      3     4          11
##   Postgraduate                       3     0           2
```

```
chi.edu.barrier2.sim$expected
```

```
##
##                 Limited alternatives No SUP-free alternatives
##   Primary                      0.450                   0.4250
##   Secondary                   21.825                  20.6125
##   High school                 29.250                  27.6250
##   Undergraduate               30.375                  28.6875
##   Postgraduate                 8.100                   7.6500
##
##                 Alternatives expensive Limited functioning Forgetting reusables
##   Primary                       0.4250              0.1950                  0.2
##   Secondary                    20.6125              9.4575                  9.7
##   High school                  27.6250             12.6750                 13.0
##   Undergraduate                28.6875             13.1625                 13.5
##   Postgraduate                  7.6500              3.5100                  3.6
##
##                 Reducing not important Other No barriers
##   Primary                       0.0650  0.04         0.2
##   Secondary                     3.1525  1.94         9.7
##   High school                   4.2250  2.60        13.0
##   Undergraduate                 4.3875  2.70        13.5
##   Postgraduate                  1.1700  0.72         3.6
```

```
chi.edu.mainconsid.sim$observed
```

```
##
##                 Value for money Price Quality Deals/discounts
##   Primary                     0     0       0               0
##   Secondary                  27    27      18               7
##   High school                40    29      30               7
##   Undergraduate              41    34      28              10
##   Postgraduate               11     7      12               2
##
##                 Use-by-dates/longevity Convenience Ease of recycling packaging
##   Primary                            1           0                           0
##   Secondary                          2           3                           6
##   High school                        7           3                          10
##   Undergraduate                      3           4                           5
##   Postgraduate                       1           0                           1
##
```

```
##                 Sustainability Brand Ethics Other
##   Primary                    1     0      0     0
##   Secondary                  2     2      1     2
##   High school                1     1      2     0
##   Undergraduate              8     1      1     0
##   Postgraduate               1     0      1     0
```

```
chi.edu.mainconsid.sim$expected
```

```
##
##                 Value for money   Price Quality Deals/discounts
##   Primary                0.5950  0.4850    0.44           0.130
##   Secondary             28.8575 23.5225   21.34           6.305
##   High school           38.6750 31.5250   28.60           8.450
##   Undergraduate         40.1625 32.7375   29.70           8.775
##   Postgraduate          10.7100  8.7300    7.92           2.340
##
##                 Use-by-dates/longevity Convenience Ease of recycling packaging
##   Primary                        0.070       0.050                       0.110
##   Secondary                      3.395       2.425                       5.335
##   High school                    4.550       3.250                       7.150
##   Undergraduate                  4.725       3.375                       7.425
##   Postgraduate                   1.260       0.900                       1.980
##
##                 Sustainability Brand Ethics Other
##   Primary               0.0650  0.02 0.0250 0.010
##   Secondary             3.1525  0.97 1.2125 0.485
##   High school           4.2250  1.30 1.6250 0.650
##   Undergraduate         4.3875  1.35 1.6875 0.675
##   Postgraduate          1.1700  0.36 0.4500 0.180
```

```
chi.edu.litter.sim$observed
```

```
##
##                 Strongly agree Agree Neither agree nor disagree Disagree
##   Primary                    0     2                           0        0
##   Secondary                 42    37                          17        1
##   High school               57    54                          16        2
##   Undergraduate             56    55                          16        5
##   Postgraduate              18    14                           4        0
##
##                 Strongly disagree
##   Primary                       0
##   Secondary                     0
##   High school                   1
##   Undergraduate                 3
##   Postgraduate                  0
```

```
chi.edu.litter.sim$expected
```

```
##
##                 Strongly agree  Agree Neither agree nor disagree Disagree
```

```
##   Primary              0.8650  0.810                      0.2650     0.04
##   Secondary           41.9525 39.285                     12.8525     1.94
##   High school         56.2250 52.650                     17.2250     2.60
##   Undergraduate       58.3875 54.675                     17.8875     2.70
##   Postgraduate        15.5700 14.580                      4.7700     0.72
##
##                 Strongly disagree
##   Primary                    0.02
##   Secondary                  0.97
##   High school                1.30
##   Undergraduate              1.35
##   Postgraduate               0.36
```

```
chi.edu.attitude.sim$observed
```

```
##
##                 1 (Not concerned)  2  3  4  5  6 7 (Very concerned
##   Primary                       0  0  1  0  0  1                 0
##   Secondary                     9  8  7 15 25 12                21
##   High school                   7  1 19 28 40 16                19
##   Undergraduate                 8 10 14 25 36 18                24
##   Postgraduate                  3  1  0  5 13  9                 5
```

```
chi.edu.attitude.sim$expected
```

```
##
##                 1 (Not concerned)    2       3       4      5     6
##   Primary                  0.1350 0.10  0.2050  0.3650  0.570  0.28
##   Secondary                6.5475 4.85  9.9425 17.7025 27.645 13.58
##   High school              8.7750 6.50 13.3250 23.7250 37.050 18.20
##   Undergraduate            9.1125 6.75 13.8375 24.6375 38.475 18.90
##   Postgraduate             2.4300 1.80  3.6900  6.5700 10.260  5.04
##
##                 7 (Very concerned
##   Primary                  0.3450
##   Secondary               16.7325
##   High school             22.4250
##   Undergraduate           23.2875
##   Postgraduate             6.2100
```

```
chi.edu.ocean.sim$observed
```

```
##
##                 Always Most of the time Sometimes Rarely Never
##   Primary            0                1         1      0     0
##   Secondary          5               20        50     16     6
##   High school        8               31        72     17     2
##   Undergraduate      5               41        62     21     6
##   Postgraduate       1               11        15      6     3
```

```r
chi.edu.ocean.sim$expected
```

```
##
##                Always Most of the time Sometimes Rarely  Never
##   Primary      0.0950             0.52       1.0   0.30 0.0850
##   Secondary    4.6075            25.22      48.5  14.55 4.1225
##   High school  6.1750            33.80      65.0  19.50 5.5250
##   Undergraduate 6.4125           35.10      67.5  20.25 5.7375
##   Postgraduate  1.7100            9.36      18.0   5.40 1.5300
```

```r
chi.edu.actions.sim$observed
```

```
##
##                Yes, definitely Yes, probably No, probably not
##   Primary                    2             0                0
##   Secondary                 30            46               15
##   High school               45            62               15
##   Undergraduate             55            62               11
##   Postgraduate              10            21                2
##
##                No, definitely not I don't know
##   Primary                       0            0
##   Secondary                     2            4
##   High school                   5            3
##   Undergraduate                 2            5
##   Postgraduate                  2            1
```

```r
chi.edu.actions.sim$expected
```

```
##
##                Yes, definitely Yes, probably No, probably not
##   Primary                0.710        0.9550           0.2150
##   Secondary             34.435       46.3175          10.4275
##   High school           46.150       62.0750          13.9750
##   Undergraduate         47.925       64.4625          14.5125
##   Postgraduate          12.780       17.1900           3.8700
##
##                No, definitely not I don't know
##   Primary                  0.0550       0.0650
##   Secondary                2.6675       3.1525
##   High school              3.5750       4.2250
##   Undergraduate            3.7125       4.3875
##   Postgraduate             0.9900       1.1700
```

```r
chi.edu.attplast.sim$observed
```

```
##
##                Will go out of their way to avoid
##   Primary                                      0
##   Secondary                                   17
##   High school                                 35
```

```
##   Undergraduate                               36
##   Postgraduate                                14
##
##                 If option is readily available, will avoid
##   Primary                                              2
##   Secondary                                           54
##   High school                                         71
##   Undergraduate                                       76
##   Postgraduate                                        15
##
##                 Will avoid only without extra costs Not a priority
##   Primary                                         0              0
##   Secondary                                      19              7
##   High school                                    16              8
##   Undergraduate                                  17              6
##   Postgraduate                                    6              1
```

```
chi.edu.attplast.sim$expected
```

```
##
##                 Will go out of their way to avoid
##   Primary                                 0.510
##   Secondary                              24.735
##   High school                            33.150
##   Undergraduate                          34.425
##   Postgraduate                            9.180
##
##                 If option is readily available, will avoid
##   Primary                                          1.090
##   Secondary                                       52.865
##   High school                                     70.850
##   Undergraduate                                   73.575
##   Postgraduate                                    19.620
##
##                 Will avoid only without extra costs Not a priority
##   Primary                                     0.290          0.110
##   Secondary                                  14.065          5.335
##   High school                                18.850          7.150
##   Undergraduate                              19.575          7.425
##   Postgraduate                                5.220          1.980
```

```
chi.edu.zerow.sim$observed
```

```
##
##                 Yes, shops regularly Yes, shops occasionally
##   Primary                          1                       0
##   Secondary                        3                       4
##   High school                      3                       7
##   Undergraduate                    4                      14
##   Postgraduate                     3                       5
##
##                 Yes, visited at least once Yes, never visited
##   Primary                                0                  0
```

```
##   Secondary                          3                 17
##   High school                        8                 29
##   Undergraduate                      9                 38
##   Postgraduate                       4                  4
##
##                  No, but would like to shop there No, not likely to shop there
##   Primary                                       1                            0
##   Secondary                                    48                           22
##   High school                                  60                           23
##   Undergraduate                                51                           19
##   Postgraduate                                 15                            5
```

```
chi.edu.zerow.sim$expected
```

```
##
##                  Yes, shops regularly Yes, shops occasionally
##   Primary                       0.070                   0.150
##   Secondary                     3.395                   7.275
##   High school                   4.550                   9.750
##   Undergraduate                 4.725                  10.125
##   Postgraduate                  1.260                   2.700
##
##                  Yes, visited at least once Yes, never visited
##   Primary                             0.12               0.44
##   Secondary                           5.82              21.34
##   High school                         7.80              28.60
##   Undergraduate                       8.10              29.70
##   Postgraduate                        2.16               7.92
##
##                  No, but would like to shop there No, not likely to shop there
##   Primary                                  0.8750                       0.3450
##   Secondary                               42.4375                      16.7325
##   High school                             56.8750                      22.4250
##   Undergraduate                           59.0625                      23.2875
##   Postgraduate                            15.7500                       6.2100
```

```
chi.edu.bottpurchase.sim$observed
```

```
##
##                  None 1-2 3-5 6-10 11-15 16+ I don't know
##   Primary           0   0   1    1     0   0            0
##   Secondary         2  36  29   21     6   1            2
##   High school       5  44  44   22     6   3            6
##   Undergraduate     3  46  49   23     6   6            2
##   Postgraduate      2  11  11    7     1   4            0
```

```
chi.edu.bottpurchase.sim$expected
```

```
##
##                  None     1-2    3-5   6-10  11-15   16+ I don't know
##   Primary        0.06  0.6850  0.670  0.370 0.0950 0.070        0.050
##   Secondary      2.91 33.2225 32.495 17.945 4.6075 3.395        2.425
```

```
##   High school   3.90 44.5250 43.550 24.050 6.1750 4.550        3.250
##   Undergraduate 4.05 46.2375 45.225 24.975 6.4125 4.725        3.375
##   Postgraduate  1.08 12.3300 12.060  6.660 1.7100 1.260        0.900
```

```
chi.edu.bottreuse.sim$observed
```

```
##
##                 Never Once 2-4 times 5-10 times More often
##   Primary           1    0         1          0          0
##   Secondary        23   18        23         20         13
##   High school      23   22        39         18         28
##   Undergraduate    19   21        53         15         27
##   Postgraduate      5    5        13          4          9
```

```
chi.edu.bottreuse.sim$expected
```

```
##
##                   Never   Once 2-4 times 5-10 times More often
##   Primary        0.3550  0.330    0.6450     0.2850     0.3850
##   Secondary     17.2175 16.005   31.2825    13.8225    18.6725
##   High school   23.0750 21.450   41.9250    18.5250    25.0250
##   Undergraduate 23.9625 22.275   43.5375    19.2375    25.9875
##   Postgraduate   6.3900  5.940   11.6100     5.1300     6.9300
```

```
chi.edu.bottdisp.sim$observed
```

```
##
##                 General waste bin Recycling bin Recycling centre
##   Primary                       0             1                0
##   Secondary                     7            86                1
##   High school                   8           105                3
##   Undergraduate                 9           108                5
##   Postgraduate                  3            28                1
##
##                 Specialist waste collection Landfill Deposit return scheme
##   Primary                                 0        0                     1
##   Secondary                               1        1                     0
##   High school                             2        0                     2
##   Undergraduate                           2        4                     1
##   Postgraduate                            2        0                     0
##
##                 Indefinite storage Other I don't know
##   Primary                        0     0            0
##   Secondary                      0     0            1
##   High school                    9     1            0
##   Undergraduate                  5     1            0
##   Postgraduate                   2     0            0
```

```
chi.edu.bottdisp.sim$expected
```

```
##
```

```
##                 General waste bin Recycling bin Recycling centre
##   Primary                  0.1350          1.64            0.050
##   Secondary                6.5475         79.54            2.425
##   High school              8.7750        106.60            3.250
##   Undergraduate            9.1125        110.70            3.375
##   Postgraduate             2.4300         29.52            0.900
##
##                 Specialist waste collection Landfill Deposit return scheme
##   Primary                            0.0350   0.0250                  0.02
##   Secondary                          1.6975   1.2125                  0.97
##   High school                        2.2750   1.6250                  1.30
##   Undergraduate                      2.3625   1.6875                  1.35
##   Postgraduate                       0.6300   0.4500                  0.36
##
##                 Indefinite storage Other I don't know
##   Primary                     0.08 0.010       0.0050
##   Secondary                   3.88 0.485       0.2425
##   High school                 5.20 0.650       0.3250
##   Undergraduate               5.40 0.675       0.3375
##   Postgraduate                1.44 0.180       0.0900
```

```
chi.edu.tubspurchase.sim$observed
```

```
##
##                 None 1-2 3-5 6-10 11-15 16+ I don't know
##   Primary          0   0   2    0     0   0            0
##   Secondary        5  28  41   12     8   1            2
##   High school      4  35  43   32    10   2            4
##   Undergraduate    3  38  43   30     9   8            4
##   Postgraduate     0   7  14   11     2   2            0
```

```
chi.edu.tubspurchase.sim$expected
```

```
##
##                 None   1-2     3-5    6-10  11-15    16+ I don't know
##   Primary       0.06  0.54  0.7150  0.4250 0.1450 0.0650        0.050
##   Secondary     2.91 26.19 34.6775 20.6125 7.0325 3.1525        2.425
##   High school   3.90 35.10 46.4750 27.6250 9.4250 4.2250        3.250
##   Undergraduate 4.05 36.45 48.2625 28.6875 9.7875 4.3875        3.375
##   Postgraduate  1.08  9.72 12.8700  7.6500 2.6100 1.1700        0.900
```

```
chi.edu.tubsreuse.sim$observed
```

```
##
##                 Never Once 2-4 times 5-10 times More often
##   Primary           0    0         1          0          1
##   Secondary        18   14        24         14         27
##   High school      12   13        32         17         56
##   Undergraduate    28    9        33         18         47
##   Postgraduate      3    5         7          9         12
```

```r
chi.edu.tubsreuse.sim$expected
```

```
##
##                    Never    Once 2-4 times 5-10 times More often
##   Primary        0.3050  0.2050    0.4850      0.290     0.7150
##   Secondary     14.7925  9.9425   23.5225     14.065    34.6775
##   High school   19.8250 13.3250   31.5250     18.850    46.4750
##   Undergraduate 20.5875 13.8375   32.7375     19.575    48.2625
##   Postgraduate   5.4900  3.6900    8.7300      5.220    12.8700
```

```r
chi.edu.tubdisp.sim$observed
```

```
##
##                 General waste bin Recycling bin Recycling centre
##   Primary                       0             2                0
##   Secondary                    24            55                3
##   High school                  22            68                9
##   Undergraduate                33            68               12
##   Postgraduate                  7            18                5
##
##                 Specialist waste collection Landfill Deposit return scheme
##   Primary                                 0        0                     0
##   Secondary                               3        1                     0
##   High school                             5        0                     2
##   Undergraduate                           3        1                     2
##   Postgraduate                            1        1                     0
##
##                 Indefinite storage Other I don't know
##   Primary                        0     0            0
##   Secondary                      9     2            0
##   High school                   19     0            5
##   Undergraduate                 10     5            1
##   Postgraduate                   4     0            0
```

```r
chi.edu.tubdisp.sim$expected
```

```
##
##                 General waste bin Recycling bin Recycling centre
##   Primary                   0.430        1.0550           0.1450
##   Secondary                20.855       51.1675           7.0325
##   High school              27.950       68.5750           9.4250
##   Undergraduate            29.025       71.2125           9.7875
##   Postgraduate              7.740       18.9900           2.6100
##
##                 Specialist waste collection Landfill Deposit return scheme
##   Primary                              0.06   0.0150                  0.02
##   Secondary                            2.91   0.7275                  0.97
##   High school                          3.90   0.9750                  1.30
##   Undergraduate                        4.05   1.0125                  1.35
##   Postgraduate                         1.08   0.2700                  0.36
##
```

```
##                  Indefinite storage  Other I don't know
##   Primary                     0.210 0.0350        0.030
##   Secondary                  10.185 1.6975        1.455
##   High school                13.650 2.2750        1.950
##   Undergraduate              14.175 2.3625        2.025
##   Postgraduate                3.780 0.6300        0.540
```

```r
chi.edu.filmpurchase.sim$observed
```

```
##
##                  None 1-2 3-5 6-10 11-15 16+ I don't know
##   Primary           0   1   0    1     0   0            0
##   Secondary         3  20  29   31    10   2            2
##   High school       5  29  36   38    13   5            4
##   Undergraduate     4  29  41   37    11   8            5
##   Postgraduate      0   5  16    6     5   3            1
```

```r
chi.edu.filmpurchase.sim$expected
```

```
##
##                  None   1-2    3-5    6-10   11-15   16+ I don't know
##   Primary       0.06  0.42  0.610  0.5650  0.1950 0.090         0.06
##   Secondary     2.91 20.37 29.585 27.4025  9.4575 4.365         2.91
##   High school   3.90 27.30 39.650 36.7250 12.6750 5.850         3.90
##   Undergraduate 4.05 28.35 41.175 38.1375 13.1625 6.075         4.05
##   Postgraduate  1.08  7.56 10.980 10.1700  3.5100 1.620         1.08
```

```r
chi.edu.filmreuse.sim$observed
```

```
##
##                  Never Once 2-4 times 5-10 times More often
##   Primary           0    0         1          0          1
##   Secondary        62   20         8          6          1
##   High school      84   27        13          2          4
##   Undergraduate    70   30        22          4          9
##   Postgraduate     20    6        10          0          0
```

```r
chi.edu.filmreuse.sim$expected
```

```
##
##                  Never    Once 2-4 times 5-10 times More often
##   Primary        1.18  0.4150     0.270       0.06     0.0750
##   Secondary     57.23 20.1275    13.095       2.91     3.6375
##   High school   76.70 26.9750    17.550       3.90     4.8750
##   Undergraduate 79.65 28.0125    18.225       4.05     5.0625
##   Postgraduate  21.24  7.4700     4.860       1.08     1.3500
```

```r
chi.edu.filmdisp.sim$observed
```

```
##
##                 General waste bin Recycling bin Recycling centre
##   Primary                       0             1                0
##   Secondary                    71            18                4
##   High school                  89            34                1
##   Undergraduate                85            28               11
##   Postgraduate                 20            11                1
##
##                 Specialist waste collection Landfill Deposit return scheme
##   Primary                                 0        0                     0
##   Secondary                               0        0                     0
##   High school                             2        0                     1
##   Undergraduate                           3        1                     1
##   Postgraduate                            1        2                     0
##
##                 Indefinite storage Other I don't know
##   Primary                        0     1            0
##   Secondary                      0     1            3
##   High school                    1     0            2
##   Undergraduate                  2     3            1
##   Postgraduate                   0     1            0
```

```
chi.edu.filmdisp.sim$expected
```

```
##
##                 General waste bin Recycling bin Recycling centre
##   Primary                  1.3250          0.46           0.0850
##   Secondary               64.2625         22.31           4.1225
##   High school             86.1250         29.90           5.5250
##   Undergraduate           89.4375         31.05           5.7375
##   Postgraduate            23.8500          8.28           1.5300
##
##                 Specialist waste collection Landfill Deposit return scheme
##   Primary                             0.030   0.0150                 0.010
##   Secondary                           1.455   0.7275                 0.485
##   High school                         1.950   0.9750                 0.650
##   Undergraduate                       2.025   1.0125                 0.675
##   Postgraduate                        0.540   0.2700                 0.180
##
##                 Indefinite storage Other I don't know
##   Primary                   0.0150 0.030        0.030
##   Secondary                 0.7275 1.455        1.455
##   High school               0.9750 1.950        1.950
##   Undergraduate             1.0125 2.025        2.025
##   Postgraduate              0.2700 0.540        0.540
```

```
chi.edu.bagpurchase.sim$observed
```

```
##
##                 None 1-2 3-5 6-10 11-15 16+ I don't know
##   Primary          1   0   1    0     0   0            0
##   Secondary       53  18  15    3     5   1            2
##   High school     78  22  15    6     4   2            3
```

```
##   Undergraduate   79  32   7    7     4   4           2
##   Postgraduate    21   5   5    3     1   1           0
```

```
chi.edu.bagpurchase.sim$expected
```

```
##
##                   None     1-2     3-5   6-10 11-15  16+ I don't know
##   Primary         1.16  0.3850  0.2150 0.0950 0.070 0.04       0.0350
##   Secondary      56.26 18.6725 10.4275 4.6075 3.395 1.94       1.6975
##   High school    75.40 25.0250 13.9750 6.1750 4.550 2.60       2.2750
##   Undergraduate 78.30 25.9875 14.5125 6.4125 4.725 2.70       2.3625
##   Postgraduate  20.88  6.9300  3.8700 1.7100 1.260 0.72       0.6300
```

```
chi.edu.bagreuse.sim$observed
```

```
##
##                 Never Once 2-4 times 5-10 times More often
##   Primary           0    0         1          0          1
##   Secondary        11   12        24         18         32
##   High school      10   13        29         21         57
##   Undergraduate     8   16        31         26         54
##   Postgraduate      2    3         8          5         18
```

```
chi.edu.bagreuse.sim$expected
```

```
##
##                   Never  Once 2-4 times 5-10 times More often
##   Primary        0.1550  0.22    0.4650      0.350      0.810
##   Secondary      7.5175 10.67   22.5525     16.975     39.285
##   High school   10.0750 14.30   30.2250     22.750     52.650
##   Undergraduate 10.4625 14.85   31.3875     23.625     54.675
##   Postgraduate   2.7900  3.96    8.3700      6.300     14.580
```

```
chi.edu.bagdisp.sim$observed
```

```
##
##                 General waste bin Recycling bin Recycling centre
##   Primary                       1             1                0
##   Secondary                    43            21                5
##   High school                  48            31                6
##   Undergraduate                37            33               14
##   Postgraduate                 14             6                4
##
##                 Specialist waste collection Landfill Deposit return scheme
##   Primary                                 0        0                     0
##   Secondary                               2        0                     1
##   High school                             3        0                     3
##   Undergraduate                           3        1                     6
##   Postgraduate                            0        1                     1
##
##                 Indefinite storage Other I don't know
```

```
##    Primary                          0     0            0
##    Secondary                       21     1            3
##    High school                     31     4            4
##    Undergraduate                   23    16            2
##    Postgraduate                     8     2            0
```

```r
chi.edu.bagdisp.sim$expected
```

```
##
##                   General waste bin Recycling bin Recycling centre
##    Primary                   0.7150          0.46           0.1450
##    Secondary                34.6775         22.31           7.0325
##    High school              46.4750         29.90           9.4250
##    Undergraduate            48.2625         31.05           9.7875
##    Postgraduate             12.8700          8.28           2.6100
##
##                   Specialist waste collection Landfill Deposit return scheme
##    Primary                               0.04    0.010                0.0550
##    Secondary                             1.94    0.485                2.6675
##    High school                           2.60    0.650                3.5750
##    Undergraduate                         2.70    0.675                3.7125
##    Postgraduate                          0.72    0.180                0.9900
##
##                   Indefinite storage  Other I don't know
##    Primary                    0.4150 0.1150       0.0450
##    Secondary                 20.1275 5.5775       2.1825
##    High school               26.9750 7.4750       2.9250
##    Undergraduate             28.0125 7.7625       3.0375
##    Postgraduate               7.4700 2.0700       0.8100
```

Gender

```r
chi.gen.choice <- chisq.test(gen.choice)
```

```
## Warning in chisq.test(gen.choice): Chi-squared approximation may be incorrect
```

```r
chi.gen.choice.sim <- chisq.test(gen.choice, simulate.p.value = TRUE)
chi.gen.choice.sim
```

```
##
##  Pearson's Chi-squared test with simulated p-value (based on 2000
##  replicates)
##
## data:  gen.choice
## X-squared = 7.8125, df = NA, p-value = 0.2409
```

```r
chi.gen.barrier <- chisq.test(gen.barrier)
```

```
## Warning in chisq.test(gen.barrier): Chi-squared approximation may be incorrect
```

```
chi.gen.barrier.sim <- chisq.test(gen.barrier, simulate.p.value = TRUE)
chi.gen.barrier.sim
```

```
##
##  Pearson's Chi-squared test with simulated p-value (based on 2000
##  replicates)
##
## data:  gen.barrier
## X-squared = 22.552, df = NA, p-value = 0.3553
```

```
chi.gen.barrier2 <- chisq.test(gen.barrier2)
```

```
## Warning in chisq.test(gen.barrier2): Chi-squared approximation may be incorrect
```

```
chi.gen.barrier2.sim <- chisq.test(gen.barrier2, simulate.p.value = TRUE)
chi.gen.barrier2.sim
```

```
##
##  Pearson's Chi-squared test with simulated p-value (based on 2000
##  replicates)
##
## data:  gen.barrier2
## X-squared = 15.784, df = NA, p-value = 0.3148
```

```
chi.gen.mainconsid <- chisq.test(gen.mainconsid)
```

```
## Warning in chisq.test(gen.mainconsid): Chi-squared approximation may be
## incorrect
```

```
chi.gen.mainconsid.sim <- chisq.test(gen.mainconsid, simulate.p.value =
TRUE)
chi.gen.mainconsid.sim
```

```
##
##  Pearson's Chi-squared test with simulated p-value (based on 2000
##  replicates)
##
## data:  gen.mainconsid
## X-squared = 15.92, df = NA, p-value = 0.5482
```

```
chi.gen.litter <- chisq.test(gen.litter)
```

```
## Warning in chisq.test(gen.litter): Chi-squared approximation may be incorrect
```

```
chi.gen.litter.sim <- chisq.test(gen.litter, simulate.p.value = TRUE)
chi.gen.litter.sim
```

```
##
##  Pearson's Chi-squared test with simulated p-value (based on 2000
##  replicates)
##
## data:  gen.litter
## X-squared = 9.2783, df = NA, p-value = 0.2519
```

```
chi.gen.attitude <- chisq.test(gen.attitude)
```

```
## Warning in chisq.test(gen.attitude): Chi-squared approximation may be incorrect
```

```
chi.gen.attitude.sim <- chisq.test(gen.attitude, simulate.p.value = TRUE)
chi.gen.attitude.sim
```

```
##
##  Pearson's Chi-squared test with simulated p-value (based on 2000
##  replicates)
##
## data:  gen.attitude
## X-squared = 10.289, df = NA, p-value = 0.5852
```

```
chi.gen.ocean <- chisq.test(gen.ocean)
```

```
## Warning in chisq.test(gen.ocean): Chi-squared approximation may be incorrect
```

```
chi.gen.ocean.sim <- chisq.test(gen.ocean, simulate.p.value = TRUE)
chi.gen.ocean.sim
```

```
##
##  Pearson's Chi-squared test with simulated p-value (based on 2000
##  replicates)
##
## data:  gen.ocean
## X-squared = 12.974, df = NA, p-value = 0.1244
```

```
chi.gen.actions <- chisq.test(gen.actions)
```

```
## Warning in chisq.test(gen.actions): Chi-squared approximation may be incorrect
```

```
chi.gen.actions.sim <- chisq.test(gen.actions, simulate.p.value = TRUE)
chi.gen.actions.sim
```

```
##
##  Pearson's Chi-squared test with simulated p-value (based on 2000
##  replicates)
##
## data:  gen.actions
## X-squared = 12.17, df = NA, p-value = 0.2074
```

```r
chi.gen.attplast <- chisq.test(gen.attplast)
```

```
## Warning in chisq.test(gen.attplast): Chi-squared approximation may be incorrect
```

```r
chi.gen.attplast.sim <- chisq.test(gen.attplast, simulate.p.value = TRUE)
chi.gen.attplast.sim
```

```
##
##  Pearson's Chi-squared test with simulated p-value (based on 2000
##  replicates)
##
## data:  gen.attplast
## X-squared = 6.1731, df = NA, p-value = 0.3808
```

```r
chi.gen.zerow <- chisq.test(gen.zerow)
```

```
## Warning in chisq.test(gen.zerow): Chi-squared approximation may be incorrect
```

```r
chi.gen.zerow.sim <- chisq.test(gen.zerow, simulate.p.value = TRUE)
chi.gen.zerow.sim
```

```
##
##  Pearson's Chi-squared test with simulated p-value (based on 2000
##  replicates)
##
## data:  gen.zerow
## X-squared = 20.793, df = NA, p-value = 0.03248
```

```r
chi.gen.bottpurchase <- chisq.test(gen.bottpurchase)
```

```
## Warning in chisq.test(gen.bottpurchase): Chi-squared approximation may be
## incorrect
```

```r
chi.gen.bottpurchase.sim <- chisq.test(gen.bottpurchase, simulate.p.value =
TRUE)
chi.gen.bottpurchase.sim
```

```
##
##  Pearson's Chi-squared test with simulated p-value (based on 2000
##  replicates)
##
## data:  gen.bottpurchase
## X-squared = 29.989, df = NA, p-value = 0.02449
```

```r
chi.gen.bottreuse <- chisq.test(gen.bottreuse)
```

```
## Warning in chisq.test(gen.bottreuse): Chi-squared approximation may be incorrect
```

```r
chi.gen.bottreuse.sim <- chisq.test(gen.bottreuse, simulate.p.value = TRUE)
chi.gen.bottreuse.sim
```

```
##
##  Pearson's Chi-squared test with simulated p-value (based on 2000
##  replicates)
##
## data:  gen.bottreuse
## X-squared = 9.8272, df = NA, p-value = 0.2754
```

```r
chi.gen.bottdisp <- chisq.test(gen.bottdisp)
```

```
## Warning in chisq.test(gen.bottdisp): Chi-squared approximation may be incorrect
```

```r
chi.gen.bottdisp.sim <- chisq.test(gen.bottdisp, simulate.p.value = TRUE)
chi.gen.bottdisp.sim
```

```
##
##  Pearson's Chi-squared test with simulated p-value (based on 2000
##  replicates)
##
## data:  gen.bottdisp
## X-squared = 166.58, df = NA, p-value = 0.0004998
```

```r
chi.gen.tubspurchase <- chisq.test(gen.tubspurchase)
```

```
## Warning in chisq.test(gen.tubspurchase): Chi-squared approximation may be
## incorrect
```

```r
chi.gen.tubspurchase.sim <- chisq.test(gen.tubspurchase, simulate.p.value =
TRUE)
chi.gen.tubspurchase.sim
```

```
##
##  Pearson's Chi-squared test with simulated p-value (based on 2000
##  replicates)
##
## data:  gen.tubspurchase
## X-squared = 21.081, df = NA, p-value = 0.07446
```

```r
chi.gen.tubreuse <- chisq.test(gen.tubreuse)
```

```
## Warning in chisq.test(gen.tubreuse): Chi-squared approximation may be incorrect
```

```r
chi.gen.tubreuse.sim <- chisq.test(gen.tubreuse, simulate.p.value = TRUE)
chi.gen.tubreuse.sim
```

```
##
##  Pearson's Chi-squared test with simulated p-value (based on 2000
##  replicates)
##
## data:  gen.tubreuse
## X-squared = 16.833, df = NA, p-value = 0.02949
```

```
chi.gen.tubdisp <- chisq.test(gen.tubdisp)
```

```
## Warning in chisq.test(gen.tubdisp): Chi-squared approximation may be incorrect
```

```
chi.gen.tubdisp.sim <- chisq.test(gen.tubdisp, simulate.p.value = TRUE)
chi.gen.tubdisp.sim
```

```
##
##  Pearson's Chi-squared test with simulated p-value (based on 2000
##  replicates)
##
## data:  gen.tubdisp
## X-squared = 42.329, df = NA, p-value = 0.04198
```

```
chi.gen.filmpurchase <- chisq.test(gen.filmpurchase)
```

```
## Warning in chisq.test(gen.filmpurchase): Chi-squared approximation may be
## incorrect
```

```
chi.gen.filmpurchase.sim <- chisq.test(gen.filmpurchase, simulate.p.value =
TRUE)
chi.gen.filmpurchase.sim
```

```
##
##  Pearson's Chi-squared test with simulated p-value (based on 2000
##  replicates)
##
## data:  gen.filmpurchase
## X-squared = 18.059, df = NA, p-value = 0.1509
```

```
chi.gen.filmreuse <- chisq.test(gen.filmreuse)
```

```
## Warning in chisq.test(gen.filmreuse): Chi-squared approximation may be incorrect
```

```
chi.gen.filmreuse.sim <- chisq.test(gen.filmreuse, simulate.p.value = TRUE)
chi.gen.filmreuse.sim
```

```
##
##  Pearson's Chi-squared test with simulated p-value (based on 2000
##  replicates)
##
## data:  gen.filmreuse
## X-squared = 14.054, df = NA, p-value = 0.1039
```

```r
chi.gen.filmdisp <- chisq.test(gen.filmdisp)
```

```
## Warning in chisq.test(gen.filmdisp): Chi-squared approximation may be incorrect
```

```r
chi.gen.filmdisp.sim <- chisq.test(gen.filmdisp, simulate.p.value = TRUE)
chi.gen.filmdisp.sim
```

```
##
##  Pearson's Chi-squared test with simulated p-value (based on 2000
##  replicates)
##
## data:  gen.filmdisp
## X-squared = 51.309, df = NA, p-value = 0.04448
```

```r
chi.gen.bagpurhcase <- chisq.test(gen.bagpurchase)
```

```
## Warning in chisq.test(gen.bagpurchase): Chi-squared approximation may be
## incorrect
```

```r
chi.gen.bagpurhcase.sim <- chisq.test(gen.bagpurchase, simulate.p.value =
TRUE)
chi.gen.bagpurhcase.sim
```

```
##
##  Pearson's Chi-squared test with simulated p-value (based on 2000
##  replicates)
##
## data:  gen.bagpurchase
## X-squared = 23.072, df = NA, p-value = 0.07946
```

```r
chi.gen.bagreuse <- chisq.test(gen.bagreuse)
```

```
## Warning in chisq.test(gen.bagreuse): Chi-squared approximation may be incorrect
```

```r
chi.gen.bagreuse.sim <- chisq.test(gen.bagreuse, simulate.p.value = TRUE)
chi.gen.bagreuse.sim
```

```
##
##  Pearson's Chi-squared test with simulated p-value (based on 2000
##  replicates)
##
## data:  gen.bagreuse
## X-squared = 10.725, df = NA, p-value = 0.2144
```

```r
chi.gen.bagdisp <- chisq.test(gen.bagdisp)
```

```
## Warning in chisq.test(gen.bagdisp): Chi-squared approximation may be incorrect
```

```r
chi.gen.bagdisp.sim <- chisq.test(gen.bagdisp, simulate.p.value = TRUE)
chi.gen.bagdisp.sim
```

```
##
##  Pearson's Chi-squared test with simulated p-value (based on 2000
##  replicates)
##
## data:  gen.bagdisp
## X-squared = 41.385, df = NA, p-value = 0.01699
```

Simulated residuals for 'gender'

```r
chi.gen.choice.sim$residuals
```

```
##
##              Always As often as they can If cheaper or preferred
##   Male    0.66390964         -0.78991945              0.02659163
##   Female -0.47400020          0.69249506             -0.03723606
##   Other  -0.58736701         -0.44835883              0.13937463
##
##          Rarely or never
##   Male        1.20556025
##   Female     -1.16325962
##   Other       1.63384346
```

```r
chi.gen.barrier.sim$residuals
```

```
##
##          Council collection Unclear information Difficult transport
##   Male          0.233686213        -1.311445672        -0.641559515
##   Female       -0.085954178         1.142700283         0.575657095
##   Other        -0.928708781        -0.681909085        -0.482182538
##
##          No local facilities   No support Ends up in landfills   Forgetting
##   Male          -1.028723994  1.083834678          0.267283282  1.630892433
##   Female         0.893829715 -0.840071780         -0.386786067 -1.536259790
##   Other         -0.512347538 -0.367423461          1.512581912  1.876388375
##
##          Recycling a hassle Household disagrees Recycling not important
##   Male          0.528312336         0.017789202             0.017789202
##   Female       -0.398873938         0.007231961             0.007231961
##   Other        -0.273861279        -0.193649167            -0.193649167
##
##                 Other Already doing everything
##   Male   -0.461423225              0.312961185
##   Female  0.394576199             -0.352560344
##   Other  -0.173205081              0.875603384
```

```r
chi.gen.barrier2.sim$residuals
```

```
##
##          Limited alternatives No SUP-free alternatives Alternatives expensive
##   Male             0.57862780               1.10883039            -1.13471748
##   Female          -0.51478602              -0.81210575             0.87174203
##   Other            0.39557740              -0.79843597             0.45401261
##
##          Limited functioning Forgetting reusables Reducing not important
##   Male            0.66115991          -1.96230296            -0.05957493
##   Female         -0.47697804           1.65686097             0.08342233
##   Other          -0.54083269          -0.54772256            -0.31224990
##
##                Other No barriers
##   Male    0.47253692  0.30189276
##   Female -0.35676370 -0.38864640
##   Other  -0.24494897  1.27801930
```

```
chi.gen.mainconsid.sim$residuals
```

```
##
##          Value for money         Price       Quality Deals/discounts
##   Male     -0.0007292862 -0.8586552980  1.3976142750   -1.0203813938
##   Female   -0.1307483337  0.6623554055 -1.0453432392    0.8791188862
##   Other     1.1723023147  0.3194846581 -0.8124038405   -0.4415880433
##
##          Use-by-dates/longevity   Convenience Ease of recycling packaging
##   Male            -1.0758654366  0.0251577303                0.7836138289
##   Female           0.9110614003  0.0102275369               -0.5916256591
##   Other           -0.3240370349 -0.2738612788               -0.4062019202
##
##          Sustainability         Brand        Ethics         Other
##   Male    -0.0595749305  0.3341340594 -0.6937788653  1.3613563601
##   Female   0.0834223320 -0.2522700287  0.5857888148 -1.0931605555
##   Other   -0.3122498999 -0.1732050808 -0.1936491673 -0.1224744871
```

```
chi.gen.litter.sim$residuals
```

```
##
##          Strongly agree      Agree Neither agree nor disagree   Disagree
##   Male       -1.3711972  1.2513478                  0.4513190 -0.6525510
##   Female      1.0457874 -0.8939421                 -0.4740210  0.5580150
##   Other       0.6167266 -1.1022704                  0.9556272 -0.2449490
##
##          Strongly disagree
##   Male           0.3341341
##   Female        -0.2522700
##   Other         -0.1732051
```

```
chi.gen.attitude.sim$residuals
```

```
##
##          1 (Not concerned)          2          3          4          5
##   Male           0.1025804  0.3913624 -0.2969460 -1.0866280 -0.3025137
```

```
##   Female        -0.2819599 -0.2748145  0.3035663  0.9664078  0.2283968
##   Other          1.7722222 -0.3872983 -0.5545268 -0.7399324  0.1568140
##
##                  6 7 (Very concerned
##   Male    0.8249719         0.7173461
##   Female -0.7710311        -0.5026582
##   Other   0.8949594        -0.7193747
```

```
chi.gen.ocean.sim$residuals
```

```
##
##              Always Most of the time   Sometimes      Rarely       Never
##   Male   -0.54936521      -0.01248173 -0.45003516  1.08868364  0.10998206
##   Female  0.48896713      -0.14461696  0.50312829 -0.81002264 -0.36318473
##   Other  -0.37749172       1.38137798 -1.22474487 -0.67082039  2.44348875
```

```
chi.gen.actions.sim$residuals
```

```
##
##         Yes, definitely Yes, probably No, probably not No, definitely not
##   Male        0.25502963   -0.51174845       0.48892503         0.31422911
##   Female     -0.20030106    0.45657449      -0.33390698        -0.61337399
##   Other      -0.06298521   -0.36135893      -0.56789083         3.19432499
##
##         I don't know
##   Male    -0.05957493
##   Female   0.08342233
##   Other   -0.31224990
```

```
chi.gen.attplast.sim$residuals
```

```
##
##         Will go out of their way to avoid
##   Male                         -0.5183192
##   Female                        0.3913291
##   Other                         0.2686811
##
##         If option is readily available, will avoid
##   Male                                   0.3114376
##   Female                                -0.1975829
##   Other                                 -0.4966093
##
##         Will avoid only without extra costs Not a priority
##   Male                           -0.1901205      0.4443871
##   Female                          0.2284753     -0.5916257
##   Other                          -0.6595453      2.0556279
```

```
chi.gen.bottpurchase.sim$residuals
```

```
##
##                None        1-2        3-5       6-10      11-15        16+
```

```
##   Male    1.0380524 -0.9671995 -0.5401839  0.6973117  1.6407951  1.0503508
##   Female -0.8104014  0.8999710  0.5515258 -0.7842770 -1.2917933 -0.8177079
##   Other  -0.3000000 -1.0136567 -1.0024969  1.9396410 -0.3774917 -0.3240370
##
##          I don't know
##   Male     -0.9811515
##   Female    0.4193290
##   Other     3.3776224
```

```
chi.gen.bottreuse.sim$residuals
```

```
##
##               Never       Once  2-4 times 5-10 times More often
##   Male    1.1244860  0.9655536 -0.4139654 -0.1085354 -1.3445199
##   Female -0.9860660 -0.8654832  0.4467856  0.1615009  1.0309038
##   Other   0.6406514  0.7177757 -0.9836158 -0.6538348  0.5559692
```

```
chi.gen.bottdisp.sim$residuals
```

```
##
##          General waste bin Recycling bin Recycling centre
##   Male         -0.81605003    0.39007445       0.52831234
##   Female        0.71392500   -0.21286800      -0.39887394
##   Other        -0.45000000   -0.93086200      -0.27386128
##
##          Specialist waste collection    Landfill Deposit return scheme
##   Male                    0.14132542  0.72935727            0.33413406
##   Female                 -0.08923699 -1.14988175           -0.25227003
##   Other                  -0.22912878  4.97032863           -0.17320508
##
##          Indefinite storage       Other I don't know
##   Male          -1.32062509 -0.88881944  -0.62849025
##   Female         1.11257551  0.73639686  -0.77298124
##   Other         -0.34641016 -0.12247449  11.46040284
```

```
chi.gen.tubspurchase.sim$residuals
```

```
##
##                 None         1-2         3-5        6-10       11-15
##   Male    1.49736759  0.20516079 -0.33064338 -0.09923385 -1.02082314
##   Female -1.18385819 -0.19046299  0.27668057  0.17013879  0.88225444
##   Other  -0.30000000  0.21111111 -0.07000666 -0.79843597 -0.46636895
##
##                  16+ I don't know
##   Male    1.26431241  -0.47799688
##   Female -0.99299486   0.01022754
##   Other  -0.31224990   3.37762244
```

```
chi.gen.tubreuse.sim$residuals
```

```
##
```

```
##               Never        Once   2-4 times  5-10 times  More often
##   Male    1.61041723  1.19399612 -0.05088926 -1.23473835 -0.86286611
##   Female -1.23360647 -0.90867858  0.13693714  0.73808576  0.70941657
##   Other  -0.67638746 -0.55452683 -0.85293611  2.37284688 -0.07000666
```

```
chi.gen.tubdisp.sim$residuals
```

```
##
##          General waste bin Recycling bin Recycling centre
##   Male         -0.33800136    1.16711507       0.75195221
##   Female        0.36479889   -0.80801011      -0.55914151
##   Other        -0.80311892   -1.25797456      -0.46636895
##
##          Specialist waste collection    Landfill Deposit return scheme
##   Male                   -0.33989326 -0.16994663            0.33413406
##   Female                 -0.06348766  0.15498459           -0.25227003
##   Other                   3.03333333 -0.15000000           -0.17320508
##
##          Indefinite storage       Other I don't know
##   Male          -2.10896791 -0.46005936  -0.88991063
##   Female         1.57800463 -0.08923699   0.74732904
##   Other          1.22049300  4.13522902  -0.21213203
```

```
chi.gen.filmpurchase.sim$residuals
```

```
##
##                 None         1-2         3-5        6-10       11-15
##   Male    0.11942196 -0.72566820 -0.31547551  1.10238928 -0.86753352
##   Female -0.06348766  0.67894795  0.36367440 -0.91488237  0.55880489
##   Other  -0.30000000 -0.79372539 -0.95655632  0.16565326  1.30816796
##
##                  16+ I don't know
##   Male    0.70880538   0.11942196
##   Female -0.53514554  -0.43694451
##   Other  -0.36742346   3.03333333
```

```
chi.gen.filmreuse.sim$residuals
```

```
##
##              Never       Once  2-4 times 5-10 times More often
##   Male   -0.1263587 -0.8356884  1.0111637 -0.3398933  0.8524598
##   Female  0.1675823  0.7678715 -0.9268993  0.3099692 -0.9895637
##   Other  -0.5787674 -0.7889867  0.9349523 -0.3000000  2.6460138
```

```
chi.gen.bagpurhcase.sim$residuals
```

```
##
##                 None         1-2         3-5        6-10       11-15
##   Male    0.24652982 -1.16319560  0.00363964  0.91074166  0.62510759
##   Female -0.13759481  0.88347390  0.06066552 -0.69820648 -0.47195401
##   Other  -0.56099255  0.55596918 -0.56789083 -0.37749172 -0.32403703
```

```
##
##                 16+ I don't know
##   Male    0.47253692  -0.46005936
##   Female -0.35676370  -0.08923699
##   Other  -0.24494897   4.13522902
```

```r
chi.gen.bagreuse.sim$residuals
```

```
##
##               Never        Once   2-4 times  5-10 times  More often
##   Male    0.50153094  1.58793640 -0.12126831  0.82725936 -1.49886710
##   Female -0.35375900 -1.22674798  0.05801973 -0.59144377  1.13889949
##   Other  -0.48218254 -0.57445626  0.36220403 -0.72456884  0.71216646
```

```r
chi.gen.bagdisp.sim$residuals
```

```
##
##          General waste bin Recycling bin Recycling centre
##   Male          0.86685778    1.43656549      -0.72536058
##   Female       -0.58879141   -1.07496779       0.64202178
##   Other        -1.03561576   -0.83066239      -0.46636895
##
##          Specialist waste collection    Landfill Deposit return scheme
##   Male                   -0.65255098  0.23626846           -0.16550999
##   Female                  0.10062566 -0.17838185            0.16675191
##   Other                   3.83753393 -0.12247449           -0.28722813
##
##          Indefinite storage       Other I don't know
##   Male          -1.70892592 -0.02820048  -0.82472772
##   Female         1.33587624  0.06946153   0.26844118
##   Other          0.47846181 -0.41533119   3.58919417
```

Simulated observed and expected values for 'gender'

```r
chi.gen.choice.sim$observed
```

```
##
##          Always As often as they can If cheaper or preferred Rarely or never
##   Male       21                   75                      46              16
##   Female     25                  132                      69              13
##   Other       0                    1                       1               1
```

```r
chi.gen.choice.sim$expected
```

```
##
##          Always As often as they can If cheaper or preferred Rarely or never
##   Male   18.170                82.16                   45.82          11.850
##   Female 27.485               124.28                   69.31          17.925
##   Other   0.345                 1.56                    0.87           0.225
```

```r
chi.gen.barrier.sim$observed
```

```
##
##          Council collection Unclear information Difficult transport
##   Male                   47                  18                  10
##   Female                 68                  44                  21
##   Other                   0                   0                   0
##
##          No local facilities No support Ends up in landfills Forgetting
##   Male                    10         10                   14         15
##   Female                  25          8                   18          9
##   Other                    0          0                    1          1
##
##          Recycling a hassle Household disagrees Recycling not important Other
##   Male                    5                   2                       2     1
##   Female                  5                   3                       3     3
##   Other                   0                   0                       0     0
##
##          Already doing everything
##   Male                         24
##   Female                       32
##   Other                         1
```

```r
chi.gen.barrier.sim$expected
```

```
##
##          Council collection Unclear information Difficult transport
##   Male              45.4250              24.490             12.2450
##   Female            68.7125              37.045             18.5225
##   Other              0.8625               0.465              0.2325
##
##          No local facilities No support Ends up in landfills Forgetting
##   Male               13.8250      7.110              13.0350     9.8750
##   Female             20.9125     10.755              19.7175    14.9375
##   Other               0.2625      0.135               0.2475     0.1875
##
##          Recycling a hassle Household disagrees Recycling not important Other
##   Male                3.950              1.9750                  1.9750  1.58
##   Female              5.975              2.9875                  2.9875  2.39
##   Other               0.075              0.0375                  0.0375  0.03
##
##          Already doing everything
##   Male                    22.5150
##   Female                  34.0575
##   Other                    0.4275
```

```r
chi.gen.barrier2.sim$observed
```

```
##
##          Limited alternatives No SUP-free alternatives Alternatives expensive
##   Male                     39                       40                     27
```

```
##   Female                    50                       45                     57
##   Other                      1                        0                      1
##
##          Limited functioning Forgetting reusables Reducing not important Other
##   Male                    18                    8                      5     4
##   Female                  21                   32                      8     4
##   Other                    0                    0                      0     0
##
##          No barriers
##   Male            17
##   Female          22
##   Other            1
```

```
chi.gen.barrier2.sim$expected
```

```
##
##          Limited alternatives No SUP-free alternatives Alternatives expensive
##   Male                 35.550                  33.5750                33.5750
##   Female               53.775                  50.7875                50.7875
##   Other                 0.675                   0.6375                 0.6375
##
##          Limited functioning Forgetting reusables Reducing not important Other
##   Male               15.4050                 15.8                 5.1350  3.16
##   Female             23.3025                 23.9                 7.7675  4.78
##   Other               0.2925                  0.3                 0.0975  0.06
##
##          No barriers
##   Male          15.8
##   Female        23.9
##   Other          0.3
```

```
chi.gen.mainconsid.sim$observed
```

```
##
##          Value for money Price Quality Deals/discounts Use-by-dates/longevity
##   Male                47    33      43               7                      3
##   Female              70    63      45              19                     11
##   Other                2     1       0               0                      0
##
##          Convenience Ease of recycling packaging Sustainability Brand Ethics
##   Male             4                          11              5     2      1
##   Female           6                          11              8     2      4
##   Other            0                           0              0     0      0
##
##          Other
##   Male       2
##   Female     0
##   Other      0
```

```
chi.gen.mainconsid.sim$expected
```

```
##
```

```
##          Value for money   Price Quality Deals/discounts Use-by-dates/longevity
##   Male           47.0050 38.3150   34.76          10.270                  5.530
##   Female         71.1025 57.9575   52.58          15.535                  8.365
##   Other           0.8925  0.7275    0.66           0.195                  0.105
##
##          Convenience Ease of recycling packaging Sustainability Brand Ethics
##   Male         3.950                       8.690         5.1350  1.58 1.9750
##   Female       5.975                      13.145         7.7675  2.39 2.9875
##   Other        0.075                       0.165         0.0975  0.03 0.0375
##
##          Other
##   Male   0.790
##   Female 1.195
##   Other  0.015
```

```
chi.gen.litter.sim$observed
```

```
##
##          Strongly agree Agree Neither agree nor disagree Disagree
##   Male               57    74                         23        2
##   Female            114    88                         29        6
##   Other               2     0                          1        0
##
##          Strongly disagree
##   Male                  2
##   Female                2
##   Other                 0
```

```
chi.gen.litter.sim$expected
```

```
##
##          Strongly agree  Agree Neither agree nor disagree Disagree
##   Male          68.3350 63.990                    20.9350     3.16
##   Female       103.3675 96.795                    31.6675     4.78
##   Other          1.2975  1.215                     0.3975     0.06
##
##          Strongly disagree
##   Male               1.58
##   Female             2.39
##   Other              0.03
```

```
chi.gen.attitude.sim$observed
```

```
##
##          1 (Not concerned)  2  3  4  5  6 7 (Very concerned
##   Male                  11  9 15 23 43 26                31
##   Female                15 11 26 50 70 29                38
##   Other                  1  0  0  0  1  1                 0
```

```
chi.gen.attitude.sim$expected
```

```
##
##         1 (Not concerned)     2       3       4      5     6 7 (Very concerned
##   Male            10.6650  7.90 16.1950 28.8350 45.030 22.12           27.2550
##   Female          16.1325 11.95 24.4975 43.6175 68.115 33.46           41.2275
##   Other            0.2025  0.15  0.3075  0.5475  0.855  0.42            0.5175
```

```r
chi.gen.ocean.sim$observed
```

```
##
##         Always Most of the time Sometimes Rarely Never
##   Male       6               41        75     29     7
##   Female    13               61       125     31     9
##   Other      0                2         0      0     1
```

```r
chi.gen.ocean.sim$expected
```

```
##
##          Always Most of the time Sometimes Rarely   Never
##   Male    7.5050            41.08      79.0  23.70  6.7150
##   Female 11.3525            62.14     119.5  35.85 10.1575
##   Other   0.1425             0.78       1.5   0.45  0.1275
```

```r
chi.gen.actions.sim$observed
```

```
##
##         Yes, definitely Yes, probably No, probably not No, definitely not
##   Male               58            71               19                  5
##   Female             83           119               24                  5
##   Other               1             1                0                  1
##
##         I don't know
##   Male             5
##   Female           8
##   Other            0
```

```r
chi.gen.actions.sim$expected
```

```
##
##         Yes, definitely Yes, probably No, probably not No, definitely not
##   Male           56.090       75.4450          16.9850             4.3450
##   Female         84.845      114.1225          25.6925             6.5725
##   Other           1.065        1.4325           0.3225             0.0825
##
##         I don't know
##   Male        5.1350
##   Female      7.7675
##   Other       0.0975
```

```r
chi.gen.attplast.sim$observed
```

```
##
##          Will go out of their way to avoid
##   Male                                  37
##   Female                                64
##   Other                                  1
##
##          If option is readily available, will avoid
##   Male                                           89
##   Female                                        128
##   Other                                           1
##
##          Will avoid only without extra costs Not a priority
##   Male                                    22             10
##   Female                                  36             11
##   Other                                    0              1
```

```
chi.gen.attplast.sim$expected
```

```
##
##          Will go out of their way to avoid
##   Male                              40.290
##   Female                            60.945
##   Other                              0.765
##
##          If option is readily available, will avoid
##   Male                                       86.110
##   Female                                    130.255
##   Other                                       1.635
##
##          Will avoid only without extra costs Not a priority
##   Male                                22.910          8.690
##   Female                              34.655         13.145
##   Other                                0.435          0.165
```

```
chi.gen.bottpurchase.sim$observed
```

```
##
##          None 1-2 3-5 6-10 11-15 16+ I don't know
##   Male      7  47  49   33    12   8            2
##   Female    5  90  85   39     7   6            7
##   Other     0   0   0    2     0   0            1
```

```
chi.gen.bottpurchase.sim$expected
```

```
##
##          None     1-2    3-5   6-10   11-15   16+ I don't know
##   Male   4.74 54.1150 52.930 29.230  7.5050 5.530        3.950
##   Female 7.17 81.8575 80.065 44.215 11.3525 8.365        5.975
##   Other  0.09  1.0275  1.005  0.555  0.1425 0.105        0.075
```

```r
chi.gen.bottreuse.sim$observed
```

```
##
##          Never Once 2-4 times 5-10 times More often
##   Male      34   31        48         22         23
##   Female    36   34        81         35         53
##   Other      1    1         0          0          1
```

```r
chi.gen.bottreuse.sim$expected
```

```
##
##            Never   Once 2-4 times 5-10 times More often
##   Male   28.0450 26.070   50.9550    22.5150    30.4150
##   Female 42.4225 39.435   77.0775    34.0575    46.0075
##   Other   0.5325  0.495    0.9675     0.4275     0.5775
```

```r
chi.gen.bottdisp.sim$observed
```

```
##
##          General waste bin Recycling bin Recycling centre
##   Male                   8           134                5
##   Female                19           193                5
##   Other                  0             1                0
##
##          Specialist waste collection Landfill Deposit return scheme
##   Male                             3        3                     2
##   Female                           4        1                     2
##   Other                            0        1                     0
##
##          Indefinite storage Other I don't know
##   Male                    3     0            0
##   Female                 13     2            0
##   Other                   0     0            1
```

```r
chi.gen.bottdisp.sim$expected
```

```
##
##          General waste bin Recycling bin Recycling centre
##   Male             10.6650        129.56            3.950
##   Female           16.1325        195.98            5.975
##   Other             0.2025          2.46            0.075
##
##          Specialist waste collection Landfill Deposit return scheme
##   Male                        2.7650   1.9750                  1.58
##   Female                      4.1825   2.9875                  2.39
##   Other                       0.0525   0.0375                  0.03
##
##          Indefinite storage Other I don't know
##   Male                 6.32 0.790       0.3950
##   Female               9.56 1.195       0.5975
##   Other                0.12 0.015       0.0075
```

```r
chi.gen.tubspurchase.sim$observed
```

```
##
##          None 1-2 3-5 6-10 11-15 16+ I don't know
##   Male      8  44  54   33     8   8            3
##   Female    4  63  88   52    21   5            6
##   Other     0   1   1    0     0   0            1
```

```r
chi.gen.tubspurchase.sim$expected
```

```
##
##          None   1-2     3-5    6-10   11-15    16+ I don't know
##   Male   4.74 42.66 56.4850 33.5750 11.4550 5.1350        3.950
##   Female 7.17 64.53 85.4425 50.7875 17.3275 7.7675        5.975
##   Other  0.09  0.81  1.0725  0.6375  0.2175 0.0975        0.075
```

```r
chi.gen.tubreuse.sim$observed
```

```
##
##          Never Once 2-4 times 5-10 times More often
##   Male      32   21        38         17         50
##   Female    29   20        59         39         92
##   Other      0    0         0          2          1
```

```r
chi.gen.tubreuse.sim$expected
```

```
##
##            Never    Once 2-4 times 5-10 times More often
##   Male   24.0950 16.1950   38.3150     22.910    56.4850
##   Female 36.4475 24.4975   57.9575     34.655    85.4425
##   Other   0.4575  0.3075    0.7275      0.435     1.0725
```

```r
chi.gen.tubdisp.sim$observed
```

```
##
##          General waste bin Recycling bin Recycling centre
##   Male                  32            94               14
##   Female                54           117               15
##   Other                  0             0                0
##
##          Specialist waste collection Landfill Deposit return scheme
##   Male                             4        1                     2
##   Female                           7        2                     2
##   Other                            1        0                     0
##
##          Indefinite storage Other I don't know
##   Male                    8     2            1
##   Female                 33     4            5
##   Other                   1     1            0
```

```r
chi.gen.tubdisp.sim$expected
```

```
##
##          General waste bin Recycling bin Recycling centre
##   Male              33.970       83.3450          11.4550
##   Female            51.385      126.0725          17.3275
##   Other              0.645        1.5825           0.2175
##
##          Specialist waste collection Landfill Deposit return scheme
##   Male                          4.74   1.1850                  1.58
##   Female                        7.17   1.7925                  2.39
##   Other                         0.09   0.0225                  0.03
##
##          Indefinite storage  Other I don't know
##   Male               16.590 2.7650        2.370
##   Female             25.095 4.1825        3.585
##   Other               0.315 0.0525        0.045
```

```r
chi.gen.filmpurchase.sim$observed
```

```
##
##          None 1-2 3-5 6-10 11-15 16+ I don't know
##   Male      5  29  46   52    12   9            5
##   Female    7  55  76   60    26   9            6
##   Other     0   0   0    1     1   0            1
```

```r
chi.gen.filmpurchase.sim$expected
```

```
##
##          None   1-2    3-5    6-10   11-15    16+ I don't know
##   Male   4.74 33.18 48.190 44.6350 15.4050  7.110         4.74
##   Female 7.17 50.19 72.895 67.5175 23.3025 10.755         7.17
##   Other  0.09  0.63  0.915  0.8475  0.2925  0.135         0.09
```

```r
chi.gen.filmreuse.sim$observed
```

```
##
##          Never Once 2-4 times 5-10 times More often
##   Male      92   28        26          4          8
##   Female   143   55        27          8          6
##   Other      1    0         1          0          1
```

```r
chi.gen.filmreuse.sim$expected
```

```
##
##           Never    Once 2-4 times 5-10 times More often
##   Male    93.22 32.7850    21.330       4.74     5.9250
##   Female 141.01 49.5925    32.265       7.17     8.9625
##   Other    1.77  0.6225     0.405       0.09     0.1125
```

```r
chi.gen.bagpurhcase.sim$observed
```

```
##
##          None 1-2 3-5 6-10 11-15 16+ I don't know
##   Male     94  24  17   10     7   4            2
##   Female  137  52  26    9     7   4            4
##   Other     1   1   0    0     0   0            1
```

```r
chi.gen.bagpurhcase.sim$expected
```

```
##
##            None     1-2     3-5    6-10 11-15  16+ I don't know
##   Male    91.64 30.4150 16.9850  7.5050 5.530 3.16       2.7650
##   Female 138.62 46.0075 25.6925 11.3525 8.365 4.78       4.1825
##   Other    1.74  0.5775  0.3225  0.1425 0.105 0.06       0.0525
```

```r
chi.gen.bagreuse.sim$observed
```

```
##
##          Never Once 2-4 times 5-10 times More often
##   Male      14   24        36         32         52
##   Female    17   20        56         38        108
##   Other      0    0         1          0          2
```

```r
chi.gen.bagreuse.sim$expected
```

```
##
##            Never  Once 2-4 times 5-10 times More often
##   Male   12.2450 17.38   36.7350     27.650     63.990
##   Female 18.5225 26.29   55.5675     41.825     96.795
##   Other   0.2325  0.33    0.6975      0.525      1.215
```

```r
chi.gen.bagdisp.sim$observed
```

```
##
##          General waste bin Recycling bin Recycling centre
##   Male                  63            45                9
##   Female                80            47               20
##   Other                  0             0                0
##
##          Specialist waste collection Landfill Deposit return scheme
##   Male                             2        1                     4
##   Female                           5        1                     7
##   Other                            1        0                     0
##
##          Indefinite storage Other I don't know
##   Male                   23     9            2
##   Female                 59    14            6
##   Other                   1     0            1
```

```r
chi.gen.bagdisp.sim$expected
```

```
##
##          General waste bin Recycling bin Recycling centre
##   Male             56.4850         36.34          11.4550
##   Female           85.4425         54.97          17.3275
##   Other             1.0725          0.69           0.2175
##
##          Specialist waste collection Landfill Deposit return scheme
##   Male                          3.16    0.790                4.3450
##   Female                        4.78    1.195                6.5725
##   Other                         0.06    0.015                0.0825
##
##          Indefinite storage   Other I don't know
##   Male              32.7850  9.0850       3.5550
##   Female            49.5925 13.7425       5.3775
##   Other              0.6225  0.1725       0.0675
```

Income

```r
chi.inc.choice <- chisq.test(inc.choice)
```

```
## Warning in chisq.test(inc.choice): Chi-squared approximation may be incorrect
```

```r
chi.inc.choice.sim <- chisq.test(inc.choice, simulate.p.value = TRUE)
chi.inc.choice.sim
```

```
##
##  Pearson's Chi-squared test with simulated p-value (based on 2000
##  replicates)
##
## data:  inc.choice
## X-squared = 32.428, df = NA, p-value = 0.2089
```

```r
chi.inc.barrier <- chisq.test(inc.barrier)
```

```
## Warning in chisq.test(inc.barrier): Chi-squared approximation may be incorrect
```

```r
chi.inc.barrier.sim <- chisq.test(inc.barrier, simulate.p.value = TRUE)
chi.inc.barrier.sim
```

```
##
##  Pearson's Chi-squared test with simulated p-value (based on 2000
##  replicates)
##
## data:  inc.barrier
## X-squared = 134.43, df = NA, p-value = 0.02049
```

```r
chi.inc.barrier2 <- chisq.test(inc.barrier2)
```

```
## Warning in chisq.test(inc.barrier2): Chi-squared approximation may be incorrect
```

```r
chi.inc.barrier2.sim <- chisq.test(inc.barrier2, simulate.p.value = TRUE)
chi.inc.barrier2.sim
```

```
##
##  Pearson's Chi-squared test with simulated p-value (based on 2000
##  replicates)
##
## data:  inc.barrier2
## X-squared = 71.63, df = NA, p-value = 0.1969
```

```r
chi.inc.mainconsid <- chisq.test(inc.mainconsid)
```

```
## Warning in chisq.test(inc.mainconsid): Chi-squared approximation may be
## incorrect
```

```r
chi.inc.mainconsid.sim <- chisq.test(inc.mainconsid, simulate.p.value =
TRUE)
chi.inc.mainconsid.sim
```

```
##
##  Pearson's Chi-squared test with simulated p-value (based on 2000
##  replicates)
##
## data:  inc.mainconsid
## X-squared = 107.51, df = NA, p-value = 0.1189
```

```r
chi.inc.litter <- chisq.test(inc.litter)
```

```
## Warning in chisq.test(inc.litter): Chi-squared approximation may be incorrect
```

```r
chi.inc.litter.sim <- chisq.test(inc.litter, simulate.p.value = TRUE)
chi.inc.litter.sim
```

```
##
##  Pearson's Chi-squared test with simulated p-value (based on 2000
##  replicates)
##
## data:  inc.litter
## X-squared = 55.067, df = NA, p-value = 0.04298
```

```r
chi.inc.attitude <- chisq.test(inc.attitude)
```

```
## Warning in chisq.test(inc.attitude): Chi-squared approximation may be incorrect
```

```r
chi.inc.attitude.sim <- chisq.test(inc.attitude, simulate.p.value = TRUE)
chi.inc.attitude.sim
```

```
##
##  Pearson's Chi-squared test with simulated p-value (based on 2000
##  replicates)
##
## data:  inc.attitude
## X-squared = 68.938, df = NA, p-value = 0.08696
```

```r
chi.inc.ocean <- chisq.test(inc.ocean)
```

```
## Warning in chisq.test(inc.ocean): Chi-squared approximation may be incorrect
```

```r
chi.inc.ocean.sim <- chisq.test(inc.ocean, simulate.p.value = TRUE)
chi.inc.ocean.sim
```

```
##
##  Pearson's Chi-squared test with simulated p-value (based on 2000
##  replicates)
##
## data:  inc.ocean
## X-squared = 32.647, df = NA, p-value = 0.6237
```

```r
chi.inc.actions <- chisq.test(inc.actions)
```

```
## Warning in chisq.test(inc.actions): Chi-squared approximation may be incorrect
```

```r
chi.inc.actions.sim <- chisq.test(inc.actions, simulate.p.value = TRUE)
chi.inc.actions.sim
```

```
##
##  Pearson's Chi-squared test with simulated p-value (based on 2000
##  replicates)
##
## data:  inc.actions
## X-squared = 39.436, df = NA, p-value = 0.3228
```

```r
chi.inc.attplast <- chisq.test(inc.attplast)
```

```
## Warning in chisq.test(inc.attplast): Chi-squared approximation may be incorrect
```

```r
chi.inc.attplast.sim <- chisq.test(inc.attplast, simulate.p.value = TRUE)
chi.inc.attplast.sim
```

```
##
##  Pearson's Chi-squared test with simulated p-value (based on 2000
##  replicates)
##
## data:  inc.attplast
## X-squared = 36.308, df = NA, p-value = 0.1224
```

```r
chi.inc.zerow <- chisq.test(inc.zerow)
```

```
## Warning in chisq.test(inc.zerow): Chi-squared approximation may be incorrect
```

```r
chi.inc.zerow.sim <- chisq.test(inc.zerow, simulate.p.value = TRUE)
chi.inc.zerow.sim
```

```
##
##  Pearson's Chi-squared test with simulated p-value (based on 2000
##  replicates)
##
## data:  inc.zerow
## X-squared = 70.898, df = NA, p-value = 0.008496
```

```r
chi.inc.bottpurchase <- chisq.test(inc.bottpurchase)
```

```
## Warning in chisq.test(inc.bottpurchase): Chi-squared approximation may be
## incorrect
```

```r
chi.inc.bottpurchase.sim <- chisq.test(inc.bottpurchase, simulate.p.value =
TRUE)
chi.inc.bottpurchase.sim
```

```
##
##  Pearson's Chi-squared test with simulated p-value (based on 2000
##  replicates)
##
## data:  inc.bottpurchase
## X-squared = 74.086, df = NA, p-value = 0.05047
```

```r
chi.inc.bottreuse <- chisq.test(inc.bottreuse)
```

```
## Warning in chisq.test(inc.bottreuse): Chi-squared approximation may be incorrect
```

```r
chi.inc.bottreuse.sim <- chisq.test(inc.bottreuse, simulate.p.value = TRUE)
chi.inc.bottreuse.sim
```

```
##
##  Pearson's Chi-squared test with simulated p-value (based on 2000
##  replicates)
##
## data:  inc.bottreuse
## X-squared = 41.092, df = NA, p-value = 0.2689
```

```r
chi.inc.bottdisp <- chisq.test(inc.bottdisp)
```

```
## Warning in chisq.test(inc.bottdisp): Chi-squared approximation may be incorrect
```

```r
chi.inc.bottdisp.sim <- chisq.test(inc.bottdisp, simulate.p.value = TRUE)
chi.inc.bottdisp.sim
```

```
##
##  Pearson's Chi-squared test with simulated p-value (based on 2000
##  replicates)
##
## data:  inc.bottdisp
## X-squared = 91.26, df = NA, p-value = 0.1074
```

```r
chi.inc.tubspurchase <- chisq.test(inc.tubspurchase)
```

```
## Warning in chisq.test(inc.tubspurchase): Chi-squared approximation may be
## incorrect
```

```r
chi.inc.tubspurchase.sim <- chisq.test(inc.tubspurchase, simulate.p.value =
TRUE)
chi.inc.tubspurchase.sim
```

```
##
##  Pearson's Chi-squared test with simulated p-value (based on 2000
##  replicates)
##
## data:  inc.tubspurchase
## X-squared = 62.136, df = NA, p-value = 0.2009
```

```r
chi.inc.tubreuse <- chisq.test(inc.tubreuse)
```

```
## Warning in chisq.test(inc.tubreuse): Chi-squared approximation may be incorrect
```

```r
chi.inc.tubreuse.sim <- chisq.test(inc.tubreuse, simulate.p.value = TRUE)
chi.inc.tubreuse.sim
```

```
##
##  Pearson's Chi-squared test with simulated p-value (based on 2000
##  replicates)
##
## data:  inc.tubreuse
## X-squared = 40.216, df = NA, p-value = 0.2914
```

```r
chi.inc.tubdisp <- chisq.test(inc.tubdisp)
```

```
## Warning in chisq.test(inc.tubdisp): Chi-squared approximation may be incorrect
```

```r
chi.inc.tubdisp.sim <- chisq.test(inc.tubdisp, simulate.p.value = TRUE)
chi.inc.tubdisp.sim
```

```
##
##  Pearson's Chi-squared test with simulated p-value (based on 2000
##  replicates)
##
## data:  inc.tubdisp
## X-squared = 72.229, df = NA, p-value = 0.4398
```

```
chi.inc.filmpurchase <- chisq.test(inc.filmpurchase)
```

```
## Warning in chisq.test(inc.filmpurchase): Chi-squared approximation may be
## incorrect
```

```
chi.inc.filmpurchase.sim <- chisq.test(inc.filmpurchase, simulate.p.value =
TRUE)
chi.inc.filmpurchase.sim
```

```
##
##  Pearson's Chi-squared test with simulated p-value (based on 2000
##  replicates)
##
## data:  inc.filmpurchase
## X-squared = 52.761, df = NA, p-value = 0.5192
```

```
chi.inc.filmreuse <- chisq.test(inc.filmreuse)
```

```
## Warning in chisq.test(inc.filmreuse): Chi-squared approximation may be incorrect
```

```
chi.inc.filmreuse.sim <- chisq.test(inc.filmreuse, simulate.p.value = TRUE)
chi.inc.filmreuse.sim
```

```
##
##  Pearson's Chi-squared test with simulated p-value (based on 2000
##  replicates)
##
## data:  inc.filmreuse
## X-squared = 45.268, df = NA, p-value = 0.1434
```

```
chi.inc.filmdisp <- chisq.test(inc.filmdisp)
```

```
## Warning in chisq.test(inc.filmdisp): Chi-squared approximation may be incorrect
```

```
chi.inc.filmdisp.sim <- chisq.test(inc.filmdisp, simulate.p.value = TRUE)
chi.inc.filmdisp.sim
```

```
##
##  Pearson's Chi-squared test with simulated p-value (based on 2000
##  replicates)
##
## data:  inc.filmdisp
## X-squared = 102.17, df = NA, p-value = 0.04648
```

```
chi.inc.bagpurchase <- chisq.test(inc.bagpurchase)
```

```
## Warning in chisq.test(inc.bagpurchase): Chi-squared approximation may be
## incorrect
```

```
chi.inc.bagpurchase.sim <- chisq.test(inc.bagpurchase, simulate.p.value =
TRUE)
chi.inc.bagpurchase.sim
```

```
##
##  Pearson's Chi-squared test with simulated p-value (based on 2000
##  replicates)
##
## data:  inc.bagpurchase
## X-squared = 64.944, df = NA, p-value = 0.1454
```

```
chi.inc.bagreuse <- chisq.test(inc.bagreuse)
```

```
## Warning in chisq.test(inc.bagreuse): Chi-squared approximation may be incorrect
```

```
chi.inc.bagreuse.sim <- chisq.test(inc.bagreuse, simulate.p.value = TRUE)
chi.inc.bagreuse.sim
```

```
##
##  Pearson's Chi-squared test with simulated p-value (based on 2000
##  replicates)
##
## data:  inc.bagreuse
## X-squared = 35.784, df = NA, p-value = 0.4673
```

```
chi.inc.bagdisp <- chisq.test(inc.bagdisp)
```

```
## Warning in chisq.test(inc.bagdisp): Chi-squared approximation may be incorrect
```

```
chi.inc.bagdisp.sim <- chisq.test(inc.bagdisp, simulate.p.value = TRUE)
chi.inc.bagdisp.sim
```

```
##
##  Pearson's Chi-squared test with simulated p-value (based on 2000
##  replicates)
##
## data:  inc.bagdisp
## X-squared = 76.557, df = NA, p-value = 0.3153
```

Simulated residuals for 'income'

```
chi.inc.choice.sim$residuals
```

```
##
##                        Always As often as they can If cheaper or preferred
##   Less than £12,000 -0.37766308         -1.03566439             0.80802624
##   £12,000-14,999    -1.23715763          0.11531133             0.65975066
##   £15,000-19,999     2.88112836         -0.83163930            -0.99898416
##   £20,000-24,999    -0.56086957          0.22082242             0.45449611
##   £25,000-34,999    -0.80968221          0.76153846            -0.27811415
##   £35,000-49,999     1.10271145          0.07635417            -0.64754177
##   £50,000-74,999    -1.39568867          0.74420841            -0.29065918
##   £100,000-149,000   0.11795356          0.92912283            -0.76135089
##   £150,000 or more   0.79618656         -0.05547002            -0.14855627
##   Prefer not to say -1.23715763         -0.67090230             1.71254427
##
##                     Rarely or never
##   Less than £12,000      1.60579308
##   £12,000-14,999        -0.06900656
##   £15,000-19,999         0.58655573
##   £20,000-24,999        -0.78065376
##   £25,000-34,999        -0.45573272
##   £35,000-49,999        -0.29319774
##   £50,000-74,999         0.34020691
##   £100,000-149,000      -1.09544512
##   £150,000 or more      -0.54772256
##   Prefer not to say     -0.06900656
```

```
chi.inc.barrier.sim$residuals
```

```
##
##                     Council collection Unclear information Difficult transport
##   Less than £12,000       -0.884914987         0.192046141         1.521482118
##   £12,000-14,999          -1.074983750         2.716886942        -0.794247753
##   £15,000-19,999          -0.261403152        -0.244807834         0.505738954
##   £20,000-24,999          -0.061870650        -1.172194212        -0.299239335
##   £25,000-34,999          -0.762959385        -1.782308983        -0.014944064
##   £35,000-49,999           1.315677341         0.355457094         0.457367532
##   £50,000-74,999           0.573414639         0.009466031        -0.261046282
##   £100,000-149,000         0.652753366         0.330200330        -1.113552873
##   £150,000 or more        -0.139875721        -0.787400787        -0.556776436
##   Prefer not to say        0.334831004         0.796825499        -0.794247753
##
##                     No local facilities   No support Ends up in landfills
##   Less than £12,000        -0.352107358 -1.374772708         -0.249805119
##   £12,000-14,999            0.351382111 -1.122497216         -0.203965025
##   £15,000-19,999           -0.644200536  1.285823531         -0.525264770
##   £20,000-24,999            0.984428453 -1.438749457         -0.921421969
##   £25,000-34,999            1.617384947  1.738896398          0.825595780
##   £35,000-49,999            0.135724179 -1.849324201         -0.107827614
##   £50,000-74,999           -1.480360853  0.685160160          1.187210890
##   £100,000-149,000         -0.338061702  0.329983165          1.462252310
##   £150,000 or more         -0.591607978  4.289781139         -0.574456265
##   Prefer not to say        -0.287494454  0.659244397         -0.861916720
##
##                       Forgetting Recycling a hassle Household disagrees
##   Less than £12,000 -0.385758375        0.927105069         0.655562281
```

```
##   £12,000-14,999     0.188982237        0.358568583        -0.591607978
##   £15,000-19,999    -0.472455591        0.338648106         1.366332712
##   £20,000-24,999     0.663488803       -0.139875721         0.560473402
##   £25,000-34,999    -0.693375245       -1.140175425        -0.806225775
##   £35,000-49,999     0.573539335        0.072547625        -0.974679434
##   £50,000-74,999     1.304372987       -0.117851130         0.583333333
##   £100,000-149,000  -1.000000000       -0.632455532        -0.447213595
##   £150,000 or more  -0.500000000       -0.316227766        -0.223606798
##   Prefer not to say -0.566946710        0.358568583        -0.591607978
##
##                     Recycling not important        Other
##   Less than £12,000            -0.724568837 -0.648074070
##   £12,000-14,999               -0.591607978  3.250494468
##   £15,000-19,999                0.239460372  0.466156183
##   £20,000-24,999                1.879234349  0.796186563
##   £25,000-34,999               -0.806225775 -0.721110255
##   £35,000-49,999               -0.974679434 -0.871779789
##   £50,000-74,999               -0.750000000 -0.670820393
##   £100,000-149,000             -0.447213595 -0.400000000
##   £150,000 or more              4.248529157 -0.200000000
##   Prefer not to say             1.098700531 -0.529150262
##
##                     Already doing everything
##   Less than £12,000              1.232410072
##   £12,000-14,999                -0.996246087
##   £15,000-19,999                 0.007509393
##   £20,000-24,999                 0.954976128
##   £25,000-34,999                 1.686178632
##   £35,000-49,999                -1.163816595
##   £50,000-74,999                -1.347593697
##   £100,000-149,000              -0.185434530
##   £150,000 or more              -0.754983444
##   Prefer not to say              0.005006262
```

```
chi.inc.barrier2.sim$residuals
```

```
##
##                     Limited alternatives No SUP-free alternatives
##   Less than £12,000          -0.79698506              -0.30962614
##   £12,000-14,999             -0.11952286              -0.79942206
##   £15,000-19,999             -1.10890654               0.71401372
##   £20,000-24,999             -0.73046210              -1.20742058
##   £25,000-34,999              0.38005848              -0.91752668
##   £35,000-49,999              0.94311913               0.70918346
##   £50,000-74,999              0.27498597               0.46485995
##   £100,000-149,000           -0.84327404               0.32539569
##   £150,000 or more           -0.94868330               1.24735013
##   Prefer not to say           2.27093436               0.43045803
##
##                     Alternatives expensive Limited functioning
##   Less than £12,000             0.02510482         -0.04694579
##   £12,000-14,999                1.25037809          1.37386650
##   £15,000-19,999                0.16740035         -0.05749662
##   £20,000-24,999                0.39181198          0.71537113
```

```
##   £25,000-34,999            -1.21835510        -0.91931927
##   £35,000-49,999             0.21151086        -0.15061726
##   £50,000-74,999            -1.15204422         0.29241357
##   £100,000-149,000           1.41004798         0.35228194
##   £150,000 or more           0.16269784        -0.62449980
##   Prefer not to say         -0.38946203        -1.04704363
##
##                     Forgetting reusables Reducing not important       Other
##   Less than £12,000           2.34216018            -1.16833214 -0.91651514
##   £12,000-14,999             -0.47809144             0.09434564  0.58797473
##   £15,000-19,999             -0.91634193             0.66566087  1.55011520
##   £20,000-24,999             -0.27975144             1.23088053 -0.95916630
##   £25,000-34,999              1.66641024            -0.53076923 -0.03922323
##   £35,000-49,999             -0.58038100            -0.29905384 -0.42177569
##   £50,000-74,999              0.23570226             0.44445780  0.10540926
##   £100,000-149,000           -0.47434165            -0.72111026 -0.56568542
##   £150,000 or more           -0.63245553             2.41294585 -0.28284271
##   Prefer not to say          -1.67332005            -0.95393920  0.58797473
##
##                     No barriers
##   Less than £12,000  0.39036003
##   £12,000-14,999    -1.67332005
##   £15,000-19,999     0.27888668
##   £20,000-24,999     1.58525817
##   £25,000-34,999     2.10493925
##   £35,000-49,999    -1.66859538
##   £50,000-74,999    -0.23570226
##   £100,000-149,000  -0.47434165
##   £150,000 or more  -0.63245553
##   Prefer not to say -0.47809144
```

```
chi.inc.mainconsid.sim$residuals
```

```
##
##                     Value for money        Price      Quality Deals/discounts
##   Less than £12,000    -0.140035153  0.568716872  0.578997492     0.768638966
##   £12,000-14,999        0.232141241 -0.303174175 -1.273200283     0.133424877
##   £15,000-19,999       -0.171507335 -1.094369226  0.574820381     0.447220421
##   £20,000-24,999       -0.996127870  1.750047063 -0.037721677     0.005783149
##   £25,000-34,999        0.643243438  0.673039197 -0.721401123    -1.294549338
##   £35,000-49,999        0.082018973  0.132773740  0.313034232    -1.322768536
##   £50,000-74,999        0.714013720 -2.092536374  0.031782086     2.967379632
##   £100,000-149,000      0.110003820 -0.446752313  1.854846232    -1.019803903
##   £150,000 or more     -1.090871211  0.030460385  0.127920430     1.451259400
##   Prefer not to say    -0.114338223  0.848120160 -1.273200283    -1.349073756
##
##                     Use-by-dates/longevity  Convenience
##   Less than £12,000           -0.387649466 -0.048795004
##   £12,000-14,999               0.020203051  1.553797192
##   £15,000-19,999              -0.811489211  0.338648106
##   £20,000-24,999               0.307363058 -1.072380529
##   £25,000-34,999              -0.607824440 -1.140175425
##   £35,000-49,999              -0.404671964  0.072547625
##   £50,000-74,999               0.338648106 -0.117851130
```

```
##   £100,000-149,000            0.587974732 -0.632455532
##   £150,000 or more           -0.374165739 -0.316227766
##   Prefer not to say           2.040508140  1.553797192
##
##                      Ease of recycling packaging Sustainability        Brand
##   Less than £12,000                 -0.861916720   -1.168332145 -0.648074070
##   £12,000-14,999                     0.370678563    0.094345635  1.360672103
##   £15,000-19,999                    -1.324235738    1.364517429  1.726037760
##   £20,000-24,999                    -0.333208145    0.413019714 -0.678232998
##   £25,000-34,999                     1.856720923    0.238461538 -0.721110255
##   £35,000-49,999                     0.890191098   -0.299053839  0.275298881
##   £50,000-74,999                     0.333711906   -0.382440432 -0.670820393
##   £100,000-149,000                  -0.938083152   -0.721110255 -0.400000000
##   £150,000 or more                  -0.469041576   -0.360555128 -0.200000000
##   Prefer not to say                 -0.435144401    0.094345635 -0.529150262
##
##                            Ethics        Other
##   Less than £12,000 -0.724568837 -0.458257569
##   £12,000-14,999     1.098700531 -0.374165739
##   £15,000-19,999     0.239460372  3.002234617
##   £20,000-24,999    -0.758287544 -0.479583152
##   £25,000-34,999    -0.806225775 -0.509901951
##   £35,000-49,999     0.051298918 -0.616441400
##   £50,000-74,999    -0.750000000 -0.474341649
##   £100,000-149,000  -0.447213595 -0.282842712
##   £150,000 or more   4.248529157 -0.141421356
##   Prefer not to say  1.098700531 -0.374165739
```

```
chi.inc.litter.sim$residuals
```

```
##
##                      Strongly agree       Agree Neither agree nor disagree
##   Less than £12,000     -0.03871384 -0.72981761                -0.66340961
##   £12,000-14,999        -0.31897079  0.78990545                -0.88778777
##   £15,000-19,999        -0.23898862  0.29398737                -0.12027534
##   £20,000-24,999        -0.64904767 -0.14596009                 1.98679088
##   £25,000-34,999         0.74013747 -1.10260849                 0.80384598
##   £35,000-49,999         0.02267480  0.76063822                -0.96743911
##   £50,000-74,999         0.12183699  0.18153816                -0.39417270
##   £100,000-149,000      -0.34973152  0.98994949                -0.76921916
##   £150,000 or more       0.96556312 -0.48711800                -0.72801099
##   Prefer not to say      0.25575135 -0.69487923                 1.18890876
##
##                         Disagree Strongly disagree
##   Less than £12,000  1.26566376        5.52405993
##   £12,000-14,999     0.58797473       -0.52915026
##   £15,000-19,999     0.65924440       -0.79372539
##   £20,000-24,999    -0.95916630       -0.67823300
##   £25,000-34,999    -0.03922323       -0.72111026
##   £35,000-49,999    -0.42177569       -0.87177979
##   £50,000-74,999     0.10540926       -0.67082039
##   £100,000-149,000  -0.56568542       -0.40000000
##   £150,000 or more  -0.28284271       -0.20000000
##   Prefer not to say -0.74833148       -0.52915026
```

```r
chi.inc.attitude.sim$residuals
```

```
##
##                      1 (Not concerned)           2           3           4
##   Less than £12,000        0.69190966 -0.75907212  1.78085215  0.12100094
##   £12,000-14,999           0.08001323  0.50709255 -0.51354476  0.39371282
##   £15,000-19,999           0.84741281 -1.21138777  0.21348508  0.44311125
##   £20,000-24,999           0.50791640  0.46156633 -0.32927988 -0.48146404
##   £25,000-34,999          -0.80597837 -0.37210420  1.15650538  0.16555301
##   £35,000-49,999          -0.94041797 -0.41039134 -1.35790842  1.37746044
##   £50,000-74,999          -0.59529188  1.83333333 -0.28519237 -0.77205029
##   £100,000-149,000        -0.07698004 -0.89442719 -1.28062485 -1.12359501
##   £150,000 or more        -0.51961524 -0.44721360 -0.64031242 -0.85440037
##   Prefer not to say        0.80740619  1.35224681  0.66701791 -0.93340905
##
##                               5           6 7 (Very concerned
##   Less than £12,000 -1.14747553 -1.60008503        1.39505313
##   £12,000-14,999    -1.05490856 -0.46467017        1.44239994
##   £15,000-19,999    -0.69737244  0.06060915        0.34353690
##   £20,000-24,999     1.35054019 -0.96149470       -0.68692212
##   £25,000-34,999    -1.25205364  1.37872373        0.01001671
##   £35,000-49,999     0.07305496  1.03007409       -0.58274843
##   £50,000-74,999     0.04886629  1.47411528       -0.99152000
##   £100,000-149,000   3.01580631 -1.49666295        0.14446302
##   £150,000 or more   0.80546380  1.92428094       -0.83066239
##   Prefer not to say  0.71507225 -1.47482272       -0.37766308
```

```r
chi.inc.ocean.sim$residuals
```

```
##
##                          Always Most of the time   Sometimes      Rarely
##   Less than £12,000  1.41952461      -0.27840467 -0.43643578 -0.51793240
##   £12,000-14,999     1.44807365      -1.21564888 -0.26726124  0.39036003
##   £15,000-19,999     0.58240886       0.64735774 -0.26726124 -0.79698506
##   £20,000-24,999    -0.12515437      -0.56674863  1.04257207 -0.34262414
##   £25,000-34,999    -0.93533860       0.67447108 -0.19611614  0.42966892
##   £35,000-49,999    -0.32105263       0.95383309 -0.48666426  0.17770466
##   £50,000-74,999    -0.77803358      -0.49699954  0.10540926  0.86602540
##   £100,000-149,000  -0.87177979       0.41184388  0.35355339 -0.90369611
##   £150,000 or more   1.85826744      -1.01980390  0.70710678 -0.77459667
##   Prefer not to say -1.15325626      -0.47439956  0.26726124  0.87831007
##
##                           Never
##   Less than £12,000  1.65788583
##   £12,000-14,999     1.65922428
##   £15,000-19,999     0.19709018
##   £20,000-24,999    -1.39821315
##   £25,000-34,999    -0.81393408
##   £35,000-49,999    -0.68439031
##   £50,000-74,999     0.06327138
##   £100,000-149,000   0.38805700
##   £150,000 or more  -0.41231056
##   Prefer not to say -0.17417271
```

```r
chi.inc.actions.sim$residuals
```

```
##
##                     Yes, definitely Yes, probably No, probably not
##   Less than £12,000     0.541261908  -0.905481383      0.698871896
##   £12,000-14,999        0.019030844  -0.101189633      1.147016931
##   £15,000-19,999       -0.711542126   1.261223938     -0.681101344
##   £20,000-24,999        0.165798925  -0.632643564      1.373814408
##   £25,000-34,999       -0.572557699   0.435483168     -1.518408215
##   £35,000-49,999        1.351500278  -0.214139225     -1.458898329
##   £50,000-74,999        0.006254889  -0.752352332      1.437871399
##   £100,000-149,000     -1.124503020   1.215605337     -0.548994853
##   £150,000 or more     -0.352456170   0.788696320     -0.655743852
##   Prefer not to say    -0.298149895  -0.374675127      0.570626514
##
##                     No, definitely not I don't know
##   Less than £12,000         0.786259158 -0.312411160
##   £12,000-14,999           -0.877496439 -0.953939201
##   £15,000-19,999            0.203229695 -1.430908802
##   £20,000-24,999           -0.235613739 -0.404841106
##   £25,000-34,999            1.312899956  1.776923077
##   £35,000-49,999           -0.062254302 -0.935338602
##   £50,000-74,999           -0.213496623  0.444457799
##   £100,000-149,000         -0.663324958  0.665640235
##   £150,000 or more         -0.331662479 -0.360555128
##   Prefer not to say        -0.877496439  2.190915309
```

```r
chi.inc.bottpurchase.sim$residuals
```

```
##
##                            None         1-2         3-5        6-10       11-15
##   Less than £12,000  0.65924440  0.16215121 -0.28525727  0.80000804 -0.70445236
##   £12,000-14,999     0.17457431  1.74698011 -1.10360912 -0.95783701 -0.28614629
##   £15,000-19,999    -0.64737974  1.16734438 -1.32890211 -0.77769335  0.58240886
##   £20,000-24,999     0.52777905 -0.44214826 -0.10444377  1.19635659 -0.80166446
##   £25,000-34,999    -0.44835883 -1.13975923  0.85774649  0.76734280  0.33723092
##   £35,000-49,999     1.80136401 -0.98589554  1.09794495 -0.81607254  0.73157895
##   £50,000-74,999    -1.16189500 -0.61451258 -0.27687249  1.62027785  0.58993755
##   £100,000-149,000  -0.69282032  0.22213299  1.14030632 -0.55798867 -0.87177979
##   £150,000 or more  -0.34641016 -0.31611233 -0.29371526 -0.86023253 -0.43588989
##   Prefer not to say -0.91651514  0.77822959  0.20243718 -1.39721179 -0.28614629
##
##                             16+ I don't know
##   Less than £12,000 -1.21243557  -0.04879500
##   £12,000-14,999     0.02020305   0.35856858
##   £15,000-19,999     1.88225091   0.33864811
##   £20,000-24,999    -0.48074735  -0.13987572
##   £25,000-34,999    -1.34907376   0.61394061
##   £35,000-49,999     0.20846738  -1.37840488
##   £50,000-74,999    -0.45817097  -0.11785113
##   £100,000-149,000  -0.74833148  -0.63245553
##   £150,000 or more   4.97105910  -0.31622777
##   Prefer not to say  0.02020305   1.55379719
```

```r
chi.inc.bottreuse.sim$residuals
```

```
##
##                          Never         Once    2-4 times   5-10 times
##   Less than £12,000  0.199605580 -1.113014963 -0.148083612 -0.402628166
##   £12,000-14,999    -0.435104465  0.642034105 -0.342762538  0.005006262
##   £15,000-19,999    -1.549779305 -0.122513754 -0.292291031 -0.659992173
##   £20,000-24,999     0.992144590 -1.303087446 -0.995684148  1.345559411
##   £25,000-34,999     0.582602597 -0.198008735 -0.188028722  0.951460274
##   £35,000-49,999     0.138855856  0.129899974  0.502953334  0.355526218
##   £50,000-74,999    -0.349407054  1.678969742  0.390468369  0.232003310
##   £100,000-149,000  -0.498448296 -0.393892771  0.810014834 -0.847700709
##   £150,000 or more   1.530948339 -0.812403840  0.625120143 -0.754983444
##   Prefer not to say  0.462018143  1.107276210  0.322795788 -1.496872261
##
##                     More often
##   Less than £12,000  1.376866249
##   £12,000-14,999     0.262745600
##   £15,000-19,999     2.547770862
##   £20,000-24,999     0.384778860
##   £25,000-34,999    -0.951370010
##   £35,000-49,999    -1.210483372
##   £50,000-74,999    -1.923918148
##   £100,000-149,000   0.524218652
##   £150,000 or more  -0.877496439
##   Prefer not to say -0.598715384
```

```r
chi.inc.bottdisp.sim$residuals
```

```
##
##                     General waste bin Recycling bin Recycling centre
##   Less than £12,000       2.473651272  -0.415774888     -0.048795004
##   £12,000-14,999          0.807406194   0.008347839      0.358568583
##   £15,000-19,999         -0.607373128  -1.204871387      0.338648106
##   £20,000-24,999          0.507916397  -0.280054532     -0.139875721
##   £25,000-34,999         -0.272217861   0.514553669     -1.140175425
##   £35,000-49,999         -0.940417974   0.592832673      1.523500125
##   £50,000-74,999         -0.595291885   0.510326958     -1.060660172
##   £100,000-149,000       -0.076980036  -0.309208273     -0.632455532
##   £150,000 or more       -0.519615242  -0.154604137      2.846049894
##   Prefer not to say      -0.647379741   0.634435742     -0.836660027
##
##                     Specialist waste collection     Landfill
##   Less than £12,000                -0.857321410 -0.724568837
##   £12,000-14,999                   -0.700000000 -0.591607978
##   £15,000-19,999                    0.854761905  2.493205051
##   £20,000-24,999                    1.331894928 -0.758287544
##   £25,000-34,999                   -0.953939201  0.434121571
##   £35,000-49,999                   -0.286146290 -0.974679434
##   £50,000-74,999                   -0.887411967  0.583333333
##   £100,000-149,000                  3.250494468 -0.447213595
##   £150,000 or more                 -0.264575131 -0.223606798
##   Prefer not to say                -0.700000000 -0.591607978
```

```
##
##                     Deposit return scheme Indefinite storage       Other
##   Less than £12,000          -0.648074070       -1.296148140  1.723921333
##   £12,000-14,999             -0.529150262       -0.113389342 -0.374165739
##   £15,000-19,999              1.726037760        3.452075520 -0.561248608
##   £20,000-24,999              0.796186563        0.117953565 -0.479583152
##   £25,000-34,999             -0.721110255       -0.748845265  1.451259400
##   £35,000-49,999             -0.871779789       -1.170020243 -0.616441400
##   £50,000-74,999              0.819891592       -0.596284794 -0.474341649
##   £100,000-149,000           -0.400000000        0.450000000 -0.282842712
##   £150,000 or more           -0.200000000       -0.400000000 -0.141421356
##   Prefer not to say          -0.529150262       -0.113389342 -0.374165739
##
##                     I don't know
##   Less than £12,000  2.762029964
##   £12,000-14,999    -0.264575131
##   £15,000-19,999    -0.396862697
##   £20,000-24,999    -0.339116499
##   £25,000-34,999    -0.360555128
##   £35,000-49,999    -0.435889894
##   £50,000-74,999    -0.335410197
##   £100,000-149,000  -0.200000000
##   £150,000 or more  -0.100000000
##   Prefer not to say -0.264575131
```

```
chi.inc.tubspurchase.sim$residuals
```

```
##
##                             None          1-2          3-5         6-10
##   Less than £12,000  1.550115203 -0.100965358  0.770338613 -0.644357105
##   £12,000-14,999     0.174574312  1.251115904 -0.003160698 -0.799422060
##   £15,000-19,999     0.807406194 -0.002424643 -1.374376719  0.987320408
##   £20,000-24,999    -1.174734012  0.732080616 -0.356328786 -0.567727556
##   £25,000-34,999    -1.248999600  0.523085302 -0.136839764 -0.015041421
##   £35,000-49,999     0.476831649 -1.439325161  0.542927221  1.455692360
##   £50,000-74,999     0.559430928 -0.903696114 -0.769773022  0.464859948
##   £100,000-149,000  -0.692820323 -0.153960072  0.953315891 -1.301582747
##   £150,000 or more  -0.346410162 -0.076980036  0.476657946 -0.921954446
##   Prefer not to say -0.916515139  0.887419420  0.945048614 -0.799422060
##
##                            11-15          16+ I don't know
##   Less than £12,000 -0.598856327 -1.168332145 -0.048795004
##   £12,000-14,999    -0.021055872 -0.953939201 -0.836660027
##   £15,000-19,999    -1.201354486  2.762230545  0.338648106
##   £20,000-24,999     0.364144476  0.413019714  0.792629087
##   £25,000-34,999     0.633482229 -1.300000000  0.613940614
##   £35,000-49,999    -0.643281627 -0.299053839 -0.652928625
##   £50,000-74,999     2.622853072 -0.382440432 -0.117851130
##   £100,000-149,000   0.779920420  0.665640235 -0.632455532
##   £150,000 or more  -0.538516481  2.412945854 -0.316227766
##   Prefer not to say -1.424780685 -0.953939201  0.358568583
```

```r
chi.inc.tubreuse.sim$residuals
```

```
##
##                           Never         Once    2-4 times   5-10 times
##   Less than £12,000 -0.555158198 -0.628961314  0.568716872 -0.036469840
##   £12,000-14,999    -0.614596076  0.667017910  0.080590603 -0.526069167
##   £15,000-19,999     0.126629375 -1.754119370 -1.094369226  0.286194861
##   £20,000-24,999    -0.005663402  0.591782719 -0.046408434 -0.646626582
##   £25,000-34,999     0.735078553 -1.009235031 -1.016598954  1.988414721
##   £35,000-49,999    -1.054514924  1.508389036  1.064519282 -1.813450452
##   £50,000-74,999     1.197684849  0.180427826  0.329205542  0.968913362
##   £100,000-149,000  -0.921865536  0.281112771  1.076266935  0.446441872
##   £150,000 or more   1.779712631  0.921425195 -0.984885780  0.551487018
##   Prefer not to say  0.353271761  0.076736574 -0.303174175 -0.526069167
##
##                      More often
##   Less than £12,000  0.254198839
##   £12,000-14,999     0.312909073
##   £15,000-19,999     1.575607807
##   £20,000-24,999     0.136859845
##   £25,000-34,999    -0.368771568
##   £35,000-49,999     0.159233072
##   £50,000-74,999    -1.767049811
##   £100,000-149,000  -0.719168129
##   £150,000 or more  -1.195826074
##   Prefer not to say  0.312909073
```

```r
chi.inc.tubdisp.sim$residuals
```

```
##
##                     General waste bin Recycling bin Recycling centre
##   Less than £12,000      -0.675541701   0.391976918     -0.598856327
##   £12,000-14,999         -0.008151391  -0.200355135     -1.424780685
##   £15,000-19,999         -1.506648855  -0.040331228     -0.733446215
##   £20,000-24,999          0.352959465   0.149209746     -0.183441202
##   £25,000-34,999         -0.352907677   0.108833339      1.663534634
##   £35,000-49,999          0.163274330   0.143722037     -0.643281627
##   £50,000-74,999          2.033457233  -0.356621337      0.961943475
##   £100,000-149,000       -0.237232101  -0.151454246      1.708397111
##   £150,000 or more        0.150965882  -0.764155514     -0.538516481
##   Prefer not to say       0.399418182   0.059846339     -0.021055872
##
##                     Specialist waste collection     Landfill
##   Less than £12,000                 0.659244397 -0.561248608
##   £12,000-14,999                    1.265663763 -0.458257569
##   £15,000-19,999                    0.807406194  0.767399581
##   £20,000-24,999                   -1.174734012  1.115146055
##   £25,000-34,999                   -1.248999600 -0.624499800
##   £35,000-49,999                    0.476831649  0.569548914
##   £50,000-74,999                   -1.161895004 -0.580947502
##   £100,000-149,000                  0.750555350 -0.346410162
##   £150,000 or more                  2.540341184 -0.173205081
##   Prefer not to say                -0.916515139 -0.458257569
```

```
##
##                      Deposit return scheme Indefinite storage        Other
##   Less than £12,000           -0.648074070       -0.195238095  2.641949651
##   £12,000-14,999              -0.529150262        1.201416398 -0.700000000
##   £15,000-19,999               1.726037760        2.093730518 -0.097619048
##   £20,000-24,999              -0.678232998        0.077352678 -0.897217922
##   £25,000-34,999              -0.721110255       -0.196861827 -0.953939201
##   £35,000-49,999               1.422377550       -0.700912402 -0.286146290
##   £50,000-74,999              -0.670820393       -1.713662806  0.239460372
##   £100,000-149,000            -0.400000000       -0.524631390 -0.529150262
##   £150,000 or more            -0.200000000        0.894959430 -0.264575131
##   Prefer not to say           -0.529150262       -0.548219133  0.728571429
##
##                      I don't know
##   Less than £12,000 -0.793725393
##   £12,000-14,999     0.894959430
##   £15,000-19,999    -0.972111105
##   £20,000-24,999     0.373196145
##   £25,000-34,999     1.381377982
##   £35,000-49,999    -0.131122014
##   £50,000-74,999    -0.821583836
##   £100,000-149,000  -0.489897949
##   £150,000 or more  -0.244948974
##   Prefer not to say  0.894959430
```

```r
chi.inc.filmpurchase.sim$residuals
```

```
##
##                              None          1-2          3-5         6-10
##   Less than £12,000  0.659244397  0.397326668  0.332485260 -0.831746065
##   £12,000-14,999     2.356753215  0.461880215  0.499601715 -1.034676886
##   £15,000-19,999     1.534799161 -0.063233601 -0.961562205 -0.426078282
##   £20,000-24,999    -1.174734012  0.109393207 -0.008009260  0.001387017
##   £25,000-34,999    -1.248999600  0.629436634  0.035154137  0.341790823
##   £35,000-49,999    -0.847700709 -0.991239825  1.416536139  0.546014906
##   £50,000-74,999    -0.301232038 -0.146385011 -1.005472277  0.361103547
##   £100,000-149,000  -0.692820323 -0.196396101 -0.398357283  1.166493877
##   £150,000 or more  -0.346410162  0.174574312 -0.199178641 -0.122293713
##   Prefer not to say  0.174574312  0.049487166 -0.184784196  0.032000316
##
##                            11-15          16+ I don't know
##   Less than £12,000  0.941386632 -1.374772708 -0.231626410
##   £12,000-14,999     0.163411434 -1.122497216 -0.916515139
##   £15,000-19,999    -0.864466659  2.473651272  1.534799161
##   £20,000-24,999     0.243178965  0.646394684 -0.323477482
##   £25,000-34,999    -0.475203683 -0.875985404  0.352281938
##   £35,000-49,999    -0.885335622 -0.227109990 -0.847700709
##   £50,000-74,999     2.202053238 -0.720296578 -0.301232038
##   £100,000-149,000  -0.448358831  0.329983165 -0.692820323
##   £150,000 or more  -0.624499800  1.932758535 -0.346410162
##   Prefer not to say -0.441816099 -0.231626410  1.265663763
```

```r
chi.inc.filmreuse.sim$residuals
```

```
##
##                          Never        Once   2-4 times  5-10 times  More often
##   Less than £12,000 -0.35757681 -0.58093886  0.97850802 -0.23162641  1.13546718
##   £12,000-14,999     0.11809628 -1.16578444  0.62750029  1.26566376 -0.04879500
##   £15,000-19,999    -0.51995168 -0.29663198  0.51263002  0.08001323  1.71595763
##   £20,000-24,999    -0.02687343  0.79462739 -1.28812863  0.52777905  0.20938142
##   £25,000-34,999     1.68262848 -2.37151969 -0.38497419  0.35228194 -0.68030913
##   £35,000-49,999    -0.57345378  2.32426613 -0.39336604 -0.84770071 -1.68819430
##   £50,000-74,999     0.08733338  0.21680564 -0.84186986  0.55943093  0.24056261
##   £100,000-149,000  -0.79415236 -0.72444412  2.61278906 -0.69282032  0.51639778
##   £150,000 or more  -0.23434004  0.18659924  0.62598071 -0.34641016 -0.38729833
##   Prefer not to say  0.36413020  0.90856510 -0.91553321 -0.91651514 -1.02469508
```

```r
chi.inc.filmdisp.sim$residuals
```

```
##
##                     General waste bin Recycling bin Recycling centre
##   Less than £12,000       -0.53555104    0.10939321       0.16092346
##   £12,000-14,999          -0.35988168    1.00878132      -0.17417271
##   £15,000-19,999          -0.11415602   -0.65413164       0.19709018
##   £20,000-24,999          -0.08604423    0.43656158       0.03218393
##   £25,000-34,999          -0.07666866    0.58988123      -0.14126129
##   £35,000-49,999           0.09160383   -0.11480757      -0.12797542
##   £50,000-74,999           0.21748756    0.82371258      -0.65983014
##   £100,000-149,000         0.43000658   -1.39704657       1.60073513
##   £150,000 or more        -0.39929183   -0.95916630      -0.41231056
##   Prefer not to say        0.80102697   -0.96149470      -0.17417271
##
##                     Specialist waste collection    Landfill
##   Less than £12,000                 -0.79372539 -0.56124861
##   £12,000-14,999                     0.89495943 -0.45825757
##   £15,000-19,999                     2.11395589  0.76739958
##   £20,000-24,999                    -0.83066239 -0.58736701
##   £25,000-34,999                    -0.88317609 -0.62449980
##   £35,000-49,999                    -0.13112201 -0.75498344
##   £50,000-74,999                    -0.82158384 -0.58094750
##   £100,000-149,000                  -0.48989795  2.54034118
##   £150,000 or more                   3.83753393  5.60029761
##   Prefer not to say                 -0.64807407 -0.45825757
##
##                     Deposit return scheme Indefinite storage       Other
##   Less than £12,000           -0.45825757         1.22049300  0.46615618
##   £12,000-14,999              -0.37416574        -0.45825757 -0.64807407
##   £15,000-19,999               1.22049300        -0.68738635  1.08526689
##   £20,000-24,999              -0.47958315         1.11514606  0.37319614
##   £25,000-34,999              -0.50990195        -0.62449980  0.24910095
##   £35,000-49,999               1.00577281         0.56954891 -1.06770783
##   £50,000-74,999              -0.47434165        -0.58094750 -0.82158384
##   £100,000-149,000            -0.28284271        -0.34641016 -0.48989795
##   £150,000 or more            -0.14142136        -0.17320508 -0.24494897
##   Prefer not to say           -0.37416574        -0.45825757  0.89495943
```

```
##
##                      I don't know
##   Less than £12,000   2.98591934
##   £12,000-14,999     -0.64807407
##   £15,000-19,999     -0.97211110
##   £20,000-24,999     -0.83066239
##   £25,000-34,999      0.24910095
##   £35,000-49,999      0.80546380
##   £50,000-74,999     -0.82158384
##   £100,000-149,000   -0.48989795
##   £150,000 or more   -0.24494897
##   Prefer not to say  -0.64807407
```

```
chi.inc.bagpurchase.sim$residuals
```

```
##
##                            None         1-2         3-5        6-10       11-15
##   Less than £12,000 -0.07293968  1.37686625 -0.71299052 -1.41244469  0.43713663
##   £12,000-14,999    -1.30028417  1.55493708  1.14701693  0.58096368  0.02020305
##   £15,000-19,999    -0.58562417 -0.32376575 -1.83388218  1.73855549  1.88225091
##   £20,000-24,999    -0.71245085  0.38477886  0.92412066 -0.12515437 -0.48074735
##   £25,000-34,999     0.51713350  0.94504861 -0.24954341 -0.93533860 -1.34907376
##   £35,000-49,999     1.04228215 -1.47192686 -0.05947547 -0.32105263 -0.40467196
##   £50,000-74,999    -0.60679423  0.11467062  0.98320851  0.58993755  0.33864811
##   £100,000-149,000   0.23635158 -1.18519000  1.73848370 -0.87177979  0.58797473
##   £150,000 or more  -0.86662246  0.26210933  0.86924185  1.85826744 -0.37416574
##   Prefer not to say  1.67746583 -1.46017637 -1.15854474 -0.28614629 -0.98994949
##
##                             16+ I don't know
##   Less than £12,000 -0.91651514   0.30910228
##   £12,000-14,999    -0.74833148  -0.70000000
##   £15,000-19,999     2.44098601   0.85476190
##   £20,000-24,999     1.12597784   0.21733850
##   £25,000-34,999    -1.01980390  -0.95393920
##   £35,000-49,999    -0.42177569   0.58096368
##   £50,000-74,999     0.10540926  -0.88741197
##   £100,000-149,000  -0.56568542  -0.52915026
##   £150,000 or more  -0.28284271  -0.26457513
##   Prefer not to say -0.74833148   0.72857143
```

```
chi.inc.bagreuse.sim$residuals
```

```
##
##                           Never        Once   2-4 times  5-10 times  More often
##   Less than £12,000  0.41293413 -0.28845011 -1.20483856 -0.86681056  1.45236129
##   £12,000-14,999    -0.79424775 -0.04558423  0.19204614  0.49692935 -0.10096536
##   £15,000-19,999    -0.39938669  0.78632798 -0.16918351  0.59480937 -0.49789780
##   £20,000-24,999    -0.29923933 -1.36033593  0.70482376 -0.37007637  0.54908796
##   £25,000-34,999     0.48319142 -0.30104712 -0.02588388 -0.69614322  0.42273922
##   £35,000-49,999     0.04532471  0.91306309 -0.15938833  0.19194297 -0.50108395
##   £50,000-74,999    -0.79652583  0.92140648 -0.45214542  2.18263348 -1.22391858
##   £100,000-149,000  -0.21552636  0.18090681  1.18212493 -1.07570575 -0.18856181
##   £150,000 or more   1.23927658 -0.66332496  2.14649001 -0.83666003 -1.27279221
##   Prefer not to say  1.24228495 -1.18519000 -0.19988476 -0.85833251  0.78990545
```

```
chi.inc.bagdisp.sim$residuals
```

```
##
##                     General waste bin Recycling bin Recycling centre
##   Less than £12,000      -0.520010822   0.431137933     -1.171924582
##   £12,000-14,999         -0.003160698   0.614726117     -1.424780685
##   £15,000-19,999         -1.163663539  -0.654131637      0.202370327
##   £20,000-24,999         -0.109734470   1.358874771     -0.183441202
##   £25,000-34,999          0.327023843  -1.723378497      0.633482229
##   £35,000-49,999          1.310315518  -0.114807570      0.208747018
##   £50,000-74,999          0.476822965  -0.108792228      0.408306943
##   £100,000-149,000       -1.137289134   1.730669637     -0.148556271
##   £150,000 or more       -0.359584064  -0.959166305      3.175390283
##   Prefer not to say       0.312909073  -0.173384289     -0.021055872
##
##                     Specialist waste collection     Landfill
##   Less than £12,000                -0.916515139 -0.458257569
##   £12,000-14,999                    0.587974732 -0.374165739
##   £15,000-19,999                    0.659244397  1.220493005
##   £20,000-24,999                   -0.959166305 -0.479583152
##   £25,000-34,999                   -0.039223227 -0.509901951
##   £35,000-49,999                    0.389331411 -0.616441400
##   £50,000-74,999                    1.159501809 -0.474341649
##   £100,000-149,000                 -0.565685425  3.252691193
##   £150,000 or more                 -0.282842712 -0.141421356
##   Prefer not to say                -0.748331477 -0.374165739
##
##                     Deposit return scheme Indefinite storage        Other
##   Less than £12,000           0.786259158        0.096540860  1.019930782
##   £12,000-14,999              0.262109326        0.908565096 -1.268857754
##   £15,000-19,999              2.482441224       -0.020052045  1.249154994
##   £20,000-24,999             -1.124722188        0.147273101 -1.011470135
##   £25,000-34,999             -0.359584064        0.368361852  1.162413013
##   £35,000-49,999             -1.445683229       -0.949348139 -0.176995003
##   £50,000-74,999              0.685436527       -0.764955748 -0.365231050
##   £100,000-149,000           -0.663324958       -0.175622816  0.083405766
##   £150,000 or more           -0.331662479        0.186599242 -0.479583152
##   Prefer not to say          -0.877496439        0.908565096 -1.268857754
##
##                     I don't know
##   Less than £12,000  1.085266895
##   £12,000-14,999    -0.793725393
##   £15,000-19,999     0.489254012
##   £20,000-24,999    -0.034403123
##   £25,000-34,999     0.767335271
##   £35,000-49,999    -0.542950570
##   £50,000-74,999    -1.006230590
##   £100,000-149,000  -0.600000000
##   £150,000 or more  -0.300000000
##   Prefer not to say  0.466156183
```

Simulated observed and expected values for 'income'

```r
chi.inc.choice.sim$observed
```

```
##
##                     Always As often as they can If cheaper or preferred
##   Less than £12,000      4                   17                      15
##   £12,000-14,999         1                   15                      10
##   £15,000-19,999        15                   28                      14
##   £20,000-24,999         4                   25                      15
##   £25,000-34,999         4                   31                      14
##   £35,000-49,999        12                   40                      19
##   £50,000-74,999         2                   27                      12
##   £100,000-149,000       2                   11                       3
##   £150,000 or more       1                    2                       1
##   Prefer not to say      1                   12                      13
##
##                     Rarely or never
##   Less than £12,000               6
##   £12,000-14,999                  2
##   £15,000-19,999                  6
##   £20,000-24,999                  2
##   £25,000-34,999                  3
##   £35,000-49,999                  5
##   £50,000-74,999                  4
##   £100,000-149,000                0
##   £150,000 or more                0
##   Prefer not to say               2
```

```r
chi.inc.choice.sim$expected
```

```
##
##                     Always As often as they can If cheaper or preferred
##   Less than £12,000  4.830                21.84                   12.18
##   £12,000-14,999     3.220                14.56                    8.12
##   £15,000-19,999     7.245                32.76                   18.27
##   £20,000-24,999     5.290                23.92                   13.34
##   £25,000-34,999     5.980                27.04                   15.08
##   £35,000-49,999     8.740                39.52                   22.04
##   £50,000-74,999     5.175                23.40                   13.05
##   £100,000-149,000   1.840                 8.32                    4.64
##   £150,000 or more   0.460                 2.08                    1.16
##   Prefer not to say  3.220                14.56                    8.12
##
##                     Rarely or never
##   Less than £12,000           3.150
##   £12,000-14,999              2.100
##   £15,000-19,999              4.725
##   £20,000-24,999              3.450
##   £25,000-34,999              3.900
##   £35,000-49,999              5.700
##   £50,000-74,999              3.375
##   £100,000-149,000            1.200
##   £150,000 or more            0.300
##   Prefer not to say           2.100
```

```r
chi.inc.barrier.sim$observed
```

```
##
##                      Council collection Unclear information Difficult transport
##   Less than £12,000                   9                   7                   6
##   £12,000-14,999                      5                  10                   1
##   £15,000-19,999                     17                   9                   6
##   £20,000-24,999                     13                   4                   3
##   £25,000-34,999                     12                   3                   4
##   £35,000-49,999                     28                  13                   7
##   £50,000-74,999                     15                   7                   3
##   £100,000-149,000                    6                   3                   0
##   £150,000 or more                    1                   0                   0
##   Prefer not to say                   9                   6                   1
##
##                      No local facilities No support Ends up in landfills
##   Less than £12,000                    3          0                    3
##   £12,000-14,999                       3          0                    2
##   £15,000-19,999                       4          5                    4
##   £20,000-24,999                       6          0                    2
##   £25,000-34,999                       8          5                    6
##   £35,000-49,999                       7          0                    6
##   £50,000-74,999                       1          3                    6
##   £100,000-149,000                     1          1                    3
##   £150,000 or more                     0          2                    0
##   Prefer not to say                    2          2                    1
##
##                      Forgetting Recycling a hassle Household disagrees
##   Less than £12,000           2                  2                   1
##   £12,000-14,999              2                  1                   0
##   £15,000-19,999              3                  2                   2
##   £20,000-24,999              4                  1                   1
##   £25,000-34,999              2                  0                   0
##   £35,000-49,999              6                  2                   0
##   £50,000-74,999              5                  1                   1
##   £100,000-149,000            0                  0                   0
##   £150,000 or more            0                  0                   0
##   Prefer not to say           1                  1                   0
##
##                      Recycling not important Other Already doing everything
##   Less than £12,000                        0     0                        9
##   £12,000-14,999                           0     2                        2
##   £15,000-19,999                           1     1                        9
##   £20,000-24,999                           2     1                        9
##   £25,000-34,999                           0     0                       12
##   £35,000-49,999                           0     0                        7
##   £50,000-74,999                           0     0                        3
##   £100,000-149,000                         0     0                        2
##   £150,000 or more                         1     0                        0
##   Prefer not to say                        1     0                        4
```

```r
chi.inc.barrier.sim$expected
```

```
##
##                      Council collection Unclear information Difficult transport
##   Less than £12,000             12.0750               6.510              3.2550
##   £12,000-14,999                 8.0500               4.340              2.1700
##   £15,000-19,999                18.1125               9.765              4.8825
##   £20,000-24,999                13.2250               7.130              3.5650
##   £25,000-34,999                14.9500               8.060              4.0300
##   £35,000-49,999                21.8500              11.780              5.8900
##   £50,000-74,999                12.9375               6.975              3.4875
##   £100,000-149,000               4.6000               2.480              1.2400
##   £150,000 or more               1.1500               0.620              0.3100
##   Prefer not to say              8.0500               4.340              2.1700
##
##                      No local facilities No support Ends up in landfills
##   Less than £12,000               3.6750      1.890               3.4650
##   £12,000-14,999                  2.4500      1.260               2.3100
##   £15,000-19,999                  5.5125      2.835               5.1975
##   £20,000-24,999                  4.0250      2.070               3.7950
##   £25,000-34,999                  4.5500      2.340               4.2900
##   £35,000-49,999                  6.6500      3.420               6.2700
##   £50,000-74,999                  3.9375      2.025               3.7125
##   £100,000-149,000                1.4000      0.720               1.3200
##   £150,000 or more                0.3500      0.180               0.3300
##   Prefer not to say               2.4500      1.260               2.3100
##
##                      Forgetting Recycling a hassle Household disagrees
##   Less than £12,000      2.6250              1.050              0.5250
##   £12,000-14,999         1.7500              0.700              0.3500
##   £15,000-19,999         3.9375              1.575              0.7875
##   £20,000-24,999         2.8750              1.150              0.5750
##   £25,000-34,999         3.2500              1.300              0.6500
##   £35,000-49,999         4.7500              1.900              0.9500
##   £50,000-74,999         2.8125              1.125              0.5625
##   £100,000-149,000       1.0000              0.400              0.2000
##   £150,000 or more       0.2500              0.100              0.0500
##   Prefer not to say      1.7500              0.700              0.3500
##
##                      Recycling not important Other Already doing everything
##   Less than £12,000                   0.5250  0.42                   5.9850
##   £12,000-14,999                      0.3500  0.28                   3.9900
##   £15,000-19,999                      0.7875  0.63                   8.9775
##   £20,000-24,999                      0.5750  0.46                   6.5550
##   £25,000-34,999                      0.6500  0.52                   7.4100
##   £35,000-49,999                      0.9500  0.76                  10.8300
##   £50,000-74,999                      0.5625  0.45                   6.4125
##   £100,000-149,000                    0.2000  0.16                   2.2800
##   £150,000 or more                    0.0500  0.04                   0.5700
##   Prefer not to say                   0.3500  0.28                   3.9900
```

```
chi.inc.barrier2.sim$observed
```

```
##
##                      Limited alternatives No SUP-free alternatives
##   Less than £12,000                     7                        8
```

```
##   £12,000-14,999                     6                          4
##   £15,000-19,999                    10                         16
##   £20,000-24,999                     8                          6
##   £25,000-34,999                    13                          8
##   £35,000-49,999                    21                         19
##   £50,000-74,999                    11                         11
##   £100,000-149,000                   2                          4
##   £150,000 or more                   0                          2
##   Prefer not to say                 12                          7
##
##                       Alternatives expensive Limited functioning
##   Less than £12,000                       9                   4
##   £12,000-14,999                          9                   5
##   £15,000-19,999                         14                   6
##   £20,000-24,999                         11                   6
##   £25,000-34,999                          7                   3
##   £35,000-49,999                         17                   7
##   £50,000-74,999                          6                   5
##   £100,000-149,000                        6                   2
##   £150,000 or more                        1                   0
##   Prefer not to say                       5                   1
##
##                       Forgetting reusables Reducing not important Other
##   Less than £12,000                     9                      0     0
##   £12,000-14,999                        2                      1     1
##   £15,000-19,999                        4                      3     3
##   £20,000-24,999                        4                      3     0
##   £25,000-34,999                        9                      1     1
##   £35,000-49,999                        6                      2     1
##   £50,000-74,999                        5                      2     1
##   £100,000-149,000                      1                      0     0
##   £150,000 or more                      0                      1     0
##   Prefer not to say                     0                      0     1
##
##                       No barriers
##   Less than £12,000             5
##   £12,000-14,999                0
##   £15,000-19,999                7
##   £20,000-24,999                8
##   £25,000-34,999               10
##   £35,000-49,999                3
##   £50,000-74,999                4
##   £100,000-149,000              1
##   £150,000 or more              0
##   Prefer not to say             2
```

```
chi.inc.barrier2.sim$expected
```

```
##
##                       Limited alternatives No SUP-free alternatives
##   Less than £12,000                  9.450                   8.9250
##   £12,000-14,999                     6.300                   5.9500
##   £15,000-19,999                    14.175                  13.3875
##   £20,000-24,999                    10.350                   9.7750
```

```
##   £25,000-34,999                 11.700                  11.0500
##   £35,000-49,999                 17.100                  16.1500
##   £50,000-74,999                 10.125                   9.5625
##   £100,000-149,000                3.600                   3.4000
##   £150,000 or more                0.900                   0.8500
##   Prefer not to say               6.300                   5.9500
##
##                      Alternatives expensive Limited functioning
##   Less than £12,000                  8.9250              4.0950
##   £12,000-14,999                     5.9500              2.7300
##   £15,000-19,999                    13.3875              6.1425
##   £20,000-24,999                     9.7750              4.4850
##   £25,000-34,999                    11.0500              5.0700
##   £35,000-49,999                    16.1500              7.4100
##   £50,000-74,999                     9.5625              4.3875
##   £100,000-149,000                   3.4000              1.5600
##   £150,000 or more                   0.8500              0.3900
##   Prefer not to say                  5.9500              2.7300
##
##                      Forgetting reusables Reducing not important Other
##   Less than £12,000                   4.2                 1.3650  0.84
##   £12,000-14,999                      2.8                 0.9100  0.56
##   £15,000-19,999                      6.3                 2.0475  1.26
##   £20,000-24,999                      4.6                 1.4950  0.92
##   £25,000-34,999                      5.2                 1.6900  1.04
##   £35,000-49,999                      7.6                 2.4700  1.52
##   £50,000-74,999                      4.5                 1.4625  0.90
##   £100,000-149,000                    1.6                 0.5200  0.32
##   £150,000 or more                    0.4                 0.1300  0.08
##   Prefer not to say                   2.8                 0.9100  0.56
##
##                      No barriers
##   Less than £12,000          4.2
##   £12,000-14,999             2.8
##   £15,000-19,999             6.3
##   £20,000-24,999             4.6
##   £25,000-34,999             5.2
##   £35,000-49,999             7.6
##   £50,000-74,999             4.5
##   £100,000-149,000           1.6
##   £150,000 or more           0.4
##   Prefer not to say          2.8
```

```
chi.inc.mainconsid.sim$observed
```

```
##
##                      Value for money Price Quality Deals/discounts
##   Less than £12,000               12    12      11               4
##   £12,000-14,999                   9     6       3               2
##   £15,000-19,999                  18    11      16               5
##   £20,000-24,999                  10    17      10               3
##   £25,000-34,999                  18    15       9               1
##   £35,000-49,999                  23    19      18               2
##   £50,000-74,999                  16     4      10               8
```

```
##   £100,000-149,000               5     3       7                0
##   £150,000 or more               0     1       1                1
##   Prefer not to say              8     9       3                0
##
##                      Use-by-dates/longevity Convenience
##   Less than £12,000                       1           1
##   £12,000-14,999                          1           2
##   £15,000-19,999                          1           2
##   £20,000-24,999                          2           0
##   £25,000-34,999                          1           0
##   £35,000-49,999                          2           2
##   £50,000-74,999                          2           1
##   £100,000-149,000                        1           0
##   £150,000 or more                        0           0
##   Prefer not to say                       3           2
##
##                      Ease of recycling packaging Sustainability Brand Ethics
##   Less than £12,000                            1              0     0      0
##   £12,000-14,999                               2              1     1      1
##   £15,000-19,999                               1              4     2      1
##   £20,000-24,999                               2              2     0      0
##   £25,000-34,999                               6              2     0      0
##   £35,000-49,999                               6              2     1      1
##   £50,000-74,999                               3              1     0      0
##   £100,000-149,000                             0              0     0      0
##   £150,000 or more                             0              0     0      1
##   Prefer not to say                            1              1     0      1
##
##                      Other
##   Less than £12,000      0
##   £12,000-14,999         0
##   £15,000-19,999         2
##   £20,000-24,999         0
##   £25,000-34,999         0
##   £35,000-49,999         0
##   £50,000-74,999         0
##   £100,000-149,000       0
##   £150,000 or more       0
##   Prefer not to say      0
```

```
chi.inc.mainconsid.sim$expected
```

```
##
##                      Value for money   Price Quality Deals/discounts
##   Less than £12,000          12.4950 10.1850    9.24           2.730
##   £12,000-14,999              8.3300  6.7900    6.16           1.820
##   £15,000-19,999             18.7425 15.2775   13.86           4.095
##   £20,000-24,999             13.6850 11.1550   10.12           2.990
##   £25,000-34,999             15.4700 12.6100   11.44           3.380
##   £35,000-49,999             22.6100 18.4300   16.72           4.940
##   £50,000-74,999             13.3875 10.9125    9.90           2.925
##   £100,000-149,000            4.7600  3.8800    3.52           1.040
##   £150,000 or more            1.1900  0.9700    0.88           0.260
##   Prefer not to say           8.3300  6.7900    6.16           1.820
```

```
##
##                     Use-by-dates/longevity Convenience
##   Less than £12,000                  1.470       1.050
##   £12,000-14,999                     0.980       0.700
##   £15,000-19,999                     2.205       1.575
##   £20,000-24,999                     1.610       1.150
##   £25,000-34,999                     1.820       1.300
##   £35,000-49,999                     2.660       1.900
##   £50,000-74,999                     1.575       1.125
##   £100,000-149,000                   0.560       0.400
##   £150,000 or more                   0.140       0.100
##   Prefer not to say                  0.980       0.700
##
##                     Ease of recycling packaging Sustainability Brand Ethics
##   Less than £12,000                       2.310         1.3650  0.42 0.5250
##   £12,000-14,999                          1.540         0.9100  0.28 0.3500
##   £15,000-19,999                          3.465         2.0475  0.63 0.7875
##   £20,000-24,999                          2.530         1.4950  0.46 0.5750
##   £25,000-34,999                          2.860         1.6900  0.52 0.6500
##   £35,000-49,999                          4.180         2.4700  0.76 0.9500
##   £50,000-74,999                          2.475         1.4625  0.45 0.5625
##   £100,000-149,000                        0.880         0.5200  0.16 0.2000
##   £150,000 or more                        0.220         0.1300  0.04 0.0500
##   Prefer not to say                       1.540         0.9100  0.28 0.3500
##
##                     Other
##   Less than £12,000 0.210
##   £12,000-14,999    0.140
##   £15,000-19,999    0.315
##   £20,000-24,999    0.230
##   £25,000-34,999    0.260
##   £35,000-49,999    0.380
##   £50,000-74,999    0.225
##   £100,000-149,000  0.080
##   £150,000 or more  0.020
##   Prefer not to say 0.140
```

```
chi.inc.litter.sim$observed
```

```
##
##                     Strongly agree Agree Neither agree nor disagree Disagree
##   Less than £12,000             18    14                          4        2
##   £12,000-14,999                11    14                          2        1
##   £15,000-19,999                26    27                          8        2
##   £20,000-24,999                17    18                         11        0
##   £25,000-34,999                26    16                          9        1
##   £35,000-49,999                33    35                          7        1
##   £50,000-74,999                20    19                          5        1
##   £100,000-149,000               6     9                          1        0
##   £150,000 or more               3     1                          0        0
##   Prefer not to say             13     9                          6        0
##
##                     Strongly disagree
##   Less than £12,000                 4
```

```
##   £12,000-14,999                  0
##   £15,000-19,999                  0
##   £20,000-24,999                  0
##   £25,000-34,999                  0
##   £35,000-49,999                  0
##   £50,000-74,999                  0
##   £100,000-149,000                0
##   £150,000 or more                0
##   Prefer not to say               0
```

```
chi.inc.litter.sim$expected
```

```
##
##                     Strongly agree  Agree Neither agree nor disagree Disagree
##   Less than £12,000        18.1650 17.010                     5.5650     0.84
##   £12,000-14,999           12.1100 11.340                     3.7100     0.56
##   £15,000-19,999           27.2475 25.515                     8.3475     1.26
##   £20,000-24,999           19.8950 18.630                     6.0950     0.92
##   £25,000-34,999           22.4900 21.060                     6.8900     1.04
##   £35,000-49,999           32.8700 30.780                    10.0700     1.52
##   £50,000-74,999           19.4625 18.225                     5.9625     0.90
##   £100,000-149,000          6.9200  6.480                     2.1200     0.32
##   £150,000 or more          1.7300  1.620                     0.5300     0.08
##   Prefer not to say        12.1100 11.340                     3.7100     0.56
##
##                     Strongly disagree
##   Less than £12,000              0.42
##   £12,000-14,999                 0.28
##   £15,000-19,999                 0.63
##   £20,000-24,999                 0.46
##   £25,000-34,999                 0.52
##   £35,000-49,999                 0.76
##   £50,000-74,999                 0.45
##   £100,000-149,000               0.16
##   £150,000 or more               0.04
##   Prefer not to say              0.28
```

```
chi.inc.attitude.sim$observed
```

```
##
##                     1 (Not concerned)  2  3  4  5  6 7 (Very concerned
##   Less than £12,000                 4  1  8  8  8  2                11
##   £12,000-14,999                    2  2  2  6  5  3                 8
##   £15,000-19,999                    6  1  7 13 15  9                12
##   £20,000-24,999                    4  3  4  7 18  4                 6
##   £25,000-34,999                    2  2  8 10 10 11                 9
##   £35,000-49,999                    3  3  4 19 22 14                11
##   £50,000-74,999                    2  5  4  6 13 10                 5
##   £100,000-149,000                  1  0  0  1 11  0                 3
##   £150,000 or more                  0  0  0  0  2  2                 0
##   Prefer not to say                 3  3  4  3 10  1                 4
```

```r
chi.inc.attitude.sim$expected
```

```
##
##                      1 (Not concerned)    2      3       4      5     6
##   Less than £12,000             2.8350 2.10 4.3050  7.6650 11.970  5.88
##   £12,000-14,999                1.8900 1.40 2.8700  5.1100  7.980  3.92
##   £15,000-19,999                4.2525 3.15 6.4575 11.4975 17.955  8.82
##   £20,000-24,999                3.1050 2.30 4.7150  8.3950 13.110  6.44
##   £25,000-34,999                3.5100 2.60 5.3300  9.4900 14.820  7.28
##   £35,000-49,999                5.1300 3.80 7.7900 13.8700 21.660 10.64
##   £50,000-74,999                3.0375 2.25 4.6125  8.2125 12.825  6.30
##   £100,000-149,000              1.0800 0.80 1.6400  2.9200  4.560  2.24
##   £150,000 or more              0.2700 0.20 0.4100  0.7300  1.140  0.56
##   Prefer not to say             1.8900 1.40 2.8700  5.1100  7.980  3.92
##
##                      7 (Very concerned
##   Less than £12,000             7.2450
##   £12,000-14,999                4.8300
##   £15,000-19,999               10.8675
##   £20,000-24,999                7.9350
##   £25,000-34,999                8.9700
##   £35,000-49,999               13.1100
##   £50,000-74,999                7.7625
##   £100,000-149,000              2.7600
##   £150,000 or more              0.6900
##   Prefer not to say             4.8300
```

```r
chi.inc.ocean.sim$observed
```

```
##
##                      Always Most of the time Sometimes Rarely Never
##   Less than £12,000      4               10        19      5     4
##   £12,000-14,999         3                4        13      5     3
##   £15,000-19,999         4               19        30      7     3
##   £20,000-24,999         2               10        28      6     0
##   £25,000-34,999         1               16        25      9     1
##   £35,000-49,999         3               24        35     12     2
##   £50,000-74,999         1               10        23      9     2
##   £100,000-149,000       0                5         9      1     1
##   £150,000 or more       1                0         3      0     0
##   Prefer not to say      0                6        15      6     1
```

```r
chi.inc.ocean.sim$expected
```

```
##
##                      Always Most of the time Sometimes Rarely  Never
##   Less than £12,000 1.9950            10.92      21.0   6.30 1.7850
##   £12,000-14,999    1.3300             7.28      14.0   4.20 1.1900
##   £15,000-19,999    2.9925            16.38      31.5   9.45 2.6775
##   £20,000-24,999    2.1850            11.96      23.0   6.90 1.9550
##   £25,000-34,999    2.4700            13.52      26.0   7.80 2.2100
##   £35,000-49,999    3.6100            19.76      38.0  11.40 3.2300
```

```
##   £50,000-74,999    2.1375            11.70      22.5   6.75 1.9125
##   £100,000-149,000  0.7600             4.16       8.0   2.40 0.6800
##   £150,000 or more  0.1900             1.04       2.0   0.60 0.1700
##   Prefer not to say 1.3300             7.28      14.0   4.20 1.1900
```

```
chi.inc.actions.sim$observed
```

```
##
##                     Yes, definitely Yes, probably No, probably not
##   Less than £12,000              17            16                 6
##   £12,000-14,999                 10            13                 5
##   £15,000-19,999                 19            37                 5
##   £20,000-24,999                 17            19                 8
##   £25,000-34,999                 16            27                 2
##   £35,000-49,999                 34            35                 4
##   £50,000-74,999                 16            18                 8
##   £100,000-149,000                3            11                 1
##   £150,000 or more                1             3                 0
##   Prefer not to say               9            12                 4
##
##                     No, definitely not I don't know
##   Less than £12,000                  2            1
##   £12,000-14,999                     0            0
##   £15,000-19,999                     2            0
##   £20,000-24,999                     1            1
##   £25,000-34,999                     3            4
##   £35,000-49,999                     2            1
##   £50,000-74,999                     1            2
##   £100,000-149,000                   0            1
##   £150,000 or more                   0            0
##   Prefer not to say                  0            3
```

```
chi.inc.actions.sim$expected
```

```
##
##                     Yes, definitely Yes, probably No, probably not
##   Less than £12,000          14.910       20.0550            4.5150
##   £12,000-14,999              9.940       13.3700            3.0100
##   £15,000-19,999             22.365       30.0825            6.7725
##   £20,000-24,999             16.330       21.9650            4.9450
##   £25,000-34,999             18.460       24.8300            5.5900
##   £35,000-49,999             26.980       36.2900            8.1700
##   £50,000-74,999             15.975       21.4875            4.8375
##   £100,000-149,000            5.680        7.6400            1.7200
##   £150,000 or more            1.420        1.9100            0.4300
##   Prefer not to say           9.940       13.3700            3.0100
##
##                     No, definitely not I don't know
##   Less than £12,000             1.1550       1.3650
##   £12,000-14,999                0.7700       0.9100
##   £15,000-19,999                1.7325       2.0475
##   £20,000-24,999                1.2650       1.4950
##   £25,000-34,999                1.4300       1.6900
```

```
##   £35,000-49,999              2.0900       2.4700
##   £50,000-74,999              1.2375       1.4625
##   £100,000-149,000            0.4400       0.5200
##   £150,000 or more            0.1100       0.1300
##   Prefer not to say           0.7700       0.9100
```

```
chi.inc.bottpurchase.sim$observed
```

```
##
##                       None 1-2 3-5 6-10 11-15 16+ I don't know
##   Less than £12,000    2  15  13   10     1   0            1
##   £12,000-14,999       1  15   6    3     1   1            1
##   £15,000-19,999       1  27  15    9     4   5            2
##   £20,000-24,999       2  14  15   12     1   1            1
##   £25,000-34,999       1  13  21   12     3   0            2
##   £35,000-49,999       5  21  31   11     5   3            0
##   £50,000-74,999       0  13  14   13     3   1            1
##   £100,000-149,000     0   6   8    2     0   0            0
##   £150,000 or more     0   1   1    0     0   2            0
##   Prefer not to say    0  12  10    2     1   1            2
```

```
chi.inc.bottpurchase.sim$expected
```

```
##
##                       None     1-2    3-5   6-10  11-15   16+ I don't know
##   Less than £12,000 1.26 14.3850 14.070  7.770 1.9950 1.470        1.050
##   £12,000-14,999    0.84  9.5900  9.380  5.180 1.3300 0.980        0.700
##   £15,000-19,999    1.89 21.5775 21.105 11.655 2.9925 2.205        1.575
##   £20,000-24,999    1.38 15.7550 15.410  8.510 2.1850 1.610        1.150
##   £25,000-34,999    1.56 17.8100 17.420  9.620 2.4700 1.820        1.300
##   £35,000-49,999    2.28 26.0300 25.460 14.060 3.6100 2.660        1.900
##   £50,000-74,999    1.35 15.4125 15.075  8.325 2.1375 1.575        1.125
##   £100,000-149,000  0.48  5.4800  5.360  2.960 0.7600 0.560        0.400
##   £150,000 or more  0.12  1.3700  1.340  0.740 0.1900 0.140        0.100
##   Prefer not to say 0.84  9.5900  9.380  5.180 1.3300 0.980        0.700
```

```
chi.inc.bottreuse.sim$observed
```

```
##
##                       Never Once 2-4 times 5-10 times More often
##   Less than £12,000     8    4        13          5         12
##   £12,000-14,999        4    6         8          4          6
##   £15,000-19,999        6   10        19          7         21
##   £20,000-24,999       11    4        11         10         10
##   £25,000-34,999       11    8        16         10          7
##   £35,000-49,999       14   13        27         12         10
##   £50,000-74,999        7   12        16          7          3
##   £100,000-149,000      2    2         7          1          4
##   £150,000 or more      2    0         2          0          0
##   Prefer not to say     6    7        10          1          4
```

```r
chi.inc.bottreuse.sim$expected
```

```
##
##                       Never   Once 2-4 times 5-10 times More often
##   Less than £12,000  7.4550  6.930   13.5450     5.9850     8.0850
##   £12,000-14,999     4.9700  4.620    9.0300     3.9900     5.3900
##   £15,000-19,999    11.1825 10.395   20.3175     8.9775    12.1275
##   £20,000-24,999     8.1650  7.590   14.8350     6.5550     8.8550
##   £25,000-34,999     9.2300  8.580   16.7700     7.4100    10.0100
##   £35,000-49,999    13.4900 12.540   24.5100    10.8300    14.6300
##   £50,000-74,999     7.9875  7.425   14.5125     6.4125     8.6625
##   £100,000-149,000   2.8400  2.640    5.1600     2.2800     3.0800
##   £150,000 or more   0.7100  0.660    1.2900     0.5700     0.7700
##   Prefer not to say  4.9700  4.620    9.0300     3.9900     5.3900
```

```r
chi.inc.bottdisp.sim$observed
```

```
##
##                     General waste bin Recycling bin Recycling centre
##   Less than £12,000                 7            32                1
##   £12,000-14,999                    3            23                1
##   £15,000-19,999                    3            43                2
##   £20,000-24,999                    4            36                1
##   £25,000-34,999                    3            46                0
##   £35,000-49,999                    3            67                4
##   £50,000-74,999                    2            40                0
##   £100,000-149,000                  1            12                0
##   £150,000 or more                  0             3                1
##   Prefer not to say                 1            26                0
##
##                     Specialist waste collection Landfill Deposit return scheme
##   Less than £12,000                           0        0                     0
##   £12,000-14,999                              0        0                     0
##   £15,000-19,999                              2        3                     2
##   £20,000-24,999                              2        0                     1
##   £25,000-34,999                              0        1                     0
##   £35,000-49,999                              1        0                     0
##   £50,000-74,999                              0        1                     1
##   £100,000-149,000                            2        0                     0
##   £150,000 or more                            0        0                     0
##   Prefer not to say                           0        0                     0
##
##                     Indefinite storage Other I don't know
##   Less than £12,000                  0     1            1
##   £12,000-14,999                     1     0            0
##   £15,000-19,999                     8     0            0
##   £20,000-24,999                     2     0            0
##   £25,000-34,999                     1     1            0
##   £35,000-49,999                     1     0            0
##   £50,000-74,999                     1     0            0
##   £100,000-149,000                   1     0            0
##   £150,000 or more                   0     0            0
##   Prefer not to say                  1     0            0
```

```
chi.inc.bottdisp.sim$expected
```

```
##
##                      General waste bin Recycling bin Recycling centre
##   Less than £12,000             2.8350         34.44            1.050
##   £12,000-14,999                1.8900         22.96            0.700
##   £15,000-19,999                4.2525         51.66            1.575
##   £20,000-24,999                3.1050         37.72            1.150
##   £25,000-34,999                3.5100         42.64            1.300
##   £35,000-49,999                5.1300         62.32            1.900
##   £50,000-74,999                3.0375         36.90            1.125
##   £100,000-149,000              1.0800         13.12            0.400
##   £150,000 or more              0.2700          3.28            0.100
##   Prefer not to say             1.8900         22.96            0.700
##
##                      Specialist waste collection Landfill Deposit return scheme
##   Less than £12,000                       0.7350   0.5250                  0.42
##   £12,000-14,999                          0.4900   0.3500                  0.28
##   £15,000-19,999                          1.1025   0.7875                  0.63
##   £20,000-24,999                          0.8050   0.5750                  0.46
##   £25,000-34,999                          0.9100   0.6500                  0.52
##   £35,000-49,999                          1.3300   0.9500                  0.76
##   £50,000-74,999                          0.7875   0.5625                  0.45
##   £100,000-149,000                        0.2800   0.2000                  0.16
##   £150,000 or more                        0.0700   0.0500                  0.04
##   Prefer not to say                       0.4900   0.3500                  0.28
##
##                      Indefinite storage Other I don't know
##   Less than £12,000                1.68 0.210       0.1050
##   £12,000-14,999                   1.12 0.140       0.0700
##   £15,000-19,999                   2.52 0.315       0.1575
##   £20,000-24,999                   1.84 0.230       0.1150
##   £25,000-34,999                   2.08 0.260       0.1300
##   £35,000-49,999                   3.04 0.380       0.1900
##   £50,000-74,999                   1.80 0.225       0.1125
##   £100,000-149,000                 0.64 0.080       0.0400
##   £150,000 or more                 0.16 0.020       0.0100
##   Prefer not to say                1.12 0.140       0.0700
```

```
chi.inc.tubspurchase.sim$observed
```

```
##
##                      None 1-2 3-5 6-10 11-15 16+ I don't know
##   Less than £12,000     3  11  18    7     2   0            1
##   £12,000-14,999        1  11  10    4     2   0            0
##   £15,000-19,999        3  17  16   17     2   6            2
##   £20,000-24,999        0  15  15    8     4   2            2
##   £25,000-34,999        0  16  18   11     5   0            2
##   £35,000-49,999        3  14  30   22     4   2            1
##   £50,000-74,999        2   9  13   11     8   1            1
##   £100,000-149,000      0   4   8    1     2   1            0
##   £150,000 or more      0   1   2    0     0   1            0
##   Prefer not to say     0  10  13    4     0   0            1
```

```r
chi.inc.tubspurchase.sim$expected
```

```
##
##                     None  1-2     3-5    6-10  11-15    16+ I don't know
##   Less than £12,000 1.26 11.34 15.0150  8.9250 3.0450 1.3650        1.050
##   £12,000-14,999    0.84  7.56 10.0100  5.9500 2.0300 0.9100        0.700
##   £15,000-19,999    1.89 17.01 22.5225 13.3875 4.5675 2.0475        1.575
##   £20,000-24,999    1.38 12.42 16.4450  9.7750 3.3350 1.4950        1.150
##   £25,000-34,999    1.56 14.04 18.5900 11.0500 3.7700 1.6900        1.300
##   £35,000-49,999    2.28 20.52 27.1700 16.1500 5.5100 2.4700        1.900
##   £50,000-74,999    1.35 12.15 16.0875  9.5625 3.2625 1.4625        1.125
##   £100,000-149,000  0.48  4.32  5.7200  3.4000 1.1600 0.5200        0.400
##   £150,000 or more  0.12  1.08  1.4300  0.8500 0.2900 0.1300        0.100
##   Prefer not to say 0.84  7.56 10.0100  5.9500 2.0300 0.9100        0.700
```

```r
chi.inc.tubreuse.sim$observed
```

```
##
##                     Never Once 2-4 times 5-10 times More often
##   Less than £12,000     5    3        12          6         16
##   £12,000-14,999        3    4         7          3         11
##   £15,000-19,999       10    2        11         10         30
##   £20,000-24,999        7    6        11          5         17
##   £25,000-34,999       10    3         9         13         17
##   £35,000-49,999        8   12        23          5         28
##   £50,000-74,999       10    5        12          9          9
##   £100,000-149,000      1    2         6          3          4
##   £150,000 or more      2    1         0          1          0
##   Prefer not to say     5    3         6          3         11
```

```r
chi.inc.tubreuse.sim$expected
```

```
##
##                       Never   Once 2-4 times 5-10 times More often
##   Less than £12,000  6.4050 4.3050   10.1850      6.090    15.0150
##   £12,000-14,999     4.2700 2.8700    6.7900      4.060    10.0100
##   £15,000-19,999     9.6075 6.4575   15.2775      9.135    22.5225
##   £20,000-24,999     7.0150 4.7150   11.1550      6.670    16.4450
##   £25,000-34,999     7.9300 5.3300   12.6100      7.540    18.5900
##   £35,000-49,999    11.5900 7.7900   18.4300     11.020    27.1700
##   £50,000-74,999     6.8625 4.6125   10.9125      6.525    16.0875
##   £100,000-149,000   2.4400 1.6400    3.8800      2.320     5.7200
##   £150,000 or more   0.6100 0.4100    0.9700      0.580     1.4300
##   Prefer not to say  4.2700 2.8700    6.7900      4.060    10.0100
```

```r
chi.inc.tubdisp.sim$observed
```

```
##
##                     General waste bin Recycling bin Recycling centre
##   Less than £12,000                 7            24                2
##   £12,000-14,999                    6            14                0
```

```
##   £15,000-19,999                    8          33               3
##   £20,000-24,999                   11          25               3
##   £25,000-34,999                   10          28               7
##   £35,000-49,999                   17          41               4
##   £50,000-74,999                   16          22               5
##   £100,000-149,000                  3           8               3
##   £150,000 or more                  1           1               0
##   Prefer not to say                 7          15               2
##
##                     Specialist waste collection Landfill Deposit return scheme
##   Less than £12,000                           2        0                     0
##   £12,000-14,999                              2        0                     0
##   £15,000-19,999                              3        1                     2
##   £20,000-24,999                              0        1                     0
##   £25,000-34,999                              0        0                     0
##   £35,000-49,999                              3        1                     2
##   £50,000-74,999                              0        0                     0
##   £100,000-149,000                            1        0                     0
##   £150,000 or more                            1        0                     0
##   Prefer not to say                           0        0                     0
##
##                     Indefinite storage Other I don't know
##   Less than £12,000                  4     3            0
##   £12,000-14,999                     5     0            1
##   £15,000-19,999                    12     1            0
##   £20,000-24,999                     5     0            1
##   £25,000-34,999                     5     0            2
##   £35,000-49,999                     6     1            1
##   £50,000-74,999                     1     1            0
##   £100,000-149,000                   1     0            0
##   £150,000 or more                   1     0            0
##   Prefer not to say                  2     1            1
```

```
chi.inc.tubdisp.sim$expected
```

```
##
##                     General waste bin Recycling bin Recycling centre
##   Less than £12,000             9.030       22.1550           3.0450
##   £12,000-14,999                6.020       14.7700           2.0300
##   £15,000-19,999               13.545       33.2325           4.5675
##   £20,000-24,999                9.890       24.2650           3.3350
##   £25,000-34,999               11.180       27.4300           3.7700
##   £35,000-49,999               16.340       40.0900           5.5100
##   £50,000-74,999                9.675       23.7375           3.2625
##   £100,000-149,000              3.440        8.4400           1.1600
##   £150,000 or more              0.860        2.1100           0.2900
##   Prefer not to say             6.020       14.7700           2.0300
##
##                     Specialist waste collection Landfill Deposit return scheme
##   Less than £12,000                        1.26   0.3150                  0.42
##   £12,000-14,999                           0.84   0.2100                  0.28
##   £15,000-19,999                           1.89   0.4725                  0.63
##   £20,000-24,999                           1.38   0.3450                  0.46
##   £25,000-34,999                           1.56   0.3900                  0.52
```

```
## £35,000-49,999                          2.28  0.5700                      0.76
## £50,000-74,999                          1.35  0.3375                      0.45
## £100,000-149,000                        0.48  0.1200                      0.16
## £150,000 or more                        0.12  0.0300                      0.04
## Prefer not to say                       0.84  0.2100                      0.28
##
##                     Indefinite storage  Other I don't know
## Less than £12,000                4.410 0.7350        0.630
## £12,000-14,999                   2.940 0.4900        0.420
## £15,000-19,999                   6.615 1.1025        0.945
## £20,000-24,999                   4.830 0.8050        0.690
## £25,000-34,999                   5.460 0.9100        0.780
## £35,000-49,999                   7.980 1.3300        1.140
## £50,000-74,999                   4.725 0.7875        0.675
## £100,000-149,000                 1.680 0.2800        0.240
## £150,000 or more                 0.420 0.0700        0.060
## Prefer not to say                2.940 0.4900        0.420
```

```
chi.inc.filmpurchase.sim$observed
```

```
##
##                     None 1-2 3-5 6-10 11-15 16+ I don't know
## Less than £12,000      2  10  14    9     6   0            1
## £12,000-14,999         3   7  10    5     3   0            0
## £15,000-19,999         4  13  15   16     4   7            4
## £20,000-24,999         0  10  14   13     5   3            1
## £25,000-34,999         0  13  16   16     4   1            2
## £35,000-49,999         1  12  30   24     5   3            1
## £50,000-74,999         1   9  10   14     9   1            1
## £100,000-149,000       0   3   4    7     1   1            0
## £150,000 or more       0   1   1    1     0   1            0
## Prefer not to say      1   6   8    8     2   1            2
```

```
chi.inc.filmpurchase.sim$expected
```

```
##
##                     None   1-2    3-5    6-10  11-15   16+ I don't know
## Less than £12,000 1.26  8.82 12.810 11.8650 4.0950 1.890         1.26
## £12,000-14,999    0.84  5.88  8.540  7.9100 2.7300 1.260         0.84
## £15,000-19,999    1.89 13.23 19.215 17.7975 6.1425 2.835         1.89
## £20,000-24,999    1.38  9.66 14.030 12.9950 4.4850 2.070         1.38
## £25,000-34,999    1.56 10.92 15.860 14.6900 5.0700 2.340         1.56
## £35,000-49,999    2.28 15.96 23.180 21.4700 7.4100 3.420         2.28
## £50,000-74,999    1.35  9.45 13.725 12.7125 4.3875 2.025         1.35
## £100,000-149,000  0.48  3.36  4.880  4.5200 1.5600 0.720         0.48
## £150,000 or more  0.12  0.84  1.220  1.1300 0.3900 0.180         0.12
## Prefer not to say 0.84  5.88  8.540  7.9100 2.7300 1.260         0.84
```

```
chi.inc.filmreuse.sim$observed
```

```
##
##                     Never Once 2-4 times 5-10 times More often
```

```
##   Less than £12,000    23    7         8          1          3
##   £12,000-14,999       17    3         5          2          1
##   £15,000-19,999       34   12        10          2          5
##   £20,000-24,999       27   12         3          2          2
##   £25,000-34,999       40    3         6          2          1
##   £35,000-49,999       41   25         9          1          0
##   £50,000-74,999       27   10         4          2          2
##   £100,000-149,000      7    2         6          0          1
##   £150,000 or more      2    1         1          0          0
##   Prefer not to say    18    8         2          0          0
```

```
chi.inc.filmreuse.sim$expected
```

```
##
##                      Never    Once 2-4 times 5-10 times More often
##   Less than £12,000 24.78  8.7150     5.670       1.26     1.5750
##   £12,000-14,999    16.52  5.8100     3.780       0.84     1.0500
##   £15,000-19,999    37.17 13.0725     8.505       1.89     2.3625
##   £20,000-24,999    27.14  9.5450     6.210       1.38     1.7250
##   £25,000-34,999    30.68 10.7900     7.020       1.56     1.9500
##   £35,000-49,999    44.84 15.7700    10.260       2.28     2.8500
##   £50,000-74,999    26.55  9.3375     6.075       1.35     1.6875
##   £100,000-149,000   9.44  3.3200     2.160       0.48     0.6000
##   £150,000 or more   2.36  0.8300     0.540       0.12     0.1500
##   Prefer not to say 16.52  5.8100     3.780       0.84     1.0500
```

```
chi.inc.filmdisp.sim$observed
```

```
##
##                      General waste bin Recycling bin Recycling centre
##   Less than £12,000                 25            10                2
##   £12,000-14,999                    17             9                1
##   £15,000-19,999                    41            12                3
##   £20,000-24,999                    30            12                2
##   £25,000-34,999                    34            14                2
##   £35,000-49,999                    51            17                3
##   £50,000-74,999                    31            13                1
##   £100,000-149,000                  12             1                2
##   £150,000 or more                   2             0                0
##   Prefer not to say                 22             4                1
##
##                      Specialist waste collection Landfill Deposit return scheme
##   Less than £12,000                            0        0                     0
##   £12,000-14,999                               1        0                     0
##   £15,000-19,999                               3        1                     1
##   £20,000-24,999                               0        0                     0
##   £25,000-34,999                               0        0                     0
##   £35,000-49,999                               1        0                     1
##   £50,000-74,999                               0        0                     0
##   £100,000-149,000                             0        1                     0
##   £150,000 or more                             1        1                     0
##   Prefer not to say                            0        0                     0
##
```

```
##                        Indefinite storage Other I don't know
##   Less than £12,000                     1     1            3
##   £12,000-14,999                        0     0            0
##   £15,000-19,999                        0     2            0
##   £20,000-24,999                        1     1            0
##   £25,000-34,999                        0     1            1
##   £35,000-49,999                        1     0            2
##   £50,000-74,999                        0     0            0
##   £100,000-149,000                      0     0            0
##   £150,000 or more                      0     0            0
##   Prefer not to say                     0     1            0
```

```
chi.inc.filmdisp.sim$expected
```

```
##
##                        General waste bin Recycling bin Recycling centre
##   Less than £12,000              27.8250          9.66           1.7850
##   £12,000-14,999                 18.5500          6.44           1.1900
##   £15,000-19,999                 41.7375         14.49           2.6775
##   £20,000-24,999                 30.4750         10.58           1.9550
##   £25,000-34,999                 34.4500         11.96           2.2100
##   £35,000-49,999                 50.3500         17.48           3.2300
##   £50,000-74,999                 29.8125         10.35           1.9125
##   £100,000-149,000               10.6000          3.68           0.6800
##   £150,000 or more                2.6500          0.92           0.1700
##   Prefer not to say              18.5500          6.44           1.1900
##
##                        Specialist waste collection Landfill Deposit return scheme
##   Less than £12,000                          0.630   0.3150                 0.210
##   £12,000-14,999                             0.420   0.2100                 0.140
##   £15,000-19,999                             0.945   0.4725                 0.315
##   £20,000-24,999                             0.690   0.3450                 0.230
##   £25,000-34,999                             0.780   0.3900                 0.260
##   £35,000-49,999                             1.140   0.5700                 0.380
##   £50,000-74,999                             0.675   0.3375                 0.225
##   £100,000-149,000                           0.240   0.1200                 0.080
##   £150,000 or more                           0.060   0.0300                 0.020
##   Prefer not to say                          0.420   0.2100                 0.140
##
##                        Indefinite storage Other I don't know
##   Less than £12,000                0.3150 0.630        0.630
##   £12,000-14,999                   0.2100 0.420        0.420
##   £15,000-19,999                   0.4725 0.945        0.945
##   £20,000-24,999                   0.3450 0.690        0.690
##   £25,000-34,999                   0.3900 0.780        0.780
##   £35,000-49,999                   0.5700 1.140        1.140
##   £50,000-74,999                   0.3375 0.675        0.675
##   £100,000-149,000                 0.1200 0.240        0.240
##   £150,000 or more                 0.0300 0.060        0.060
##   Prefer not to say                0.2100 0.420        0.420
```

```
chi.inc.bagpurchase.sim$observed
```

```
##
##                      None 1-2 3-5 6-10 11-15 16+ I don't know
##   Less than £12,000   24  12   3    0     2   0            1
##   £12,000-14,999      11   9   5    2     1   0            0
##   £15,000-19,999      33  11   2    6     5   4            2
##   £20,000-24,999      23  10   7    2     1   2            1
##   £25,000-34,999      33  13   5    1     0   0            0
##   £35,000-49,999      51   9   8    3     2   1            2
##   £50,000-74,999      23   9   7    3     2   1            0
##   £100,000-149,000    10   1   4    0     1   0            0
##   £150,000 or more     1   1   1    1     0   0            0
##   Prefer not to say   23   2   1    1     0   0            1
```

```r
chi.inc.bagpurchase.sim$expected
```

```
##
##                        None     1-2    3-5   6-10 11-15  16+ I don't know
##   Less than £12,000 24.36  8.0850 4.5150 1.9950 1.470 0.84       0.7350
##   £12,000-14,999    16.24  5.3900 3.0100 1.3300 0.980 0.56       0.4900
##   £15,000-19,999    36.54 12.1275 6.7725 2.9925 2.205 1.26       1.1025
##   £20,000-24,999    26.68  8.8550 4.9450 2.1850 1.610 0.92       0.8050
##   £25,000-34,999    30.16 10.0100 5.5900 2.4700 1.820 1.04       0.9100
##   £35,000-49,999    44.08 14.6300 8.1700 3.6100 2.660 1.52       1.3300
##   £50,000-74,999    26.10  8.6625 4.8375 2.1375 1.575 0.90       0.7875
##   £100,000-149,000   9.28  3.0800 1.7200 0.7600 0.560 0.32       0.2800
##   £150,000 or more   2.32  0.7700 0.4300 0.1900 0.140 0.08       0.0700
##   Prefer not to say 16.24  5.3900 3.0100 1.3300 0.980 0.56       0.4900
```

```r
chi.inc.bagreuse.sim$observed
```

```
##
##                      Never Once 2-4 times 5-10 times More often
##   Less than £12,000     4    4         6          5         23
##   £12,000-14,999        1    3         7          6         11
##   £15,000-19,999        4    9        14         13         23
##   £20,000-24,999        3    2        13          7         21
##   £25,000-34,999        5    5        12          7         23
##   £35,000-49,999        6   11        17         14         28
##   £50,000-74,999        2    7         9         14         13
##   £100,000-149,000      1    2         6          1          6
##   £150,000 or more      1    0         3          0          0
##   Prefer not to say     4    1         6          3         14
```

```r
chi.inc.bagreuse.sim$expected
```

```
##
##                       Never Once 2-4 times 5-10 times More often
##   Less than £12,000 3.2550 4.62    9.7650      7.350     17.010
##   £12,000-14,999    2.1700 3.08    6.5100      4.900     11.340
##   £15,000-19,999    4.8825 6.93   14.6475     11.025     25.515
##   £20,000-24,999    3.5650 5.06   10.6950      8.050     18.630
##   £25,000-34,999    4.0300 5.72   12.0900      9.100     21.060
```

```
## £35,000-49,999    5.8900 8.36  17.6700    13.300    30.780
## £50,000-74,999    3.4875 4.95  10.4625     7.875    18.225
## £100,000-149,000  1.2400 1.76   3.7200     2.800     6.480
## £150,000 or more  0.3100 0.44   0.9300     0.700     1.620
## Prefer not to say 2.1700 3.08   6.5100     4.900    11.340
```

```
chi.inc.bagdisp.sim$observed
```

```
##
##                   General waste bin Recycling bin Recycling centre
## Less than £12,000                13            11                1
## £12,000-14,999                   10             8                0
## £15,000-19,999                   17            12                5
## £20,000-24,999                   16            15                3
## £25,000-34,999                   20             6                5
## £35,000-49,999                   34            17                6
## £50,000-74,999                   18            10                4
## £100,000-149,000                  3             7                1
## £150,000 or more                  1             0                2
## Prefer not to say                11             6                2
##
##                   Specialist waste collection Landfill Deposit return scheme
## Less than £12,000                           0        0                     2
## £12,000-14,999                              1        0                     1
## £15,000-19,999                              2        1                     5
## £20,000-24,999                              0        0                     0
## £25,000-34,999                              1        0                     1
## £35,000-49,999                              2        0                     0
## £50,000-74,999                              2        0                     2
## £100,000-149,000                            0        1                     0
## £150,000 or more                            0        0                     0
## Prefer not to say                           0        0                     0
##
##                   Indefinite storage Other I don't know
## Less than £12,000                  9     4            2
## £12,000-14,999                     8     0            0
## £15,000-19,999                    13     6            2
## £20,000-24,999                    10     1            1
## £25,000-34,999                    12     5            2
## £35,000-49,999                    12     4            1
## £50,000-74,999                     7     2            0
## £100,000-149,000                   3     1            0
## £150,000 or more                   1     0            0
## Prefer not to say                  8     0            1
```

```
chi.inc.bagdisp.sim$expected
```

```
##
##                   General waste bin Recycling bin Recycling centre
## Less than £12,000           15.0150          9.66           3.0450
## £12,000-14,999              10.0100          6.44           2.0300
## £15,000-19,999              22.5225         14.49           4.5675
## £20,000-24,999              16.4450         10.58           3.3350
```

```
##   £25,000-34,999              18.5900        11.96          3.7700
##   £35,000-49,999              27.1700        17.48          5.5100
##   £50,000-74,999              16.0875        10.35          3.2625
##   £100,000-149,000             5.7200         3.68          1.1600
##   £150,000 or more             1.4300         0.92          0.2900
##   Prefer not to say           10.0100         6.44          2.0300
##
##                       Specialist waste collection Landfill Deposit return scheme
##   Less than £12,000                          0.84    0.210                1.1550
##   £12,000-14,999                             0.56    0.140                0.7700
##   £15,000-19,999                             1.26    0.315                1.7325
##   £20,000-24,999                             0.92    0.230                1.2650
##   £25,000-34,999                             1.04    0.260                1.4300
##   £35,000-49,999                             1.52    0.380                2.0900
##   £50,000-74,999                             0.90    0.225                1.2375
##   £100,000-149,000                           0.32    0.080                0.4400
##   £150,000 or more                           0.08    0.020                0.1100
##   Prefer not to say                          0.56    0.140                0.7700
##
##                       Indefinite storage  Other I don't know
##   Less than £12,000               8.7150 2.4150       0.9450
##   £12,000-14,999                  5.8100 1.6100       0.6300
##   £15,000-19,999                 13.0725 3.6225       1.4175
##   £20,000-24,999                  9.5450 2.6450       1.0350
##   £25,000-34,999                 10.7900 2.9900       1.1700
##   £35,000-49,999                 15.7700 4.3700       1.7100
##   £50,000-74,999                  9.3375 2.5875       1.0125
##   £100,000-149,000                3.3200 0.9200       0.3600
##   £150,000 or more                0.8300 0.2300       0.0900
##   Prefer not to say               5.8100 1.6100       0.6300
```

Vehicle ownership

```
chi.veh.barrier <- chisq.test(veh.barrier)
```

```
## Warning in chisq.test(veh.barrier): Chi-squared approximation may be incorrect
```

```
chi.veh.barrier.sim <- chisq.test(veh.barrier, simulate.p.value = TRUE)
chi.veh.barrier.sim
```

```
##
##  Pearson's Chi-squared test with simulated p-value (based on 2000
##  replicates)
##
## data:  veh.barrier
## X-squared = 21.125, df = NA, p-value = 0.02849
```

```
chi.veh.barrier2 <- chisq.test(veh.barrier2)
```

```
## Warning in chisq.test(veh.barrier2): Chi-squared approximation may be incorrect
```

```r
chi.veh.barrier2.sim <- chisq.test(veh.barrier2, simulate.p.value = TRUE)
chi.veh.barrier2.sim
```

```
##
##  Pearson's Chi-squared test with simulated p-value (based on 2000
##  replicates)
##
## data:  veh.barrier2
## X-squared = 18.263, df = NA, p-value = 0.008496
```

```r
chi.veh.mainconsid <- chisq.test(veh.mainconsid)
```

```
## Warning in chisq.test(veh.mainconsid): Chi-squared approximation may be
## incorrect
```

```r
chi.veh.mainconsid.sim <- chisq.test(veh.mainconsid, simulate.p.value =
TRUE)
chi.veh.mainconsid.sim
```

```
##
##  Pearson's Chi-squared test with simulated p-value (based on 2000
##  replicates)
##
## data:  veh.mainconsid
## X-squared = 11.927, df = NA, p-value = 0.3003
```

```r
chi.veh.litter <- chisq.test(veh.litter)
```

```
## Warning in chisq.test(veh.litter): Chi-squared approximation may be incorrect
```

```r
chi.veh.litter.sim <- chisq.test(veh.litter, simulate.p.value = TRUE)
chi.veh.litter.sim
```

```
##
##  Pearson's Chi-squared test with simulated p-value (based on 2000
##  replicates)
##
## data:  veh.litter
## X-squared = 9.7207, df = NA, p-value = 0.03798
```

```r
chi.veh.actions <- chisq.test(veh.actions)
```

```
## Warning in chisq.test(veh.actions): Chi-squared approximation may be incorrect
```

```r
chi.veh.actions.sim <- chisq.test(veh.actions, simulate.p.value = TRUE)
chi.veh.actions.sim
```

```
##
##  Pearson's Chi-squared test with simulated p-value (based on 2000
##  replicates)
##
## data:  veh.actions
## X-squared = 3.3732, df = NA, p-value = 0.5047
```

```
chi.veh.zerow <- chisq.test(veh.zerow)
```

```
## Warning in chisq.test(veh.zerow): Chi-squared approximation may be incorrect
```

```
chi.veh.zerow.sim <- chisq.test(veh.zerow, simulate.p.value = TRUE)
chi.veh.zerow.sim
```

```
##
##  Pearson's Chi-squared test with simulated p-value (based on 2000
##  replicates)
##
## data:  veh.zerow
## X-squared = 4.1455, df = NA, p-value = 0.5347
```

```
chi.veh.bottpurchase <- chisq.test(veh.bottpurchase)
```

```
## Warning in chisq.test(veh.bottpurchase): Chi-squared approximation may be
## incorrect
```

```
chi.veh.bottpurchase.sim <- chisq.test(veh.bottpurchase, simulate.p.value =
TRUE)
chi.veh.bottpurchase.sim
```

```
##
##  Pearson's Chi-squared test with simulated p-value (based on 2000
##  replicates)
##
## data:  veh.bottpurchase
## X-squared = 14.588, df = NA, p-value = 0.02599
```

```
chi.veh.bottdisp <- chisq.test(veh.bottdisp)
```

```
## Warning in chisq.test(veh.bottdisp): Chi-squared approximation may be incorrect
```

```
chi.veh.bottdisp.sim <- chisq.test(veh.bottdisp, simulate.p.value = TRUE)
chi.veh.bottdisp.sim
```

```
##
##  Pearson's Chi-squared test with simulated p-value (based on 2000
##  replicates)
##
## data:  veh.bottdisp
## X-squared = 18.385, df = NA, p-value = 0.009995
```

```r
chi.veh.tubspurchase <- chisq.test(veh.tubspurchase)
```

```
## Warning in chisq.test(veh.tubspurchase): Chi-squared approximation may be
## incorrect
```

```r
chi.veh.tubspurchase.sim <- chisq.test(veh.tubspurchase, simulate.p.value =
TRUE)
chi.veh.tubspurchase.sim
```

```
##
##  Pearson's Chi-squared test with simulated p-value (based on 2000
##  replicates)
##
## data:  veh.tubspurchase
## X-squared = 4.6975, df = NA, p-value = 0.5912
```

```r
chi.veh.tubdisp <- chisq.test(veh.tubdisp)
```

```
## Warning in chisq.test(veh.tubdisp): Chi-squared approximation may be incorrect
```

```r
chi.veh.tubdisp.sim <- chisq.test(veh.tubdisp, simulate.p.value = TRUE)
chi.veh.tubdisp.sim
```

```
##
##  Pearson's Chi-squared test with simulated p-value (based on 2000
##  replicates)
##
## data:  veh.tubdisp
## X-squared = 11.304, df = NA, p-value = 0.1779
```

```r
chi.veh.filmpurchase <- chisq.test(veh.filmpurchase)
```

```
## Warning in chisq.test(veh.filmpurchase): Chi-squared approximation may be
## incorrect
```

```r
chi.veh.filmpurchase.sim <- chisq.test(veh.filmpurchase, simulate.p.value =
TRUE)
chi.veh.filmpurchase.sim
```

```
##
##  Pearson's Chi-squared test with simulated p-value (based on 2000
##  replicates)
##
## data:  veh.filmpurchase
## X-squared = 8.2761, df = NA, p-value = 0.2214
```

```r
chi.veh.filmreuse <- chisq.test(veh.filmreuse)
```

```
## Warning in chisq.test(veh.filmreuse): Chi-squared approximation may be incorrect
```

```r
chi.veh.filmreuse.sim <- chisq.test(veh.filmreuse, simulate.p.value = TRUE)
chi.veh.filmreuse.sim
```

```
##
##  Pearson's Chi-squared test with simulated p-value (based on 2000
##  replicates)
##
## data:  veh.filmreuse
## X-squared = 0.6904, df = NA, p-value = 0.9575
```

```r
chi.veh.filmdisp <- chisq.test(veh.filmdisp)
```

```
## Warning in chisq.test(veh.filmdisp): Chi-squared approximation may be incorrect
```

```r
chi.veh.filmdisp.sim <- chisq.test(veh.filmdisp, simulate.p.value = TRUE)
chi.veh.filmdisp.sim
```

```
##
##  Pearson's Chi-squared test with simulated p-value (based on 2000
##  replicates)
##
## data:  veh.filmdisp
## X-squared = 5.3941, df = NA, p-value = 0.7371
```

```r
chi.veh.bagpurchase <- chisq.test(veh.bagpurchase)
```

```
## Warning in chisq.test(veh.bagpurchase): Chi-squared approximation may be
## incorrect
```

```r
chi.veh.bagpurchase.sim <- chisq.test(veh.bagpurchase, simulate.p.value =
TRUE)
chi.veh.bagpurchase.sim
```

```
##
##  Pearson's Chi-squared test with simulated p-value (based on 2000
##  replicates)
##
## data:  veh.bagpurchase
## X-squared = 5.9248, df = NA, p-value = 0.4358
```

```r
chi.veh.bagdisp <- chisq.test(veh.bagdisp)
```

```
## Warning in chisq.test(veh.bagdisp): Chi-squared approximation may be incorrect
```

```r
chi.veh.bagdisp.sim <- chisq.test(veh.bagdisp, simulate.p.value = TRUE)
chi.veh.bagdisp.sim
```

```
##
##  Pearson's Chi-squared test with simulated p-value (based on 2000
##  replicates)
##
## data:  veh.bagdisp
## X-squared = 12.466, df = NA, p-value = 0.1219
```

Simulated residuals

```
chi.veh.barrier.sim$residuals
```

```
##
##                      Council collection Unclear information Difficult transport
##   No vehicle                 -0.84556365         -0.06780959          2.92817286
##   1 or more vehicles          0.54696905          0.04386394         -1.89414473
##
##                      No local facilities  No support Ends up in landfills
##   No vehicle                 -0.10114358 -1.00245461          -0.23556966
##   1 or more vehicles          0.06542666  0.64845698           0.15238275
##
##                       Forgetting Recycling a hassle Household disagrees
##   No vehicle         -0.13808619         0.02911113          0.43227816
##   1 or more vehicles  0.08932370        -0.01883109         -0.27962741
##
##                      Recycling not important       Other
##   No vehicle                     -1.21449578  1.67544580
##   1 or more vehicles              0.78561987 -1.08379423
##
##                      Already doing everything
##   No vehicle                      -0.19875093
##   1 or more vehicles               0.12856585
```

```
chi.veh.barrier2.sim$residuals
```

```
##
##                      Limited alternatives No SUP-free alternatives
##   No vehicle                    0.6695559                0.1847231
##   1 or more vehicles           -0.4331151               -0.1194917
##
##                      Alternatives expensive Limited functioning
##   No vehicle                      0.3844238          -0.7385237
##   1 or more vehicles             -0.2486719           0.4777282
##
##                      Forgetting reusables Reducing not important      Other
##   No vehicle                    2.0960010             -1.4476727 -0.2343400
##   1 or more vehicles           -1.3558384              0.9364548  0.1515873
##
##                      No barriers
##   No vehicle          -2.2706678
##   1 or more vehicles   1.4688250
```

```r
chi.veh.mainconsid.sim$residuals
```

```
##
##                       Value for money       Price     Quality Deals/discounts
##   No vehicle               0.82616769  1.00667395 -0.58095082     -1.32516020
##   1 or more vehicles     -0.53442240 -0.65118634  0.37579917      0.85720527
##
##                       Use-by-dates/longevity Convenience
##   No vehicle                     -0.55603665  1.19355614
##   1 or more vehicles              0.35968296 -0.77207467
##
##                       Ease of recycling packaging Sustainability       Brand
##   No vehicle                          -0.97741050     0.08425608 -1.08627805
##   1 or more vehicles                   0.63225671    -0.05450266  0.70267977
##
##                            Ethics       Other
##   No vehicle          -0.39110881 -0.76811457
##   1 or more vehicles   0.25299623  0.49686963
```

```r
chi.veh.litter.sim$residuals
```

```
##
##                       Strongly agree      Agree Neither agree nor disagree
##   No vehicle               0.2750607  0.6089948                 -1.6780001
##   1 or more vehicles     -0.1779283 -0.3939400                  1.0854465
##
##                         Disagree Strongly disagree
##   No vehicle          -0.8852846         1.6754458
##   1 or more vehicles   0.5726633        -1.0837942
```

```r
chi.veh.actions.sim$residuals
```

```
##
##                       Yes, definitely Yes, probably No, probably not
##   No vehicle               -0.4465218     0.8865850       -0.1923292
##   1 or more vehicles        0.2888412    -0.5735045        0.1244119
##
##                       No, definitely not I don't know
##   No vehicle                  -0.6911336   -0.9370298
##   1 or more vehicles           0.4470730    0.6061357
```

```r
chi.veh.zerow.sim$residuals
```

```
##
##                       Yes, shops regularly Yes, shops occasionally
##   No vehicle                    -1.0481045              -0.2857244
##   1 or more vehicles             0.6779865               0.1848263
##
##                       Yes, visited at least once Yes, never visited
##   No vehicle                          -1.1575349          0.4003850
##   1 or more vehicles                   0.7487736         -0.2589967
```

```
##
##                       No, but would like to shop there
##   No vehicle                                 0.3305473
##   1 or more vehicles                        -0.2138208
##
##                       No, not likely to shop there
##   No vehicle                             0.3646115
##   1 or more vehicles                    -0.2358559
```

```
chi.veh.bottpurchase.sim$residuals
```

```
##
##                              None         1-2         3-5        6-10
##   No vehicle          1.30747525  0.72122048  0.07475400 -1.24779053
##   1 or more vehicles -0.84576542 -0.46653529 -0.04835606  0.80715722
##
##                             11-15         16+ I don't know
##   No vehicle          1.43400931 -2.03224014  -0.55311138
##   1 or more vehicles -0.92761640  1.31459348   0.35779070
```

```
chi.veh.bottdisp.sim$residuals
```

```
##
##                       General waste bin Recycling bin Recycling centre
##   No vehicle                  1.4297189     0.3293799       -1.7175564
##   1 or more vehicles         -0.9248411    -0.2130657        1.1110343
##
##                       Specialist waste collection   Landfill
##   No vehicle                           -1.4370108 -1.2144958
##   1 or more vehicles                    0.9295580  0.7856199
##
##                       Deposit return scheme Indefinite storage      Other
##   No vehicle                     -0.1657034         -0.7916942  1.8356636
##   1 or more vehicles              0.1071884          0.5121225 -1.1874342
##
##                       I don't know
##   No vehicle            -0.5431390
##   1 or more vehicles     0.3513399
```

```
chi.veh.tubspurchase.sim$residuals
```

```
##
##                              None         1-2         3-5        6-10
##   No vehicle          1.30747525  0.55629706 -0.02848346 -0.61407957
##   1 or more vehicles -0.84576542 -0.35985141  0.01842507  0.39722914
##
##                             11-15         16+ I don't know
##   No vehicle         -0.18975052 -0.93702976   0.02911113
##   1 or more vehicles  0.12274376  0.60613566  -0.01883109
```

```r
chi.veh.tubdisp.sim$residuals
```

```
##
##                      General waste bin Recycling bin Recycling centre
##   No vehicle               -0.07345840    1.23644567      -0.87353618
##   1 or more vehicles        0.04751798   -0.79981858       0.56506362
##
##                      Specialist waste collection    Landfill
##   No vehicle                         -1.34999477 -0.94074439
##   1 or more vehicles                  0.87326999  0.60853854
##
##                      Deposit return scheme Indefinite storage       Other
##   No vehicle                   -1.08627805        -0.96308373 -0.04523279
##   1 or more vehicles            0.70267977         0.62298917  0.02925969
##
##                      I don't know
##   No vehicle           0.92452461
##   1 or more vehicles  -0.59804647
```

```r
chi.veh.filmpurchase.sim$residuals
```

```
##
##                            None        1-2        3-5       6-10      11-15
##   No vehicle          1.8389693 -0.1566910 -0.4984026 -0.2312230  1.0303953
##   1 or more vehicles -1.1895725  0.1013586  0.3224012  0.1495710 -0.6665309
##
##                             16+ I don't know
##   No vehicle         -1.0024546    0.2444872
##   1 or more vehicles  0.6484570   -0.1581513
```

```r
chi.veh.filmreuse.sim$residuals
```

```
##
##                           Never        Once   2-4 times  5-10 times
##   No vehicle          0.04554247  0.30616996 -0.48355894 -0.28700676
##   1 or more vehicles -0.02946002 -0.19805191  0.31279937  0.18565582
##
##                       More often
##   No vehicle          0.27334505
##   1 or more vehicles -0.17681848
```

```r
chi.veh.filmdisp.sim$residuals
```

```
##
##                      General waste bin Recycling bin Recycling centre
##   No vehicle              -0.472196251   0.932891954     -0.006698164
##   1 or more vehicles       0.305449195  -0.603459039      0.004332836
##
##                      Specialist waste collection     Landfill
##   No vehicle                        -0.578767442 -0.940744386
##   1 or more vehicles                 0.374386812  0.608538535
```

```
##
##                     Deposit return scheme Indefinite storage        Other
##   No vehicle                 -0.768114575        0.122243621  0.924524614
##   1 or more vehicles          0.496869633       -0.079075629 -0.598046465
##
##                     I don't know
##   No vehicle          0.172878586
##   1 or more vehicles -0.111829827
```

```
chi.veh.bagpurchase.sim$residuals
```

```
##
##                            None         1-2         3-5        6-10
##   No vehicle         -0.53669552  0.47943511  0.64998863  0.16684350
##   1 or more vehicles  0.34717178 -0.31013179 -0.42045760 -0.10792591
##
##                           11-15         16+ I don't know
##   No vehicle          0.92016684 -1.53622915  -0.04523279
##   1 or more vehicles -0.59522756  0.99373927   0.02925969
```

```
chi.veh.bagdisp.sim$residuals
```

```
##
##                     General waste bin Recycling bin Recycling centre
##   No vehicle              -1.56812979    1.70070426       0.15214231
##   1 or more vehicles       1.01437481   -1.10013315      -0.09841617
##
##                     Specialist waste collection    Landfill
##   No vehicle                         0.41660452 -0.76811457
##   1 or more vehicles                -0.26948861  0.49686963
##
##                     Deposit return scheme Indefinite storage       Other
##   No vehicle                  -0.69113359         0.10407758 -0.30136633
##   1 or more vehicles           0.44707301        -0.06732458  0.19494459
##
##                     I don't know
##   No vehicle           1.43916499
##   1 or more vehicles  -0.93095145
```

Simulated observed and expected values

```
chi.veh.barrier.sim$observed
```

```
##
##                     Council collection Unclear information Difficult transport
##   No vehicle                        29                  18                  18
##   1 or more vehicles                86                  44                  13
##
##                     No local facilities No support Ends up in landfills
##   No vehicle                         10          3                    9
##   1 or more vehicles                 25         15                   24
##
```

```
##                      Forgetting Recycling a hassle Household disagrees
##   No vehicle                  7                 3                   2
##   1 or more vehicles         18                 7                   3
##
##                      Recycling not important Other Already doing everything
##   No vehicle                               0     3                       16
##   1 or more vehicles                       5     1                       41
```

```
chi.veh.barrier.sim$expected
```

```
##
##                      Council collection Unclear information Difficult transport
##   No vehicle                     33.925               18.29               9.145
##   1 or more vehicles             81.075               43.71              21.855
##
##                      No local facilities No support Ends up in landfills
##   No vehicle                      10.325       5.31                9.735
##   1 or more vehicles              24.675      12.69               23.265
##
##                      Forgetting Recycling a hassle Household disagrees
##   No vehicle              7.375              2.95               1.475
##   1 or more vehicles     17.625              7.05               3.525
##
##                      Recycling not important Other Already doing everything
##   No vehicle                           1.475  1.18                   16.815
##   1 or more vehicles                   3.525  2.82                   40.185
```

```
chi.veh.barrier2.sim$observed
```

```
##
##                      Limited alternatives No SUP-free alternatives
##   No vehicle                           30                       26
##   1 or more vehicles                   60                       59
##
##                      Alternatives expensive Limited functioning
##   No vehicle                             27                   9
##   1 or more vehicles                     58                  30
##
##                      Forgetting reusables Reducing not important Other
##   No vehicle                           19                      1     2
##   1 or more vehicles                   21                     12     6
##
##                      No barriers
##   No vehicle                   4
##   1 or more vehicles          36
```

```
chi.veh.barrier2.sim$expected
```

```
##
##                      Limited alternatives No SUP-free alternatives
##   No vehicle                        26.55                   25.075
##   1 or more vehicles                63.45                   59.925
```

```
##
##                      Alternatives expensive Limited functioning
##   No vehicle                         25.075              11.505
##   1 or more vehicles                 59.925              27.495
##
##                      Forgetting reusables Reducing not important Other
##   No vehicle                         11.8                  3.835  2.36
##   1 or more vehicles                 28.2                  9.165  5.64
##
##                      No barriers
##   No vehicle                11.8
##   1 or more vehicles        28.2
```

```
chi.veh.mainconsid.sim$observed
```

```
##
##                      Value for money Price Quality Deals/discounts
##   No vehicle                      40    34      23               4
##   1 or more vehicles              79    63      65              22
##
##                      Use-by-dates/longevity Convenience
##   No vehicle                              3           5
##   1 or more vehicles                     11           5
##
##                      Ease of recycling packaging Sustainability Brand Ethics
##   No vehicle                                   4              4     0      1
##   1 or more vehicles                          18              9     4      4
##
##                      Other
##   No vehicle             0
##   1 or more vehicles     2
```

```
chi.veh.mainconsid.sim$expected
```

```
##
##                      Value for money  Price Quality Deals/discounts
##   No vehicle                  35.105 28.615   25.96            7.67
##   1 or more vehicles          83.895 68.385   62.04           18.33
##
##                      Use-by-dates/longevity Convenience
##   No vehicle                           4.13        2.95
##   1 or more vehicles                   9.87        7.05
##
##                      Ease of recycling packaging Sustainability Brand Ethics
##   No vehicle                                6.49          3.835  1.18  1.475
##   1 or more vehicles                       15.51          9.165  2.82  3.525
##
##                      Other
##   No vehicle          0.59
##   1 or more vehicles  1.41
```

```r
chi.veh.litter.sim$observed
```

```
##
##                       Strongly agree Agree Neither agree nor disagree Disagree
##   No vehicle                      53    52                          9        1
##   1 or more vehicles             120   110                         44        7
##
##                       Strongly disagree
##   No vehicle                          3
##   1 or more vehicles                  1
```

```r
chi.veh.litter.sim$expected
```

```
##
##                       Strongly agree  Agree Neither agree nor disagree Disagree
##   No vehicle                  51.035  47.79                     15.635     2.36
##   1 or more vehicles         121.965 114.21                     37.365     5.64
##
##                       Strongly disagree
##   No vehicle                       1.18
##   1 or more vehicles               2.82
```

```r
chi.veh.actions.sim$observed
```

```
##
##                       Yes, definitely Yes, probably No, probably not
##   No vehicle                       39            63               12
##   1 or more vehicles              103           128               31
##
##                       No, definitely not I don't know
##   No vehicle                           2            2
##   1 or more vehicles                   9           11
```

```r
chi.veh.actions.sim$expected
```

```
##
##                       Yes, definitely Yes, probably No, probably not
##   No vehicle                    41.89        56.345           12.685
##   1 or more vehicles           100.11       134.655           30.315
##
##                       No, definitely not I don't know
##   No vehicle                       3.245        3.835
##   1 or more vehicles               7.755        9.165
```

```r
chi.veh.zerow.sim$observed
```

```
##
##                       Yes, shops regularly Yes, shops occasionally
##   No vehicle                             2                       8
##   1 or more vehicles                    12                      22
```

```
##
##                      Yes, visited at least once Yes, never visited
##   No vehicle                                  4                 28
##   1 or more vehicles                         20                 60
##
##                      No, but would like to shop there
##   No vehicle                                       54
##   1 or more vehicles                              121
##
##                      No, not likely to shop there
##   No vehicle                                   22
##   1 or more vehicles                           47
```

```
chi.veh.zerow.sim$expected
```

```
##
##                      Yes, shops regularly Yes, shops occasionally
##   No vehicle                         4.13                    8.85
##   1 or more vehicles                 9.87                   21.15
##
##                      Yes, visited at least once Yes, never visited
##   No vehicle                               7.08              25.96
##   1 or more vehicles                      16.92              62.04
##
##                      No, but would like to shop there
##   No vehicle                                   51.625
##   1 or more vehicles                          123.375
##
##                      No, not likely to shop there
##   No vehicle                               20.355
##   1 or more vehicles                       48.645
```

```
chi.veh.bottpurchase.sim$observed
```

```
##
##                      None 1-2 3-5 6-10 11-15 16+ I don't know
##   No vehicle            6  45  40   16     9   0            2
##   1 or more vehicles    6  92  94   58    10  14            8
```

```
chi.veh.bottpurchase.sim$expected
```

```
##
##                      None    1-2   3-5  6-10  11-15  16+ I don't know
##   No vehicle         3.54 40.415 39.53 21.83  5.605 4.13         2.95
##   1 or more vehicles 8.46 96.585 94.47 52.17 13.395 9.87         7.05
```

```
chi.veh.bottdisp.sim$observed
```

```
##
##                      General waste bin Recycling bin Recycling centre
##   No vehicle                        12           100                0
```

```
##   1 or more vehicles                15          228                10
##
##                       Specialist waste collection Landfill Deposit return scheme
##   No vehicle                                    0        0                      1
##   1 or more vehicles                            7        5                      3
##
##                       Indefinite storage Other I don't know
##   No vehicle                           3     2            0
##   1 or more vehicles                  13     0            1
```

```
chi.veh.bottdisp.sim$expected
```

```
##
##                       General waste bin Recycling bin Recycling centre
##   No vehicle                      7.965         96.76             2.95
##   1 or more vehicles             19.035        231.24             7.05
##
##                       Specialist waste collection Landfill Deposit return scheme
##   No vehicle                                2.065    1.475                   1.18
##   1 or more vehicles                        4.935    3.525                   2.82
##
##                       Indefinite storage Other I don't know
##   No vehicle                        4.72  0.59        0.295
##   1 or more vehicles               11.28  1.41        0.705
```

```
chi.veh.tubspurchase.sim$observed
```

```
##
##                       None 1-2 3-5 6-10 11-15 16+ I don't know
##   No vehicle             6  35  42   22     8   2            3
##   1 or more vehicles     6  73 101   63    21  11            7
```

```
chi.veh.tubspurchase.sim$expected
```

```
##
##                       None   1-2     3-5   6-10  11-15   16+ I don't know
##   No vehicle          3.54 31.86  42.185 25.075  8.555 3.835         2.95
##   1 or more vehicles  8.46 76.14 100.815 59.925 20.445 9.165         7.05
```

```
chi.veh.tubdisp.sim$observed
```

```
##
##                       General waste bin Recycling bin Recycling centre
##   No vehicle                         25            72                6
##   1 or more vehicles                 61           139               23
##
##                       Specialist waste collection Landfill Deposit return scheme
##   No vehicle                                    1        0                      0
##   1 or more vehicles                           11        3                      4
##
##                       Indefinite storage Other I don't know
##   No vehicle                           9     2            3
##   1 or more vehicles                  33     5            3
```

```r
chi.veh.tubdisp.sim$expected
```

```
##
##                       General waste bin Recycling bin Recycling centre
##   No vehicle                      25.37        62.245            8.555
##   1 or more vehicles              60.63       148.755           20.445
##
##                       Specialist waste collection Landfill Deposit return scheme
##   No vehicle                                 3.54    0.885                  1.18
##   1 or more vehicles                         8.46    2.115                  2.82
##
##                       Indefinite storage Other I don’t know
##   No vehicle                       12.39 2.065         1.77
##   1 or more vehicles               29.61 4.935         4.23
```

```r
chi.veh.filmpurchase.sim$observed
```

```
##
##                       None 1-2 3-5 6-10 11-15 16+ I don’t know
##   No vehicle             7  24  33   32    15   3            4
##   1 or more vehicles     5  60  89   81    24  15            8
```

```r
chi.veh.filmpurchase.sim$expected
```

```
##
##                       None   1-2   3-5   6-10  11-15   16+ I don’t know
##   No vehicle          3.54 24.78 35.99 33.335 11.505  5.31         3.54
##   1 or more vehicles 8.46 59.22 86.01 79.665 27.495 12.69         8.46
```

```r
chi.veh.filmreuse.sim$observed
```

```
##
##                       Never Once 2-4 times 5-10 times More often
##   No vehicle             70   26        14          3          5
##   1 or more vehicles    166   57        40          9         10
```

```r
chi.veh.filmreuse.sim$expected
```

```
##
##                        Never   Once 2-4 times 5-10 times More often
##   No vehicle           69.62 24.485     15.93       3.54      4.425
##   1 or more vehicles 166.38 58.515     38.07       8.46     10.575
```

```r
chi.veh.filmdisp.sim$observed
```

```
##
##                       General waste bin Recycling bin Recycling centre
##   No vehicle                         74            32                5
##   1 or more vehicles                191            60               12
```

```
##
##                      Specialist waste collection Landfill Deposit return scheme
##   No vehicle                                   1        0                     0
##   1 or more vehicles                           5        3                     2
##
##                      Indefinite storage Other I don't know
##   No vehicle                          1     3            2
##   1 or more vehicles                  2     3            4
```

```
chi.veh.filmdisp.sim$expected
```

```
##
##                      General waste bin Recycling bin Recycling centre
##   No vehicle                    78.175         27.14            5.015
##   1 or more vehicles           186.825         64.86           11.985
##
##                      Specialist waste collection Landfill Deposit return scheme
##   No vehicle                                1.77    0.885                  0.59
##   1 or more vehicles                        4.23    2.115                  1.41
##
##                      Indefinite storage Other I don't know
##   No vehicle                      0.885  1.77         1.77
##   1 or more vehicles              2.115  4.23         4.23
```

```
chi.veh.bagpurchase.sim$observed
```

```
##
##                      None 1-2 3-5 6-10 11-15 16+ I don't know
##   No vehicle           64  25  15    6     6   0            2
##   1 or more vehicles  168  52  28   13     8   8            5
```

```
chi.veh.bagpurchase.sim$expected
```

```
##
##                        None    1-2    3-5   6-10 11-15  16+ I don't know
##   No vehicle          68.44 22.715 12.685  5.605  4.13 2.36        2.065
##   1 or more vehicles 163.56 54.285 30.315 13.395  9.87 5.64        4.935
```

```
chi.veh.bagdisp.sim$observed
```

```
##
##                      General waste bin Recycling bin Recycling centre
##   No vehicle                        32            36                9
##   1 or more vehicles               111            56               20
##
##                      Specialist waste collection Landfill Deposit return scheme
##   No vehicle                                   3        0                     2
##   1 or more vehicles                           5        2                     9
##
##                      Indefinite storage Other I don't know
##   No vehicle                         25     6            5
##   1 or more vehicles                 58    17            4
```

```r
chi.veh.bagdisp.sim$expected
```

```
##
##                       General waste bin Recycling bin Recycling centre
##   No vehicle                     42.185         27.14            8.555
##   1 or more vehicles            100.815         64.86           20.445
##
##                       Specialist waste collection Landfill Deposit return scheme
##   No vehicle                                 2.36     0.59                  3.245
##   1 or more vehicles                         5.64     1.41                  7.755
##
##                       Indefinite storage  Other I don't know
##   No vehicle                      24.485  6.785        2.655
##   1 or more vehicles              58.515 16.215        6.345
```

Living situation

```r
chi.liv.choice <- chisq.test(liv.choice)
```

```
## Warning in chisq.test(liv.choice): Chi-squared approximation may be incorrect
```

```r
chi.liv.choice.sim <- chisq.test(liv.choice, simulate.p.value = TRUE)
chi.liv.choice.sim
```

```
##
##  Pearson's Chi-squared test with simulated p-value (based on 2000
##  replicates)
##
## data:  liv.choice
## X-squared = 12.732, df = NA, p-value = 0.3708
```

```r
chi.liv.barrier <- chisq.test(liv.barrier)
```

```
## Warning in chisq.test(liv.barrier): Chi-squared approximation may be incorrect
```

```r
chi.liv.barrier.sim <- chisq.test(liv.barrier, simulate.p.value = TRUE)
chi.liv.barrier.sim
```

```
##
##  Pearson's Chi-squared test with simulated p-value (based on 2000
##  replicates)
##
## data:  liv.barrier
## X-squared = 40.949, df = NA, p-value = 0.5457
```

```r
chi.liv.barrier2 <- chisq.test(liv.barrier2)
```

```
## Warning in chisq.test(liv.barrier2): Chi-squared approximation may be incorrect
```

```r
chi.liv.barrier2.sim <- chisq.test(liv.barrier2, simulate.p.value = TRUE)
chi.liv.barrier2.sim
```

```
##
##  Pearson's Chi-squared test with simulated p-value (based on 2000
##  replicates)
##
## data:  liv.barrier2
## X-squared = 37.905, df = NA, p-value = 0.1119
```

```r
chi.liv.mainconsid <- chisq.test(liv.mainconsid)
```

```
## Warning in chisq.test(liv.mainconsid): Chi-squared approximation may be
## incorrect
```

```r
chi.liv.mainconsid.sim <- chisq.test(liv.mainconsid, simulate.p.value =
TRUE)
chi.liv.mainconsid.sim
```

```
##
##  Pearson's Chi-squared test with simulated p-value (based on 2000
##  replicates)
##
## data:  liv.mainconsid
## X-squared = 40.941, df = NA, p-value = 0.3808
```

```r
chi.liv.litter <- chisq.test(liv.litter)
```

```
## Warning in chisq.test(liv.litter): Chi-squared approximation may be incorrect
```

```r
chi.liv.litter.sim <- chisq.test(liv.litter, simulate.p.value = TRUE)
chi.liv.litter.sim
```

```
##
##  Pearson's Chi-squared test with simulated p-value (based on 2000
##  replicates)
##
## data:  liv.litter
## X-squared = 25.046, df = NA, p-value = 0.1104
```

```r
chi.liv.attitude <- chisq.test(liv.attitude)
```

```
## Warning in chisq.test(liv.attitude): Chi-squared approximation may be incorrect
```

```r
chi.liv.attitude.sim <- chisq.test(liv.attitude, simulate.p.value = TRUE)
chi.liv.attitude.sim
```

```
##
##  Pearson's Chi-squared test with simulated p-value (based on 2000
##  replicates)
##
## data:  liv.attitude
## X-squared = 33.337, df = NA, p-value = 0.09595
```

```
chi.liv.ocean <- chisq.test(liv.ocean)
```

```
## Warning in chisq.test(liv.ocean): Chi-squared approximation may be incorrect
```

```
chi.liv.ocean.sim <- chisq.test(liv.ocean, simulate.p.value = TRUE)
chi.liv.ocean.sim
```

```
##
##  Pearson's Chi-squared test with simulated p-value (based on 2000
##  replicates)
##
## data:  liv.ocean
## X-squared = 27.124, df = NA, p-value = 0.04498
```

```
chi.liv.actions <- chisq.test(liv.actions)
```

```
## Warning in chisq.test(liv.actions): Chi-squared approximation may be incorrect
```

```
chi.liv.actions.sim <- chisq.test(liv.actions, simulate.p.value = TRUE)
chi.liv.actions.sim
```

```
##
##  Pearson's Chi-squared test with simulated p-value (based on 2000
##  replicates)
##
## data:  liv.actions
## X-squared = 14.949, df = NA, p-value = 0.5032
```

```
chi.liv.attplast <- chisq.test(liv.attplast)
```

```
## Warning in chisq.test(liv.attplast): Chi-squared approximation may be incorrect
```

```
chi.liv.attplast.sim <- chisq.test(liv.attplast, simulate.p.value = TRUE)
chi.liv.attplast.sim
```

```
##
##  Pearson's Chi-squared test with simulated p-value (based on 2000
##  replicates)
##
## data:  liv.attplast
## X-squared = 15.685, df = NA, p-value = 0.2029
```

```r
chi.liv.zerow <- chisq.test(liv.zerow)
```

```
## Warning in chisq.test(liv.zerow): Chi-squared approximation may be incorrect
```

```r
chi.liv.zerow.sim <- chisq.test(liv.zerow, simulate.p.value = TRUE)
chi.liv.zerow.sim
```

```
##
##  Pearson's Chi-squared test with simulated p-value (based on 2000
##  replicates)
##
## data:  liv.zerow
## X-squared = 20.788, df = NA, p-value = 0.4043
```

```r
chi.liv.bottpurchase <- chisq.test(liv.bottpurchase)
```

```
## Warning in chisq.test(liv.bottpurchase): Chi-squared approximation may be
## incorrect
```

```r
chi.liv.bottpurchase.sim <- chisq.test(liv.bottpurchase, simulate.p.value =
TRUE)
chi.liv.bottpurchase.sim
```

```
##
##  Pearson's Chi-squared test with simulated p-value (based on 2000
##  replicates)
##
## data:  liv.bottpurchase
## X-squared = 29.057, df = NA, p-value = 0.2244
```

```r
chi.liv.bottreuse <- chisq.test(liv.bottreuse)
```

```
## Warning in chisq.test(liv.bottreuse): Chi-squared approximation may be incorrect
```

```r
chi.liv.bottreuse.sim <- chisq.test(liv.bottreuse, simulate.p.value = TRUE)
chi.liv.bottreuse.sim
```

```
##
##  Pearson's Chi-squared test with simulated p-value (based on 2000
##  replicates)
##
## data:  liv.bottreuse
## X-squared = 17.753, df = NA, p-value = 0.3318
```

```r
chi.liv.bottdisp <- chisq.test(liv.bottdisp)
```

```
## Warning in chisq.test(liv.bottdisp): Chi-squared approximation may be incorrect
```

```r
chi.liv.bottdisp.sim <- chisq.test(liv.bottdisp, simulate.p.value = TRUE)
chi.liv.bottdisp.sim
```

```
##
##  Pearson's Chi-squared test with simulated p-value (based on 2000
##  replicates)
##
## data:  liv.bottdisp
## X-squared = 40.696, df = NA, p-value = 0.1804
```

```r
chi.liv.tubspurchase <- chisq.test(liv.tubspurchase)
```

```
## Warning in chisq.test(liv.tubspurchase): Chi-squared approximation may be
## incorrect
```

```r
chi.liv.tubspurchase.sim <- chisq.test(liv.tubspurchase, simulate.p.value =
TRUE)
chi.liv.tubspurchase.sim
```

```
##
##  Pearson's Chi-squared test with simulated p-value (based on 2000
##  replicates)
##
## data:  liv.tubspurchase
## X-squared = 34.559, df = NA, p-value = 0.08696
```

```r
chi.liv.tubreuse <- chisq.test(liv.tubreuse)
```

```
## Warning in chisq.test(liv.tubreuse): Chi-squared approximation may be incorrect
```

```r
chi.liv.tubreuse.sim <- chisq.test(liv.tubreuse, simulate.p.value = TRUE)
chi.liv.tubreuse.sim
```

```
##
##  Pearson's Chi-squared test with simulated p-value (based on 2000
##  replicates)
##
## data:  liv.tubreuse
## X-squared = 14.717, df = NA, p-value = 0.5687
```

```r
chi.liv.tubdisp <- chisq.test(liv.tubdisp)
```

```
## Warning in chisq.test(liv.tubdisp): Chi-squared approximation may be incorrect
```

```r
chi.liv.tubdisp.sim <- chisq.test(liv.tubdisp, simulate.p.value = TRUE)
chi.liv.tubdisp.sim
```

```
##
##  Pearson's Chi-squared test with simulated p-value (based on 2000
##  replicates)
##
## data:  liv.tubdisp
## X-squared = 42.502, df = NA, p-value = 0.1559
```

```
chi.liv.filmpurchase <- chisq.test(liv.filmpurchase)
```

```
## Warning in chisq.test(liv.filmpurchase): Chi-squared approximation may be
## incorrect
```

```
chi.liv.filmpurchase.sim <- chisq.test(liv.filmpurchase, simulate.p.value =
TRUE)
chi.liv.filmpurchase.sim
```

```
##
##  Pearson's Chi-squared test with simulated p-value (based on 2000
##  replicates)
##
## data:  liv.filmpurchase
## X-squared = 49.377, df = NA, p-value = 0.007496
```

```
chi.liv.filmreuse <- chisq.test(liv.filmreuse)
```

```
## Warning in chisq.test(liv.filmreuse): Chi-squared approximation may be incorrect
```

```
chi.liv.filmreuse.sim <- chisq.test(liv.filmreuse, simulate.p.value = TRUE)
chi.liv.filmreuse.sim
```

```
##
##  Pearson's Chi-squared test with simulated p-value (based on 2000
##  replicates)
##
## data:  liv.filmreuse
## X-squared = 22.886, df = NA, p-value = 0.1359
```

```
chi.liv.filmdisp <- chisq.test(liv.filmdisp)
```

```
## Warning in chisq.test(liv.filmdisp): Chi-squared approximation may be incorrect
```

```
chi.liv.filmdisp.sim <- chisq.test(liv.filmdisp, simulate.p.value = TRUE)
chi.liv.filmdisp.sim
```

```
##
##  Pearson's Chi-squared test with simulated p-value (based on 2000
##  replicates)
##
## data:  liv.filmdisp
## X-squared = 25.955, df = NA, p-value = 0.6127
```

```r
chi.liv.bagpurchase <- chisq.test(liv.bagpurchase)
```

```
## Warning in chisq.test(liv.bagpurchase): Chi-squared approximation may be
## incorrect
```

```r
chi.liv.bagpurchase.sim <- chisq.test(liv.bagpurchase, simulate.p.value =
TRUE)
chi.liv.bagpurchase.sim
```

```
##
##  Pearson's Chi-squared test with simulated p-value (based on 2000
##  replicates)
##
## data:  liv.bagpurchase
## X-squared = 44.402, df = NA, p-value = 0.02099
```

```r
chi.liv.bagreuse <- chisq.test(liv.bagreuse)
```

```
## Warning in chisq.test(liv.bagreuse): Chi-squared approximation may be incorrect
```

```r
chi.liv.bagreuse.sim <- chisq.test(liv.bagreuse, simulate.p.value = TRUE)
chi.liv.bagreuse.sim
```

```
##
##  Pearson's Chi-squared test with simulated p-value (based on 2000
##  replicates)
##
## data:  liv.bagreuse
## X-squared = 34.492, df = NA, p-value = 0.005997
```

```r
chi.liv.bagdisp <- chisq.test(liv.bagdisp)
```

```
## Warning in chisq.test(liv.bagdisp): Chi-squared approximation may be incorrect
```

```r
chi.liv.bagdisp.sim <- chisq.test(liv.bagdisp, simulate.p.value = TRUE)
chi.liv.bagdisp.sim
```

```
##
##  Pearson's Chi-squared test with simulated p-value (based on 2000
##  replicates)
##
## data:  liv.bagdisp
## X-squared = 42.832, df = NA, p-value = 0.1144
```

Simulated residuals for ‘living situation’

```r
chi.liv.choice.sim$residuals
```

```
##
##                                      Always As often as they can
##   I live alone                   -0.17831388         -0.55518233
##   I live with my parents/family   0.46866428         -0.87771127
##   I live in a house-/ flat-share  1.11900577          0.04385290
##   I live with my partner/children -0.50411756          0.78295308
##   Other                          -0.67823300         -0.05547002
##
##                                  If cheaper or preferred Rarely or never
##   I live alone                               0.25555302      1.18014983
##   I live with my parents/family              1.61475825     -1.44445073
##   I live in a house-/ flat-share            -0.76338629      0.00000000
##   I live with my partner/children           -0.60691739     -0.24394065
##   Other                                     -0.14855627      1.27801930
```

```
chi.liv.barrier.sim$residuals
```

```
##
##                                  Council collection Unclear information
##   I live alone                          -0.08749831         -1.12810581
##   I live with my parents/family         -1.21762858          1.45647478
##   I live in a house-/ flat-share        -0.14744196          0.32128773
##   I live with my partner/children        0.73638039         -0.17664868
##   Other                                 -0.13987572          0.48260048
##
##                                  Difficult transport No local facilities
##   I live alone                            1.07482345         -0.37007637
##   I live with my parents/family           0.52688774         -0.21893874
##   I live in a house-/ flat-share         -0.62475802          0.80178373
##   I live with my partner/children        -0.61716358         -0.14766799
##   Other                                  -0.55677644          1.09870053
##
##                                   No support Ends up in landfills  Forgetting
##   I live alone                    0.42266694          -2.02904145  0.52128604
##   I live with my parents/family   1.12546771          -0.58867430  1.01520306
##   I live in a house-/ flat-share -0.59628479           0.93581920 -0.31622777
##   I live with my partner/children -0.51195667           1.29474146 -0.90787504
##   Other                          -0.42426407          -0.57445626  1.50000000
##
##                                  Recycling a hassle Household disagrees
##   I live alone                           1.12094680         -0.13987572
##   I live with my parents/family          1.52768568          0.45401261
##   I live in a house-/ flat-share         0.00000000          0.70710678
##   I live with my partner/children       -1.44089337         -0.40601406
##   Other                                 -0.31622777         -0.22360680
##
##                                  Recycling not important       Other
##   I live alone                                0.79262909 -0.95916630
##   I live with my parents/family               0.45401261 -0.71414284
##   I live in a house-/ flat-share             -0.70710678  0.94868330
##   I live with my partner/children            -0.40601406  0.59611418
##   Other                                      -0.22360680 -0.20000000
##
##                                  Already doing everything
```

```
##   I live alone                                   1.35054019
##   I live with my parents/family                 -1.58300035
##   I live in a house-/ flat-share                -0.71205164
##   I live with my partner/children                0.29903925
##   Other                                         -0.75498344
```

```
chi.liv.barrier2.sim$residuals
```

```
##
##                                    Limited alternatives No SUP-free alternatives
##   I live alone                               1.60449249              -1.02905365
##   I live with my parents/family             -1.32104220               0.04936155
##   I live in a house-/ flat-share            -1.00000000               0.85749293
##   I live with my partner/children            0.15528425               0.25825905
##   Other                                     -0.94868330               0.16269784
##
##                                    Alternatives expensive Limited functioning
##   I live alone                                -1.02905365          0.01001671
##   I live with my parents/family                1.87194195          0.01233233
##   I live in a house-/ flat-share              -0.51449576          0.55700665
##   I live with my partner/children             -0.03901755         -0.16841713
##   Other                                        0.16269784         -0.62449980
##
##                                    Forgetting reusables Reducing not important
##   I live alone                               0.26375219            -1.15084671
##   I live with my parents/family              0.39852670             1.81950323
##   I live in a house-/ flat-share             0.50000000             1.49099863
##   I live with my partner/children           -0.49835410            -1.11076718
##   Other                                     -0.63245553             2.41294585
##
##                                          Other No barriers
##   I live alone                     -0.61925622  1.25282290
##   I live with my parents/family   -1.00995049 -1.81551052
##   I live in a house-/ flat-share   0.22360680 -1.00000000
##   I live with my partner/children  0.84303275  0.36834868
##   Other                            -0.28284271  0.94868330
```

```
chi.liv.mainconsid.sim$residuals
```

```
##
##                                    Value for money       Price     Quality
##   I live alone                         -0.26186844 -1.33591730  1.50259328
##   I live with my parents/family        -0.04428540  1.60162279 -1.55838254
##   I live in a house-/ flat-share        1.47841614 -0.22475645 -1.61807967
##   I live with my partner/children      -0.67427765  0.18749215  0.60478248
##   Other                                 1.65922428  0.03046038 -0.93808315
##
##                                    Deals/discounts Use-by-dates/longevity
##   I live alone                         -0.40075180            -0.12260121
##   I live with my parents/family        -0.17300904            -1.33603892
##   I live in a house-/ flat-share        0.86824314             0.50709255
##   I live with my partner/children       0.04165673             0.56585435
##   Other                                -0.50990195            -0.37416574
```

```
##
##                                   Convenience Ease of recycling packaging
##   I live alone                     1.12094680                 -0.02667325
##   I live with my parents/family   -0.24354409                 -0.48065055
##   I live in a house-/ flat-share  -1.00000000                  0.53935989
##   I live with my partner/children -0.14083920                  0.08326715
##   Other                           -0.31622777                 -0.46904158
##
##                                   Sustainability       Brand      Ethics
##   I live alone                       -0.57253178 -0.95916630  0.79262909
##   I live with my parents/family       1.04276759  2.08641733  0.45401261
##   I live in a house-/ flat-share     -0.26311741 -0.63245553 -0.70710678
##   I live with my partner/children     0.02945576 -0.08907453 -0.40601406
##   Other                              -0.36055513 -0.20000000 -0.22360680
##
##                                         Other
##   I live alone                     2.27060612
##   I live with my parents/family   -0.50497525
##   I live in a house-/ flat-share  -0.44721360
##   I live with my partner/children -1.03198837
##   Other                           -0.14142136
```

```
chi.liv.litter.sim$residuals
```

```
##
##                                   Strongly agree       Agree
##   I live alone                       -1.39348374  0.94035203
##   I live with my parents/family       0.20067959 -0.58418705
##   I live in a house-/ flat-share     -0.55297409 -0.54659439
##   I live with my partner/children     1.23749138 -0.13619878
##   Other                              -1.31529464  0.29855620
##
##                                   Neither agree nor disagree    Disagree
##   I live alone                                    0.51841400  0.85516335
##   I live with my parents/family                   0.47797301 -0.01980295
##   I live in a house-/ flat-share                  0.73843281  2.45967478
##   I live with my partner/children                -1.17129736 -1.57947516
##   Other                                           2.01920029 -0.28284271
##
##                                   Strongly disagree
##   I live alone                           0.08340577
##   I live with my parents/family          0.68613724
##   I live in a house-/ flat-share         0.94868330
##   I live with my partner/children       -0.77426324
##   Other                                 -0.20000000
```

```
chi.liv.attitude.sim$residuals
```

```
##
##                                   1 (Not concerned)           2           3
##   I live alone                          -0.08427010 -0.27975144 -0.14002727
##   I live with my parents/family          2.45634691 -0.34442336 -0.09950259
##   I live in a house-/ flat-share         1.39973542  0.00000000  1.43220792
```

```
##   I live with my partner/children       -1.94566260  0.41367470 -0.60620314
##   Other                                  1.40488566 -0.44721360  0.92142520
##
##                                                  4           5           6
##   I live alone                      -0.19279765 -0.23825580 -0.24520241
##   I live with my parents/family      1.53811129 -0.40262511 -0.42663428
##   I live in a house-/ flat-share   -0.48115159 -1.59934197 -0.67612340
##   I live with my partner/children -0.46072184  1.06464338  0.76545993
##   Other                              0.31601110 -0.13112201 -0.74833148
##
##                                     7 (Very concerned
##   I live alone                              1.03672027
##   I live with my parents/family            -1.95461452
##   I live in a house-/ flat-share            1.18014983
##   I live with my partner/children          -0.12249324
##   Other                                    -0.83066239
```

```
chi.liv.ocean.sim$residuals
```

```
##
##                                          Always Most of the time   Sometimes
##   I live alone                      -1.13372475      -1.21043401  0.58976782
##   I live with my parents/family      1.65602459       0.47783448  0.09901475
##   I live in a house-/ flat-share     0.07254763       1.11631261  0.00000000
##   I live with my partner/children -0.03694037       0.08331347 -0.33915111
##   Other                             -0.43588989      -0.03922323 -0.70710678
##
##                                          Rarely       Never
##   I live alone                       1.39979295 -0.46020678
##   I live with my parents/family     -0.95810952 -1.47224319
##   I live in a house-/ flat-share    -2.44948974  1.76401947
##   I live with my partner/children  0.53959064 -0.01744918
##   Other                              0.51639778  2.01304569
```

```
chi.liv.actions.sim$residuals
```

```
##
##                                     Yes, definitely Yes, probably
##   I live alone                           1.10938213   -0.89469311
##   I live with my parents/family         -1.19976581    0.53649316
##   I live in a house-/ flat-share        -0.31844694    0.20593294
##   I live with my partner/children       0.15927433    0.02900343
##   Other                                 -1.19163753    1.51227093
##
##                                     No, probably not No, definitely not
##   I live alone                            0.67094097        -0.96190276
##   I live with my parents/family           0.22101464        -0.33987127
##   I live in a house-/ flat-share          0.33756998        -0.09534626
##   I live with my partner/children       -0.60552128         0.88524744
##   Other                                  -0.65574385        -0.33166248
##
##                                     I don't know
##   I live alone                        -0.57253178
```

```
##   I live with my parents/family     1.81950323
##   I live in a house-/ flat-share   -0.26311741
##   I live with my partner/children  -0.35061855
##   Other                            -0.36055513
```

```r
chi.liv.bottpurchase.sim$residuals
```

```
##
##                                          None         1-2         3-5
##   I live alone                     1.34832155  2.04689623 -0.68809285
##   I live with my parents/family   -0.42847960 -0.59039380 -0.50442728
##   I live in a house-/ flat-share  -1.09544512  0.08105148  0.71026587
##   I live with my partner/children -0.15428162 -0.93107269  0.31312192
##   Other                           -0.34641016 -1.17046999  0.57015316
##
##                                          6-10       11-15         16+
##   I live alone                    -1.45920636 -1.61208962 -0.12260121
##   I live with my parents/family   -0.14161795  1.01353202  2.40636702
##   I live in a house-/ flat-share  -0.51465024  0.07254763 -0.33806170
##   I live with my partner/children  1.05060420  0.59183191 -0.89914073
##   Other                            1.46472025 -0.43588989 -0.37416574
##
##                                   I don’t know
##   I live alone                     -0.19781414
##   I live with my parents/family     0.64207079
##   I live in a house-/ flat-share    0.00000000
##   I live with my partner/children  -0.14083920
##   Other                            -0.31622777
```

```r
chi.liv.bottreuse.sim$residuals
```

```
##
##                                       Never       Once  2-4 times 5-10 times
##   I live alone                     0.1657989 -0.5595264 -0.4901765 -0.5827484
##   I live with my parents/family   -0.3498145  0.2016642  0.6293842 -1.2120571
##   I live in a house-/ flat-share  -1.1634093 -0.2335497 -0.5290037  2.2199257
##   I live with my partner/children  0.3565748  0.4815865  0.2784114 -0.0639826
##   Other                            1.5309483 -0.8124038 -0.2553308  0.5695489
##
##                                   More often
##   I live alone                     1.4946564
##   I live with my parents/family    0.3773990
##   I live in a house-/ flat-share   0.1081125
##   I live with my partner/children -1.0935734
##   Other                           -0.8774964
```

```r
chi.liv.bottdisp.sim$residuals
```

```
##
##                                   General waste bin Recycling bin
##   I live alone                           0.31701608   -0.05065844
##   I live with my parents/family          2.99531497   -1.51851635
```

```
##   I live in a house-/ flat-share        0.18257419   -0.13968606
##   I live with my partner/children      -1.94566260    0.85805737
##   Other                                 1.40488566   -0.15460414
##
##                                  Recycling centre Specialist waste collection
##   I live alone                        0.46156633                 -1.26885775
##   I live with my parents/family       0.64207079                  1.17230231
##   I live in a house-/ flat-share      0.00000000                  0.35856858
##   I live with my partner/children    -0.57419059                  0.14114246
##   Other                              -0.31622777                 -0.26457513
##
##                                     Landfill Deposit return scheme
##   I live alone                    -0.13987572           0.08340577
##   I live with my parents/family    0.45401261           0.68613724
##   I live in a house-/ flat-share  -0.70710678          -0.63245553
##   I live with my partner/children  0.20683735          -0.08907453
##   Other                           -0.22360680          -0.20000000
##
##                                  Indefinite storage       Other I don't know
##   I live alone                           0.16681153  0.79618656  -0.47958315
##   I live with my parents/family          1.37227448 -0.50497525  -0.35707142
##   I live in a house-/ flat-share        -0.47434165  1.78885438   2.84604989
##   I live with my partner/children       -0.52074342 -1.03198837  -0.72972598
##   Other                                 -0.40000000 -0.14142136  -0.10000000
```

```
chi.liv.tubspurchase.sim$residuals
```

```
##
##                                          None          1-2          3-5
##   I live alone                     0.746392289  1.637247602  0.367917562
##   I live with my parents/family   -0.428479604 -1.554922841  0.882328180
##   I live in a house-/ flat-share  -1.095445115  1.278019301 -1.401547596
##   I live with my partner/children  0.241312270 -0.858438733  0.097693701
##   Other                           -0.346410162 -0.076980036 -1.195826074
##
##                                          6-10        11-15          16+
##   I live alone                    -1.707550556 -1.808231221  0.005783149
##   I live with my parents/family    0.049361552  0.677366385  0.266031956
##   I live in a house-/ flat-share   0.171498585  1.233162461 -0.263117406
##   I live with my partner/children  1.001450550 -0.112604169  0.029455759
##   Other                            0.162697843  3.175390283 -0.360555128
##
##                                   I don't know
##   I live alone                     0.461566331
##   I live with my parents/family    0.642070792
##   I live in a house-/ flat-share   0.000000000
##   I live with my partner/children -0.574190592
##   Other                           -0.316227766
```

```
chi.liv.tubreuse.sim$residuals
```

```
##
##                                        Never        Once   2-4 times
```

```
##   I live alone                     1.32686749 -0.14002727 -1.12420299
##   I live with my parents/family   -0.63736704 -0.09950259 -0.67320763
##   I live in a house-/ flat-share  -0.85026515 -0.54325128  0.73848549
##   I live with my partner/children -0.43557659  0.46388184  0.74405514
##   Other                            1.77971263 -0.64031242  0.03046038
##
##                                    5-10 times  More often
##   I live alone                    -0.91446808  0.71665459
##   I live with my parents/family    0.22247760  0.88232818
##   I live in a house-/ flat-share   0.49827288 -0.07933288
##   I live with my partner/children  0.20063240 -0.70448332
##   Other                            0.55148702 -1.19582607
```

```
chi.liv.tubdisp.sim$residuals
```

```
##
##                                   General waste bin Recycling bin
##   I live alone                          -0.17538056   -0.36317452
##   I live with my parents/family         -0.29142244   -1.13799322
##   I live in a house-/ flat-share        -0.20459830   -0.02177002
##   I live with my partner/children        0.17806477    0.81533926
##   Other                                  1.22929361   -0.07572712
##
##                                   Recycling centre Specialist waste collection
##   I live alone                         -0.64662658                  0.14446302
##   I live with my parents/family        -0.88278652                  0.37997248
##   I live in a house-/ flat-share       -0.52849820                 -0.18257419
##   I live with my partner/children       1.15975932                 -0.15428162
##   Other                                -0.53851648                 -0.34641016
##
##                                      Landfill Deposit return scheme
##   I live alone                    -0.83066239           -0.95916630
##   I live with my parents/family    2.61534249            2.08641733
##   I live in a house-/ flat-share   1.27801930           -0.63245553
##   I live with my partner/children -1.26392247           -0.08907453
##   Other                           -0.17320508           -0.20000000
##
##                                   Indefinite storage       Other I don't know
##   I live alone                            1.71811684  0.30736306   0.52777905
##   I live with my parents/family           1.14299923  0.11379007   2.55532892
##   I live in a house-/ flat-share          0.39036003  0.35856858   0.51639778
##   I live with my partner/children        -1.76881126 -0.37681152  -1.78745629
##   Other                                  -0.64807407 -0.26457513  -0.24494897
```

```
chi.liv.filmpurchase.sim$residuals
```

```
##
##                                          None         1-2         3-5
##   I live alone                     3.15410935  0.38221323  1.12135368
##   I live with my parents/family    0.37997248 -0.82808431 -0.64782230
##   I live in a house-/ flat-share  -1.09544512 -0.13801311 -1.20245650
##   I live with my partner/children -1.73665716  0.33941139  0.25247867
##   Other                           -0.34641016 -0.91651514 -1.10453610
```

```
##
##                                        6-10       11-15         16+
##   I live alone                   -0.97880776 -1.99332498 -0.56027943
##   I live with my parents/family    0.68300589 -1.33301325  1.78556607
##   I live in a house-/ flat-share   0.50571950  2.58248539 -0.59628479
##   I live with my partner/children -0.02223771  0.70932689 -0.51195667
##   Other                            0.81842716  0.97678174  1.93275854
##
##                                  I don't know
##   I live alone                     -0.45746624
##   I live with my parents/family     1.99687665
##   I live in a house-/ flat-share   -0.18257419
##   I live with my partner/children  -0.54987550
##   Other                            -0.34641016
```

```r
chi.liv.filmreuse.sim$residuals
```

```
##
##                                       Never        Once   2-4 times
##   I live alone                     0.36918920 -0.70722160  0.16457626
##   I live with my parents/family   -0.38100896 -0.48646274  0.80604335
##   I live in a house-/ flat-share  -1.77028198  0.93718368  0.25819889
##   I live with my partner/children  0.65386511  0.27112928 -0.51376548
##   Other                            0.41660452  0.18659924 -0.73484692
##
##                                  5-10 times  More often
##   I live alone                   -0.45746624  0.29611005
##   I live with my parents/family    1.18842456  0.06327138
##   I live in a house-/ flat-share   2.55603860  2.04124145
##   I live with my partner/children -1.34106327 -1.05706691
##   Other                           -0.34641016 -0.38729833
```

```r
chi.liv.filmdisp.sim$residuals
```

```
##
##                                  General waste bin Recycling bin
##   I live alone                         0.006404469   0.182608696
##   I live with my parents/family       -0.307516215  -0.213144364
##   I live in a house-/ flat-share      -1.068414449   1.252822899
##   I live with my partner/children      0.579800878  -0.570058172
##   Other                                0.215003291   0.083405766
##
##                                  Recycling centre Specialist waste collection
##   I live alone                        0.045514956                -0.323477482
##   I live with my parents/family       0.565463646                -0.874642784
##   I live in a house-/ flat-share      0.997054486                -0.774596669
##   I live with my partner/children    -0.682179869                 1.009814901
##   Other                              -0.412310563                -0.244948974
##
##                                      Landfill Deposit return scheme
##   I live alone                   -0.830662386          -0.678232998
##   I live with my parents/family    0.998438323          -0.504975247
##   I live in a house-/ flat-share  -0.547722558          -0.447213595
```

```
##   I live with my partner/children  0.318453078           0.906017960
##   Other                           -0.173205081          -0.141421356
##
##                                   Indefinite storage        Other I don't know
##   I live alone                           0.373196145  0.527779049 -0.323477482
##   I live with my parents/family          2.615342490  0.268681117  0.268681117
##   I live in a house-/ flat-share        -0.547722558  0.516397779  1.807392228
##   I live with my partner/children       -1.263922466 -0.668547815 -0.668547815
##   Other                                 -0.173205081 -0.244948974 -0.244948974
```

```
chi.liv.bagpurchase.sim$residuals
```

```
##
##                                          None         1-2         3-5
##   I live alone                     1.04588865 -0.16871320 -2.19089254
##   I live with my parents/family   -1.39370263 -0.58006140  2.35642174
##   I live in a house-/ flat-share  -2.32527344  1.54961244  3.23102691
##   I live with my partner/children  1.03105259 -0.46889742 -1.02348248
##   Other                           -0.21009029  1.40171509 -0.65574385
##
##                                          6-10       11-15         16+
##   I live alone                    -0.17699500 -0.67987942  0.85516335
##   I live with my parents/family    0.37103946  1.65788583  0.97034459
##   I live in a house-/ flat-share   0.07254763  0.50709255 -0.89442719
##   I live with my partner/children -0.03694037 -0.53289196 -0.61047199
##   Other                           -0.43588989 -0.37416574 -0.28284271
##
##                                   I don't know
##   I live alone                      0.30736306
##   I live with my parents/family     0.11379007
##   I live in a house-/ flat-share    0.35856858
##   I live with my partner/children  -0.37681152
##   Other                            -0.26457513
```

```
chi.liv.bagreuse.sim$residuals
```

```
##
##                                         Never        Once   2-4 times
##   I live alone                    -0.04868538 -0.35206898 -1.38164181
##   I live with my parents/family   -0.98209863  0.58685846  1.49340546
##   I live in a house-/ flat-share  -1.76068169 -0.19069252  0.22953904
##   I live with my partner/children  1.35185282  0.11775754  0.20995499
##   Other                           -0.55677644 -0.66332496 -0.96436508
##
##                                    5-10 times  More often
##   I live alone                    -0.77258942  1.75947401
##   I live with my parents/family   -1.64855000  0.07591131
##   I live in a house-/ flat-share   3.40168026 -1.54040238
##   I live with my partner/children -0.20883407 -0.67453387
##   Other                            0.35856858  1.08423040
```

```r
chi.liv.bagdisp.sim$residuals
```

```
##
##                                  General waste bin Recycling bin
##   I live alone                         -0.32955649    1.05217391
##   I live with my parents/family        -0.99122867   -0.79710153
##   I live in a house-/ flat-share       -0.34377583   -0.39562828
##   I live with my partner/children       0.89987072   -0.14144301
##   Other                                -0.35958406    0.08340577
##
##                                  Recycling centre Specialist waste collection
##   I live alone                        -1.03382813                 -1.35646600
##   I live with my parents/family        1.19741735                  2.95063968
##   I live in a house-/ flat-share       0.05872202                 -0.89442719
##   I live with my partner/children      0.14186853                 -0.12597041
##   Other                               -0.53851648                 -0.28284271
##
##                                     Landfill Deposit return scheme
##   I live alone                     -0.67823300           -1.59059737
##   I live with my parents/family   -0.50497525            0.50452940
##   I live in a house-/ flat-share  -0.44721360           -0.09534626
##   I live with my partner/children  0.90601796            0.88524744
##   Other                           -0.14142136           -0.33166248
##
##                                  Indefinite storage       Other I don't know
##   I live alone                           0.89489853  0.30869565  -0.04865336
##   I live with my parents/family          0.74314292 -1.12849722   0.79582585
##   I live in a house-/ flat-share        -0.79834165  3.09908822   1.15950181
##   I live with my partner/children       -0.78179996 -0.92795184  -0.81880051
##   Other                                  1.28424184 -0.47958315  -0.30000000
```

Simulated observed and expected values for 'living situation'

```r
chi.liv.choice.sim$observed
```

```
##
##                                  Always As often as they can
##   I live alone                       10                   44
##   I live with my parents/family       7                   22
##   I live in a house-/ flat-share      7                   21
##   I live with my partner/children    22                  119
##   Other                               0                    2
##
##                                  If cheaper or preferred Rarely or never
##   I live alone                                        28              10
##   I live with my parents/family                       21               1
##   I live in a house-/ flat-share                       9               3
##   I live with my partner/children                     57              15
##   Other                                                1               1
```

```
chi.liv.choice.sim$expected
```

```
##
##                                  Always As often as they can
##   I live alone                   10.580               47.84
##   I live with my parents/family   5.865               26.52
##   I live in a house-/ flat-share  4.600               20.80
##   I live with my partner/children 24.495             110.76
##   Other                           0.460                2.08
##
##                                  If cheaper or preferred Rarely or never
##   I live alone                                     26.68           6.900
##   I live with my parents/family                    14.79           3.825
##   I live in a house-/ flat-share                   11.60           3.000
##   I live with my partner/children                  61.77          15.975
##   Other                                             1.16           0.300
```

```
chi.liv.barrier.sim$observed
```

```
##
##                                  Council collection Unclear information
##   I live alone                                   26                  10
##   I live with my parents/family                  10                  12
##   I live in a house-/ flat-share                 11                   7
##   I live with my partner/children                67                  32
##   Other                                           1                   1
##
##                                  Difficult transport No local facilities
##   I live alone                                    10                   7
##   I live with my parents/family                    5                   4
##   I live in a house-/ flat-share                   2                   5
##   I live with my partner/children                 14                  18
##   Other                                            0                   1
##
##                                  No support Ends up in landfills Forgetting
##   I live alone                            5                    2          7
##   I live with my parents/family           4                    3          5
##   I live in a house-/ flat-share          1                    5          2
##   I live with my partner/children         8                   23         10
##   Other                                   0                    0          1
##
##                                  Recycling a hassle Household disagrees
##   I live alone                                    4                   1
##   I live with my parents/family                   3                   1
##   I live in a house-/ flat-share                  1                   1
##   I live with my partner/children                 2                   2
##   Other                                           0                   0
##
##                                  Recycling not important Other
##   I live alone                                         2     0
##   I live with my parents/family                        1     0
##   I live in a house-/ flat-share                       0     1
##   I live with my partner/children                      2     3
```

```
##   Other                                              0     0
##
##                                    Already doing everything
##   I live alone                                           18
##   I live with my parents/family                           3
##   I live in a house-/ flat-share                          4
##   I live with my partner/children                        32
##   Other                                                   0
```

```
chi.liv.barrier.sim$expected
```

```
##
##                                    Council collection Unclear information
##   I live alone                                26.4500              14.260
##   I live with my parents/family               14.6625               7.905
##   I live in a house-/ flat-share              11.5000               6.200
##   I live with my partner/children             61.2375              33.015
##   Other                                        1.1500               0.620
##
##                                    Difficult transport No local facilities
##   I live alone                                  7.1300              8.0500
##   I live with my parents/family                 3.9525              4.4625
##   I live in a house-/ flat-share                3.1000              3.5000
##   I live with my partner/children              16.5075             18.6375
##   Other                                         0.3100              0.3500
##
##                                    No support Ends up in landfills Forgetting
##   I live alone                          4.140               7.5900     5.7500
##   I live with my parents/family         2.295               4.2075     3.1875
##   I live in a house-/ flat-share        1.800               3.3000     2.5000
##   I live with my partner/children       9.585              17.5725    13.3125
##   Other                                 0.180               0.3300     0.2500
##
##                                    Recycling a hassle Household disagrees
##   I live alone                                  2.300              1.1500
##   I live with my parents/family                 1.275              0.6375
##   I live in a house-/ flat-share                1.000              0.5000
##   I live with my partner/children               5.325              2.6625
##   Other                                         0.100              0.0500
##
##                                    Recycling not important Other
##   I live alone                                      1.1500  0.92
##   I live with my parents/family                     0.6375  0.51
##   I live in a house-/ flat-share                    0.5000  0.40
##   I live with my partner/children                   2.6625  2.13
##   Other                                             0.0500  0.04
##
##                                    Already doing everything
##   I live alone                                      13.1100
##   I live with my parents/family                      7.2675
##   I live in a house-/ flat-share                     5.7000
##   I live with my partner/children                   30.3525
##   Other                                              0.5700
```

```r
chi.liv.barrier2.sim$observed
```

```
##
##                                  Limited alternatives No SUP-free alternatives
##   I live alone                                     28                       15
##   I live with my parents/family                     7                       11
##   I live in a house-/ flat-share                    6                       11
##   I live with my partner/children                  49                       47
##   Other                                             0                        1
##
##                                  Alternatives expensive Limited functioning
##   I live alone                                       15                   9
##   I live with my parents/family                      17                   5
##   I live in a house-/ flat-share                      7                   5
##   I live with my partner/children                    45                  20
##   Other                                               1                   0
##
##                                  Forgetting reusables Reducing not important
##   I live alone                                     10                      1
##   I live with my parents/family                     6                      4
##   I live in a house-/ flat-share                    5                      3
##   I live with my partner/children                  19                      4
##   Other                                             0                      1
##
##                                  Other No barriers
##   I live alone                       1          13
##   I live with my parents/family      0           1
##   I live in a house-/ flat-share     1           2
##   I live with my partner/children    6          23
##   Other                              0           1
```

```r
chi.liv.barrier2.sim$expected
```

```
##
##                                  Limited alternatives No SUP-free alternatives
##   I live alone                                 20.700                  19.5500
##   I live with my parents/family                11.475                  10.8375
##   I live in a house-/ flat-share                9.000                   8.5000
##   I live with my partner/children              47.925                  45.2625
##   Other                                         0.900                   0.8500
##
##                                  Alternatives expensive Limited functioning
##   I live alone                                  19.5500              8.9700
##   I live with my parents/family                 10.8375              4.9725
##   I live in a house-/ flat-share                 8.5000              3.9000
##   I live with my partner/children               45.2625             20.7675
##   Other                                          0.8500              0.3900
##
##                                  Forgetting reusables Reducing not important
##   I live alone                                    9.2                 2.9900
##   I live with my parents/family                   5.1                 1.6575
##   I live in a house-/ flat-share                  4.0                 1.3000
##   I live with my partner/children                21.3                 6.9225
```

```
##   Other                                            0.4                  0.1300
##
##                                    Other No barriers
##   I live alone                      1.84         9.2
##   I live with my parents/family     1.02         5.1
##   I live in a house-/ flat-share    0.80         4.0
##   I live with my partner/children   4.26        21.3
##   Other                             0.08         0.4
```

```
chi.liv.mainconsid.sim$observed
```

```
##
##                                    Value for money Price Quality Deals/discounts
##   I live alone                                  26    16      27               5
##   I live with my parents/family                 15    18       6               3
##   I live in a house-/ flat-share                17     9       4               4
##   I live with my partner/children               58    53      51              14
##   Other                                          3     1       0               0
##
##                                    Use-by-dates/longevity Convenience
##   I live alone                                          3           4
##   I live with my parents/family                         0           1
##   I live in a house-/ flat-share                        2           0
##   I live with my partner/children                       9           5
##   Other                                                 0           0
##
##                                    Ease of recycling packaging Sustainability
##   I live alone                                               5              2
##   I live with my parents/family                              2              3
##   I live in a house-/ flat-share                             3              1
##   I live with my partner/children                           12              7
##   Other                                                      0              0
##
##                                    Brand Ethics Other
##   I live alone                         0      2     2
##   I live with my parents/family        2      1     0
##   I live in a house-/ flat-share       0      0     0
##   I live with my partner/children      2      2     0
##   Other                                0      0     0
```

```
chi.liv.mainconsid.sim$expected
```

```
##
##                                    Value for money   Price Quality
##   I live alone                             27.3700 22.3100   20.24
##   I live with my parents/family            15.1725 12.3675   11.22
##   I live in a house-/ flat-share           11.9000  9.7000    8.80
##   I live with my partner/children          63.3675 51.6525   46.86
##   Other                                     1.1900  0.9700    0.88
##
##                                    Deals/discounts Use-by-dates/longevity
##   I live alone                               5.980                  3.220
##   I live with my parents/family              3.315                  1.785
```

```
##   I live in a house-/ flat-share           2.600                   1.400
##   I live with my partner/children         13.845                   7.455
##   Other                                    0.260                   0.140
##
##                                    Convenience Ease of recycling packaging
##   I live alone                           2.300                       5.060
##   I live with my parents/family          1.275                       2.805
##   I live in a house-/ flat-share         1.000                       2.200
##   I live with my partner/children        5.325                      11.715
##   Other                                  0.100                       0.220
##
##                                    Sustainability Brand Ethics Other
##   I live alone                             2.9900  0.92 1.1500 0.460
##   I live with my parents/family            1.6575  0.51 0.6375 0.255
##   I live in a house-/ flat-share           1.3000  0.40 0.5000 0.200
##   I live with my partner/children          6.9225  2.13 2.6625 1.065
##   Other                                    0.1300  0.04 0.0500 0.020
```

```
chi.liv.litter.sim$observed
```

```
##
##                                    Strongly agree Agree
##   I live alone                                 31    43
##   I live with my parents/family                23    18
##   I live in a house-/ flat-share               15    14
##   I live with my partner/children             104    85
##   Other                                         0     2
##
##                                    Neither agree nor disagree Disagree
##   I live alone                                             14        3
##   I live with my parents/family                             8        1
##   I live in a house-/ flat-share                            7        3
##   I live with my partner/children                          22        1
##   Other                                                     2        0
##
##                                    Strongly disagree
##   I live alone                                     1
##   I live with my parents/family                    1
##   I live in a house-/ flat-share                   1
##   I live with my partner/children                  1
##   Other                                            0
```

```
chi.liv.litter.sim$expected
```

```
##
##                                    Strongly agree  Agree
##   I live alone                            39.7900 37.260
##   I live with my parents/family           22.0575 20.655
##   I live in a house-/ flat-share          17.3000 16.200
##   I live with my partner/children         92.1225 86.265
##   Other                                    1.7300  1.620
##
##                                    Neither agree nor disagree Disagree
```

```
##   I live alone                                          12.1900     1.84
##   I live with my parents/family                          6.7575     1.02
##   I live in a house-/ flat-share                         5.3000     0.80
##   I live with my partner/children                       28.2225     4.26
##   Other                                                  0.5300     0.08
##
##                                    Strongly disagree
##   I live alone                                  0.92
##   I live with my parents/family                 0.51
##   I live in a house-/ flat-share                0.40
##   I live with my partner/children               2.13
##   Other                                         0.04
```

```
chi.liv.attitude.sim$observed
```

```
##
##                                    1 (Not concerned)  2  3  4  5  6
##   I live alone                                     6  4  9 16 25 12
##   I live with my parents/family                    8  2  5 14 13  6
##   I live in a house-/ flat-share                   5  2  7  6  6  4
##   I live with my partner/children                  7 12 19 36 69 34
##   Other                                            1  0  1  1  1  0
##
##                                    7 (Very concerned
##   I live alone                                    20
##   I live with my parents/family                    3
##   I live in a house-/ flat-share                  10
##   I live with my partner/children                 36
##   Other                                            0
```

```
chi.liv.attitude.sim$expected
```

```
##
##                                    1 (Not concerned)     2       3       4
##   I live alone                                6.2100  4.60  9.4300 16.7900
##   I live with my parents/family               3.4425  2.55  5.2275  9.3075
##   I live in a house-/ flat-share              2.7000  2.00  4.1000  7.3000
##   I live with my partner/children            14.3775 10.65 21.8325 38.8725
##   Other                                       0.2700  0.20  0.4100  0.7300
##
##                                         5     6 7 (Very concerned
##   I live alone                     26.220 12.88           15.8700
##   I live with my parents/family    14.535  7.14            8.7975
##   I live in a house-/ flat-share   11.400  5.60            6.9000
##   I live with my partner/children 60.705 29.82           36.7425
##   Other                             1.140  0.56            0.6900
```

```
chi.liv.ocean.sim$observed
```

```
##
##                                    Always Most of the time Sometimes Rarely
##   I live alone                          2               18        50     19
```

```
##   I live with my parents/family       5              15        26      5
##   I live in a house-/ flat-share      2              14        20      0
##   I live with my partner/children    10              56       103     35
##   Other                               0               1         1      1
##
##                                    Never
##   I live alone                         3
##   I live with my parents/family        0
##   I live in a house-/ flat-share       4
##   I live with my partner/children      9
##   Other                                1
```

```
chi.liv.ocean.sim$expected
```

```
##
##                                     Always Most of the time Sometimes Rarely
##   I live alone                      4.3700            23.92      46.0  13.80
##   I live with my parents/family     2.4225            13.26      25.5   7.65
##   I live in a house-/ flat-share    1.9000            10.40      20.0   6.00
##   I live with my partner/children 10.1175            55.38     106.5  31.95
##   Other                             0.1900             1.04       2.0   0.60
##
##                                     Never
##   I live alone                    3.9100
##   I live with my parents/family   2.1675
##   I live in a house-/ flat-share  1.7000
##   I live with my partner/children 9.0525
##   Other                           0.1700
```

```
chi.liv.actions.sim$observed
```

```
##
##                                   Yes, definitely Yes, probably
##   I live alone                                 39            38
##   I live with my parents/family                13            27
##   I live in a house-/ flat-share               13            20
##   I live with my partner/children              77           102
##   Other                                         0             4
##
##                                   No, probably not No, definitely not
##   I live alone                                  12                  1
##   I live with my parents/family                  6                  1
##   I live in a house-/ flat-share                 5                  1
##   I live with my partner/children               20                  8
##   Other                                          0                  0
##
##                                   I don't know
##   I live alone                               2
##   I live with my parents/family              4
##   I live in a house-/ flat-share             1
##   I live with my partner/children            6
##   Other                                      0
```

```r
chi.liv.actions.sim$expected
```

```
##
##                                   Yes, definitely Yes, probably
##   I live alone                             32.660       43.9300
##   I live with my parents/family            18.105       24.3525
##   I live in a house-/ flat-share           14.200       19.1000
##   I live with my partner/children          75.615      101.7075
##   Other                                     1.420        1.9100
##
##                                   No, probably not No, definitely not
##   I live alone                               9.8900             2.5300
##   I live with my parents/family              5.4825             1.4025
##   I live in a house-/ flat-share             4.3000             1.1000
##   I live with my partner/children           22.8975             5.8575
##   Other                                      0.4300             0.1100
##
##                                   I don't know
##   I live alone                          2.9900
##   I live with my parents/family         1.6575
##   I live in a house-/ flat-share        1.3000
##   I live with my partner/children       6.9225
##   Other                                 0.1300
```

```r
chi.liv.bottpurchase.sim$observed
```

```
##
##                                   None 1-2 3-5 6-10 11-15 16+ I don't know
##   I live alone                       5  43  27   11     1   3            2
##   I live with my parents/family      1  15  15    9     4   5            2
##   I live in a house-/ flat-share     0  14  16    6     2   1            1
##   I live with my partner/children    6  65  74   46    12   5            5
##   Other                              0   0   2    2     0   0            0
```

```r
chi.liv.bottpurchase.sim$expected
```

```
##
##                                   None     1-2    3-5   6-10   11-15   16+
##   I live alone                    2.76 31.5100 30.820 17.020  4.3700 3.220
##   I live with my parents/family   1.53 17.4675 17.085  9.435  2.4225 1.785
##   I live in a house-/ flat-share  1.20 13.7000 13.400  7.400  1.9000 1.400
##   I live with my partner/children 6.39 72.9525 71.355 39.405 10.1175 7.455
##   Other                           0.12  1.3700  1.340  0.740  0.1900 0.140
##
##                                   I don't know
##   I live alone                           2.300
##   I live with my parents/family          1.275
##   I live in a house-/ flat-share         1.000
##   I live with my partner/children        5.325
##   Other                                  0.100
```

```
chi.liv.bottreuse.sim$observed
```

```
##
##                                   Never Once 2-4 times 5-10 times More often
##   I live alone                       17   13        27         11         24
##   I live with my parents/family       8    9        19          4         11
##   I live in a house-/ flat-share      4    6        11         11          8
##   I live with my partner/children    40   38        71         30         34
##   Other                               2    0         1          1          0
```

```
chi.liv.bottreuse.sim$expected
```

```
##
##                                     Never   Once 2-4 times 5-10 times
##   I live alone                    16.3300 15.180   29.6700    13.1100
##   I live with my parents/family    9.0525  8.415   16.4475     7.2675
##   I live in a house-/ flat-share   7.1000  6.600   12.9000     5.7000
##   I live with my partner/children 37.8075 35.145   68.6925    30.3525
##   Other                            0.7100  0.660    1.2900     0.5700
##
##                                   More often
##   I live alone                       17.7100
##   I live with my parents/family       9.8175
##   I live in a house-/ flat-share      7.7000
##   I live with my partner/children    41.0025
##   Other                               0.7700
```

```
chi.liv.bottdisp.sim$observed
```

```
##
##                                   General waste bin Recycling bin
##   I live alone                                    7            75
##   I live with my parents/family                   9            32
##   I live in a house-/ flat-share                  3            32
##   I live with my partner/children                 7           186
##   Other                                           1             3
##
##                                   Recycling centre Specialist waste collection
##   I live alone                                   3                           0
##   I live with my parents/family                  2                           2
##   I live in a house-/ flat-share                 1                           1
##   I live with my partner/children                4                           4
##   Other                                          0                           0
##
##                                   Landfill Deposit return scheme
##   I live alone                           1                     1
##   I live with my parents/family          1                     1
##   I live in a house-/ flat-share         0                     0
##   I live with my partner/children        3                     2
##   Other                                  0                     0
##
##                                   Indefinite storage Other I don't know
```

```
##   I live alone                                     4     1             0
##   I live with my parents/family                    4     0             0
##   I live in a house-/ flat-share                   1     1             1
##   I live with my partner/children                  7     0             0
##   Other                                            0     0             0
```

```
chi.liv.bottdisp.sim$expected
```

```
##
##                                    General waste bin Recycling bin
##   I live alone                                6.2100         75.44
##   I live with my parents/family               3.4425         41.82
##   I live in a house-/ flat-share              2.7000         32.80
##   I live with my partner/children            14.3775        174.66
##   Other                                       0.2700          3.28
##
##                                    Recycling centre Specialist waste collection
##   I live alone                                2.300                      1.6100
##   I live with my parents/family               1.275                      0.8925
##   I live in a house-/ flat-share              1.000                      0.7000
##   I live with my partner/children             5.325                      3.7275
##   Other                                       0.100                      0.0700
##
##                                    Landfill Deposit return scheme
##   I live alone                       1.1500                  0.92
##   I live with my parents/family      0.6375                  0.51
##   I live in a house-/ flat-share     0.5000                  0.40
##   I live with my partner/children    2.6625                  2.13
##   Other                              0.0500                  0.04
##
##                                    Indefinite storage Other I don't know
##   I live alone                                   3.68 0.460       0.2300
##   I live with my parents/family                  2.04 0.255       0.1275
##   I live in a house-/ flat-share                 1.60 0.200       0.1000
##   I live with my partner/children                8.52 1.065       0.5325
##   Other                                          0.16 0.020       0.0100
```

```
chi.liv.tubspurchase.sim$observed
```

```
##
##                                    None 1-2 3-5 6-10 11-15 16+ I don't know
##   I live alone                        4  33  35   12     2   3            3
##   I live with my parents/family       1   8  22   11     5   2            2
##   I live in a house-/ flat-share      0  15   9    9     5   1            1
##   I live with my partner/children     7  51  77   52    15   7            4
##   Other                               0   1   0    1     2   0            0
```

```
chi.liv.tubspurchase.sim$expected
```

```
##
##                                    None   1-2     3-5    6-10   11-15    16+
##   I live alone                     2.76 24.84 32.8900 19.5500  6.6700 2.9900
```

```
##   I live with my parents/family   1.53 13.77 18.2325 10.8375  3.6975 1.6575
##   I live in a house-/ flat-share  1.20 10.80 14.3000  8.5000  2.9000 1.3000
##   I live with my partner/children 6.39 57.51 76.1475 45.2625 15.4425 6.9225
##   Other                           0.12  1.08  1.4300  0.8500  0.2900 0.1300
##
##                                   I don't know
##   I live alone                           2.300
##   I live with my parents/family          1.275
##   I live in a house-/ flat-share         1.000
##   I live with my partner/children        5.325
##   Other                                  0.100
```

```
chi.liv.tubreuse.sim$observed
```

```
##
##                                   Never Once 2-4 times 5-10 times More often
##   I live alone                       19    9        17         10         37
##   I live with my parents/family       6    5        10          8         22
##   I live in a house-/ flat-share      4    3        12          7         14
##   I live with my partner/children    30   24        57         32         70
##   Other                               2    0         1          1          0
```

```
chi.liv.tubreuse.sim$expected
```

```
##
##                                     Never    Once 2-4 times 5-10 times
##   I live alone                    14.0300  9.4300   22.3100     13.340
##   I live with my parents/family    7.7775  5.2275   12.3675      7.395
##   I live in a house-/ flat-share   6.1000  4.1000    9.7000      5.800
##   I live with my partner/children 32.4825 21.8325   51.6525     30.885
##   Other                            0.6100  0.4100    0.9700      0.580
##
##                                   More often
##   I live alone                       32.8900
##   I live with my parents/family      18.2325
##   I live in a house-/ flat-share     14.3000
##   I live with my partner/children    76.1475
##   Other                               1.4300
```

```
chi.liv.tubdisp.sim$observed
```

```
##
##                                   General waste bin Recycling bin
##   I live alone                                   19            46
##   I live with my parents/family                  10            21
##   I live in a house-/ flat-share                  8            21
##   I live with my partner/children                47           121
##   Other                                           2             2
##
##                                   Recycling centre Specialist waste collection
##   I live alone                                   5                           3
##   I live with my parents/family                  2                           2
```

```
##   I live in a house-/ flat-share                2                             1
##   I live with my partner/children              20                             6
##   Other                                         0                             0
##
##                                    Landfill Deposit return scheme
##   I live alone                            0                      0
##   I live with my parents/family           2                      2
##   I live in a house-/ flat-share          1                      0
##   I live with my partner/children         0                      2
##   Other                                   0                      0
##
##                                    Indefinite storage Other I don't know
##   I live alone                                    15     2            2
##   I live with my parents/family                    8     1            3
##   I live in a house-/ flat-share                   5     1            1
##   I live with my partner/children                 14     3            0
##   Other                                            0     0            0
```

```
chi.liv.tubdisp.sim$expected
```

```
##
##                                    General waste bin Recycling bin
##   I live alone                                19.780       48.5300
##   I live with my parents/family               10.965       26.9025
##   I live in a house-/ flat-share               8.600       21.1000
##   I live with my partner/children             45.795      112.3575
##   Other                                        0.860        2.1100
##
##                                    Recycling centre Specialist waste collection
##   I live alone                               6.6700                        2.76
##   I live with my parents/family              3.6975                        1.53
##   I live in a house-/ flat-share             2.9000                        1.20
##   I live with my partner/children           15.4425                        6.39
##   Other                                      0.2900                        0.12
##
##                                    Landfill Deposit return scheme
##   I live alone                       0.6900                   0.92
##   I live with my parents/family      0.3825                   0.51
##   I live in a house-/ flat-share     0.3000                   0.40
##   I live with my partner/children    1.5975                   2.13
##   Other                              0.0300                   0.04
##
##                                    Indefinite storage  Other I don't know
##   I live alone                                  9.660 1.6100        1.380
##   I live with my parents/family                 5.355 0.8925        0.765
##   I live in a house-/ flat-share                4.200 0.7000        0.600
##   I live with my partner/children              22.365 3.7275        3.195
##   Other                                         0.420 0.0700        0.060
```

```
chi.liv.filmpurchase.sim$observed
```

```
##
##                                    None 1-2 3-5 6-10 11-15 16+ I don't know
```

```
##   I live alone                       8  21  34   21     3   3            2
##   I live with my parents/family      2   8  13   17     2   5            4
##   I live in a house-/ flat-share     0   8   8   13     9   1            1
##   I live with my partner/children    2  47  67   60    24   8            5
##   Other                              0   0   0    2     1   1            0
```

```
chi.liv.filmpurchase.sim$expected
```

```
##
##                                   None   1-2    3-5    6-10   11-15   16+
##   I live alone                    2.76 19.32 28.060 25.9900  8.9700 4.140
##   I live with my parents/family   1.53 10.71 15.555 14.4075  4.9725 2.295
##   I live in a house-/ flat-share  1.20  8.40 12.200 11.3000  3.9000 1.800
##   I live with my partner/children 6.39 44.73 64.965 60.1725 20.7675 9.585
##   Other                           0.12  0.84  1.220  1.1300  0.3900 0.180
##
##                                   I don't know
##   I live alone                            2.76
##   I live with my parents/family           1.53
##   I live in a house-/ flat-share          1.20
##   I live with my partner/children         6.39
##   Other                                   0.12
```

```
chi.liv.filmreuse.sim$observed
```

```
##
##                                   Never Once 2-4 times 5-10 times More often
##   I live alone                       57   16        13          2          4
##   I live with my parents/family      28    9         9          3          2
##   I live in a house-/ flat-share     15   11         6          4          4
##   I live with my partner/children   133   46        26          3          5
##   Other                               3    1         0          0          0
```

```
chi.liv.filmreuse.sim$expected
```

```
##
##                                    Never    Once 2-4 times 5-10 times
##   I live alone                     54.28 19.0900    12.420       2.76
##   I live with my parents/family    30.09 10.5825     6.885       1.53
##   I live in a house-/ flat-share   23.60  8.3000     5.400       1.20
##   I live with my partner/children 125.67 44.1975    28.755       6.39
##   Other                             2.36  0.8300     0.540       0.12
##
##                                   More often
##   I live alone                        3.4500
##   I live with my parents/family       1.9125
##   I live in a house-/ flat-share      1.5000
##   I live with my partner/children     7.9875
##   Other                               0.1500
```

```
chi.liv.filmdisp.sim$observed
```

```
##
##                                   General waste bin Recycling bin
##   I live alone                                   61            22
##   I live with my parents/family                  32            11
##   I live in a house-/ flat-share                 21            13
##   I live with my partner/children               148            45
##   Other                                           3             1
##
##                                   Recycling centre Specialist waste collection
##   I live alone                                   4                           1
##   I live with my parents/family                  3                           0
##   I live in a house-/ flat-share                 3                           0
##   I live with my partner/children                7                           5
##   Other                                          0                           0
##
##                                   Landfill Deposit return scheme
##   I live alone                           0                     0
##   I live with my parents/family          1                     0
##   I live in a house-/ flat-share         0                     0
##   I live with my partner/children        2                     2
##   Other                                  0                     0
##
##                                   Indefinite storage Other I don't know
##   I live alone                                    1     2            1
##   I live with my parents/family                   2     1            1
##   I live in a house-/ flat-share                  0     1            2
##   I live with my partner/children                 0     2            2
##   Other                                           0     0            0
```

```
chi.liv.filmdisp.sim$expected
```

```
##
##                                   General waste bin Recycling bin
##   I live alone                             60.9500         21.16
##   I live with my parents/family            33.7875         11.73
##   I live in a house-/ flat-share           26.5000          9.20
##   I live with my partner/children         141.1125         48.99
##   Other                                     2.6500          0.92
##
##                                   Recycling centre Specialist waste collection
##   I live alone                              3.9100                       1.380
##   I live with my parents/family             2.1675                       0.765
##   I live in a house-/ flat-share            1.7000                       0.600
##   I live with my partner/children           9.0525                       3.195
##   Other                                     0.1700                       0.060
##
##                                   Landfill Deposit return scheme
##   I live alone                      0.6900                 0.460
##   I live with my parents/family     0.3825                 0.255
##   I live in a house-/ flat-share    0.3000                 0.200
```

```
##   I live with my partner/children   1.5975                 1.065
##   Other                             0.0300                 0.020
##
##                                    Indefinite storage Other I don't know
##   I live alone                                 0.6900 1.380        1.380
##   I live with my parents/family                0.3825 0.765        0.765
##   I live in a house-/ flat-share               0.3000 0.600        0.600
##   I live with my partner/children              1.5975 3.195        3.195
##   Other                                        0.0300 0.060        0.060
```

```
chi.liv.bagpurchase.sim$observed
```

```
##
##                                    None 1-2 3-5 6-10 11-15 16+ I don't know
##   I live alone                       61  17   3    4     2   3            2
##   I live with my parents/family      22   8  11    3     4   2            1
##   I live in a house-/ flat-share     12  12  11    2     2   0            1
##   I live with my partner/children  135  38  18   10     6   3            3
##   Other                               2   2   0    0     0   0            0
```

```
chi.liv.bagpurchase.sim$expected
```

```
##
##                                      None     1-2     3-5    6-10 11-15  16+
##   I live alone                      53.36 17.7100  9.8900  4.3700 3.220 1.84
##   I live with my parents/family     29.58  9.8175  5.4825  2.4225 1.785 1.02
##   I live in a house-/ flat-share    23.20  7.7000  4.3000  1.9000 1.400 0.80
##   I live with my partner/children 123.54 41.0025 22.8975 10.1175 7.455 4.26
##   Other                              2.32  0.7700  0.4300  0.1900 0.140 0.08
##
##                                    I don't know
##   I live alone                           1.6100
##   I live with my parents/family          0.8925
##   I live in a house-/ flat-share         0.7000
##   I live with my partner/children        3.7275
##   Other                                  0.0700
```

```
chi.liv.bagreuse.sim$observed
```

```
##
##                                    Never Once 2-4 times 5-10 times More often
##   I live alone                         7    9        15         13         48
##   I live with my parents/family        2    7        17          4         21
##   I live in a house-/ flat-share       0    4        10         16         10
##   I live with my partner/children     22   24        51         36         80
##   Other                                0    0         0          1          3
```

```
chi.liv.bagreuse.sim$expected
```

```
##
##                                      Never  Once 2-4 times 5-10 times More often
```

```
##   I live alone                     7.1300 10.12   21.3900     16.100     37.260
##   I live with my parents/family    3.9525  5.61   11.8575      8.925     20.655
##   I live in a house-/ flat-share   3.1000  4.40    9.3000      7.000     16.200
##   I live with my partner/children 16.5075 23.43   49.5225     37.275     86.265
##   Other                            0.3100  0.44    0.9300      0.700      1.620
```

```
chi.liv.bagdisp.sim$observed
```

```
##
##                                   General waste bin Recycling bin
##   I live alone                                   31            26
##   I live with my parents/family                  14             9
##   I live in a house-/ flat-share                 13             8
##   I live with my partner/children                84            48
##   Other                                           1             1
##
##                                   Recycling centre Specialist waste collection
##   I live alone                                   4                           0
##   I live with my parents/family                  6                           4
##   I live in a house-/ flat-share                 3                           0
##   I live with my partner/children               16                           4
##   Other                                          0                           0
##
##                                   Landfill Deposit return scheme
##   I live alone                           0                     0
##   I live with my parents/family          0                     2
##   I live in a house-/ flat-share         0                     1
##   I live with my partner/children        2                     8
##   Other                                  0                     0
##
##                                   Indefinite storage Other I don't know
##   I live alone                                    23     6            2
##   I live with my parents/family                   13     1            2
##   I live in a house-/ flat-share                   6     7            2
##   I live with my partner/children                 39     9            3
##   Other                                            2     0            0
```

```
chi.liv.bagdisp.sim$expected
```

```
##
##                                   General waste bin Recycling bin
##   I live alone                              32.8900         21.16
##   I live with my parents/family             18.2325         11.73
##   I live in a house-/ flat-share            14.3000          9.20
##   I live with my partner/children           76.1475         48.99
##   Other                                      1.4300          0.92
##
##                                   Recycling centre Specialist waste collection
##   I live alone                              6.6700                        1.84
##   I live with my parents/family             3.6975                        1.02
##   I live in a house-/ flat-share            2.9000                        0.80
##   I live with my partner/children          15.4425                        4.26
##   Other                                     0.2900                        0.08
```

```
##
##                                  Landfill Deposit return scheme
##   I live alone                      0.460                2.5300
##   I live with my parents/family     0.255                1.4025
##   I live in a house-/ flat-share    0.200                1.1000
##   I live with my partner/children   1.065                5.8575
##   Other                             0.020                0.1100
##
##                                  Indefinite storage   Other I don't know
##   I live alone                              19.0900  5.2900       2.0700
##   I live with my parents/family             10.5825  2.9325       1.1475
##   I live in a house-/ flat-share             8.3000  2.3000       0.9000
##   I live with my partner/children           44.1975 12.2475       4.7925
##   Other                                      0.8300  0.2300       0.0900
```

Postcode

```r
chi.post.choice <- chisq.test(post.choice)
```

```
## Warning in chisq.test(post.choice): Chi-squared approximation may be incorrect
```

```r
chi.post.barrier <- chisq.test(post.barrier)
```

```
## Warning in chisq.test(post.barrier): Chi-squared approximation may be incorrect
```

```r
chi.post.barrier2 <- chisq.test(post.barrier2)
```

```
## Warning in chisq.test(post.barrier2): Chi-squared approximation may be incorrect
```

```r
chi.post.mainconsid <- chisq.test(post.mainconsid)
```

```
## Warning in chisq.test(post.mainconsid): Chi-squared approximation may be
## incorrect
```

```r
chi.post.litter <- chisq.test(post.litter)
```

```
## Warning in chisq.test(post.litter): Chi-squared approximation may be incorrect
```

```r
chi.post.attitude <- chisq.test(post.attitude)
```

```
## Warning in chisq.test(post.attitude): Chi-squared approximation may be incorrect
```

```r
chi.post.ocean <- chisq.test(post.ocean)
```

```
## Warning in chisq.test(post.ocean): Chi-squared approximation may be incorrect
```

```
chi.post.actions <- chisq.test(post.actions)
```

```
## Warning in chisq.test(post.actions): Chi-squared approximation may be incorrect
```

```
chi.post.zerow <- chisq.test(post.zerow)
```

```
## Warning in chisq.test(post.zerow): Chi-squared approximation may be incorrect
```

```
chi.post.attplast <- chisq.test(post.attplast)
```

```
## Warning in chisq.test(post.attplast): Chi-squared approximation may be incorrect
```

```
chi.post.bottpurchase <- chisq.test(post.bottpurchase)
```

```
## Warning in chisq.test(post.bottpurchase): Chi-squared approximation may be
## incorrect
```

```
chi.post.bottdisp <- chisq.test(post.bottdisp)
```

```
## Warning in chisq.test(post.bottdisp): Chi-squared approximation may be incorrect
```

```
chi.post.tubspurchase <- chisq.test(post.tubspurchase)
```

```
## Warning in chisq.test(post.tubspurchase): Chi-squared approximation may be
## incorrect
```

```
chi.post.tubdisp <- chisq.test(post.tubdisp)
```

```
## Warning in chisq.test(post.tubdisp): Chi-squared approximation may be incorrect
```

```
chi.post.filmpurchase <- chisq.test(post.filmpurchase)
```

```
## Warning in chisq.test(post.filmpurchase): Chi-squared approximation may be
## incorrect
```

```
chi.post.filmreuse <- chisq.test(post.filmreuse)
```

```
## Warning in chisq.test(post.filmreuse): Chi-squared approximation may be
## incorrect
```

```
chi.post.filmdisp <- chisq.test(post.filmdisp)
```

```
## Warning in chisq.test(post.filmdisp): Chi-squared approximation may be incorrect
```

```r
chi.post.bagpurchase <- chisq.test(post.bagpurchase)
```

```
## Warning in chisq.test(post.bagpurchase): Chi-squared approximation may be
## incorrect
```

```r
chi.post.bagreuse <- chisq.test(post.bagreuse)
```

```
## Warning in chisq.test(post.bagreuse): Chi-squared approximation may be incorrect
```

```r
chi.post.bagdisp <- chisq.test(post.bagdisp)
```

```
## Warning in chisq.test(post.bagdisp): Chi-squared approximation may be incorrect
```

```r
chi.post.choice.sim <- chisq.test(post.choice, simulate.p.value = TRUE)
chi.post.choice.sim
```

```
##
##  Pearson's Chi-squared test with simulated p-value (based on 2000
##  replicates)
##
## data:  post.choice
## X-squared = 19.059, df = NA, p-value = 0.1979
```

```r
chi.post.barrier.sim <- chisq.test(post.barrier, simulate.p.value = TRUE)
chi.post.barrier.sim
```

```
##
##  Pearson's Chi-squared test with simulated p-value (based on 2000
##  replicates)
##
## data:  post.barrier
## X-squared = 47.618, df = NA, p-value = 0.7611
```

```r
chi.post.barrier2.sim <- chisq.test(post.barrier2, simulate.p.value = TRUE)
chi.post.barrier2.sim
```

```
##
##  Pearson's Chi-squared test with simulated p-value (based on 2000
##  replicates)
##
## data:  post.barrier2
## X-squared = 37.022, df = NA, p-value = 0.3748
```

```r
chi.post.mainconsid.sim <- chisq.test(post.mainconsid, simulate.p.value = TRUE)
chi.post.mainconsid.sim
```

```
##
##  Pearson's Chi-squared test with simulated p-value (based on 2000
##  replicates)
##
## data:  post.mainconsid
## X-squared = 46.348, df = NA, p-value = 0.6167
```

```r
chi.post.litter.sim <- chisq.test(post.litter, simulate.p.value = TRUE)
chi.post.litter.sim
```

```
##
##  Pearson's Chi-squared test with simulated p-value (based on 2000
##  replicates)
##
## data:  post.litter
## X-squared = 23.412, df = NA, p-value = 0.2674
```

```r
chi.post.attitude.sim <- chisq.test(post.attitude, simulate.p.value = TRUE)
chi.post.attitude.sim
```

```
##
##  Pearson's Chi-squared test with simulated p-value (based on 2000
##  replicates)
##
## data:  post.attitude
## X-squared = 28.904, df = NA, p-value = 0.5287
```

```r
chi.post.ocean.sim <- chisq.test(post.ocean, simulate.p.value = TRUE)
chi.post.ocean.sim
```

```
##
##  Pearson's Chi-squared test with simulated p-value (based on 2000
##  replicates)
##
## data:  post.ocean
## X-squared = 10.852, df = NA, p-value = 0.963
```

```r
chi.post.actions.sim <- chisq.test(post.actions, simulate.p.value = TRUE)
chi.post.actions.sim
```

```
##
##  Pearson's Chi-squared test with simulated p-value (based on 2000
##  replicates)
##
## data:  post.actions
## X-squared = 25.422, df = NA, p-value = 0.1744
```

```r
chi.post.attplast.sim <- chisq.test(post.attplast, simulate.p.value = TRUE)
chi.post.attplast.sim
```

```
##
##  Pearson's Chi-squared test with simulated p-value (based on 2000
##  replicates)
##
## data:  post.attplast
## X-squared = 12.939, df = NA, p-value = 0.6122
```

```r
chi.post.zerow.sim <- chisq.test(post.zerow, simulate.p.value = TRUE)
chi.post.zerow.sim
```

```
##
##  Pearson's Chi-squared test with simulated p-value (based on 2000
##  replicates)
##
## data:  post.zerow
## X-squared = 47.264, df = NA, p-value = 0.003498
```

```r
chi.post.bottpurchase.sim <- chisq.test(post.bottpurchase, simulate.p.value = TRUE)
chi.post.bottpurchase.sim
```

```
##
##  Pearson's Chi-squared test with simulated p-value (based on 2000
##  replicates)
##
## data:  post.bottpurchase
## X-squared = 53.875, df = NA, p-value = 0.004498
```

```r
chi.post.bottdisp.sim <- chisq.test(post.bottdisp, simulate.p.value = TRUE)
chi.post.bottdisp.sim
```

```
##
##  Pearson's Chi-squared test with simulated p-value (based on 2000
##  replicates)
##
## data:  post.bottdisp
## X-squared = 34.676, df = NA, p-value = 0.7546
```

```r
chi.post.tubspurchase.sim <- chisq.test(post.tubspurchase, simulate.p.value = TRUE)
chi.post.tubspurchase.sim
```

```
##
##  Pearson's Chi-squared test with simulated p-value (based on 2000
##  replicates)
##
## data:  post.tubspurchase
## X-squared = 33.853, df = NA, p-value = 0.2959
```

```r
chi.post.tubdisp.sim <- chisq.test(post.tubdisp, simulate.p.value = TRUE)
chi.post.tubdisp.sim
```

```
##
##  Pearson's Chi-squared test with simulated p-value (based on 2000
##  replicates)
##
## data:  post.tubdisp
## X-squared = 40.575, df = NA, p-value = 0.4338
```

```r
chi.post.filmpurchase.sim <- chisq.test(post.filmpurchase, simulate.p.value = TRUE)
chi.post.filmpurchase.sim
```

```
##
##  Pearson's Chi-squared test with simulated p-value (based on 2000
##  replicates)
##
## data:  post.filmpurchase
## X-squared = 44.685, df = NA, p-value = 0.03948
```

```r
chi.post.filmreuse.sim <- chisq.test(post.filmreuse, simulate.p.value = TRUE)
chi.post.filmreuse.sim
```

```
##
##  Pearson's Chi-squared test with simulated p-value (based on 2000
##  replicates)
##
## data:  post.filmreuse
## X-squared = 23.012, df = NA, p-value = 0.2864
```

```r
chi.post.filmdisp.sim <- chisq.test(post.filmdisp, simulate.p.value = TRUE)
chi.post.filmdisp.sim
```

```
##
##  Pearson's Chi-squared test with simulated p-value (based on 2000
##  replicates)
##
## data:  post.filmdisp
## X-squared = 34.581, df = NA, p-value = 0.7551
```

```r
chi.post.bagpurchase.sim <- chisq.test(post.bagpurchase, simulate.p.value = TRUE)
chi.post.bagpurchase.sim
```

```
##
##  Pearson's Chi-squared test with simulated p-value (based on 2000
##  replicates)
##
## data:  post.bagpurchase
## X-squared = 36.374, df = NA, p-value = 0.1824
```

```r
chi.post.bagreuse.sim <- chisq.test(post.bagreuse, simulate.p.value = TRUE)
chi.post.bagreuse.sim
```

```
##
##  Pearson's Chi-squared test with simulated p-value (based on 2000
##  replicates)
##
## data:  post.bagreuse
## X-squared = 30.598, df = NA, p-value = 0.05347
```

```r
chi.post.bagdisp.sim <- chisq.test(post.bagdisp, simulate.p.value = TRUE)
chi.post.bagdisp.sim
```

```
##
##  Pearson's Chi-squared test with simulated p-value (based on 2000
##  replicates)
##
## data:  post.bagdisp
## X-squared = 63.609, df = NA, p-value = 0.007996
```

Simulated residuals for

```r
chi.post.choice.sim$residuals
```

```
##
##             Always As often as they can If cheaper or preferred Rarely or never
##   P01  0.023074903         -0.814692287             0.668417242     0.802246243
##   P02 -1.666064450          1.310954385            -0.032000522    -1.325926396
##   P03 -0.688764567          0.895444602            -0.055708601    -1.395388420
##   P04  1.488196757         -0.251151196            -0.365017829    -0.463724757
##   P05  0.250217297         -0.071909250            -0.192582768     0.258198890
##   P06  0.325817561         -0.922855639             0.004716666     2.017264197
```

```r
chi.post.barrier.sim$residuals
```

```
##
##       Council collection Unclear information Difficult transport
##   P01        -0.18803615          0.09326301         -1.01514997
##   P02         0.69443961         -0.69721552          1.27562493
##   P03         0.50954727         -0.57875772         -0.40924351
##   P04        -0.32438048          1.76432208          0.65254148
##   P05        -0.15385544         -1.24526343         -0.24553358
##   P06        -0.43226304          0.44838710         -0.36723933
##
##       No local facilities  No support Ends up in landfills  Forgetting
##   P01          0.39171677 -0.05958796           1.38627162 -1.59511255
##   P02         -0.73862221  0.59766662          -1.90490281  0.43082022
##   P03         -0.13884677 -0.13805418           1.96832092  0.53571429
##   P04         -0.81198556  1.20253797          -1.06306846  0.82823645
##   P05          1.07570575 -0.68888889           0.84522824 -0.23570226
##   P06          0.24686980 -1.07164497          -0.93516351  0.06350006
##
##       Recycling a hassle Household disagrees Recycling not important
##   P01         0.20938142         -0.92870878              0.14805502
##   P02        -0.50602431          0.19266866             -0.90829511
##   P03        -0.20328928         -0.78262379             -0.78262379
##   P04        -0.73335181         -1.01242284             -0.02469324
##   P05         0.14907120          1.15950181              0.10540926
##   P06         1.16466803          1.39150649              1.39150649
##
##             Other Already doing everything
```

```
##   P01  0.37319614              0.69123724
##   P02  0.41851107              0.52009333
##   P03 -0.70000000             -0.75025297
##   P04 -0.90553851             -0.78547060
##   P05  0.32998316              0.23102450
##   P06  0.48260048              0.05551121
```

```r
chi.post.barrier2.sim$residuals
```

```
##
##       Limited alternatives No SUP-free alternatives Alternatives expensive
##   P01           1.13573559               0.08813933            -0.95647499
##   P02          -0.48007435              -0.27369851             0.52737030
##   P03          -0.91103714               0.18206663             1.11176860
##   P04          -0.10476454              -0.34137245            -0.82049168
##   P05          -0.79504639               1.20157848            -0.07669650
##   P06           1.08434609              -0.87471871             0.50279107
##
##       Limited functioning Forgetting reusables Reducing not important
##   P01          1.26169041          -0.72331764            -0.16193679
##   P02         -0.56568886           1.71269768             0.58378437
##   P03         -0.35571326          -0.40657856            -1.26194295
##   P04         -0.35189561          -0.41905818             0.81777280
##   P05         -0.38497419           0.67082039             1.08517595
##   P06          0.38842325          -0.88354126            -1.41950696
##
##             Other No barriers
##   P01 -1.17473401 -0.34262414
##   P02  0.59186403 -1.40129810
##   P03  0.02020305  0.94868330
##   P04 -0.49975604  2.37466301
##   P05  0.46666667 -1.56524758
##   P06  0.68250015 -0.08032193
```

```r
chi.post.mainconsid.sim$residuals
```

```
##
##       Value for money        Price      Quality Deals/discounts
##   P01     0.325002928 -0.179071805 -0.816189934     0.715371129
##   P02    -0.143303987  0.248711142 -1.711056252     0.825595780
##   P03     0.896400173 -0.836210092  0.676150843    -0.663993200
##   P04    -0.687368091  1.147051297 -0.480299242     0.290209215
##   P05     0.773523110 -0.588725690  1.045239328    -0.776580275
##   P06    -1.034981139 -0.009026436  1.451301368    -0.513079546
##
##       Use-by-dates/longevity  Convenience Ease of recycling packaging
##   P01            1.663420234  0.209381422                -1.434748972
##   P02            0.453986670  0.272474630                 1.768791279
##   P03           -0.545976537 -1.106797181                 0.794934100
##   P04           -1.694107435 -0.034921515                -0.240149621
##   P05           -0.327569210 -0.596284794                -0.482418151
##   P06            0.563440714  1.164668027                -0.222027448
##
```

```
##       Sustainability        Brand       Ethics        Other
##   P01    0.505843777  0.373196145  0.148055023 -0.587367006
##   P02   -0.099004368  0.418511069  0.192668659 -0.574456265
##   P03   -1.261942946  0.728571429  0.495129338 -0.494974747
##   P04    0.205208904  0.198776747  0.963036357  2.483162814
##   P05    0.431455497 -0.848528137 -0.948683298 -0.600000000
##   P06   -0.010567049 -0.787400787 -0.880340843 -0.556776436
```

```
chi.post.litter.sim$residuals
```

```
##
##       Strongly agree       Agree Neither agree nor disagree    Disagree
##   P01    -0.70339014  0.57790860                -0.37785374 -0.32347748
##   P02     1.58251774 -1.30171403                 0.08623037 -1.14891253
##   P03    -0.47626482  0.48375083                 0.19917282  0.02020305
##   P04     0.25775577 -0.38349335                 0.04095613  0.28111277
##   P05    -1.10029482  1.26666667                -0.49859341  1.30000000
##   P06     0.42195151 -0.62063610                 0.62277968 -0.21552636
##
##       Strongly disagree
##   P01        2.78091321
##   P02       -0.81240384
##   P03       -0.70000000
##   P04        0.19877675
##   P05       -0.84852814
##   P06       -0.78740079
```

```
chi.post.attitude.sim$residuals
```

```
##
##       1 (Not concerned)           2           3           4           5
##   P01        0.15870264 -0.78065376  0.72478285 -1.01237357 -1.05197319
##   P02        0.25820976 -0.16514456  0.85929766  0.27516929 -1.57019183
##   P03       -0.71893855 -0.28749445 -0.90246124  1.02243802  0.54455775
##   P04       -0.65245350  0.44447832 -1.17448712  1.30155077  0.33717724
##   P05       -0.39010392  1.26491106 -0.13987993 -1.41796476  1.20973955
##   P06        1.37603876 -0.62475802  0.65254148 -0.09364472  0.55429077
##
##                  6 7 (Very concerned
##   P01  0.43113793        1.76739130
##   P02  0.25002164        0.77500620
##   P03  0.43525402       -0.84356087
##   P04 -0.43680819       -0.30444161
##   P05 -0.34016803       -0.11917591
##   P06 -0.23080704       -1.43563885
```

```
chi.post.ocean.sim$residuals
```

```
##
##            Always Most of the time   Sometimes      Rarely       Never
##   P01  0.95145418       0.25026185 -0.59587957  0.20204271  0.03941711
##   P02 -0.07624564       0.20277791  0.17407766 -0.28603878 -0.48065055
```

```
##   P03 -1.52561463       0.35300904  0.70710678 -0.86681056 -0.05716911
##   P04  1.06659212       0.14727035 -0.46852129 -0.37067287  0.81154245
##   P05  0.31362808      -0.39753514  0.16666667  0.36514837 -0.60596167
##   P06 -1.13338368      -0.52802361  0.17960530  0.88536488  0.22485500
```

```r
chi.post.actions.sim$residuals
```

```
##
##       Yes, definitely Yes, probably No, probably not No, definitely not
##   P01     -0.70616869   -0.33928623       1.68257215         0.07441029
##   P02      1.35731055   -1.69492364       1.09061116        -0.60494974
##   P03      0.38482459    0.33129380      -0.98797288        -0.29935769
##   P04     -1.31779695    0.61447289       0.06231040         2.49389721
##   P05      0.28482760    0.61738438      -0.98487266        -1.40712473
##   P06      0.21102076    0.44017258      -1.03227910        -0.53991690
##
##       I don't know
##   P01   0.50584378
##   P02   0.58378437
##   P03  -0.46951409
##   P04  -0.40735499
##   P05  -0.22226495
##   P06  -0.01056705
```

```r
chi.post.attplast.sim$residuals
```

```
##
##       Will go out of their way to avoid
##   P01                        0.57335083
##   P02                       -0.44607618
##   P03                        0.70866274
##   P04                       -0.19900518
##   P05                       -0.31739682
##   P06                       -0.20371316
##
##       If option is readily available, will avoid
##   P01                                -0.75094277
##   P02                                 0.17173824
##   P03                                 0.83112602
##   P04                                -0.25280259
##   P05                                -0.19795077
##   P06                                 0.38018780
##
##       Will avoid only without extra costs Not a priority
##   P01                          1.26301421    -0.92142197
##   P02                          0.13899926     0.19419964
##   P03                         -1.91519811    -1.03250061
##   P04                          0.32190821     0.70161360
##   P05                          0.79230011     0.02010076
##   P06                         -0.99722083     0.86103327
```

```
chi.post.zerow.sim$residuals
```

```
##
##       Yes, shops regularly Yes, shops occasionally Yes, visited at least once
##   P01           0.37644133             -0.95610169                 0.42266694
##   P02          -1.51986842             -1.77539297                -0.48241815
##   P03          -0.54597654              0.16953317                -1.13143098
##   P04          -0.51354476             -0.06048584                 0.48690160
##   P05           2.19219394              1.54919334                 0.80829038
##   P06          -0.11540352              1.08978641                -0.37330261
##
##       Yes, never visited No, but would like to shop there
##   P01        -0.04619943                     -0.03412618
##   P02        -0.92376043                      0.76764947
##   P03         0.37157839                      0.33746828
##   P04         0.93234559                      0.68869669
##   P05         1.54775824                     -1.51448037
##   P06        -2.06864598                     -0.21600691
##
##       No, not likely to shop there
##   P01                   0.31811594
##   P02                   1.96048413
##   P03                  -0.15564171
##   P04                  -2.16565670
##   P05                  -1.82168898
##   P06                   2.23372562
```

```
chi.post.bottpurchase.sim$residuals
```

```
##
##              None         1-2         3-5        6-10       11-15         16+
##   P01 -0.74370141  0.07559664 -0.43990958  0.62555733  1.50382236 -0.26704812
##   P02 -1.40712473 -0.33757695  0.82728515  0.22608370  1.05331935 -1.51986842
##   P03  4.56106713  0.29719441 -0.84288876 -0.68586107 -0.21466758 -0.54597654
##   P04 -0.29328529 -0.39343124  0.67351206 -0.30039526 -0.45349166 -0.51354476
##   P05 -0.78928003  0.87396330 -0.02443389 -0.08767946 -0.76784806  0.30237158
##   P06 -0.63058259 -0.48501078 -0.38837836  0.15649273 -1.13338368  2.59997341
##
##       I don't know
##   P01  -1.31339255
##   P02  -0.50602431
##   P03  -0.20328928
##   P04   1.36193908
##   P05  -1.34164079
##   P06   1.96788736
```

```
chi.post.bottdisp.sim$residuals
```

```
##
##       General waste bin Recycling bin Recycling centre
##   P01       -0.30466273    0.05583645      -0.55200557
```

```
##   P02       -0.68934898    0.52741543      -0.50602431
##   P03       -0.71893855    0.60264061      -1.10679718
##   P04        0.19764878   -0.39512195       2.06036937
##   P05        0.97072371   -0.78607460       0.14907120
##   P06        0.39839133    0.16268789      -0.44177063
##
##       Specialist waste collection    Landfill Deposit return scheme
##   P01                 -0.18883154  0.14805502            1.57705468
##   P02                 -0.14422505 -0.90829511            0.41851107
##   P03                  0.15388554 -0.78262379           -0.70000000
##   P04                 -1.19791486 -0.02469324           -0.90553851
##   P05                  1.55011520  1.15950181           -0.84852814
##   P06                 -0.08160261  0.25558283            0.48260048
##
##       Indefinite storage       Other I don't know
##   P01        -0.45746624  1.11514606  -0.41533119
##   P02        -0.39389277 -0.57445626  -0.40620192
##   P03        -0.68571429  1.52533034  -0.35000000
##   P04         0.94971112 -0.64031242   1.75586126
##   P05         1.24922198 -0.60000000  -0.42426407
##   P06        -0.93980094 -0.55677644  -0.39370039
```

```
chi.post.tubspurchase.sim$residuals
```

```
##
##               None          1-2          3-5         6-10        11-15
##   P01 -0.048653363  0.780770639  1.073663763 -1.739935742 -0.001117755
##   P02 -0.696455673 -0.904918596  0.906850970 -0.273698508  1.012587961
##   P03 -0.387649466 -0.063233601 -1.318275797  2.661271866  0.237424754
##   P04  0.344291425  0.395297562 -0.612263801 -0.101812836  0.432689673
##   P05  0.571547607 -1.007012450  0.051247074  0.690268490 -0.533979494
##   P06  0.102652981  0.796782658 -0.247452621 -0.874718713 -1.176808243
##
##                16+ I don't know
##   P01 -0.161936787 -1.313392554
##   P02 -0.099004368 -0.506024314
##   P03 -1.261942946 -1.106797181
##   P04 -0.407354988  0.663508781
##   P05  1.085175948  0.149071198
##   P06  0.693902904  1.967887356
```

```
chi.post.tubdisp.sim$residuals
```

```
##
##       General waste bin Recycling bin Recycling centre
##   P01       -0.73605334    1.26014538      -0.44821959
##   P02        1.80782303   -0.30760503      -0.81601332
##   P03       -0.16483010   -0.16669802       1.29854097
##   P04       -0.62636804   -0.49491753      -0.38757511
##   P05       -0.12199886    0.16550941      -0.53397949
##   P06       -0.09038552   -0.47299897       1.18152491
##
##       Specialist waste collection    Landfill Deposit return scheme
```

```
##   P01                 -0.74370141  0.67072137           0.37319614
##   P02                 -1.40712473  0.71777575          -0.81240384
##   P03                  0.43713663 -0.60621778          -0.70000000
##   P04                  2.25702156 -0.78421936           1.30309201
##   P05                 -0.10886621  0.62598071           0.32998316
##   P06                 -0.63058259 -0.68190908          -0.78740079
##
##       Indefinite storage       Other I don't know
##   P01        -0.46254092 -1.09886305  -1.01734950
##   P02        -0.35327779 -0.14422505   0.01005038
##   P03        -0.50479239 -0.92601296   1.47552596
##   P04         0.81450982  1.30643676  -1.10905365
##   P05         0.16002645 -1.12249722   0.88527041
##   P06         0.19204614  1.83845883   0.07258662
```

```
chi.post.filmpurchase.sim$residuals
```

```
##
##              None         1-2         3-5        6-10       11-15         16+
##   P01  2.03649078 -0.39142817  0.42615962 -0.33804971  0.49060383 -0.62709231
##   P02 -0.69645567 -0.23100258  0.19390873  0.08221426  0.61693593 -0.56285109
##   P03  0.43713663 -0.40214440  0.01422705  1.38621939 -1.72824029  0.53538085
##   P04  0.34429143  2.11581622  0.19796041 -1.07313454 -1.41288739 -1.40035806
##   P05 -1.46969385 -0.54520517 -0.84504341  0.14634182  2.25700555  1.53333333
##   P06 -0.63058259 -0.83695373  0.02069649  0.11588752 -0.42502858  0.72440805
##
##       I don't know
##   P01  -1.43874946
##   P02   0.01421338
##   P03  -1.21243557
##   P04   0.98186814
##   P05  -0.78928003
##   P06   2.30235971
```

```
chi.post.filmreuse.sim$residuals
```

```
##
##             Never        Once   2-4 times  5-10 times  More often
##   P01 -0.58146471  0.18037197  0.55208821  0.64639468  0.25643882
##   P02  0.65062097 -0.72824540 -0.63987408  1.43555149 -0.93757155
##   P03  0.20272273  1.51553082 -1.79434844  0.43713663 -1.35554417
##   P04 -0.34217165 -0.97335121  1.48174377 -0.93086200  1.66802790
##   P05  0.23321224 -0.50191067  0.08981004 -0.10886621  0.18257419
##   P06 -0.09589725  0.87404270 -0.12789071 -1.36381817 -0.21314340
```

```
chi.post.filmdisp.sim$residuals
```

```
##
##       General waste bin Recycling bin Recycling centre
##   P01       -0.10538223    0.03263284       0.62337427
##   P02       -0.10964103    0.98045457      -1.67481342
##   P03        0.97190263   -0.08042699      -1.44308697
```

```
##   P04       -1.26517026    0.72303414       1.34721403
##   P05        0.33301872   -1.12055887       0.53736223
##   P06        0.45639101   -0.59847867       0.22485500
##
##       Specialist waste collection    Landfill Deposit return scheme
##   P01                 -0.03440312 -0.71937473           -0.58736701
##   P02                  0.01005038 -0.70356236           -0.57445626
##   P03                 -0.85732141 -0.60621778           -0.49497475
##   P04                  0.69428562  0.49093407            0.92142520
##   P05                  0.88527041  0.62598071            1.06666667
##   P06                 -0.96436508  0.78456207           -0.55677644
##
##       Indefinite storage       Other I don't know
##   P01        -0.71937473  0.94854325  -0.03440312
##   P02        -0.70356236  1.01508819   0.01005038
##   P03        -0.60621778 -0.85732141  -0.85732141
##   P04         1.76608750 -0.20738402   0.69428562
##   P05        -0.73484692 -1.03923048   0.88527041
##   P06         0.78456207  0.07258662  -0.96436508
```

```
chi.post.bagpurchase.sim$residuals
```

```
##
##              None         1-2         3-5        6-10       11-15         16+
##   P01 -0.47738460  0.19687111 -0.15329490  0.95145418  1.66342023 -0.32347748
##   P02 -0.04525557  0.92441734 -0.41109095 -0.07624564 -0.86191672 -1.14891253
##   P03  0.29637738  0.83598223 -0.55226268 -0.21466758 -1.30958009  0.02020305
##   P04  0.35380902  0.05411481 -0.27450259 -0.45349166  0.07673657 -1.28062485
##   P05 -0.89133762 -0.23100258  1.89066796  0.31362808  0.93231237  0.46666667
##   P06  0.84046706 -1.71794601 -0.64493235 -0.55066714 -0.79424775  2.47855317
##
##       I don't know
##   P01  -1.09886305
##   P02   0.78625916
##   P03  -0.92601296
##   P04   0.47165289
##   P05  -1.12249722
##   P06   1.83845883
```

```
chi.post.bagreuse.sim$residuals
```

```
##
##            Never       Once  2-4 times 5-10 times More often
##   P01  0.7146050  0.8747746  1.4874009 -1.1726922 -1.1246045
##   P02 -1.3773212 -0.8387647  0.6777680  0.1324102  0.4390625
##   P03  0.1039145  1.1242066 -0.4125586 -0.8793468  0.2592725
##   P04  1.0492232 -2.0044407 -2.0761054  1.4914974  1.1782443
##   P05 -1.0922011  1.4497649 -0.4252766  0.6761234 -0.4000000
##   P06  0.5451565 -0.3139942  0.9442388 -0.5616385 -0.4210747
```

```
chi.post.bagdisp.sim$residuals
```

```
##
##       General waste bin Recycling bin Recycling centre
##   P01       -0.53708356   -0.21838901       1.78728958
##   P02        1.11271952   -0.55952643      -0.81601332
##   P03        1.54883967    0.81320621      -0.82369146
##   P04       -2.27451847    0.03223719      -0.38757511
##   P05        0.24835121   -0.87482227      -0.09629138
##   P06        0.38976443    0.99040275       0.23819165
##
##       Specialist waste collection    Landfill Deposit return scheme
##   P01                  1.37903558 -0.58736701            0.07441029
##   P02                 -1.14891253 -0.57445626            0.87958950
##   P03                 -0.98994949 -0.49497475           -1.16081868
##   P04                  1.84285039 -0.64031242           -0.83573858
##   P05                 -1.20000000  2.73333333            2.14622054
##   P06                 -0.21552636 -0.55677644           -1.30575649
##
##       Indefinite storage       Other I don't know
##   P01        -0.61247187  0.01631642   0.35915113
##   P02         0.62285924 -1.94807597   0.42261412
##   P03        -0.67975438 -1.67854103  -0.09761905
##   P04         1.93579313  1.97337662   0.11411258
##   P05        -0.76062752  1.40561332  -0.48711800
##   P06        -0.79876630 -0.29923933  -0.33443367
```

Simulated observed and expected values

```
chi.post.choice.sim$observed
```

```
##
##       Always As often as they can If cheaper or preferred Rarely or never
##   P01      8                   31                      23               7
##   P02      3                   42                      19               2
##   P03      4                   30                      14               1
##   P04     14                   41                      22               5
##   P05      9                   37                      20               6
##   P06      8                   27                      18               9
```

```
chi.post.barrier.sim$observed
```

```
##
##       Council collection Unclear information Difficult transport
##   P01                 19                  11                   3
##   P02                 22                   8                   8
##   P03                 16                   6                   3
##   P04                 22                  19                   8
##   P05                 20                   7                   5
##   P06                 16                  11                   4
##
##       No local facilities No support Ends up in landfills Forgetting
##   P01                   7          3                    9          1
##   P02                   4          4                    1          5
```

```
##   P03                  4         2                 8         4
##   P04                  5         6                 4         7
##   P05                  9         2                 8         4
##   P06                  6         1                 3         4
##
##       Recycling a hassle Household disagrees Recycling not important Other
##   P01                  2                   0                       1     1
##   P02                  1                   1                       0     1
##   P03                  1                   0                       0     0
##   P04                  1                   0                       1     0
##   P05                  2                   2                       1     1
##   P06                  3                   2                       2     1
##
##       Already doing everything
##   P01                       12
##   P02                       11
##   P03                        5
##   P04                        9
##   P05                       11
##   P06                        9
```

```
chi.post.barrier2.sim$observed
```

```
##
##       Limited alternatives No SUP-free alternatives Alternatives expensive
##   P01                   20                       15                     11
##   P02                   13                       13                     16
##   P03                    8                       11                     14
##   P04                   18                       16                     14
##   P05                   13                       20                     15
##   P06                   18                       10                     15
##
##       Limited functioning Forgetting reusables Reducing not important Other
##   P01                  10                    5                      2     0
##   P02                   5                   11                      3     2
##   P03                   4                    4                      0     1
##   P04                   7                    7                      4     1
##   P05                   6                    9                      4     2
##   P06                   7                    4                      0     2
##
##       No barriers
##   P01           6
##   P02           3
##   P03           7
##   P04          15
##   P05           3
##   P06           6
```

```
chi.post.mainconsid.sim$observed
```

```
##
##       Value for money Price Quality Deals/discounts Use-by-dates/longevity
##   P01              22    16      12               6                      5
```

```
##   P02              19    17       8               6                      3
##   P03              18     9      13               2                      1
##   P04              21    25      16               6                      0
##   P05              25    15      20               3                      2
##   P06              14    15      19               3                      3
##
##       Convenience Ease of recycling packaging Sustainability Brand Ethics Other
##   P01           2                            1              3     1      1     0
##   P02           2                            7              2     1      1     0
##   P03           0                            4              0     1      1     0
##   P04           2                            4              3     1      2     2
##   P05           1                            3              3     0      0     0
##   P06           3                            3              2     0      0     0
```

```
chi.post.litter.sim$observed
```

```
##
##       Strongly agree Agree Neither agree nor disagree Disagree
##   P01             26    31                          8        1
##   P02             37    20                          9        0
##   P03             19    22                          7        1
##   P04             37    31                         11        2
##   P05             25    36                          8        3
##   P06             29    22                         10        1
##
##       Strongly disagree
##   P01                 3
##   P02                 0
##   P03                 0
##   P04                 1
##   P05                 0
##   P06                 0
```

```
chi.post.litter.sim$expected
```

```
##
##       Strongly agree  Agree Neither agree nor disagree Disagree
##   P01        29.8425 27.945                     9.1425     1.38
##   P02        28.5450 26.730                     8.7450     1.32
##   P03        21.1925 19.845                     6.4925     0.98
##   P04        35.4650 33.210                    10.8650     1.64
##   P05        31.1400 29.160                     9.5400     1.44
##   P06        26.8150 25.110                     8.2150     1.24
##
##       Strongly disagree
##   P01              0.69
##   P02              0.66
##   P03              0.49
##   P04              0.82
##   P05              0.72
##   P06              0.62
```

```
chi.post.attitude.sim$observed
```

```
##
##       1 (Not concerned)  2  3  4  5  6 7 (Very concerned
##   P01                 5  2  9  9 15 11                18
##   P02                 5  3  9 13 12 10                14
##   P03                 2  2  3 12 16  8                 6
##   P04                 4  5  5 20 25 10                13
##   P05                 4  6  7  8 26  9                12
##   P06                 7  2  8 11 20  8                 6
```

```
chi.post.attitude.sim$expected
```

```
##
##       1 (Not concerned)    2      3       4      5     6 7 (Very concerned
##   P01            4.6575 3.45 7.0725 12.5925 19.665  9.66           11.9025
##   P02            4.4550 3.30 6.7650 12.0450 18.810  9.24           11.3850
##   P03            3.3075 2.45 5.0225  8.9425 13.965  6.86            8.4525
##   P04            5.5350 4.10 8.4050 14.9650 23.370 11.48           14.1450
##   P05            4.8600 3.60 7.3800 13.1400 20.520 10.08           12.4200
##   P06            4.1850 3.10 6.3550 11.3150 17.670  8.68           10.6950
```

```
chi.post.ocean.sim$observed
```

```
##
##       Always Most of the time Sometimes Rarely Never
##   P01      5               19        31     11     3
##   P02      3               18        34      9     2
##   P03      0               14        28      5     2
##   P04      6               22        38     11     5
##   P05      4               17        37     12     2
##   P06      1               14        32     12     3
```

```
chi.post.ocean.sim$expected
```

```
##
##       Always Most of the time Sometimes Rarely  Never
##   P01 3.2775            17.94      34.5  10.35 2.9325
##   P02 3.1350            17.16      33.0   9.90 2.8050
##   P03 2.3275            12.74      24.5   7.35 2.0825
##   P04 3.8950            21.32      41.0  12.30 3.4850
##   P05 3.4200            18.72      36.0  10.80 3.0600
##   P06 2.9450            16.12      31.0   9.30 2.6350
```

```
chi.post.actions.sim$observed
```

```
##
##       Yes, definitely Yes, probably No, probably not No, definitely not
##   P01              21            31               12                  2
##   P02              30            22               10                  1
```

```
## P03             19          25              3                 1
## P04             22          43              9                 6
## P05             27          38              5                 0
## P06             23          32              4                 1
##
##     I don't know
## P01            3
## P02            3
## P03            1
## P04            2
## P05            2
## P06            2
```

```
chi.post.actions.sim$expected
```

```
##
##     Yes, definitely Yes, probably No, probably not No, definitely not
## P01          24.495       32.9475          7.4175             1.8975
## P02          23.430       31.5150          7.0950             1.8150
## P03          17.395       23.3975          5.2675             1.3475
## P04          29.110       39.1550          8.8150             2.2550
## P05          25.560       34.3800          7.7400             1.9800
## P06          22.010       29.6050          6.6650             1.7050
##
##     I don't know
## P01       2.2425
## P02       2.1450
## P03       1.5925
## P04       2.6650
## P05       2.3400
## P06       2.0150
```

```
chi.post.attplast.sim$observed
```

```
##
##     Will go out of their way to avoid
## P01                                20
## P02                                15
## P03                                15
## P04                                20
## P05                                17
## P06                                15
##
##     If option is readily available, will avoid
## P01                                         33
## P02                                         37
## P03                                         31
## P04                                         43
## P05                                         38
## P06                                         36
##
##     Will avoid only without extra costs Not a priority
## P01                                  14              2
```

```
##   P02                                    10             4
##   P03                                     2             1
##   P04                                    13             6
##   P05                                    13             4
##   P06                                     6             5
```

```
chi.post.attplast.sim$expected
```

```
##
##       Will go out of their way to avoid
##   P01                            17.595
##   P02                            16.830
##   P03                            12.495
##   P04                            20.910
##   P05                            18.360
##   P06                            15.810
##
##       If option is readily available, will avoid
##   P01                                      37.605
##   P02                                      35.970
##   P03                                      26.705
##   P04                                      44.690
##   P05                                      39.240
##   P06                                      33.790
##
##       Will avoid only without extra costs Not a priority
##   P01                              10.005          3.795
##   P02                               9.570          3.630
##   P03                               7.105          2.695
##   P04                              11.890          4.510
##   P05                              10.440          3.960
##   P06                               8.990          3.410
```

```
chi.post.zerow.sim$observed
```

```
##
##       Yes, shops regularly Yes, shops occasionally Yes, visited at least once
##   P01                    3                       3                          5
##   P02                    0                       1                          3
##   P03                    1                       4                          1
##   P04                    2                       6                          6
##   P05                    6                       9                          6
##   P06                    2                       7                          3
##
##       Yes, never visited No, but would like to shop there
##   P01                 15                               30
##   P02                 11                               33
##   P03                 12                               23
##   P04                 22                               40
##   P05                 22                               23
##   P06                  6                               26
##
##       No, not likely to shop there
```

```
## P01                          13
## P02                          18
## P03                           8
## P04                           6
## P05                           6
## P06                          18
```

```
chi.post.zerow.sim$expected
```

```
##
##       Yes, shops regularly Yes, shops occasionally Yes, visited at least once
##   P01                2.415                   5.175                       4.14
##   P02                2.310                   4.950                       3.96
##   P03                1.715                   3.675                       2.94
##   P04                2.870                   6.150                       4.92
##   P05                2.520                   5.400                       4.32
##   P06                2.170                   4.650                       3.72
##
##       Yes, never visited No, but would like to shop there
##   P01              15.18                          30.1875
##   P02              14.52                          28.8750
##   P03              10.78                          21.4375
##   P04              18.04                          35.8750
##   P05              15.84                          31.5000
##   P06              13.64                          27.1250
##
##       No, not likely to shop there
##   P01                      11.9025
##   P02                      11.3850
##   P03                       8.4525
##   P04                      14.1450
##   P05                      12.4200
##   P06                      10.6950
```

```
chi.post.bottpurchase.sim$observed
```

```
##
##       None 1-2 3-5 6-10 11-15 16+ I don't know
##   P01    1  24  21   15     6   2            0
##   P02    0  21  26   13     5   0            1
##   P03    7  18  13    7     2   1            1
##   P04    2  26  31   14     3   2            4
##   P05    1  29  24   13     2   3            0
##   P06    1  19  19   12     1   6            4
```

```
chi.post.bottdisp.sim$observed
```

```
##
##       General waste bin Recycling bin Recycling centre
##   P01                 4            57                1
##   P02                 3            58                1
##   P03                 2            44                0
```

```
## 	P04		6		64		5
## 	P05		7		53		2
## 	P06		5		52		1
##
##     Specialist waste collection Landfill Deposit return scheme
##   P01                           1        1                    2
##   P02                           1        0                    1
##   P03                           1        0                    0
##   P04                           0        1                    0
##   P05                           3        2                    0
##   P06                           1        1                    1
##
##     Indefinite storage Other I don't know
##   P01                  2     1            0
##   P02                  2     0            0
##   P03                  1     1            0
##   P04                  5     0            1
##   P05                  5     0            0
##   P06                  1     0            0
```

```
chi.post.tubspurchase.sim$observed
```

```
##
##     None 1-2 3-5 6-10 11-15 16+ I don't know
##   P01    2  22  30    8     5   2            0
##   P02    1  14  28   13     7   2            1
##   P03    1  13  12   19     4   0            0
##   P04    3  24  26   17     7   2            3
##   P05    3  15  26   18     4   4            2
##   P06    2  20  21   10     2   3            4
```

```
chi.post.tubdisp.sim$observed
```

```
##
##     General waste bin Recycling bin Recycling centre
##   P01                12            44                4
##   P02                21            33                3
##   P03                10            25                6
##   P04                15            40                5
##   P05                15            39                4
##   P06                13            30                7
##
##     Specialist waste collection Landfill Deposit return scheme
##   P01                           1        1                    1
##   P02                           0        1                    0
##   P03                           2        0                    0
##   P04                           6        0                    2
##   P05                           2        1                    1
##   P06                           1        0                    0
##
##     Indefinite storage Other I don't know
##   P01                  6     0            0
##   P02                  6     1            1
```

```
##   P03                   4     0            2
##   P04                  11     3            0
##   P05                   8     0            2
##   P06                   7     3            1
```

```
chi.post.filmpurchase.sim$observed
```

```
##
##       None 1-2 3-5 6-10 11-15 16+ I don't know
##   P01    5  13  23   18     8   2            0
##   P02    1  13  21   19     8   2            2
##   P03    2   9  15   19     1   3            0
##   P04    3  26  26   18     4   1            4
##   P05    0  13  18   21    13   6            1
##   P06    1  10  19   18     5   4            5
```

```
chi.post.filmreuse.sim$observed
```

```
##
##       Never Once 2-4 times 5-10 times More often
##   P01    37   15        11          3          3
##   P02    43   11         7          4          1
##   P03    30   15         2          2          0
##   P04    46   13        16          1          6
##   P05    44   13        10          2          3
##   P06    36   16         8          0          2
```

```
chi.post.filmdisp.sim$observed
```

```
##
##       General waste bin Recycling bin Recycling centre
##   P01                45            16                4
##   P02                43            19                0
##   P03                38            11                0
##   P04                45            22                6
##   P05                50            12                4
##   P06                44            12                3
##
##       Specialist waste collection Landfill Deposit return scheme
##   P01                           1        0                     0
##   P02                           1        0                     0
##   P03                           0        0                     0
##   P04                           2        1                     1
##   P05                           2        1                     1
##   P06                           0        1                     0
##
##       Indefinite storage Other I don't know
##   P01                  0     2            1
##   P02                  0     2            1
##   P03                  0     0            0
##   P04                  2     1            2
##   P05                  0     0            2
##   P06                  1     1            0
```

```r
chi.post.bagpurchase.sim$observed
```

```
##
##       None 1-2 3-5 6-10 11-15 16+ I don't know
##   P01   37  14   7    5     5   1            0
##   P02   38  16   6    3     1   0            2
##   P03   30  12   4    2     0   1            0
##   P04   50  16   8    3     3   0            2
##   P05   36  13  13    4     4   2            0
##   P06   41   6   5    2     1   4            3
```

```r
chi.post.bagreuse.sim$observed
```

```
##
##       Never Once 2-4 times 5-10 times More often
##   P01     7   10        22          8         22
##   P02     2    5        18         12         29
##   P03     4    8        10          6         21
##   P04     9    3        10         20         40
##   P05     3   12        15         15         27
##   P06     6    6        18          9         23
```

```r
chi.post.bagdisp.sim$observed
```

```
##
##       General waste bin Recycling bin Recycling centre
##   P01                22            15                9
##   P02                29            13                3
##   P03                24            14                2
##   P04                17            19                5
##   P05                27            13                5
##   P06                24            18                5
##
##       Specialist waste collection Landfill Deposit return scheme
##   P01                           3        0                     2
##   P02                           0        0                     3
##   P03                           0        0                     0
##   P04                           4        0                     1
##   P05                           0        2                     5
##   P06                           1        0                     0
##
##       Indefinite storage Other I don't know
##   P01                 12     4            2
##   P02                 16     0            2
##   P03                  8     0            1
##   P04                 25     9            2
##   P05                 12     7            1
##   P06                 10     3            1
```
